# Bibliometric Study of Pain after Spinal Cord Injury

**DOI:** 10.1155/2021/6634644

**Published:** 2021-02-19

**Authors:** Yi-Zu Wang, Cheng-Cheng Wu, Xue-Qiang Wang

**Affiliations:** ^1^Department of Sport Rehabilitation, Shanghai University of Sport, 399 Changhai RD, Shanghai 200438, China; ^2^Department of Rehabilitation Medicine, Shanghai Shangti Orthopaedic Hospital, 188 Hengren RD, Shanghai 200438, China

## Abstract

**Background:**

The prevalence of comorbid pain after spinal cord injury (SCI) is relatively high in clinical observations and has continued to increase over time. Neuropathic pain (70.14%) is the most popular subject in academic journals after SCI. However, studies that used the bibliometric method to analyze comorbid pain after SCI are still lacking. This study is aimed at combining and integrating acquired information to analyze the global trends of research on the comorbidity of pain after SCI in the last three decades (1990–2019).

**Methods:**

Systematic works of literature published from 1990 to 2019 were obtained from the Web of Science Core Collection. CiteSpace software was used to analyze the relationship of publication year with the country, institution, journals, authors, references, and keywords. The regression analysis is used to evaluate the percentage of the category increase or decrease over time significantly. IBM SPSS Statistics was used in the statistical analysis.

**Results:**

A total of 730 publications were included in the analysis. A remarkable increase in the number of publications was observed in the study period (*P* < 0.05). A total of 202 academic journals focused on the categories of clinical neurology, neurosciences, and rehabilitation, and the annual growth rate of articles in these three categories was statistically significant (*P* < 0.05). The USA (356, 48.77%) and the University of Miami (64, 8.77%) were the country and institution with the highest number of publications, respectively. *Spinal Cord*, which was the main journal for research on pain after SCI, had the most publications (88, 12.05%). Burst keywords showed that the individual, inflammation, and central sensitization with pain after SCI are the research development trends and focus in this research field.

**Conclusions:**

Overall, this study provides the latest research direction for pain after SCI. This historical overview of research into pain after SCI will be a useful basis for further research into development trends, focus issues, cooperators, and cooperative institutions.

## 1. Introduction

Pain is a frequent complication of spinal cord injury (SCI), and approximately half to two-thirds of patients with SCI suffer from pain; pain after SCI is typically chronic and neuropathic [1–3]. The male-to-female ratio of SCI is high. Most cases are caused by traffic accidents, falls, sport activities, or violence [4]. SCI causes permanent disabilities and brings a heavy burden to people's quality of life, level of functioning, and chances of returning to work [5]. Pain after SCI manifests in many ways. More than 50% of patients with SCI suffered chronic pain within 1 year after SCI [6]. The presence of chronic pain seriously affects the patients' daily life and the social impact. Chronic pain in SCI is related to great emotional distress, and pain hinders the ability of SCI patients to participate in active rehabilitation programs [7]. Acute pain is accompanied by injury and recovery and subsides as tissue scars fade, whereas chronic pain occurs because of poor neuroplasticity [8, 9]. Pain after SCI is difficult to treat because the underlying mechanisms are not yet fully understood. At present, the larger proportion of patients with SCI in most countries and regions is under 30 years old, and the highest incidence rate of 49 every 1 million was recorded in New Zealand [10]. Pain after SCI also brings a medical and economic burden to the government. People with pain after SCI in Canada spend more than US$2.67 billion annually [11].

In view of the high incidence of pain after SCI, a growing number of researchers have studied pain after SCI, and relevant articles have been published in academic journals. A number of randomized controlled trials have studied the treatment of pain after SCI [12–15]. Scientific research studies increasingly apply bibliometrics in quantitative analysis [16–18]. Bibliometric analysis revealed that research on the application of stem cells in SCI is a rapidly developing research field [19]. However, a quantitative analysis of pain associated with SCI has not yet been conducted.

To address the shortage of quantitative analysis of pain after SCI research, the purpose of this study is to provide a basis for the global scientific research on pain after SCI over the last 30 years (1990–2019). By being able to understand the types of pain after SCI in the past 30 years, current research hotspots provide a theoretical basis for follow-up research. Papers using CiteSpace are on the rise, especially in the medical field, where there have been many related studies [20–22]. CiteSpace 5.6.R5 (Drexel University, Philadelphia, USA) is a commonly used software application for bibliometric analysis. The global research trend on pain after SCI includes four aspects: the number of published papers; the distribution and cooperation between authors, institutions, and countries; a citation burst analysis of keywords; and the cocitation analysis of authors and references.

## 2. Methods

### 2.1. Search Strategy

The publications included in this study were those published within the last 30 years (1990–2019). The publications were downloaded from the Science Citation Index Expanded (SCI-Expanded) Web of Science (WoS). The search strategy was as follows: ([TI = spinal cord injury] OR [TI = spinal cord injuries]) AND ([TI = pain] OR [TI = painful]).

### 2.2. Inclusion Criteria and Exclusion Criteria

Publications related to pain and SCI, such as articles, reviews, letters, and editorial materials, published in different academic journals were included. 872 articles were identified from the Web of Science Core Collection. Conference presentations, meeting abstracts, book reviews, news items, and corrections were excluded. After excluding 136 articles, 736 articles were included. No other specific limitation was imposed except that the chosen language was English. Six non-English language papers were excluded. Finally, 730 articles were included.

### 2.3. Data Extraction

We followed a previous search strategy to search through the WoS database and then imported the gathered information to CiteSpace for analysis [22–25]. We obtained bibliometric indicators by calculating the number of publications, journals, references, citations, extracted *H*-index, and keywords. The *H*-index is a mixed quantitative index that can be used to evaluate the amount and level of the academic output of researchers. The *H*-index means that an academic journal or researcher has at least *H* published papers that have at least *H* citation times per paper. For instance, an *H*-index of 20 indicates that academic journals or researchers had at least published 20 papers, and the citation frequency is at least 20.

### 2.4. Statistical Methods

We used CiteSpace 5.6.R5 and Microsoft Excel 2016 to extract and analyze the number of publications (including different journals, countries, institutions, and authors), citation frequency, and keyword trends. We visualized the structure, regular pattern, and distribution of scientific knowledge using CiteSpace and Microsoft Excel:

1. Analysis of the distribution and trend of journals, countries, institutions, and authors
2. Analysis of the number of papers, citations, citations per paper, and open-access papers and *H*-index in the top 10 journals
3. Assess country-to-country cooperation/institutions/authors
4. Citation analysis and *H*-index refer to the number of published papers or research *H* and at least *H* paper quality
5. Analysis of citations and keywords
6. Cocitation analysis according to references, cited authors, and cited journals
7. Cooccurrence analysis of terms, keywords, sources, and categories

Besides, we calculated the number of single-author and multiauthor publications, the frequency of WoS subject categories, and types of pain category ranking percentage scores annually (the number of publications every year divided by the total number of publications in each category). The regression analysis is used to evaluate the percentage of the category increase or decrease over time significantly (the category as the dependent variable and the year as the independent variable). SPSS Statistics 22.0 software (Chicago, USA) was used for statistical analysis. *P* < 0.05 was considered statistically significant.

## 3. Results

### 3.1. Publication Output and Growth Trends

**Figure 1:**
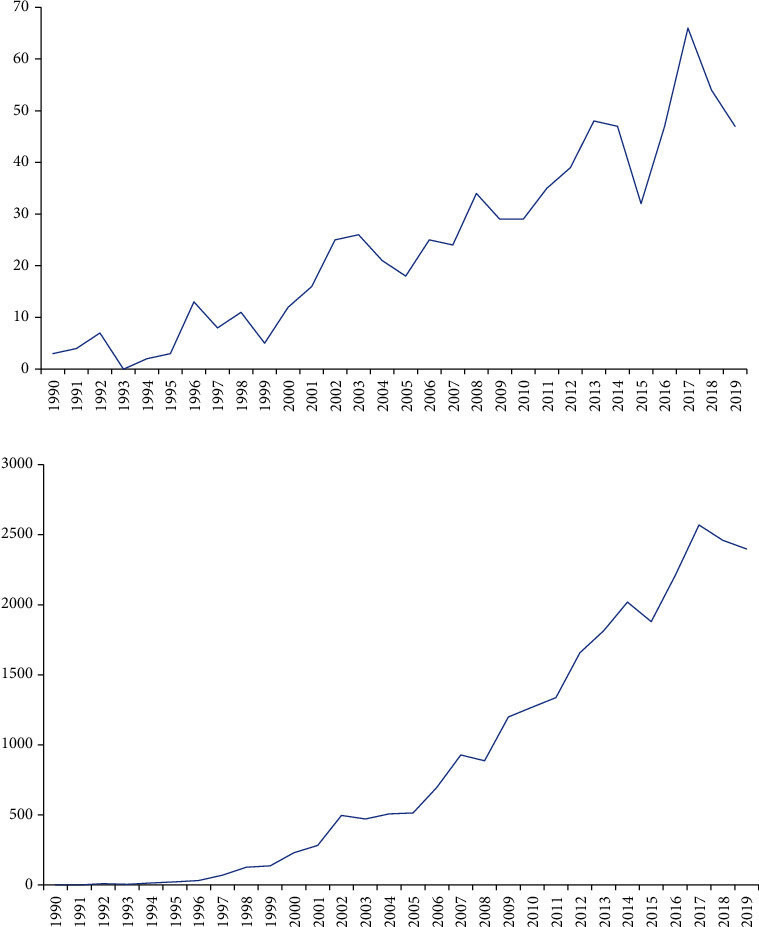
Number of publications and citations. (a) The number of annual publications on pain after spinal cord injury research from 1990 to 2019. (b) The number of annual citations on pain after spinal cord injury research from 1990 to 2019.

**Figure 2:**
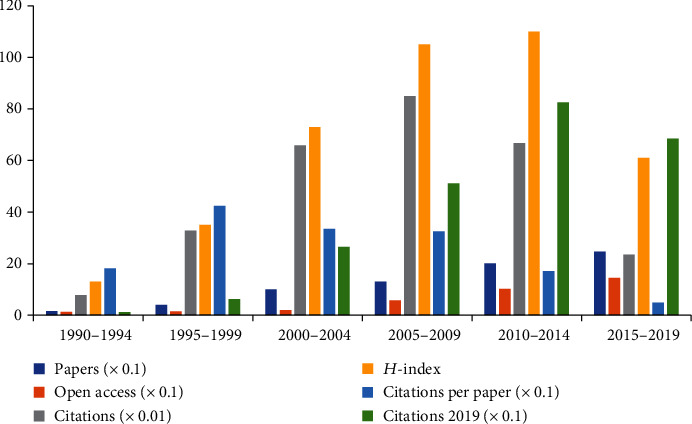
Number of papers, citations, citations per paper, open-access papers, and citations in 2019 and *H*-index for each 5-year time period.

A total of 730 articles were included (Supplementary Figure 1). According to Figure 1(a), although the number of publications has increased and decreased over the past 30 years, the overall trend has continued to increase. The initial three publications increased to 47 publications from 1990 to 2019 (Figure 1(a)). The number of articles published in 2017 was 66. The results of linear regression analysis indicated that the number of articles published increased significantly with time over the last 30 years (*t* = 14.762, *P* < 0.001). The number of citations increased from 0 citations in 1990 to 2397 citations in 2019. A total of 26,232 citations are cited in all the papers with an average of 874.4 times per year.  Figure 1(b) shows that the number of article citations had a significant increase over time (*t* = 17.066, *P* < 0.001). We divided the study period of 30 years into six groups (1990–1994, 1995–1999, 2000–2004, 2005–2009, 2010–2014, and 2015–2019). The largest number of citations per paper was 423.89 from 1995 to 1999. The highest *H*-index value was 110, and the most cited papers were from 2010 to 2014 (826). The most published papers (247) and the highest number of open-access papers (145) were recorded in 2015–2019 (Figure 2). And the results of linear regression analysis of the *H*-index value and the number of open-access papers also have a significant increase with time over the last 30 years (*t* = 4.252, *P* < 0.001; *t* = 8.823, *P* < 0.001).

### 3.2. Distribution by Journals

Supplementary Table 1 shows that 730 articles were selected through WoS screening, and these 730 articles were published in 202 academic journals. We selected the top 20 of these 202 academic journals according to the number of publications (Table 1). The total number of published articles in the top 20 academic journals exceeded half of the total number of articles (58.91%). The academic journal *Spinal Cord* had published the largest number of articles (88 publications, 12.06%), and its impact factor (IF) is 1.773. *Pain*, which has an IF of 5.483, contributed to the second most published articles (60 publications, 8.22%). The *Archives of Physical Medicine and Rehabilitation* (IF 2019, 3.098; 41 publications, 5.62%), *Journal of Neurotrauma* (IF 2019, 3.793; 33 publications, 4.52%), and *Journal of Spinal Cord Medicine* (IF 2019, 1.816; 28 publications, 3.84%) ranked the third to fifth, respectively, in terms of the number of publications. The *Pain* journal had the highest number of citations (4845) and the highest *H*-index value (37). *Neurology* had the highest IF amongst the top 20 journals (IF 2019, 8.77), and *Journal of Neuroscience* had the largest number of citations per paper (116.36). In accordance with the journal IF quartile of WoS, 35% of the 20 journals were in the first quartile (Q1), and 45% of the journals were in the second quartile (Q2).

**Figure 3:**
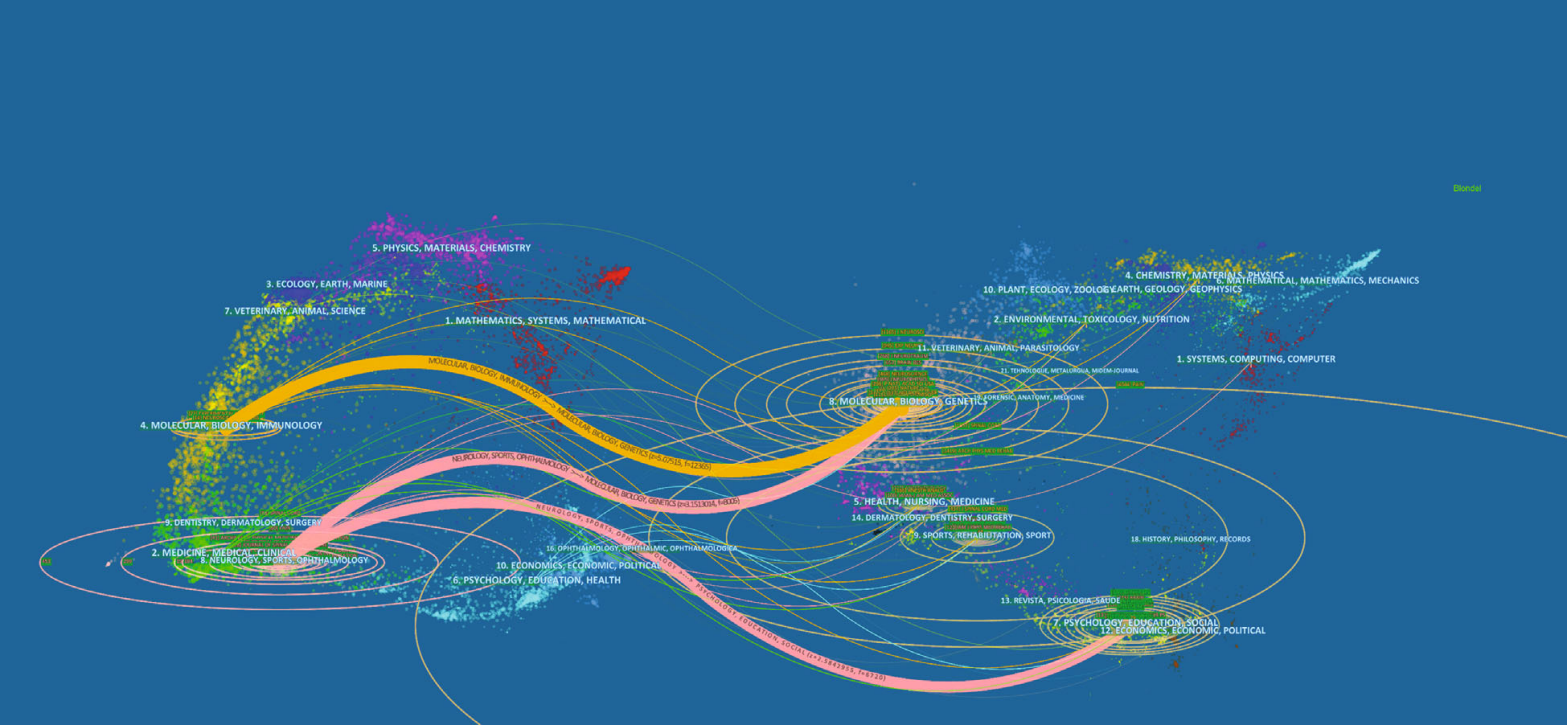
The dual-map overlay of journals related to pain after spinal cord injury.

Figure 3 indicates the dual map of the journal. The map on the left represents the citing journals, and the map on the right represents the cited journals. In the dual-map overlay, the labels are marked according to the disciplines of the subject. A line connects the citing journal on the left side to the cited journal on the right side. The dual map indicates that most of the journals were from the molecular, biology, and immunology fields. Simultaneously, most journals were cited from the molecular, biology, and genetics fields.

### 3.3. Subject Categories of WoS

**Figure 4:**
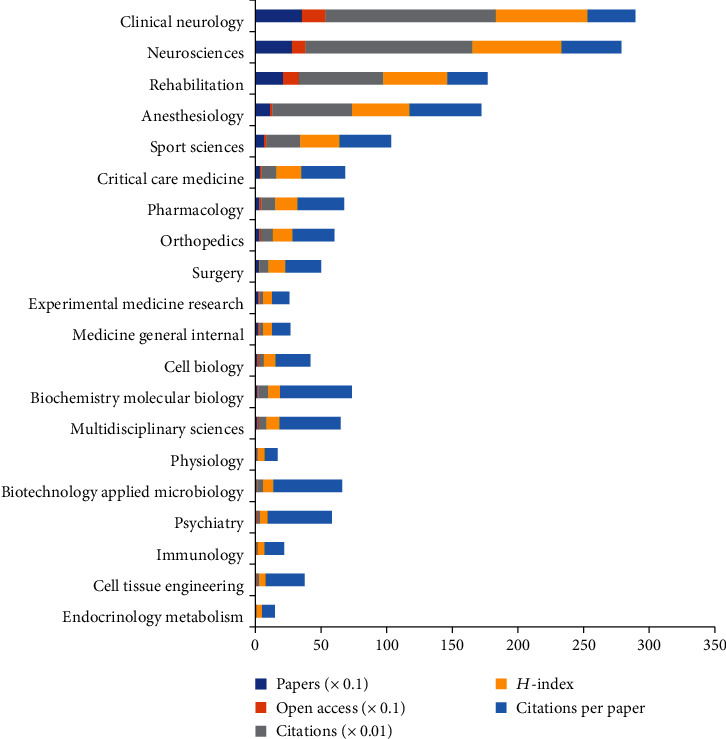
The number of papers, citations, citations per paper, and open-access papers and *H*-index of the top 20 subject categories of Web of Science.

We classified the 730 articles into the 51 subject categories of WoS and ranked the top 20 journals on the basis of the number of publications (Figure 4). Anesthesiology was the subject category with the largest number of citations per paper (54.85). Clinical neurology had the largest number of publications (355), citations (13,008), and open-access papers (181) and the highest *H*-index (70).

### 3.4. Types of Pain

**Figure 5:**
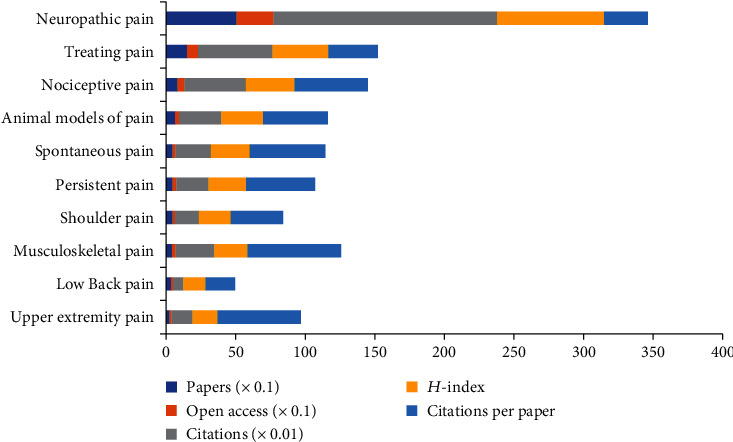
The number of papers, citations, citations per paper, and open-access papers and *H*-index of the top 10 types of pain.

The top 10 types of pain after SCI were ranked as shown in Figure 5. Neuropathic pain (70.14%) is the most popular subject after SCI. Moreover, neuropathic pain had the highest number of publications (512), citations (16,045), and open-access papers (268) and the highest *H*-index value (77). Notably, average musculoskeletal muscle pain had the most citations per article (67.17).

### 3.5. Distribution by Countries and Institutions

**Figure 6:**
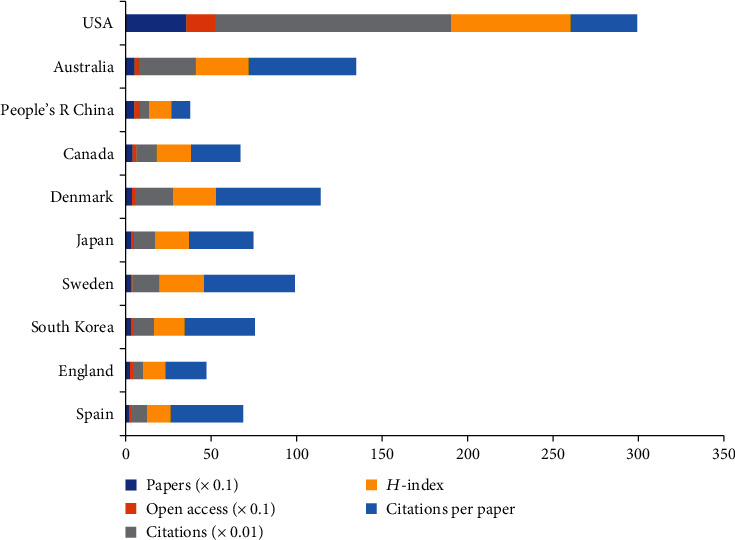
The number of papers, citations, citations per paper, and open-access papers and *H*-index of the top 10 countries.

**Figure 7:**
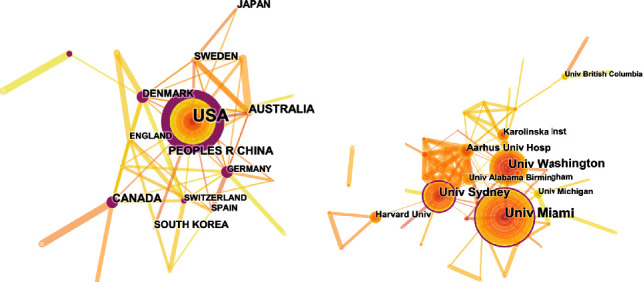
The analysis of countries and institutions. (a) Network map of countries/territories engaged in pain after spinal cord injury. (b) Network map of institutions engaged in pain after spinal cord injury.

**Figure 8:**
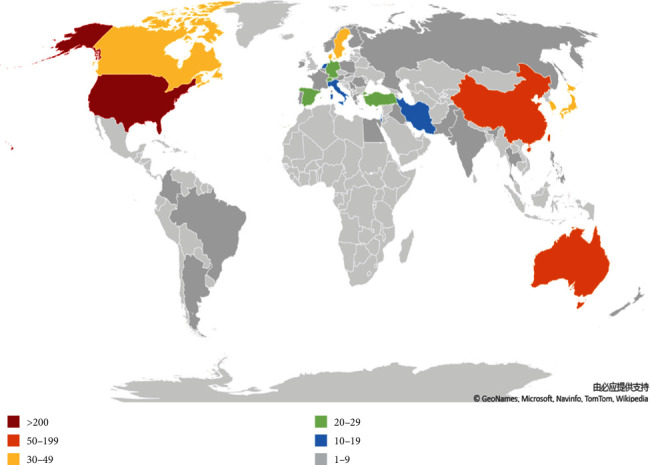
World map of the total country output based on pain after spinal cord injury.

The 730 articles on pain after SCI were contributed by 42 countries or regions (Supplementary Table 2).  Figure 6 shows the top 10 countries or regions according to the number of publications. The United States of America (USA) had the highest number of publications (356), citations (13,874), and open-access papers (168) and the highest *H*-index value (70), followed by Australia (53), which had the most citations per paper (63.06), and China (53).  Figure 7(a) indicates that the contributing countries/regions have extensive and close cooperation and contact. The countries or regions of the included 730 articles are presented in the world map in Figure 8.

A total of 795 institutions (Supplementary Table 3) contributed to 730 papers on pain after SCI. Supplementary Figure 2 indicates the top 10 institutions in terms of the number of published papers. The University of Miami had the largest number of publications (64) and open-access papers (64) and the highest *H*-index (31). The University of Seattle and the University of Washington had the largest number of citations (2605). The University of Texas Medical Branch Galveston had the largest number of citations per paper (81.56).  Figure 7(b) indicates the collaborations between institutions. The University of Miami and the University of Sydney had a strong partnership.

### 3.6. Distribution by Authors

**Figure 9:**
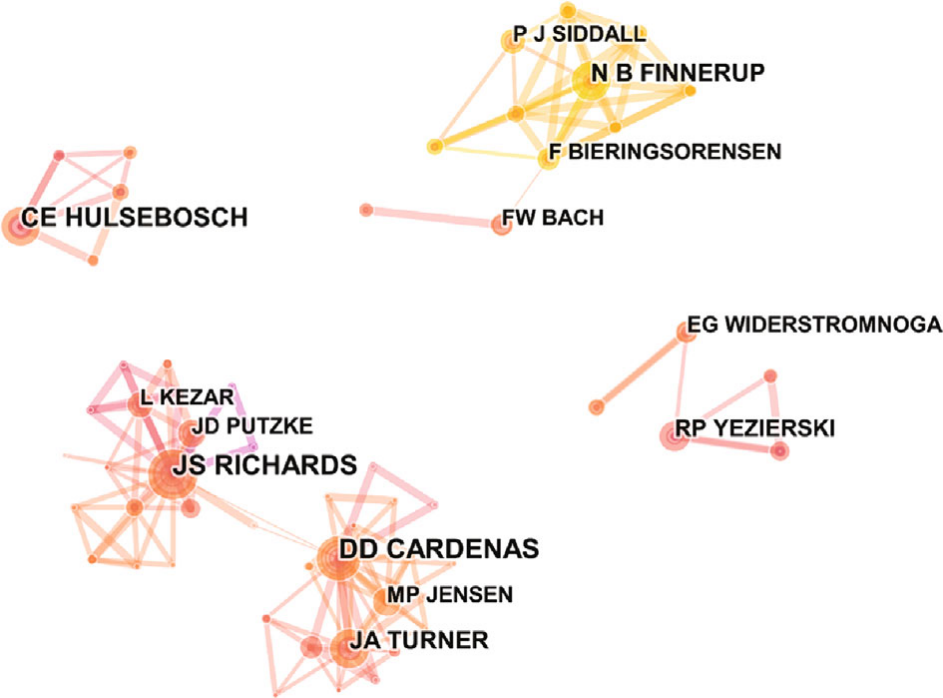
The analysis of authors. Network map of active authors that contributed to pain after spinal cord injury.

**Figure 10:**
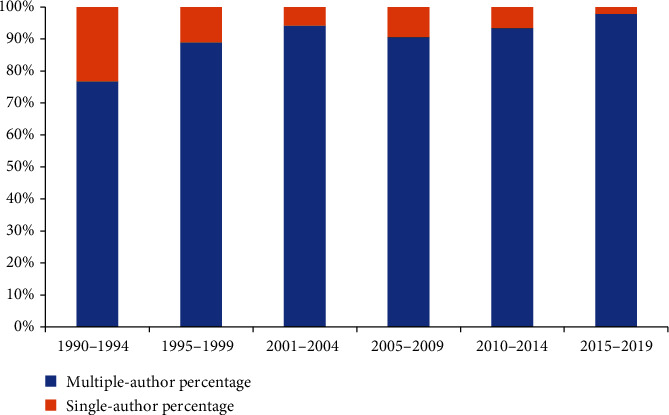
Trends in the percentage of single- vs. multiple-author articles per 5 years.

The 730 articles were contributed by 1000 authors. The top 10 authors and cocited authors were ranked based on the number of journals published (Table 2). Finnerup NB, who published 34 articles, ranked first, followed by Cardenas DD (29 publications) and Siddall PJ (27 publications). Siddall PJ was cocited 403 times, followed by Finnerup NB (269 times) and Widerstrom-noga EG (164 times).  Figure 9 indicates the cooperation between authors. Amongst the authors, Cardenas DD not only has many cooperative relations with Jensen MP but also has close cooperation with Turner JA. There is also close cooperation between Finnerup NB, which has the highest number of publications, and Siddall PJ, who ranks third in the number of publications. The proportion of single authors and multiple authors (authors ≥ 2) every 5 years is shown in Figure 10. The linear regression results showed that the percentage of papers with a single author significantly decreased (*t* = −3.557, *P* < 0.05) over time.

### 3.7. Analysis of References

**Figure 11:**
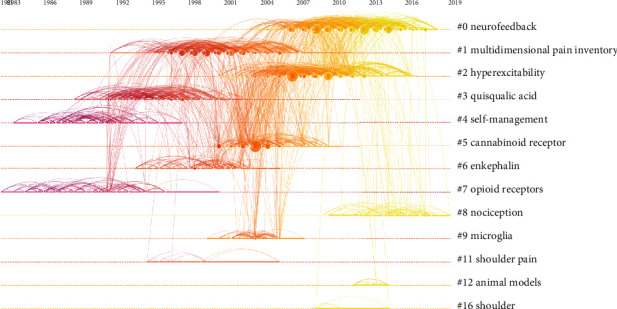
The analysis of references. Cocitation map (timeline view) of references from publications on pain after spinal cord injury.

The cocitation analysis of references is shown in the timeline view in Figure 11. CiteSpace automatically generated the top 17 clusters. The modular *Q* value shows the significance of the community structure. The modularity *Q* score was 0.8557 (higher than 0.5), which indicated that the network was reasonably distributed to loosely coupled clusters. The largest cluster was labeled “neurofeedback,” the second-largest clusters were “multidimensional” (#1) and “hyperexcitability” (#2), and the third-largest cluster (#3) was “quisqualic acid.”

### 3.8. Analysis of Keywords

**Figure 12:**
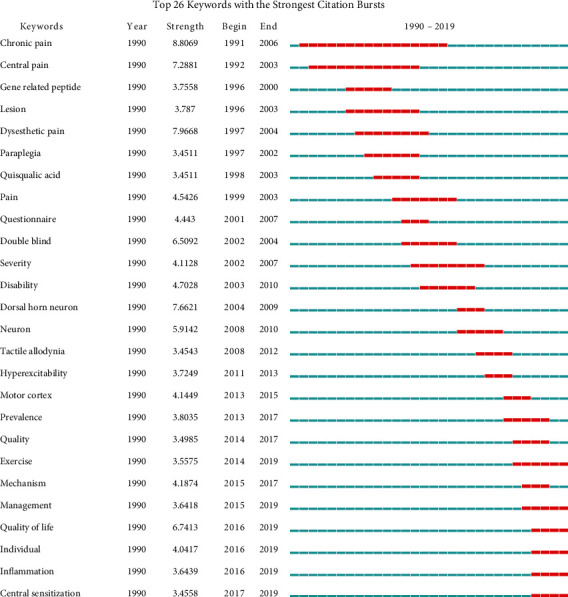
The keywords with the strongest citation bursts of publications on pain after spinal cord injury.

The top keyword with the strongest citation burst since 1991 was chronic pain, followed by central pain since 1992 (Figure 12). These keywords with high citation bursts reflect the topic frontier. The current keywords with the strongest citation bursts included “management” (2015–2019), “quality of life” (2016–2019), “individual” (2016–2019), “inflammation” (2016–2019), and “central sensitization” (2017–2019) amongst the top 26 keywords (chronic pain, central pain, gene-related peptide, lesion, dysesthetic pain, quisqualic pain, pain, questionnaire, double blind, severity, disability, dorsal horn neuron, neuron, tactile allodynia, receptor, hyperexcitability, motor cortex, efficacy, quality, exercise, mechanism, management, quality of life, individual, inflammation, and central sensitization).

### 3.9. Characteristics of the Top 10 Papers Cited Most Frequently

Table 3 shows the top 10 papers on pain after SCI with the largest number of citations. The 10 papers had 3166 citations, which is 12.07% of all the citations of the included articles. The article of Siddall PJ [26] with the title, “A longitudinal study of the prevalence and characteristics of pain in the first 5 years following spinal cord injury,” which was published in 2003 in *Journal of Pain*, was the most cited article (505 citations). The top 10 papers included one [27] journal with IF > 8, five [26, 28–31] journals with 5 ≤ IF < 8, three [32–34] journals with 3 ≤ IF < 5, and one [35] journal with 1 ≤ IF < 3.

## 4. Discussion

### 4.1. Global Trends of the Research on the Comorbidity of Pain and SCI

This paper presents a systematic overview by using bibliometric analysis to measure the studies on pain after SCI in the last three decades. The results showed that the global trend of the published works of literature on neuropathic pain after SCI had continued growth over time, indicating that pain after SCI attracted wide attention from researchers and provided a rich foundation for the follow-up research. Although the related publications showed a statistical growth year by year, the fastest growth rate of articles and open-access publications appeared from 2015 to 2019. The fastest growth rate of the number of citations appeared from 2005 to 2009, and the related papers published in 2010–2014 had the highest *H*-index value, indicating that the quality of papers published in the 2005–2014 period was improved.

The top 20 journals contributed to 58.43% (430) of the total number of publications on pain after SCI. *Spinal Cord* had a dominative contribution in terms of the number of works of literature on neuropathic pain and SCI research (11.96%), followed by *Pain* (8.15%), *Archives of Physical Medicine and Rehabilitation* (5.57%), and *Journal of Neurotrauma* (4.484%). The high number of citation frequency and citations per paper implied that *Spinal Cord* and *Pain* had superior quality and academic impression and were known as an unarguable mainstream subject on pain after SCI research. According to Journal Citation Reports (2019 edition), none of the top 20 journals had an IF greater than 10. Seven journals had an IF of 2–3 (*Clinical Journal of Pain*, *Neuroscience Letters*, *Disability and Rehabilitation*, *Journal of Rehabilitation Medicine*, *Journal of Pain Research*, *Spine*, and *Molecular Pain*), four journals had an IF of 3–5 (*Archives of Physical Medicine and Rehabilitation*, *Experimental Neurology*, *Journal of Neurotrauma*, and *European Journal of Pain*), and three papers had an IF of 5–10 (*Neurology*, *Journal of Neuroscience*, and *Neurology*). Amongst the top 20 journals, 35% were in Q1 and 45% were in Q2 according to the journal IF quartile in WoS. There are only three journals with IF > 5, and the average IF of the remaining was 3.265. There was still a challenge in writing in a high IF factor journal.

Based on the quantity of related publications on pain after SCI, the USA had a dominative contribution to the number of works of literature (356), followed by Australia (53), China (52), and Canada (41). The top 10 countries included two American countries, three Asia-Pacific countries, and four European countries.  Figure 7(b) shows the expansive network map of the cooperation of the countries by CiteSpace V with 97 nodes and 164 links. The link between the two nodes represents the frequency of cociting articles published by the two nodes, which implies the closeness of the connection between the two nodes. And we can easily acknowledge from Figure 7(b) that the University of Sydney had relatively close collaborations with others. A total of 805 institutions published papers on pain after SCI. Australia had three institutions, and the USA had six institutions (University of Miami, University of Texas System, University of Sydney, Veterans Health Administration, US Department of Veterans Affairs, and University of Alabama System). These results indicated that the USA was the main power in this field. The top 10 institutions were mainly from the USA with the most publications. The USA, as a developed country, is at the forefront of this research.

### 4.2. Research Focuses on the Comorbidity of Pain and Spine Core Injury Research

As shown in Figure 4, clinical neurology was the most prolific research field on pain after SCI according to the subject categories of WoS (355), followed by neurosciences (279), rehabilitation (210), and anesthesiology (111). The top 10 subject categories were rehabilitation, clinical neurology, neurosciences, anesthesiology, sport sciences, critical care medicine, pharmacology, orthopedics, surgery, and experimental medicine research. According to the synthetic analysis of the number of publications and citations, the number of citations per paper, and the*H*-index, we could acknowledge that the proportions of the top three subject categories of WoS (clinical neurology, neurosciences, and rehabilitation) were all above 20% and the number of citations per paper was all above 30, implying that the top three subject categories had superior quality and were recognized as the mainstream subject on the pain after SCI research. Based on the types of pain, the majority of the included articles involved neuropathic pain and treating pain after SCI. Amongst the top 10 types of pain (neuropathic pain, treating pain, nociceptive pain, animal models of pain, spontaneous pain, persistent pain, shoulder pain, musculoskeletal pain, low back pain, and upper extremity pain), neuropathic pain had the largest number of publications (506), citations (16,045), and open-access papers (266) and the highest *H*-index value (77), indicating that neuropathic pain has attracted wide attention from researchers, and it is also an urgent problem for patients after spinal cord injury.

In the cocitation map of references, “neurofeedback” was labeled as the largest cluster (#0), the second-largest clusters were “multidimensional pain inventory” (#1) and “hyperexcitability” (#2), and the third was “quisqualic acid” (#3). Based on the analysis of keywords, “chronic pain” had the strongest citation bursts since 1991. The top 26 keywords by the end of 2019 included “exercise” (2014–2019), “management” (2015–2019), “quality of life” (2016–2019), “individual” (2016–2019), “inflammation” (2016–2019), and “central sensitization” (2017–2019). Because of the multiple causes of neuropathic pain, this research area is very broad. However, these publications were mainly focused on neurofeedback and pain management. Patients after spinal cord injury inevitably suffer from different degrees and types of pain, so pain management is becoming more and more important. In addition, emerging interventions to manage pain are diverse, including exercise therapy that is not limited by venue and time. At present, many clinical studies [36–39] have shown that patients with spinal cord injury have good compliance with exercise therapy to relieve pain. Sumizono et al. [36] and Ditor et al. [38] have confirmed aerobic exercise can significantly alleviate neuropathic pain in patients with spinal cord injury. And the finding of Sumizono et al. [36] indicated that aerobic exercise alleviated neuropathic pain through the regulation of glial cell activation and expression of BDNF in the ipsilateral spinal dorsal horn and the endogenous opioid system. In addition, the result of Detloff et al.'s [40] study also suggested that there is a critical therapeutic window when exercise therapy may be effective at treating SCI-induced allodynia and that there are postinjury periods when exercise can be deleterious.

### 4.3. Strengths and Limitations

This study was the first to combine and integrate acquired information for the bibliometric analyses of the focus issues, direction, and development trend of research studies about pain after SCI over the last 30 years. These publications were retrieved from the SCI-Expanded WoS. The publication selects a variety of journals to ensure the integrity and diversity of the data. Our study included 730 articles on pain after SCI that were published in academic journals, such as *Pain*, *Neurology*, and *Journal of Neuroscience*. Subject categories, the number of publications and citations, the *H*-index in WoS, the collaborative analyses of journals and countries or institutions, and the cocitation analyses of references or authors and keywords were included.

This study has several limitations. This study only selected the SCI-Expanded WoS database for the retrieval of articles, and non-English papers were not included. Therefore, these factors may cause publication bias. Some influential papers may not be highly cited, whereas others are frequently cited, and the results are widely known.

## 5. Conclusions

This study provides the latest research direction for pain after SCI. This analysis can enable research teams to collaborate and promote the clinical management of pain after SCI. The initial three publications substantially increased to 47 publications from 1990 to 2019. The USA contributed the greatest number of published articles, and *Neurology* was the most influential journal on pain after SCI. Although this study has some limitations, it showed the common types of pain after SCI, especially neuropathic pain. According to the type of pain, 512 papers focused on neuropathic pain; thus, neuropathic pain is the most common type of pain after SCI. This historical overview of research into pain after SCI will be a useful basis for further research into development trends, focus issues, cooperators, and cooperative institutions.

## Figures and Tables

**Table 1:** The top 20 journals of origin of papers in pain after spinal cord injury.

Journals	Papers	Citations (WoS)	Citations per paper	Open-access papers	WoS categories	IF 2019	Quartile	*H*-index
*Spinal Cord*	88	3164	35.95	84	Clinical neurology; rehabilitation	1.773	Q3; Q2	34
*Pain*	60	4845	80.75	8	Anesthesiology; clinical neurology; neurosciences	5.483	Q1; Q1; Q1	37
*Archives of Physical Medicine and Rehabilitation*	41	2109	51.44	5	Rehabilitation; sport sciences	3.098	Q1; Q1	25
*Journal of Neurotrauma*	33	1127	34.15	12	Clinical neurology; critical care medicine; neurosciences	3.793	Q2; Q2; Q2	19
*Journal of Spinal Cord Medicine*	28	483	17.25	24	Clinical neurology	1.816	Q3	10
*Experimental Neurology*	22	1312	59.64	10	Neurosciences	4.691	Q1	17
*Clinical Journal of Pain*	18	684	38.00	2	Anesthesiology; clinical neurology	2.893	Q2; Q2	14
*Journal of Pain*	18	684	38.00	7	Clinical neurology; neurosciences	4.621	Q1; Q1	14
*Journal of Rehabilitation Research and Development*	15	597	39.80	5	Rehabilitation (SSCI); rehabilitation (SCIE)	1.277	Q2; Q3	15
*Journal of Neuroscience*	14	1629	116.36	12	Neurosciences	5.673	Q1	14
*Neuroscience Letters*	14	255	18.21	2	Neurosciences	2.274	Q3	10
*Disability and Rehabilitation*	12	184	15.33	2	Rehabilitation (SSCI); rehabilitation (SCIE)	2.222	Q1; Q1	9
*European Journal of Pain*	11	225	20.45	4	Anesthesiology; clinical neurology; neurosciences	3.492	Q2; Q2; Q2	6
*Journal of Rehabilitation Medicine*	11	224	20.36	11	Rehabilitation (SCIE); sport sciences	2.046	Q2; Q2	5
*Journal of Pain Research*	9	59	6.56	9	Clinical neurology	2.386	Q3	4
*Spine*	9	440	48.89	1	Clinical neurology; orthopedics	2.646	Q2; Q2	6
*American Journal of Physical Medicine Rehabilitation*	7	200	28.57	0	Rehabilitation (SCIE); sport sciences	1.838	Q2; Q3	6
*Neurology*	7	439	62.71	3	Clinical neurology	8.77	Q1	4
*PM R*	7	100	14.29	2	Rehabilitation (SCIE); sport sciences	1.821	Q2; Q3	4
*Molecular Pain*	6	152	25.33	6	Neurosciences	2.696	Q3	6

**Table 2:** The top 10 authors, cocited authors, and cocited references in pain after spinal cord injury.

Author	Published articles	Cocited author	Cited times	Cocited reference	Cited times
Finnerup NB	34	Siddall PJ	403	Siddall PJ, 2003, PAIN, V103, P249, DOI 10.1016/S0304-3959(02)00452-9	67
Cardenas DD	29	Finnerup NB	269	Bryce TN, 2012, SPINAL CORD, V50, P413, DOI 10.1038/sc.2011.156	52
Siddall PJ	27	Widerstrom-noga EG	164	Hains BC, 2006, J NEUROSCI, V26, P4308, DOI 10.1523/JNEUROSCI.0003-06.2006	44
Jensen MP	26	Jensen MP	140	Widerstrom-noga E, 2008, SPINAL CORD, V46, P818, DOI 10.1038/sc.2008.64	44
Richards JS	26	Yezierski RP	132	Hulsebosch CE, 2009, BRAIN RES REV, V60, P202, DOI 10.1016/j.brainresrev.2008.12.010	43
Hulsebosch CE	22	Rintala DH	123	Turner JA, 2001, ARCH PHYS MED REHAB, V82, P501, DOI 10.1053/apmr.2001.21855	42
Widerstrom-noga E	16	Hains BC	117	Siddall PJ, 1999, PAIN, V81, P187, DOI 10.1016/S0304-3959(99)00023-8	42
Widerstrom-noga EG	16	Cardenas DD	107	Siddall PJ, 2009, SPINAL CORD, V47, P352, DOI 10.1038/sc.2008.136	39
Yezierski RP	15	Hulsebosch CE	102	Finnerup NB, 2014, J PAIN, V15, P40, DOI 10.1016/j.jpain.2013.09.008	38
Jensen TS	13	Melzack R	99	Finnerup NB, 2012, CURR PAIN HEADACHE R, V16, P207, DOI 10.1007/s11916-012-0259-x	37

**Table 3:** The top 10 papers with the most citation frequency in pain after spinal cord injury.

Title	First author	Journal	Impact factor	Year	Citations (WoS)	WoS categories	Category ranking
A longitudinal study of the prevalence and characteristics of pain in the first 5 years following spinal cord injury	Siddall PJ	*Pain*	5.483	2003	505	Anesthesiology; clinical neurology; neurosciences	6/32; 25/204; 43/271
A sham-controlled, phase II trial of transcranial direct current stimulation for the treatment of central pain in traumatic spinal cord injury	Fregni F	*Pain*	5.483	2006	431	Anesthesiology; clinical neurology; neurosciences	6/32; 25/204; 43/271
Activated microglia contribute to the maintenance of chronic pain after spinal cord injury	Hains BC	*Journal of Neuroscience*	5.673	2006	425	Neurosciences	39/271
Incidence, prevalence, costs, and impact on disability of common conditions requiring rehabilitation in the United States: stroke, spinal cord injury, traumatic brain injury, multiple sclerosis, osteoarthritis, rheumatoid arthritis, limb loss, and back pain	Ma VY	*Archives of Physical Medicine and Rehabilitation*	3.098	2014	353	Rehabilitation in SCIE edition; sport sciences	9/68; 17/85
Evidence for spinal cord hypersensitivity in chronic pain after whiplash injury and in fibromyalgia	Banic B	*Pain*	5.483	2004	305	Anesthesiology; clinical neurology; neurosciences	6/32; 25/204; 43/271
Pregabalin in central neuropathic pain associated with spinal cord injury: a placebo-controlled trial	Siddall PJ	*Neurology*	8.77	2006	261	Clinical neurology	10/204
Upregulation of sodium channel Nav1.3 and functional involvement in neuronal hyperexcitability associated with central neuropathic pain after spinal cord injury	Hains BC	*Journal of Neuroscience*	5.673	2003	253	Neurosciences	39/271
A critical role of toll-like receptor 2 in nerve injury-induced spinal cord glial cell activation and pain hypersensitivity	Kim D	*Journal of Biological Chemistry*	4.238	2007	215	Biochemistry & molecular biology	87/297
Pain following spinal cord injury	Siddall PJ	*Spinal Cord*	1.773	2001	209	Clinical neurology; rehabilitation (SCIE)	150/204; 28/68
Chronic central pain after spinal cord injury	Christensen MD	*Journal of Neurotrauma*	3.793	1997	209	Clinical neurology; critical care medicine; neurosciences	52/204; 10/36; 95/271

## Data Availability

All research data used to support the findings of this study are included within the article and the supplementary information file.

## Supplementary Materials

Supplementary 1 Supplementary Figure 1: overview of the paper selection process. Supplementary Figure 2: the number of papers, citations, citations per paper, open-access papers, and *H*-index of the top 10 institutions.

Supplementary 2 Supplement Supplementary Table 1: raw data on journal sources of pain after spinal cord injury. Supplementary Table 2: raw data on countries/territories involved in pain after spinal cord injury. Supplementary Table 3: raw data on institutions involved in pain after spinal cord injury.
