# Supplementary material for: Bibliometric Study of Pain after Spinal Cord Injury
Source: Neural Plast. 2021 Feb 19;2021:6634644. doi: 10.1155/2021/6634644 (PMC7914384; doi:10.1155/2021/6634644)
Supplement: Supplementary 1 — Supplementary Figure 1: overview of the paper selection process. Supplementary Figure 2: the number of papers, citations, citations per paper, open-access papers, and H-index of the top 10 institutions. [file 6634644.f1.zip › Supplementary figure1.docx]

872 Papers Identified from Web of Science Core Collection

736 Papers Identified

730 Papers Identified

Excluded 136 Papers : Meeting Abstract, Proceedings Paper, Note, Correction, Book Review, News Item；Retraction

Excluded Papers 6 Non-English Papers

Supplementary Figure 1. Overview of paper selection process.
