# Supplementary material for: Bibliometric Study of Pain after Spinal Cord Injury
Source: Neural Plast. 2021 Feb 19;2021:6634644. doi: 10.1155/2021/6634644 (PMC7914384; doi:10.1155/2021/6634644)
Supplement: Supplementary 2 — Supplement Supplementary Table 1: raw data on journal sources of pain after spinal cord injury. Supplementary Table 2: raw data on countries/territories involved in pain after spinal cord injury. Supplementary Table 3: raw data on institutions involved in pain after spinal cord injury. [file 6634644.f2.zip › Supplementary Table 3.docx]

Supplementary Table 3. Raw data on journal involved in pain and spine core injury publications.

| **Journal** | **records** | **% of 3423** |
| --- | --- | --- |
| SPINAL CORD | 88 | 12.055 |
| PAIN | 60 | 8.219 |
| ARCHIVES OF PHYSICAL MEDICINE AND REHABILITATION | 41 | 5.616 |
| JOURNAL OF NEUROTRAUMA | 33 | 4.521 |
| JOURNAL OF SPINAL CORD MEDICINE | 28 | 3.836 |
| EXPERIMENTAL NEUROLOGY | 22 | 3.014 |
| CLINICAL JOURNAL OF PAIN | 18 | 2.466 |
| JOURNAL OF PAIN | 18 | 2.466 |
| JOURNAL OF REHABILITATION RESEARCH AND DEVELOPMENT | 15 | 2.055 |
| JOURNAL OF NEUROSCIENCE | 14 | 1.918 |
| NEUROSCIENCE LETTERS | 14 | 1.918 |
| DISABILITY AND REHABILITATION | 12 | 1.644 |
| EUROPEAN JOURNAL OF PAIN | 11 | 1.507 |
| JOURNAL OF REHABILITATION MEDICINE | 11 | 1.507 |
| JOURNAL OF PAIN RESEARCH | 9 | 1.233 |
| SPINE | 9 | 1.233 |
| AMERICAN JOURNAL OF PHYSICAL MEDICINE REHABILITATION | 7 | 0.959 |
| NEUROLOGY | 7 | 0.959 |
| PM R | 7 | 0.959 |
| MOLECULAR PAIN | 6 | 0.822 |
| NEUROREHABILITATION AND NEURAL REPAIR | 6 | 0.822 |
| NEUROSCIENCE | 6 | 0.822 |
| PAIN PHYSICIAN | 6 | 0.822 |
| ACTA NEUROCHIRURGICA | 5 | 0.685 |
| ANESTHESIA AND ANALGESIA | 5 | 0.685 |
| PARAPLEGIA | 5 | 0.685 |
| PLOS ONE | 5 | 0.685 |
| BRAIN | 4 | 0.548 |
| BRAIN RESEARCH | 4 | 0.548 |
| BRAIN RESEARCH BULLETIN | 4 | 0.548 |
| GLIA | 4 | 0.548 |
| JOURNAL OF NEUROSURGERY | 4 | 0.548 |
| JOURNAL OF NEUROSURGERY SPINE | 4 | 0.548 |
| SCIENTIFIC REPORTS | 4 | 0.548 |
| CLINICAL NEUROPHYSIOLOGY | 3 | 0.411 |
| CLINICAL REHABILITATION | 3 | 0.411 |
| EUROPEAN JOURNAL OF PHARMACOLOGY | 3 | 0.411 |
| FRONTIERS IN PHYSIOLOGY | 3 | 0.411 |
| JOURNAL OF BACK AND MUSCULOSKELETAL REHABILITATION | 3 | 0.411 |
| JOURNAL OF NEUROCHEMISTRY | 3 | 0.411 |
| JOURNAL OF NEUROINFLAMMATION | 3 | 0.411 |
| JOURNAL OF PHARMACOLOGY AND EXPERIMENTAL THERAPEUTICS | 3 | 0.411 |
| MEDICINE | 3 | 0.411 |
| NEURAL REGENERATION RESEARCH | 3 | 0.411 |
| PAIN MEDICINE | 3 | 0.411 |
| PHYSICAL THERAPY | 3 | 0.411 |
| PROCEEDINGS OF THE NATIONAL ACADEMY OF SCIENCES OF THE UNITED STATES OF AMERICA | 3 | 0.411 |
| SOMATOSENSORY AND MOTOR RESEARCH | 3 | 0.411 |
| SPINE JOURNAL | 3 | 0.411 |
| WORLD NEUROSURGERY | 3 | 0.411 |
| ACTA MEDICA MEDITERRANEA | 2 | 0.274 |
| ANNALS OF NEUROLOGY | 2 | 0.274 |
| ANNALS OF PHYSICAL AND REHABILITATION MEDICINE | 2 | 0.274 |
| ARQUIVOS DE NEURO PSIQUIATRIA | 2 | 0.274 |
| BMC NEUROLOGY | 2 | 0.274 |
| BRAIN RESEARCH REVIEWS | 2 | 0.274 |
| BRITISH JOURNAL OF PHARMACOLOGY | 2 | 0.274 |
| CELL DEATH DISEASE | 2 | 0.274 |
| CLINICAL ORTHOPAEDICS AND RELATED RESEARCH | 2 | 0.274 |
| COMPLEMENTARY THERAPIES IN MEDICINE | 2 | 0.274 |
| CURRENT PAIN AND HEADACHE REPORTS | 2 | 0.274 |
| EUROPEAN JOURNAL OF PHYSICAL AND REHABILITATION MEDICINE | 2 | 0.274 |
| EXPERIMENTAL AND THERAPEUTIC MEDICINE | 2 | 0.274 |
| FRONTIERS IN SYSTEMS NEUROSCIENCE | 2 | 0.274 |
| INTERNATIONAL JOURNAL OF REHABILITATION RESEARCH | 2 | 0.274 |
| JOURNAL OF NEUROLOGY NEUROSURGERY AND PSYCHIATRY | 2 | 0.274 |
| JOURNAL OF NEUROSCIENCE RESEARCH | 2 | 0.274 |
| JOURNAL OF ORTHOPAEDIC SURGERY AND RESEARCH | 2 | 0.274 |
| JOURNAL OF PAIN AND SYMPTOM MANAGEMENT | 2 | 0.274 |
| METABOLIC BRAIN DISEASE | 2 | 0.274 |
| MOLECULAR BRAIN RESEARCH | 2 | 0.274 |
| MOLECULAR THERAPY | 2 | 0.274 |
| NEUROBIOLOGY OF DISEASE | 2 | 0.274 |
| NEUROIMAGE | 2 | 0.274 |
| NEUROMODULATION | 2 | 0.274 |
| NEUROPHARMACOLOGY | 2 | 0.274 |
| NEUROSCIENCE BULLETIN | 2 | 0.274 |
| NEUROSCIENCE RESEARCH | 2 | 0.274 |
| NEUROSCIENCES | 2 | 0.274 |
| PAIN CLINIC | 2 | 0.274 |
| PAIN PRACTICE | 2 | 0.274 |
| PHARMACOLOGY BIOCHEMISTRY AND BEHAVIOR | 2 | 0.274 |
| QUALITY OF LIFE RESEARCH | 2 | 0.274 |
| REGIONAL ANESTHESIA AND PAIN MEDICINE | 2 | 0.274 |
| RESTORATIVE NEUROLOGY AND NEUROSCIENCE | 2 | 0.274 |
| STEM CELL RESEARCH THERAPY | 2 | 0.274 |
| STEM CELLS | 2 | 0.274 |
| ACTA ANAESTHESIOLOGICA SCANDINAVICA | 1 | 0.137 |
| ACTA BIOCHIMICA ET BIOPHYSICA SINICA | 1 | 0.137 |
| ACTA PHARMACOLOGICA SINICA | 1 | 0.137 |
| ACUPUNCTURE IN MEDICINE | 1 | 0.137 |
| ADVANCES IN EXPERIMENTAL MEDICINE AND BIOLOGY | 1 | 0.137 |
| ADVANCES IN PAIN RESEARCH AND THERAPY | 1 | 0.137 |
| AMERICAN JOURNAL OF NEURORADIOLOGY | 1 | 0.137 |
| AMERICAN JOURNAL OF SURGERY | 1 | 0.137 |
| ANAESTHESIA AND INTENSIVE CARE | 1 | 0.137 |
| ANESTHESIOLOGY | 1 | 0.137 |
| ANKARA UNIVERSITESI VETERINER FAKULTESI DERGISI | 1 | 0.137 |
| ANNALS OF SAUDI MEDICINE | 1 | 0.137 |
| ANNALS OF THE NEW YORK ACADEMY OF SCIENCES | 1 | 0.137 |
| AUSTRALIAN OCCUPATIONAL THERAPY JOURNAL | 1 | 0.137 |
| AUTONOMIC NEUROSCIENCE BASIC CLINICAL | 1 | 0.137 |
| BEHAVIOURAL BRAIN RESEARCH | 1 | 0.137 |
| BEHAVIOURAL PHARMACOLOGY | 1 | 0.137 |
| BIOCHIMICA ET BIOPHYSICA ACTA MOLECULAR BASIS OF DISEASE | 1 | 0.137 |
| BIOLOGICAL RESEARCH FOR NURSING | 1 | 0.137 |
| BIOMED RESEARCH INTERNATIONAL | 1 | 0.137 |
| BIOMEDICAL RESEARCH INDIA | 1 | 0.137 |
| BMJ OPEN | 1 | 0.137 |
| BRAIN BEHAVIOR AND IMMUNITY | 1 | 0.137 |
| CANADIAN JOURNAL OF PHYSIOLOGY AND PHARMACOLOGY | 1 | 0.137 |
| CANADIAN MEDICAL ASSOCIATION JOURNAL | 1 | 0.137 |
| CARDIOLOGY IN THE YOUNG | 1 | 0.137 |
| CELL STEM CELL | 1 | 0.137 |
| CELL TRANSPLANTATION | 1 | 0.137 |
| CEREBRAL CORTEX | 1 | 0.137 |
| CHINESE MEDICAL JOURNAL | 1 | 0.137 |
| CLINICAL NEUROLOGY AND NEUROSURGERY | 1 | 0.137 |
| CLINICAL PHARMACOLOGY IN DRUG DEVELOPMENT | 1 | 0.137 |
| CLINICAL THERAPEUTICS | 1 | 0.137 |
| CNS DRUGS | 1 | 0.137 |
| COCHRANE DATABASE OF SYSTEMATIC REVIEWS | 1 | 0.137 |
| COMPUTER METHODS AND PROGRAMS IN BIOMEDICINE | 1 | 0.137 |
| CURRENT OPINION IN NEUROLOGY | 1 | 0.137 |
| CURRENT PHARMACEUTICAL DESIGN | 1 | 0.137 |
| CURRENT PROTEIN PEPTIDE SCIENCE | 1 | 0.137 |
| DRUG DEVELOPMENT RESEARCH | 1 | 0.137 |
| DRUGS | 1 | 0.137 |
| ELECTROMAGNETIC BIOLOGY AND MEDICINE | 1 | 0.137 |
| ENDOCRINOLOGY | 1 | 0.137 |
| ERGONOMICS | 1 | 0.137 |
| EUROPEAN JOURNAL OF CLINICAL PHARMACOLOGY | 1 | 0.137 |
| EUROPEAN JOURNAL OF NEUROLOGY | 1 | 0.137 |
| EUROPEAN JOURNAL OF NEUROSCIENCE | 1 | 0.137 |
| EUROPEAN REVIEW FOR MEDICAL AND PHARMACOLOGICAL SCIENCES | 1 | 0.137 |
| EXPERIMENTAL BRAIN RESEARCH | 1 | 0.137 |
| EXPERT OPINION ON BIOLOGICAL THERAPY | 1 | 0.137 |
| EXPLORE THE JOURNAL OF SCIENCE AND HEALING | 1 | 0.137 |
| FRONTIERS IN CELLULAR NEUROSCIENCE | 1 | 0.137 |
| FRONTIERS IN HUMAN NEUROSCIENCE | 1 | 0.137 |
| FRONTIERS IN NEUROLOGY | 1 | 0.137 |
| FRONTIERS IN NEUROSCIENCE | 1 | 0.137 |
| FRONTIERS IN PHARMACOLOGY | 1 | 0.137 |
| GENE THERAPY | 1 | 0.137 |
| INTERNATIONAL JOURNAL OF INDUSTRIAL ERGONOMICS | 1 | 0.137 |
| INTERNATIONAL JOURNAL OF PHARMACOLOGY | 1 | 0.137 |
| JCPSP JOURNAL OF THE COLLEGE OF PHYSICIANS AND SURGEONS PAKISTAN | 1 | 0.137 |
| JOURNAL OF BIOLOGICAL CHEMISTRY | 1 | 0.137 |
| JOURNAL OF BIOMEDICINE AND BIOTECHNOLOGY | 1 | 0.137 |
| JOURNAL OF CHEMICAL NEUROANATOMY | 1 | 0.137 |
| JOURNAL OF CLINICAL ANESTHESIA | 1 | 0.137 |
| JOURNAL OF CLINICAL NEUROSCIENCE | 1 | 0.137 |
| JOURNAL OF COMPARATIVE NEUROLOGY | 1 | 0.137 |
| JOURNAL OF CONTROLLED RELEASE | 1 | 0.137 |
| JOURNAL OF ENDOCRINOLOGICAL INVESTIGATION | 1 | 0.137 |
| JOURNAL OF KOREAN NEUROSURGICAL SOCIETY | 1 | 0.137 |
| JOURNAL OF MOLECULAR NEUROSCIENCE | 1 | 0.137 |
| JOURNAL OF MUSCULOSKELETAL PAIN | 1 | 0.137 |
| JOURNAL OF NEUROENGINEERING AND REHABILITATION | 1 | 0.137 |
| JOURNAL OF NEUROIMMUNE PHARMACOLOGY | 1 | 0.137 |
| JOURNAL OF NEUROIMMUNOLOGY | 1 | 0.137 |
| JOURNAL OF NEUROLOGIC PHYSICAL THERAPY | 1 | 0.137 |
| JOURNAL OF NEUROPHYSIOLOGY | 1 | 0.137 |
| JOURNAL OF NEUROSCIENCE NURSING | 1 | 0.137 |
| JOURNAL OF NEUROSURGICAL SCIENCES | 1 | 0.137 |
| JOURNAL OF NURSING RESEARCH | 1 | 0.137 |
| JOURNAL OF ORTHOPAEDIC RESEARCH | 1 | 0.137 |
| JOURNAL OF PHARMACOLOGICAL SCIENCES | 1 | 0.137 |
| JOURNAL OF PHYSICAL THERAPY SCIENCE | 1 | 0.137 |
| JOURNAL OF TRANSLATIONAL MEDICINE | 1 | 0.137 |
| KOREAN JOURNAL OF PHYSIOLOGY PHARMACOLOGY | 1 | 0.137 |
| MEDICAL HYPOTHESES | 1 | 0.137 |
| MEDICAL SCIENCE MONITOR | 1 | 0.137 |
| MOLECULAR MEDICINE | 1 | 0.137 |
| MOLECULAR MEDICINE REPORTS | 1 | 0.137 |
| MOLECULAR NEUROBIOLOGY | 1 | 0.137 |
| MOLECULES AND CELLS | 1 | 0.137 |
| NEURAL PLASTICITY | 1 | 0.137 |
| NEUROCHEMICAL RESEARCH | 1 | 0.137 |
| NEUROIMAGE CLINICAL | 1 | 0.137 |
| NEUROLOGICAL RESEARCH | 1 | 0.137 |
| NEURON | 1 | 0.137 |
| NEURONS AND NETWORKS IN THE SPINAL CORD | 1 | 0.137 |
| NEUROPEPTIDES | 1 | 0.137 |
| NEUROPHYSIOLOGY | 1 | 0.137 |
| NEUROREHABILITATION | 1 | 0.137 |
| NEUROSIGNALS | 1 | 0.137 |
| NEUROSURGERY | 1 | 0.137 |
| NEUROTHERAPEUTICS | 1 | 0.137 |
| ONCOLOGY | 1 | 0.137 |
| ORTHOPEDICS | 1 | 0.137 |
| PAKISTAN JOURNAL OF MEDICAL SCIENCES | 1 | 0.137 |
| PHYSICAL MEDICINE AND REHABILITATION CLINICS OF NORTH AMERICA | 1 | 0.137 |
| PHYSIOLOGICAL RESEARCH | 1 | 0.137 |
| REGENERATIVE BIOLOGY OF THE SPINE AND SPINAL CORD | 1 | 0.137 |
| SEMINARS IN IMMUNOLOGY | 1 | 0.137 |
| STEREOTACTIC AND FUNCTIONAL NEUROSURGERY | 1 | 0.137 |
| TOXINS | 1 | 0.137 |
| TRANSLATIONAL NEUROSCIENCE | 1 | 0.137 |
| TRENDS IN NEUROSCIENCES | 1 | 0.137 |
| TURKIYE FIZIKSEL TIP VE REHABILITASYON DERGISI TURKISH JOURNAL OF PHYSICAL MEDICINE AND REHABILITATION | 1 | 0.137 |
| SPINAL CORD | 88 | 12.055 |
| PAIN | 60 | 8.219 |
| ARCHIVES OF PHYSICAL MEDICINE AND REHABILITATION | 41 | 5.616 |
| JOURNAL OF NEUROTRAUMA | 33 | 4.521 |
| JOURNAL OF SPINAL CORD MEDICINE | 28 | 3.836 |
| EXPERIMENTAL NEUROLOGY | 22 | 3.014 |
| CLINICAL JOURNAL OF PAIN | 18 | 2.466 |
| JOURNAL OF PAIN | 18 | 2.466 |
| JOURNAL OF REHABILITATION RESEARCH AND DEVELOPMENT | 15 | 2.055 |
| JOURNAL OF NEUROSCIENCE | 14 | 1.918 |
| NEUROSCIENCE LETTERS | 14 | 1.918 |
| DISABILITY AND REHABILITATION | 12 | 1.644 |
| EUROPEAN JOURNAL OF PAIN | 11 | 1.507 |
| JOURNAL OF REHABILITATION MEDICINE | 11 | 1.507 |
| JOURNAL OF PAIN RESEARCH | 9 | 1.233 |
| SPINE | 9 | 1.233 |
| AMERICAN JOURNAL OF PHYSICAL MEDICINE REHABILITATION | 7 | 0.959 |
| NEUROLOGY | 7 | 0.959 |
| PM R | 7 | 0.959 |
| MOLECULAR PAIN | 6 | 0.822 |
| NEUROREHABILITATION AND NEURAL REPAIR | 6 | 0.822 |
| NEUROSCIENCE | 6 | 0.822 |
| PAIN PHYSICIAN | 6 | 0.822 |
| ACTA NEUROCHIRURGICA | 5 | 0.685 |
| ANESTHESIA AND ANALGESIA | 5 | 0.685 |
| PARAPLEGIA | 5 | 0.685 |
| PLOS ONE | 5 | 0.685 |
| BRAIN | 4 | 0.548 |
| BRAIN RESEARCH | 4 | 0.548 |
| BRAIN RESEARCH BULLETIN | 4 | 0.548 |
| GLIA | 4 | 0.548 |
| JOURNAL OF NEUROSURGERY | 4 | 0.548 |
| JOURNAL OF NEUROSURGERY SPINE | 4 | 0.548 |
| SCIENTIFIC REPORTS | 4 | 0.548 |
| CLINICAL NEUROPHYSIOLOGY | 3 | 0.411 |
| CLINICAL REHABILITATION | 3 | 0.411 |
| EUROPEAN JOURNAL OF PHARMACOLOGY | 3 | 0.411 |
| FRONTIERS IN PHYSIOLOGY | 3 | 0.411 |
| JOURNAL OF BACK AND MUSCULOSKELETAL REHABILITATION | 3 | 0.411 |
| JOURNAL OF NEUROCHEMISTRY | 3 | 0.411 |
| JOURNAL OF NEUROINFLAMMATION | 3 | 0.411 |
| JOURNAL OF PHARMACOLOGY AND EXPERIMENTAL THERAPEUTICS | 3 | 0.411 |
| MEDICINE | 3 | 0.411 |
| NEURAL REGENERATION RESEARCH | 3 | 0.411 |
| PAIN MEDICINE | 3 | 0.411 |
| PHYSICAL THERAPY | 3 | 0.411 |
| PROCEEDINGS OF THE NATIONAL ACADEMY OF SCIENCES OF THE UNITED STATES OF AMERICA | 3 | 0.411 |
| SOMATOSENSORY AND MOTOR RESEARCH | 3 | 0.411 |
| SPINE JOURNAL | 3 | 0.411 |
| WORLD NEUROSURGERY | 3 | 0.411 |
| ACTA MEDICA MEDITERRANEA | 2 | 0.274 |
| ANNALS OF NEUROLOGY | 2 | 0.274 |
| ANNALS OF PHYSICAL AND REHABILITATION MEDICINE | 2 | 0.274 |
| ARQUIVOS DE NEURO PSIQUIATRIA | 2 | 0.274 |
| BMC NEUROLOGY | 2 | 0.274 |
| BRAIN RESEARCH REVIEWS | 2 | 0.274 |
| BRITISH JOURNAL OF PHARMACOLOGY | 2 | 0.274 |
| CELL DEATH DISEASE | 2 | 0.274 |
| CLINICAL ORTHOPAEDICS AND RELATED RESEARCH | 2 | 0.274 |
| COMPLEMENTARY THERAPIES IN MEDICINE | 2 | 0.274 |
| CURRENT PAIN AND HEADACHE REPORTS | 2 | 0.274 |
| EUROPEAN JOURNAL OF PHYSICAL AND REHABILITATION MEDICINE | 2 | 0.274 |
| EXPERIMENTAL AND THERAPEUTIC MEDICINE | 2 | 0.274 |
| FRONTIERS IN SYSTEMS NEUROSCIENCE | 2 | 0.274 |
| INTERNATIONAL JOURNAL OF REHABILITATION RESEARCH | 2 | 0.274 |
| JOURNAL OF NEUROLOGY NEUROSURGERY AND PSYCHIATRY | 2 | 0.274 |
| JOURNAL OF NEUROSCIENCE RESEARCH | 2 | 0.274 |
| JOURNAL OF ORTHOPAEDIC SURGERY AND RESEARCH | 2 | 0.274 |
| JOURNAL OF PAIN AND SYMPTOM MANAGEMENT | 2 | 0.274 |
| METABOLIC BRAIN DISEASE | 2 | 0.274 |
| MOLECULAR BRAIN RESEARCH | 2 | 0.274 |
| MOLECULAR THERAPY | 2 | 0.274 |
| NEUROBIOLOGY OF DISEASE | 2 | 0.274 |
| NEUROIMAGE | 2 | 0.274 |
| NEUROMODULATION | 2 | 0.274 |
| NEUROPHARMACOLOGY | 2 | 0.274 |
| NEUROSCIENCE BULLETIN | 2 | 0.274 |
| NEUROSCIENCE RESEARCH | 2 | 0.274 |
| NEUROSCIENCES | 2 | 0.274 |
| PAIN CLINIC | 2 | 0.274 |
| PAIN PRACTICE | 2 | 0.274 |
| PHARMACOLOGY BIOCHEMISTRY AND BEHAVIOR | 2 | 0.274 |
| QUALITY OF LIFE RESEARCH | 2 | 0.274 |
| REGIONAL ANESTHESIA AND PAIN MEDICINE | 2 | 0.274 |
| RESTORATIVE NEUROLOGY AND NEUROSCIENCE | 2 | 0.274 |
| STEM CELL RESEARCH THERAPY | 2 | 0.274 |
| STEM CELLS | 2 | 0.274 |
| ACTA ANAESTHESIOLOGICA SCANDINAVICA | 1 | 0.137 |
| ACTA BIOCHIMICA ET BIOPHYSICA SINICA | 1 | 0.137 |
| ACTA PHARMACOLOGICA SINICA | 1 | 0.137 |
| ACUPUNCTURE IN MEDICINE | 1 | 0.137 |
| ADVANCES IN EXPERIMENTAL MEDICINE AND BIOLOGY | 1 | 0.137 |
| ADVANCES IN PAIN RESEARCH AND THERAPY | 1 | 0.137 |
| AMERICAN JOURNAL OF NEURORADIOLOGY | 1 | 0.137 |
| AMERICAN JOURNAL OF SURGERY | 1 | 0.137 |
| ANAESTHESIA AND INTENSIVE CARE | 1 | 0.137 |
| ANESTHESIOLOGY | 1 | 0.137 |
| ANKARA UNIVERSITESI VETERINER FAKULTESI DERGISI | 1 | 0.137 |
| ANNALS OF SAUDI MEDICINE | 1 | 0.137 |
| ANNALS OF THE NEW YORK ACADEMY OF SCIENCES | 1 | 0.137 |
| AUSTRALIAN OCCUPATIONAL THERAPY JOURNAL | 1 | 0.137 |
| AUTONOMIC NEUROSCIENCE BASIC CLINICAL | 1 | 0.137 |
| BEHAVIOURAL BRAIN RESEARCH | 1 | 0.137 |
| BEHAVIOURAL PHARMACOLOGY | 1 | 0.137 |
| BIOCHIMICA ET BIOPHYSICA ACTA MOLECULAR BASIS OF DISEASE | 1 | 0.137 |
| BIOLOGICAL RESEARCH FOR NURSING | 1 | 0.137 |
| BIOMED RESEARCH INTERNATIONAL | 1 | 0.137 |
| BIOMEDICAL RESEARCH INDIA | 1 | 0.137 |
| BMJ OPEN | 1 | 0.137 |
| BRAIN BEHAVIOR AND IMMUNITY | 1 | 0.137 |
| CANADIAN JOURNAL OF PHYSIOLOGY AND PHARMACOLOGY | 1 | 0.137 |
| CANADIAN MEDICAL ASSOCIATION JOURNAL | 1 | 0.137 |
| CARDIOLOGY IN THE YOUNG | 1 | 0.137 |
| CELL STEM CELL | 1 | 0.137 |
| CELL TRANSPLANTATION | 1 | 0.137 |
| CEREBRAL CORTEX | 1 | 0.137 |
| CHINESE MEDICAL JOURNAL | 1 | 0.137 |
| CLINICAL NEUROLOGY AND NEUROSURGERY | 1 | 0.137 |
| CLINICAL PHARMACOLOGY IN DRUG DEVELOPMENT | 1 | 0.137 |
| CLINICAL THERAPEUTICS | 1 | 0.137 |
| CNS DRUGS | 1 | 0.137 |
| COCHRANE DATABASE OF SYSTEMATIC REVIEWS | 1 | 0.137 |
| COMPUTER METHODS AND PROGRAMS IN BIOMEDICINE | 1 | 0.137 |
| CURRENT OPINION IN NEUROLOGY | 1 | 0.137 |
| CURRENT PHARMACEUTICAL DESIGN | 1 | 0.137 |
| CURRENT PROTEIN PEPTIDE SCIENCE | 1 | 0.137 |
| DRUG DEVELOPMENT RESEARCH | 1 | 0.137 |
| DRUGS | 1 | 0.137 |
| ELECTROMAGNETIC BIOLOGY AND MEDICINE | 1 | 0.137 |
| ENDOCRINOLOGY | 1 | 0.137 |
| ERGONOMICS | 1 | 0.137 |
| EUROPEAN JOURNAL OF CLINICAL PHARMACOLOGY | 1 | 0.137 |
| EUROPEAN JOURNAL OF NEUROLOGY | 1 | 0.137 |
| EUROPEAN JOURNAL OF NEUROSCIENCE | 1 | 0.137 |
| EUROPEAN REVIEW FOR MEDICAL AND PHARMACOLOGICAL SCIENCES | 1 | 0.137 |
| EXPERIMENTAL BRAIN RESEARCH | 1 | 0.137 |
| EXPERT OPINION ON BIOLOGICAL THERAPY | 1 | 0.137 |
| EXPLORE THE JOURNAL OF SCIENCE AND HEALING | 1 | 0.137 |
| FRONTIERS IN CELLULAR NEUROSCIENCE | 1 | 0.137 |
| FRONTIERS IN HUMAN NEUROSCIENCE | 1 | 0.137 |
| FRONTIERS IN NEUROLOGY | 1 | 0.137 |
| FRONTIERS IN NEUROSCIENCE | 1 | 0.137 |
| FRONTIERS IN PHARMACOLOGY | 1 | 0.137 |
| GENE THERAPY | 1 | 0.137 |
| INTERNATIONAL JOURNAL OF INDUSTRIAL ERGONOMICS | 1 | 0.137 |
| INTERNATIONAL JOURNAL OF PHARMACOLOGY | 1 | 0.137 |
| JCPSP JOURNAL OF THE COLLEGE OF PHYSICIANS AND SURGEONS PAKISTAN | 1 | 0.137 |
| JOURNAL OF BIOLOGICAL CHEMISTRY | 1 | 0.137 |
| JOURNAL OF BIOMEDICINE AND BIOTECHNOLOGY | 1 | 0.137 |
| JOURNAL OF CHEMICAL NEUROANATOMY | 1 | 0.137 |
| JOURNAL OF CLINICAL ANESTHESIA | 1 | 0.137 |
| JOURNAL OF CLINICAL NEUROSCIENCE | 1 | 0.137 |
| JOURNAL OF COMPARATIVE NEUROLOGY | 1 | 0.137 |
| JOURNAL OF CONTROLLED RELEASE | 1 | 0.137 |
| JOURNAL OF ENDOCRINOLOGICAL INVESTIGATION | 1 | 0.137 |
| JOURNAL OF KOREAN NEUROSURGICAL SOCIETY | 1 | 0.137 |
| JOURNAL OF MOLECULAR NEUROSCIENCE | 1 | 0.137 |
| JOURNAL OF MUSCULOSKELETAL PAIN | 1 | 0.137 |
| JOURNAL OF NEUROENGINEERING AND REHABILITATION | 1 | 0.137 |
| JOURNAL OF NEUROIMMUNE PHARMACOLOGY | 1 | 0.137 |
| JOURNAL OF NEUROIMMUNOLOGY | 1 | 0.137 |
| JOURNAL OF NEUROLOGIC PHYSICAL THERAPY | 1 | 0.137 |
| JOURNAL OF NEUROPHYSIOLOGY | 1 | 0.137 |
| JOURNAL OF NEUROSCIENCE NURSING | 1 | 0.137 |
| JOURNAL OF NEUROSURGICAL SCIENCES | 1 | 0.137 |
| JOURNAL OF NURSING RESEARCH | 1 | 0.137 |
| JOURNAL OF ORTHOPAEDIC RESEARCH | 1 | 0.137 |
| JOURNAL OF PHARMACOLOGICAL SCIENCES | 1 | 0.137 |
| JOURNAL OF PHYSICAL THERAPY SCIENCE | 1 | 0.137 |
| JOURNAL OF TRANSLATIONAL MEDICINE | 1 | 0.137 |
| KOREAN JOURNAL OF PHYSIOLOGY PHARMACOLOGY | 1 | 0.137 |
| MEDICAL HYPOTHESES | 1 | 0.137 |
| MEDICAL SCIENCE MONITOR | 1 | 0.137 |
| MOLECULAR MEDICINE | 1 | 0.137 |
| MOLECULAR MEDICINE REPORTS | 1 | 0.137 |
| MOLECULAR NEUROBIOLOGY | 1 | 0.137 |
| MOLECULES AND CELLS | 1 | 0.137 |
| NEURAL PLASTICITY | 1 | 0.137 |
| NEUROCHEMICAL RESEARCH | 1 | 0.137 |
| NEUROIMAGE CLINICAL | 1 | 0.137 |
| NEUROLOGICAL RESEARCH | 1 | 0.137 |
| NEURON | 1 | 0.137 |
| NEURONS AND NETWORKS IN THE SPINAL CORD | 1 | 0.137 |
| NEUROPEPTIDES | 1 | 0.137 |
| NEUROPHYSIOLOGY | 1 | 0.137 |
| NEUROREHABILITATION | 1 | 0.137 |
| NEUROSIGNALS | 1 | 0.137 |
| NEUROSURGERY | 1 | 0.137 |
| NEUROTHERAPEUTICS | 1 | 0.137 |
| ONCOLOGY | 1 | 0.137 |
| ORTHOPEDICS | 1 | 0.137 |
| PAKISTAN JOURNAL OF MEDICAL SCIENCES | 1 | 0.137 |
| PHYSICAL MEDICINE AND REHABILITATION CLINICS OF NORTH AMERICA | 1 | 0.137 |
| PHYSIOLOGICAL RESEARCH | 1 | 0.137 |
| REGENERATIVE BIOLOGY OF THE SPINE AND SPINAL CORD | 1 | 0.137 |
| SEMINARS IN IMMUNOLOGY | 1 | 0.137 |
| STEREOTACTIC AND FUNCTIONAL NEUROSURGERY | 1 | 0.137 |
| TOXINS | 1 | 0.137 |
| TRANSLATIONAL NEUROSCIENCE | 1 | 0.137 |
| TRENDS IN NEUROSCIENCES | 1 | 0.137 |
| TURKIYE FIZIKSEL TIP VE REHABILITASYON DERGISI TURKISH JOURNAL OF PHYSICAL MEDICINE AND REHABILITATION | 1 | 0.137 |
| SPINAL CORD | 88 | 12.055 |
| PAIN | 60 | 8.219 |
| ARCHIVES OF PHYSICAL MEDICINE AND REHABILITATION | 41 | 5.616 |
| JOURNAL OF NEUROTRAUMA | 33 | 4.521 |
| JOURNAL OF SPINAL CORD MEDICINE | 28 | 3.836 |
| EXPERIMENTAL NEUROLOGY | 22 | 3.014 |
| CLINICAL JOURNAL OF PAIN | 18 | 2.466 |
| JOURNAL OF PAIN | 18 | 2.466 |
| JOURNAL OF REHABILITATION RESEARCH AND DEVELOPMENT | 15 | 2.055 |
| JOURNAL OF NEUROSCIENCE | 14 | 1.918 |
| NEUROSCIENCE LETTERS | 14 | 1.918 |
| DISABILITY AND REHABILITATION | 12 | 1.644 |
| EUROPEAN JOURNAL OF PAIN | 11 | 1.507 |
| JOURNAL OF REHABILITATION MEDICINE | 11 | 1.507 |
| JOURNAL OF PAIN RESEARCH | 9 | 1.233 |
| SPINE | 9 | 1.233 |
| AMERICAN JOURNAL OF PHYSICAL MEDICINE REHABILITATION | 7 | 0.959 |
| NEUROLOGY | 7 | 0.959 |
| PM R | 7 | 0.959 |
| MOLECULAR PAIN | 6 | 0.822 |
| NEUROREHABILITATION AND NEURAL REPAIR | 6 | 0.822 |
| NEUROSCIENCE | 6 | 0.822 |
| PAIN PHYSICIAN | 6 | 0.822 |
| ACTA NEUROCHIRURGICA | 5 | 0.685 |
| ANESTHESIA AND ANALGESIA | 5 | 0.685 |
| PARAPLEGIA | 5 | 0.685 |
| PLOS ONE | 5 | 0.685 |
| BRAIN | 4 | 0.548 |
| BRAIN RESEARCH | 4 | 0.548 |
| BRAIN RESEARCH BULLETIN | 4 | 0.548 |
| GLIA | 4 | 0.548 |
| JOURNAL OF NEUROSURGERY | 4 | 0.548 |
| JOURNAL OF NEUROSURGERY SPINE | 4 | 0.548 |
| SCIENTIFIC REPORTS | 4 | 0.548 |
| CLINICAL NEUROPHYSIOLOGY | 3 | 0.411 |
| CLINICAL REHABILITATION | 3 | 0.411 |
| EUROPEAN JOURNAL OF PHARMACOLOGY | 3 | 0.411 |
| FRONTIERS IN PHYSIOLOGY | 3 | 0.411 |
| JOURNAL OF BACK AND MUSCULOSKELETAL REHABILITATION | 3 | 0.411 |
| JOURNAL OF NEUROCHEMISTRY | 3 | 0.411 |
| JOURNAL OF NEUROINFLAMMATION | 3 | 0.411 |
| JOURNAL OF PHARMACOLOGY AND EXPERIMENTAL THERAPEUTICS | 3 | 0.411 |
| MEDICINE | 3 | 0.411 |
| NEURAL REGENERATION RESEARCH | 3 | 0.411 |
| PAIN MEDICINE | 3 | 0.411 |
| PHYSICAL THERAPY | 3 | 0.411 |
| PROCEEDINGS OF THE NATIONAL ACADEMY OF SCIENCES OF THE UNITED STATES OF AMERICA | 3 | 0.411 |
| SOMATOSENSORY AND MOTOR RESEARCH | 3 | 0.411 |
| SPINE JOURNAL | 3 | 0.411 |
| WORLD NEUROSURGERY | 3 | 0.411 |
| ACTA MEDICA MEDITERRANEA | 2 | 0.274 |
| ANNALS OF NEUROLOGY | 2 | 0.274 |
| ANNALS OF PHYSICAL AND REHABILITATION MEDICINE | 2 | 0.274 |
| ARQUIVOS DE NEURO PSIQUIATRIA | 2 | 0.274 |
| BMC NEUROLOGY | 2 | 0.274 |
| BRAIN RESEARCH REVIEWS | 2 | 0.274 |
| BRITISH JOURNAL OF PHARMACOLOGY | 2 | 0.274 |
| CELL DEATH DISEASE | 2 | 0.274 |
| CLINICAL ORTHOPAEDICS AND RELATED RESEARCH | 2 | 0.274 |
| COMPLEMENTARY THERAPIES IN MEDICINE | 2 | 0.274 |
| CURRENT PAIN AND HEADACHE REPORTS | 2 | 0.274 |
| EUROPEAN JOURNAL OF PHYSICAL AND REHABILITATION MEDICINE | 2 | 0.274 |
| EXPERIMENTAL AND THERAPEUTIC MEDICINE | 2 | 0.274 |
| FRONTIERS IN SYSTEMS NEUROSCIENCE | 2 | 0.274 |
| INTERNATIONAL JOURNAL OF REHABILITATION RESEARCH | 2 | 0.274 |
| JOURNAL OF NEUROLOGY NEUROSURGERY AND PSYCHIATRY | 2 | 0.274 |
| JOURNAL OF NEUROSCIENCE RESEARCH | 2 | 0.274 |
| JOURNAL OF ORTHOPAEDIC SURGERY AND RESEARCH | 2 | 0.274 |
| JOURNAL OF PAIN AND SYMPTOM MANAGEMENT | 2 | 0.274 |
| METABOLIC BRAIN DISEASE | 2 | 0.274 |
| MOLECULAR BRAIN RESEARCH | 2 | 0.274 |
| MOLECULAR THERAPY | 2 | 0.274 |
| NEUROBIOLOGY OF DISEASE | 2 | 0.274 |
| NEUROIMAGE | 2 | 0.274 |
| NEUROMODULATION | 2 | 0.274 |
| NEUROPHARMACOLOGY | 2 | 0.274 |
| NEUROSCIENCE BULLETIN | 2 | 0.274 |
| NEUROSCIENCE RESEARCH | 2 | 0.274 |
| NEUROSCIENCES | 2 | 0.274 |
| PAIN CLINIC | 2 | 0.274 |
| PAIN PRACTICE | 2 | 0.274 |
| PHARMACOLOGY BIOCHEMISTRY AND BEHAVIOR | 2 | 0.274 |
| QUALITY OF LIFE RESEARCH | 2 | 0.274 |
| REGIONAL ANESTHESIA AND PAIN MEDICINE | 2 | 0.274 |
| RESTORATIVE NEUROLOGY AND NEUROSCIENCE | 2 | 0.274 |
| STEM CELL RESEARCH THERAPY | 2 | 0.274 |
| STEM CELLS | 2 | 0.274 |
| ACTA ANAESTHESIOLOGICA SCANDINAVICA | 1 | 0.137 |
| ACTA BIOCHIMICA ET BIOPHYSICA SINICA | 1 | 0.137 |
| ACTA PHARMACOLOGICA SINICA | 1 | 0.137 |
| ACUPUNCTURE IN MEDICINE | 1 | 0.137 |
| ADVANCES IN EXPERIMENTAL MEDICINE AND BIOLOGY | 1 | 0.137 |
| ADVANCES IN PAIN RESEARCH AND THERAPY | 1 | 0.137 |
| AMERICAN JOURNAL OF NEURORADIOLOGY | 1 | 0.137 |
| AMERICAN JOURNAL OF SURGERY | 1 | 0.137 |
| ANAESTHESIA AND INTENSIVE CARE | 1 | 0.137 |
| ANESTHESIOLOGY | 1 | 0.137 |
| ANKARA UNIVERSITESI VETERINER FAKULTESI DERGISI | 1 | 0.137 |
| ANNALS OF SAUDI MEDICINE | 1 | 0.137 |
| ANNALS OF THE NEW YORK ACADEMY OF SCIENCES | 1 | 0.137 |
| AUSTRALIAN OCCUPATIONAL THERAPY JOURNAL | 1 | 0.137 |
| AUTONOMIC NEUROSCIENCE BASIC CLINICAL | 1 | 0.137 |
| BEHAVIOURAL BRAIN RESEARCH | 1 | 0.137 |
| BEHAVIOURAL PHARMACOLOGY | 1 | 0.137 |
| BIOCHIMICA ET BIOPHYSICA ACTA MOLECULAR BASIS OF DISEASE | 1 | 0.137 |
| BIOLOGICAL RESEARCH FOR NURSING | 1 | 0.137 |
| BIOMED RESEARCH INTERNATIONAL | 1 | 0.137 |
| BIOMEDICAL RESEARCH INDIA | 1 | 0.137 |
| BMJ OPEN | 1 | 0.137 |
| BRAIN BEHAVIOR AND IMMUNITY | 1 | 0.137 |
| CANADIAN JOURNAL OF PHYSIOLOGY AND PHARMACOLOGY | 1 | 0.137 |
| CANADIAN MEDICAL ASSOCIATION JOURNAL | 1 | 0.137 |
| CARDIOLOGY IN THE YOUNG | 1 | 0.137 |
| CELL STEM CELL | 1 | 0.137 |
| CELL TRANSPLANTATION | 1 | 0.137 |
| CEREBRAL CORTEX | 1 | 0.137 |
| CHINESE MEDICAL JOURNAL | 1 | 0.137 |
| CLINICAL NEUROLOGY AND NEUROSURGERY | 1 | 0.137 |
| CLINICAL PHARMACOLOGY IN DRUG DEVELOPMENT | 1 | 0.137 |
| CLINICAL THERAPEUTICS | 1 | 0.137 |
| CNS DRUGS | 1 | 0.137 |
| COCHRANE DATABASE OF SYSTEMATIC REVIEWS | 1 | 0.137 |
| COMPUTER METHODS AND PROGRAMS IN BIOMEDICINE | 1 | 0.137 |
| CURRENT OPINION IN NEUROLOGY | 1 | 0.137 |
| CURRENT PHARMACEUTICAL DESIGN | 1 | 0.137 |
| CURRENT PROTEIN PEPTIDE SCIENCE | 1 | 0.137 |
| DRUG DEVELOPMENT RESEARCH | 1 | 0.137 |
| DRUGS | 1 | 0.137 |
| ELECTROMAGNETIC BIOLOGY AND MEDICINE | 1 | 0.137 |
| ENDOCRINOLOGY | 1 | 0.137 |
| ERGONOMICS | 1 | 0.137 |
| EUROPEAN JOURNAL OF CLINICAL PHARMACOLOGY | 1 | 0.137 |
| EUROPEAN JOURNAL OF NEUROLOGY | 1 | 0.137 |
| EUROPEAN JOURNAL OF NEUROSCIENCE | 1 | 0.137 |
| EUROPEAN REVIEW FOR MEDICAL AND PHARMACOLOGICAL SCIENCES | 1 | 0.137 |
| EXPERIMENTAL BRAIN RESEARCH | 1 | 0.137 |
| EXPERT OPINION ON BIOLOGICAL THERAPY | 1 | 0.137 |
| EXPLORE THE JOURNAL OF SCIENCE AND HEALING | 1 | 0.137 |
| FRONTIERS IN CELLULAR NEUROSCIENCE | 1 | 0.137 |
| FRONTIERS IN HUMAN NEUROSCIENCE | 1 | 0.137 |
| FRONTIERS IN NEUROLOGY | 1 | 0.137 |
| FRONTIERS IN NEUROSCIENCE | 1 | 0.137 |
| FRONTIERS IN PHARMACOLOGY | 1 | 0.137 |
| GENE THERAPY | 1 | 0.137 |
| INTERNATIONAL JOURNAL OF INDUSTRIAL ERGONOMICS | 1 | 0.137 |
| INTERNATIONAL JOURNAL OF PHARMACOLOGY | 1 | 0.137 |
| JCPSP JOURNAL OF THE COLLEGE OF PHYSICIANS AND SURGEONS PAKISTAN | 1 | 0.137 |
| JOURNAL OF BIOLOGICAL CHEMISTRY | 1 | 0.137 |
| JOURNAL OF BIOMEDICINE AND BIOTECHNOLOGY | 1 | 0.137 |
| JOURNAL OF CHEMICAL NEUROANATOMY | 1 | 0.137 |
| JOURNAL OF CLINICAL ANESTHESIA | 1 | 0.137 |
| JOURNAL OF CLINICAL NEUROSCIENCE | 1 | 0.137 |
| JOURNAL OF COMPARATIVE NEUROLOGY | 1 | 0.137 |
| JOURNAL OF CONTROLLED RELEASE | 1 | 0.137 |
| JOURNAL OF ENDOCRINOLOGICAL INVESTIGATION | 1 | 0.137 |
| JOURNAL OF KOREAN NEUROSURGICAL SOCIETY | 1 | 0.137 |
| JOURNAL OF MOLECULAR NEUROSCIENCE | 1 | 0.137 |
| JOURNAL OF MUSCULOSKELETAL PAIN | 1 | 0.137 |
| JOURNAL OF NEUROENGINEERING AND REHABILITATION | 1 | 0.137 |
| JOURNAL OF NEUROIMMUNE PHARMACOLOGY | 1 | 0.137 |
| JOURNAL OF NEUROIMMUNOLOGY | 1 | 0.137 |
| JOURNAL OF NEUROLOGIC PHYSICAL THERAPY | 1 | 0.137 |
| JOURNAL OF NEUROPHYSIOLOGY | 1 | 0.137 |
| JOURNAL OF NEUROSCIENCE NURSING | 1 | 0.137 |
| JOURNAL OF NEUROSURGICAL SCIENCES | 1 | 0.137 |
| JOURNAL OF NURSING RESEARCH | 1 | 0.137 |
| JOURNAL OF ORTHOPAEDIC RESEARCH | 1 | 0.137 |
| JOURNAL OF PHARMACOLOGICAL SCIENCES | 1 | 0.137 |
| JOURNAL OF PHYSICAL THERAPY SCIENCE | 1 | 0.137 |
| JOURNAL OF TRANSLATIONAL MEDICINE | 1 | 0.137 |
| KOREAN JOURNAL OF PHYSIOLOGY PHARMACOLOGY | 1 | 0.137 |
| MEDICAL HYPOTHESES | 1 | 0.137 |
| MEDICAL SCIENCE MONITOR | 1 | 0.137 |
| MOLECULAR MEDICINE | 1 | 0.137 |
| MOLECULAR MEDICINE REPORTS | 1 | 0.137 |
| MOLECULAR NEUROBIOLOGY | 1 | 0.137 |
| MOLECULES AND CELLS | 1 | 0.137 |
| NEURAL PLASTICITY | 1 | 0.137 |
| NEUROCHEMICAL RESEARCH | 1 | 0.137 |
| NEUROIMAGE CLINICAL | 1 | 0.137 |
| NEUROLOGICAL RESEARCH | 1 | 0.137 |
| NEURON | 1 | 0.137 |
| NEURONS AND NETWORKS IN THE SPINAL CORD | 1 | 0.137 |
| NEUROPEPTIDES | 1 | 0.137 |
| NEUROPHYSIOLOGY | 1 | 0.137 |
| NEUROREHABILITATION | 1 | 0.137 |
| NEUROSIGNALS | 1 | 0.137 |
| NEUROSURGERY | 1 | 0.137 |
| NEUROTHERAPEUTICS | 1 | 0.137 |
| ONCOLOGY | 1 | 0.137 |
| ORTHOPEDICS | 1 | 0.137 |
| PAKISTAN JOURNAL OF MEDICAL SCIENCES | 1 | 0.137 |
| PHYSICAL MEDICINE AND REHABILITATION CLINICS OF NORTH AMERICA | 1 | 0.137 |
| PHYSIOLOGICAL RESEARCH | 1 | 0.137 |
| REGENERATIVE BIOLOGY OF THE SPINE AND SPINAL CORD | 1 | 0.137 |
| SEMINARS IN IMMUNOLOGY | 1 | 0.137 |
| STEREOTACTIC AND FUNCTIONAL NEUROSURGERY | 1 | 0.137 |
| TOXINS | 1 | 0.137 |
| TRANSLATIONAL NEUROSCIENCE | 1 | 0.137 |
| TRENDS IN NEUROSCIENCES | 1 | 0.137 |
| TURKIYE FIZIKSEL TIP VE REHABILITASYON DERGISI TURKISH JOURNAL OF PHYSICAL MEDICINE AND REHABILITATION | 1 | 0.137 |
| SPINAL CORD | 88 | 12.055 |
| PAIN | 60 | 8.219 |
| ARCHIVES OF PHYSICAL MEDICINE AND REHABILITATION | 41 | 5.616 |
| JOURNAL OF NEUROTRAUMA | 33 | 4.521 |
| JOURNAL OF SPINAL CORD MEDICINE | 28 | 3.836 |
| EXPERIMENTAL NEUROLOGY | 22 | 3.014 |
| CLINICAL JOURNAL OF PAIN | 18 | 2.466 |
| JOURNAL OF PAIN | 18 | 2.466 |
| JOURNAL OF REHABILITATION RESEARCH AND DEVELOPMENT | 15 | 2.055 |
| JOURNAL OF NEUROSCIENCE | 14 | 1.918 |
| NEUROSCIENCE LETTERS | 14 | 1.918 |
| DISABILITY AND REHABILITATION | 12 | 1.644 |
| EUROPEAN JOURNAL OF PAIN | 11 | 1.507 |
| JOURNAL OF REHABILITATION MEDICINE | 11 | 1.507 |
| JOURNAL OF PAIN RESEARCH | 9 | 1.233 |
| SPINE | 9 | 1.233 |
| AMERICAN JOURNAL OF PHYSICAL MEDICINE REHABILITATION | 7 | 0.959 |
| NEUROLOGY | 7 | 0.959 |
| PM R | 7 | 0.959 |
| MOLECULAR PAIN | 6 | 0.822 |
| NEUROREHABILITATION AND NEURAL REPAIR | 6 | 0.822 |
| NEUROSCIENCE | 6 | 0.822 |
| PAIN PHYSICIAN | 6 | 0.822 |
| ACTA NEUROCHIRURGICA | 5 | 0.685 |
| ANESTHESIA AND ANALGESIA | 5 | 0.685 |
| PARAPLEGIA | 5 | 0.685 |
| PLOS ONE | 5 | 0.685 |
| BRAIN | 4 | 0.548 |
| BRAIN RESEARCH | 4 | 0.548 |
| BRAIN RESEARCH BULLETIN | 4 | 0.548 |
| GLIA | 4 | 0.548 |
| JOURNAL OF NEUROSURGERY | 4 | 0.548 |
| JOURNAL OF NEUROSURGERY SPINE | 4 | 0.548 |
| SCIENTIFIC REPORTS | 4 | 0.548 |
| CLINICAL NEUROPHYSIOLOGY | 3 | 0.411 |
| CLINICAL REHABILITATION | 3 | 0.411 |
| EUROPEAN JOURNAL OF PHARMACOLOGY | 3 | 0.411 |
| FRONTIERS IN PHYSIOLOGY | 3 | 0.411 |
| JOURNAL OF BACK AND MUSCULOSKELETAL REHABILITATION | 3 | 0.411 |
| JOURNAL OF NEUROCHEMISTRY | 3 | 0.411 |
| JOURNAL OF NEUROINFLAMMATION | 3 | 0.411 |
| JOURNAL OF PHARMACOLOGY AND EXPERIMENTAL THERAPEUTICS | 3 | 0.411 |
| MEDICINE | 3 | 0.411 |
| NEURAL REGENERATION RESEARCH | 3 | 0.411 |
| PAIN MEDICINE | 3 | 0.411 |
| PHYSICAL THERAPY | 3 | 0.411 |
| PROCEEDINGS OF THE NATIONAL ACADEMY OF SCIENCES OF THE UNITED STATES OF AMERICA | 3 | 0.411 |
| SOMATOSENSORY AND MOTOR RESEARCH | 3 | 0.411 |
| SPINE JOURNAL | 3 | 0.411 |
| WORLD NEUROSURGERY | 3 | 0.411 |
| ACTA MEDICA MEDITERRANEA | 2 | 0.274 |
| ANNALS OF NEUROLOGY | 2 | 0.274 |
| ANNALS OF PHYSICAL AND REHABILITATION MEDICINE | 2 | 0.274 |
| ARQUIVOS DE NEURO PSIQUIATRIA | 2 | 0.274 |
| BMC NEUROLOGY | 2 | 0.274 |
| BRAIN RESEARCH REVIEWS | 2 | 0.274 |
| BRITISH JOURNAL OF PHARMACOLOGY | 2 | 0.274 |
| CELL DEATH DISEASE | 2 | 0.274 |
| CLINICAL ORTHOPAEDICS AND RELATED RESEARCH | 2 | 0.274 |
| COMPLEMENTARY THERAPIES IN MEDICINE | 2 | 0.274 |
| CURRENT PAIN AND HEADACHE REPORTS | 2 | 0.274 |
| EUROPEAN JOURNAL OF PHYSICAL AND REHABILITATION MEDICINE | 2 | 0.274 |
| EXPERIMENTAL AND THERAPEUTIC MEDICINE | 2 | 0.274 |
| FRONTIERS IN SYSTEMS NEUROSCIENCE | 2 | 0.274 |
| INTERNATIONAL JOURNAL OF REHABILITATION RESEARCH | 2 | 0.274 |
| JOURNAL OF NEUROLOGY NEUROSURGERY AND PSYCHIATRY | 2 | 0.274 |
| JOURNAL OF NEUROSCIENCE RESEARCH | 2 | 0.274 |
| JOURNAL OF ORTHOPAEDIC SURGERY AND RESEARCH | 2 | 0.274 |
| JOURNAL OF PAIN AND SYMPTOM MANAGEMENT | 2 | 0.274 |
| METABOLIC BRAIN DISEASE | 2 | 0.274 |
| MOLECULAR BRAIN RESEARCH | 2 | 0.274 |
| MOLECULAR THERAPY | 2 | 0.274 |
| NEUROBIOLOGY OF DISEASE | 2 | 0.274 |
| NEUROIMAGE | 2 | 0.274 |
| NEUROMODULATION | 2 | 0.274 |
| NEUROPHARMACOLOGY | 2 | 0.274 |
| NEUROSCIENCE BULLETIN | 2 | 0.274 |
| NEUROSCIENCE RESEARCH | 2 | 0.274 |
| NEUROSCIENCES | 2 | 0.274 |
| PAIN CLINIC | 2 | 0.274 |
| PAIN PRACTICE | 2 | 0.274 |
| PHARMACOLOGY BIOCHEMISTRY AND BEHAVIOR | 2 | 0.274 |
| QUALITY OF LIFE RESEARCH | 2 | 0.274 |
| REGIONAL ANESTHESIA AND PAIN MEDICINE | 2 | 0.274 |
| RESTORATIVE NEUROLOGY AND NEUROSCIENCE | 2 | 0.274 |
| STEM CELL RESEARCH THERAPY | 2 | 0.274 |
| STEM CELLS | 2 | 0.274 |
| ACTA ANAESTHESIOLOGICA SCANDINAVICA | 1 | 0.137 |
| ACTA BIOCHIMICA ET BIOPHYSICA SINICA | 1 | 0.137 |
| ACTA PHARMACOLOGICA SINICA | 1 | 0.137 |
| ACUPUNCTURE IN MEDICINE | 1 | 0.137 |
| ADVANCES IN EXPERIMENTAL MEDICINE AND BIOLOGY | 1 | 0.137 |
| ADVANCES IN PAIN RESEARCH AND THERAPY | 1 | 0.137 |
| AMERICAN JOURNAL OF NEURORADIOLOGY | 1 | 0.137 |
| AMERICAN JOURNAL OF SURGERY | 1 | 0.137 |
| ANAESTHESIA AND INTENSIVE CARE | 1 | 0.137 |
| ANESTHESIOLOGY | 1 | 0.137 |
| ANKARA UNIVERSITESI VETERINER FAKULTESI DERGISI | 1 | 0.137 |
| ANNALS OF SAUDI MEDICINE | 1 | 0.137 |
| ANNALS OF THE NEW YORK ACADEMY OF SCIENCES | 1 | 0.137 |
| AUSTRALIAN OCCUPATIONAL THERAPY JOURNAL | 1 | 0.137 |
| AUTONOMIC NEUROSCIENCE BASIC CLINICAL | 1 | 0.137 |
| BEHAVIOURAL BRAIN RESEARCH | 1 | 0.137 |
| BEHAVIOURAL PHARMACOLOGY | 1 | 0.137 |
| BIOCHIMICA ET BIOPHYSICA ACTA MOLECULAR BASIS OF DISEASE | 1 | 0.137 |
| BIOLOGICAL RESEARCH FOR NURSING | 1 | 0.137 |
| BIOMED RESEARCH INTERNATIONAL | 1 | 0.137 |
| BIOMEDICAL RESEARCH INDIA | 1 | 0.137 |
| BMJ OPEN | 1 | 0.137 |
| BRAIN BEHAVIOR AND IMMUNITY | 1 | 0.137 |
| CANADIAN JOURNAL OF PHYSIOLOGY AND PHARMACOLOGY | 1 | 0.137 |
| CANADIAN MEDICAL ASSOCIATION JOURNAL | 1 | 0.137 |
| CARDIOLOGY IN THE YOUNG | 1 | 0.137 |
| CELL STEM CELL | 1 | 0.137 |
| CELL TRANSPLANTATION | 1 | 0.137 |
| CEREBRAL CORTEX | 1 | 0.137 |
| CHINESE MEDICAL JOURNAL | 1 | 0.137 |
| CLINICAL NEUROLOGY AND NEUROSURGERY | 1 | 0.137 |
| CLINICAL PHARMACOLOGY IN DRUG DEVELOPMENT | 1 | 0.137 |
| CLINICAL THERAPEUTICS | 1 | 0.137 |
| CNS DRUGS | 1 | 0.137 |
| COCHRANE DATABASE OF SYSTEMATIC REVIEWS | 1 | 0.137 |
| COMPUTER METHODS AND PROGRAMS IN BIOMEDICINE | 1 | 0.137 |
| CURRENT OPINION IN NEUROLOGY | 1 | 0.137 |
| CURRENT PHARMACEUTICAL DESIGN | 1 | 0.137 |
| CURRENT PROTEIN PEPTIDE SCIENCE | 1 | 0.137 |
| DRUG DEVELOPMENT RESEARCH | 1 | 0.137 |
| DRUGS | 1 | 0.137 |
| ELECTROMAGNETIC BIOLOGY AND MEDICINE | 1 | 0.137 |
| ENDOCRINOLOGY | 1 | 0.137 |
| ERGONOMICS | 1 | 0.137 |
| EUROPEAN JOURNAL OF CLINICAL PHARMACOLOGY | 1 | 0.137 |
| EUROPEAN JOURNAL OF NEUROLOGY | 1 | 0.137 |
| EUROPEAN JOURNAL OF NEUROSCIENCE | 1 | 0.137 |
| EUROPEAN REVIEW FOR MEDICAL AND PHARMACOLOGICAL SCIENCES | 1 | 0.137 |
| EXPERIMENTAL BRAIN RESEARCH | 1 | 0.137 |
| EXPERT OPINION ON BIOLOGICAL THERAPY | 1 | 0.137 |
| EXPLORE THE JOURNAL OF SCIENCE AND HEALING | 1 | 0.137 |
| FRONTIERS IN CELLULAR NEUROSCIENCE | 1 | 0.137 |
| FRONTIERS IN HUMAN NEUROSCIENCE | 1 | 0.137 |
| FRONTIERS IN NEUROLOGY | 1 | 0.137 |
| FRONTIERS IN NEUROSCIENCE | 1 | 0.137 |
| FRONTIERS IN PHARMACOLOGY | 1 | 0.137 |
| GENE THERAPY | 1 | 0.137 |
| INTERNATIONAL JOURNAL OF INDUSTRIAL ERGONOMICS | 1 | 0.137 |
| INTERNATIONAL JOURNAL OF PHARMACOLOGY | 1 | 0.137 |
| JCPSP JOURNAL OF THE COLLEGE OF PHYSICIANS AND SURGEONS PAKISTAN | 1 | 0.137 |
| JOURNAL OF BIOLOGICAL CHEMISTRY | 1 | 0.137 |
| JOURNAL OF BIOMEDICINE AND BIOTECHNOLOGY | 1 | 0.137 |
| JOURNAL OF CHEMICAL NEUROANATOMY | 1 | 0.137 |
| JOURNAL OF CLINICAL ANESTHESIA | 1 | 0.137 |
| JOURNAL OF CLINICAL NEUROSCIENCE | 1 | 0.137 |
| JOURNAL OF COMPARATIVE NEUROLOGY | 1 | 0.137 |
| JOURNAL OF CONTROLLED RELEASE | 1 | 0.137 |
| JOURNAL OF ENDOCRINOLOGICAL INVESTIGATION | 1 | 0.137 |
| JOURNAL OF KOREAN NEUROSURGICAL SOCIETY | 1 | 0.137 |
| JOURNAL OF MOLECULAR NEUROSCIENCE | 1 | 0.137 |
| JOURNAL OF MUSCULOSKELETAL PAIN | 1 | 0.137 |
| JOURNAL OF NEUROENGINEERING AND REHABILITATION | 1 | 0.137 |
| JOURNAL OF NEUROIMMUNE PHARMACOLOGY | 1 | 0.137 |
| JOURNAL OF NEUROIMMUNOLOGY | 1 | 0.137 |
| JOURNAL OF NEUROLOGIC PHYSICAL THERAPY | 1 | 0.137 |
| JOURNAL OF NEUROPHYSIOLOGY | 1 | 0.137 |
| JOURNAL OF NEUROSCIENCE NURSING | 1 | 0.137 |
| JOURNAL OF NEUROSURGICAL SCIENCES | 1 | 0.137 |
| JOURNAL OF NURSING RESEARCH | 1 | 0.137 |
| JOURNAL OF ORTHOPAEDIC RESEARCH | 1 | 0.137 |
| JOURNAL OF PHARMACOLOGICAL SCIENCES | 1 | 0.137 |
| JOURNAL OF PHYSICAL THERAPY SCIENCE | 1 | 0.137 |
| JOURNAL OF TRANSLATIONAL MEDICINE | 1 | 0.137 |
| KOREAN JOURNAL OF PHYSIOLOGY PHARMACOLOGY | 1 | 0.137 |
| MEDICAL HYPOTHESES | 1 | 0.137 |
| MEDICAL SCIENCE MONITOR | 1 | 0.137 |
| MOLECULAR MEDICINE | 1 | 0.137 |
| MOLECULAR MEDICINE REPORTS | 1 | 0.137 |
| MOLECULAR NEUROBIOLOGY | 1 | 0.137 |
| MOLECULES AND CELLS | 1 | 0.137 |
| NEURAL PLASTICITY | 1 | 0.137 |
| NEUROCHEMICAL RESEARCH | 1 | 0.137 |
| NEUROIMAGE CLINICAL | 1 | 0.137 |
| NEUROLOGICAL RESEARCH | 1 | 0.137 |
| NEURON | 1 | 0.137 |
| NEURONS AND NETWORKS IN THE SPINAL CORD | 1 | 0.137 |
| NEUROPEPTIDES | 1 | 0.137 |
| NEUROPHYSIOLOGY | 1 | 0.137 |
| NEUROREHABILITATION | 1 | 0.137 |
| NEUROSIGNALS | 1 | 0.137 |
| NEUROSURGERY | 1 | 0.137 |
| NEUROTHERAPEUTICS | 1 | 0.137 |
| ONCOLOGY | 1 | 0.137 |
| ORTHOPEDICS | 1 | 0.137 |
| PAKISTAN JOURNAL OF MEDICAL SCIENCES | 1 | 0.137 |
| PHYSICAL MEDICINE AND REHABILITATION CLINICS OF NORTH AMERICA | 1 | 0.137 |
| PHYSIOLOGICAL RESEARCH | 1 | 0.137 |
| REGENERATIVE BIOLOGY OF THE SPINE AND SPINAL CORD | 1 | 0.137 |
| SEMINARS IN IMMUNOLOGY | 1 | 0.137 |
| STEREOTACTIC AND FUNCTIONAL NEUROSURGERY | 1 | 0.137 |
| TOXINS | 1 | 0.137 |
| TRANSLATIONAL NEUROSCIENCE | 1 | 0.137 |
| TRENDS IN NEUROSCIENCES | 1 | 0.137 |
| TURKIYE FIZIKSEL TIP VE REHABILITASYON DERGISI TURKISH JOURNAL OF PHYSICAL MEDICINE AND REHABILITATION | 1 | 0.137 |
| SPINAL CORD | 88 | 12.055 |
| PAIN | 60 | 8.219 |
| ARCHIVES OF PHYSICAL MEDICINE AND REHABILITATION | 41 | 5.616 |
| JOURNAL OF NEUROTRAUMA | 33 | 4.521 |
| JOURNAL OF SPINAL CORD MEDICINE | 28 | 3.836 |
| EXPERIMENTAL NEUROLOGY | 22 | 3.014 |
| CLINICAL JOURNAL OF PAIN | 18 | 2.466 |
| JOURNAL OF PAIN | 18 | 2.466 |
| JOURNAL OF REHABILITATION RESEARCH AND DEVELOPMENT | 15 | 2.055 |
| JOURNAL OF NEUROSCIENCE | 14 | 1.918 |
| NEUROSCIENCE LETTERS | 14 | 1.918 |
| DISABILITY AND REHABILITATION | 12 | 1.644 |
| EUROPEAN JOURNAL OF PAIN | 11 | 1.507 |
| JOURNAL OF REHABILITATION MEDICINE | 11 | 1.507 |
| JOURNAL OF PAIN RESEARCH | 9 | 1.233 |
| SPINE | 9 | 1.233 |
| AMERICAN JOURNAL OF PHYSICAL MEDICINE REHABILITATION | 7 | 0.959 |
| NEUROLOGY | 7 | 0.959 |
| PM R | 7 | 0.959 |
| MOLECULAR PAIN | 6 | 0.822 |
| NEUROREHABILITATION AND NEURAL REPAIR | 6 | 0.822 |
| NEUROSCIENCE | 6 | 0.822 |
| PAIN PHYSICIAN | 6 | 0.822 |
| ACTA NEUROCHIRURGICA | 5 | 0.685 |
| ANESTHESIA AND ANALGESIA | 5 | 0.685 |
| PARAPLEGIA | 5 | 0.685 |
| PLOS ONE | 5 | 0.685 |
| BRAIN | 4 | 0.548 |
| BRAIN RESEARCH | 4 | 0.548 |
| BRAIN RESEARCH BULLETIN | 4 | 0.548 |
| GLIA | 4 | 0.548 |
| JOURNAL OF NEUROSURGERY | 4 | 0.548 |
| JOURNAL OF NEUROSURGERY SPINE | 4 | 0.548 |
| SCIENTIFIC REPORTS | 4 | 0.548 |
| CLINICAL NEUROPHYSIOLOGY | 3 | 0.411 |
| CLINICAL REHABILITATION | 3 | 0.411 |
| EUROPEAN JOURNAL OF PHARMACOLOGY | 3 | 0.411 |
| FRONTIERS IN PHYSIOLOGY | 3 | 0.411 |
| JOURNAL OF BACK AND MUSCULOSKELETAL REHABILITATION | 3 | 0.411 |
| JOURNAL OF NEUROCHEMISTRY | 3 | 0.411 |
| JOURNAL OF NEUROINFLAMMATION | 3 | 0.411 |
| JOURNAL OF PHARMACOLOGY AND EXPERIMENTAL THERAPEUTICS | 3 | 0.411 |
| MEDICINE | 3 | 0.411 |
| NEURAL REGENERATION RESEARCH | 3 | 0.411 |
| PAIN MEDICINE | 3 | 0.411 |
| PHYSICAL THERAPY | 3 | 0.411 |
| PROCEEDINGS OF THE NATIONAL ACADEMY OF SCIENCES OF THE UNITED STATES OF AMERICA | 3 | 0.411 |
| SOMATOSENSORY AND MOTOR RESEARCH | 3 | 0.411 |
| SPINE JOURNAL | 3 | 0.411 |
| WORLD NEUROSURGERY | 3 | 0.411 |
| ACTA MEDICA MEDITERRANEA | 2 | 0.274 |
| ANNALS OF NEUROLOGY | 2 | 0.274 |
| ANNALS OF PHYSICAL AND REHABILITATION MEDICINE | 2 | 0.274 |
| ARQUIVOS DE NEURO PSIQUIATRIA | 2 | 0.274 |
| BMC NEUROLOGY | 2 | 0.274 |
| BRAIN RESEARCH REVIEWS | 2 | 0.274 |
| BRITISH JOURNAL OF PHARMACOLOGY | 2 | 0.274 |
| CELL DEATH DISEASE | 2 | 0.274 |
| CLINICAL ORTHOPAEDICS AND RELATED RESEARCH | 2 | 0.274 |
| COMPLEMENTARY THERAPIES IN MEDICINE | 2 | 0.274 |
| CURRENT PAIN AND HEADACHE REPORTS | 2 | 0.274 |
| EUROPEAN JOURNAL OF PHYSICAL AND REHABILITATION MEDICINE | 2 | 0.274 |
| EXPERIMENTAL AND THERAPEUTIC MEDICINE | 2 | 0.274 |
| FRONTIERS IN SYSTEMS NEUROSCIENCE | 2 | 0.274 |
| INTERNATIONAL JOURNAL OF REHABILITATION RESEARCH | 2 | 0.274 |
| JOURNAL OF NEUROLOGY NEUROSURGERY AND PSYCHIATRY | 2 | 0.274 |
| JOURNAL OF NEUROSCIENCE RESEARCH | 2 | 0.274 |
| JOURNAL OF ORTHOPAEDIC SURGERY AND RESEARCH | 2 | 0.274 |
| JOURNAL OF PAIN AND SYMPTOM MANAGEMENT | 2 | 0.274 |
| METABOLIC BRAIN DISEASE | 2 | 0.274 |
| MOLECULAR BRAIN RESEARCH | 2 | 0.274 |
| MOLECULAR THERAPY | 2 | 0.274 |
| NEUROBIOLOGY OF DISEASE | 2 | 0.274 |
| NEUROIMAGE | 2 | 0.274 |
| NEUROMODULATION | 2 | 0.274 |
| NEUROPHARMACOLOGY | 2 | 0.274 |
| NEUROSCIENCE BULLETIN | 2 | 0.274 |
| NEUROSCIENCE RESEARCH | 2 | 0.274 |
| NEUROSCIENCES | 2 | 0.274 |
| PAIN CLINIC | 2 | 0.274 |
| PAIN PRACTICE | 2 | 0.274 |
| PHARMACOLOGY BIOCHEMISTRY AND BEHAVIOR | 2 | 0.274 |
| QUALITY OF LIFE RESEARCH | 2 | 0.274 |
| REGIONAL ANESTHESIA AND PAIN MEDICINE | 2 | 0.274 |
| RESTORATIVE NEUROLOGY AND NEUROSCIENCE | 2 | 0.274 |
| STEM CELL RESEARCH THERAPY | 2 | 0.274 |
| STEM CELLS | 2 | 0.274 |
| ACTA ANAESTHESIOLOGICA SCANDINAVICA | 1 | 0.137 |
| ACTA BIOCHIMICA ET BIOPHYSICA SINICA | 1 | 0.137 |
| ACTA PHARMACOLOGICA SINICA | 1 | 0.137 |
| ACUPUNCTURE IN MEDICINE | 1 | 0.137 |
| ADVANCES IN EXPERIMENTAL MEDICINE AND BIOLOGY | 1 | 0.137 |
| ADVANCES IN PAIN RESEARCH AND THERAPY | 1 | 0.137 |
| AMERICAN JOURNAL OF NEURORADIOLOGY | 1 | 0.137 |
| AMERICAN JOURNAL OF SURGERY | 1 | 0.137 |
| ANAESTHESIA AND INTENSIVE CARE | 1 | 0.137 |
| ANESTHESIOLOGY | 1 | 0.137 |
| ANKARA UNIVERSITESI VETERINER FAKULTESI DERGISI | 1 | 0.137 |
| ANNALS OF SAUDI MEDICINE | 1 | 0.137 |
| ANNALS OF THE NEW YORK ACADEMY OF SCIENCES | 1 | 0.137 |
| AUSTRALIAN OCCUPATIONAL THERAPY JOURNAL | 1 | 0.137 |
| AUTONOMIC NEUROSCIENCE BASIC CLINICAL | 1 | 0.137 |
| BEHAVIOURAL BRAIN RESEARCH | 1 | 0.137 |
| BEHAVIOURAL PHARMACOLOGY | 1 | 0.137 |
| BIOCHIMICA ET BIOPHYSICA ACTA MOLECULAR BASIS OF DISEASE | 1 | 0.137 |
| BIOLOGICAL RESEARCH FOR NURSING | 1 | 0.137 |
| BIOMED RESEARCH INTERNATIONAL | 1 | 0.137 |
| BIOMEDICAL RESEARCH INDIA | 1 | 0.137 |
| BMJ OPEN | 1 | 0.137 |
| BRAIN BEHAVIOR AND IMMUNITY | 1 | 0.137 |
| CANADIAN JOURNAL OF PHYSIOLOGY AND PHARMACOLOGY | 1 | 0.137 |
| CANADIAN MEDICAL ASSOCIATION JOURNAL | 1 | 0.137 |
| CARDIOLOGY IN THE YOUNG | 1 | 0.137 |
| CELL STEM CELL | 1 | 0.137 |
| CELL TRANSPLANTATION | 1 | 0.137 |
| CEREBRAL CORTEX | 1 | 0.137 |
| CHINESE MEDICAL JOURNAL | 1 | 0.137 |
| CLINICAL NEUROLOGY AND NEUROSURGERY | 1 | 0.137 |
| CLINICAL PHARMACOLOGY IN DRUG DEVELOPMENT | 1 | 0.137 |
| CLINICAL THERAPEUTICS | 1 | 0.137 |
| CNS DRUGS | 1 | 0.137 |
| COCHRANE DATABASE OF SYSTEMATIC REVIEWS | 1 | 0.137 |
| COMPUTER METHODS AND PROGRAMS IN BIOMEDICINE | 1 | 0.137 |
| CURRENT OPINION IN NEUROLOGY | 1 | 0.137 |
| CURRENT PHARMACEUTICAL DESIGN | 1 | 0.137 |
| CURRENT PROTEIN PEPTIDE SCIENCE | 1 | 0.137 |
| DRUG DEVELOPMENT RESEARCH | 1 | 0.137 |
| DRUGS | 1 | 0.137 |
| ELECTROMAGNETIC BIOLOGY AND MEDICINE | 1 | 0.137 |
| ENDOCRINOLOGY | 1 | 0.137 |
| ERGONOMICS | 1 | 0.137 |
| EUROPEAN JOURNAL OF CLINICAL PHARMACOLOGY | 1 | 0.137 |
| EUROPEAN JOURNAL OF NEUROLOGY | 1 | 0.137 |
| EUROPEAN JOURNAL OF NEUROSCIENCE | 1 | 0.137 |
| EUROPEAN REVIEW FOR MEDICAL AND PHARMACOLOGICAL SCIENCES | 1 | 0.137 |
| EXPERIMENTAL BRAIN RESEARCH | 1 | 0.137 |
| EXPERT OPINION ON BIOLOGICAL THERAPY | 1 | 0.137 |
| EXPLORE THE JOURNAL OF SCIENCE AND HEALING | 1 | 0.137 |
| FRONTIERS IN CELLULAR NEUROSCIENCE | 1 | 0.137 |
| FRONTIERS IN HUMAN NEUROSCIENCE | 1 | 0.137 |
| FRONTIERS IN NEUROLOGY | 1 | 0.137 |
| FRONTIERS IN NEUROSCIENCE | 1 | 0.137 |
| FRONTIERS IN PHARMACOLOGY | 1 | 0.137 |
| GENE THERAPY | 1 | 0.137 |
| INTERNATIONAL JOURNAL OF INDUSTRIAL ERGONOMICS | 1 | 0.137 |
| INTERNATIONAL JOURNAL OF PHARMACOLOGY | 1 | 0.137 |
| JCPSP JOURNAL OF THE COLLEGE OF PHYSICIANS AND SURGEONS PAKISTAN | 1 | 0.137 |
| JOURNAL OF BIOLOGICAL CHEMISTRY | 1 | 0.137 |
| JOURNAL OF BIOMEDICINE AND BIOTECHNOLOGY | 1 | 0.137 |
| JOURNAL OF CHEMICAL NEUROANATOMY | 1 | 0.137 |
| JOURNAL OF CLINICAL ANESTHESIA | 1 | 0.137 |
| JOURNAL OF CLINICAL NEUROSCIENCE | 1 | 0.137 |
| JOURNAL OF COMPARATIVE NEUROLOGY | 1 | 0.137 |
| JOURNAL OF CONTROLLED RELEASE | 1 | 0.137 |
| JOURNAL OF ENDOCRINOLOGICAL INVESTIGATION | 1 | 0.137 |
| JOURNAL OF KOREAN NEUROSURGICAL SOCIETY | 1 | 0.137 |
| JOURNAL OF MOLECULAR NEUROSCIENCE | 1 | 0.137 |
| JOURNAL OF MUSCULOSKELETAL PAIN | 1 | 0.137 |
| JOURNAL OF NEUROENGINEERING AND REHABILITATION | 1 | 0.137 |
| JOURNAL OF NEUROIMMUNE PHARMACOLOGY | 1 | 0.137 |
| JOURNAL OF NEUROIMMUNOLOGY | 1 | 0.137 |
| JOURNAL OF NEUROLOGIC PHYSICAL THERAPY | 1 | 0.137 |
| JOURNAL OF NEUROPHYSIOLOGY | 1 | 0.137 |
| JOURNAL OF NEUROSCIENCE NURSING | 1 | 0.137 |
| JOURNAL OF NEUROSURGICAL SCIENCES | 1 | 0.137 |
| JOURNAL OF NURSING RESEARCH | 1 | 0.137 |
| JOURNAL OF ORTHOPAEDIC RESEARCH | 1 | 0.137 |
| JOURNAL OF PHARMACOLOGICAL SCIENCES | 1 | 0.137 |
| JOURNAL OF PHYSICAL THERAPY SCIENCE | 1 | 0.137 |
| JOURNAL OF TRANSLATIONAL MEDICINE | 1 | 0.137 |
| KOREAN JOURNAL OF PHYSIOLOGY PHARMACOLOGY | 1 | 0.137 |
| MEDICAL HYPOTHESES | 1 | 0.137 |
| MEDICAL SCIENCE MONITOR | 1 | 0.137 |
| MOLECULAR MEDICINE | 1 | 0.137 |
| MOLECULAR MEDICINE REPORTS | 1 | 0.137 |
| MOLECULAR NEUROBIOLOGY | 1 | 0.137 |
| MOLECULES AND CELLS | 1 | 0.137 |
| NEURAL PLASTICITY | 1 | 0.137 |
| NEUROCHEMICAL RESEARCH | 1 | 0.137 |
| NEUROIMAGE CLINICAL | 1 | 0.137 |
| NEUROLOGICAL RESEARCH | 1 | 0.137 |
| NEURON | 1 | 0.137 |
| NEURONS AND NETWORKS IN THE SPINAL CORD | 1 | 0.137 |
| NEUROPEPTIDES | 1 | 0.137 |
| NEUROPHYSIOLOGY | 1 | 0.137 |
| NEUROREHABILITATION | 1 | 0.137 |
| NEUROSIGNALS | 1 | 0.137 |
| NEUROSURGERY | 1 | 0.137 |
| NEUROTHERAPEUTICS | 1 | 0.137 |
| ONCOLOGY | 1 | 0.137 |
| ORTHOPEDICS | 1 | 0.137 |
| PAKISTAN JOURNAL OF MEDICAL SCIENCES | 1 | 0.137 |
| PHYSICAL MEDICINE AND REHABILITATION CLINICS OF NORTH AMERICA | 1 | 0.137 |
| PHYSIOLOGICAL RESEARCH | 1 | 0.137 |
| REGENERATIVE BIOLOGY OF THE SPINE AND SPINAL CORD | 1 | 0.137 |
| SEMINARS IN IMMUNOLOGY | 1 | 0.137 |
| STEREOTACTIC AND FUNCTIONAL NEUROSURGERY | 1 | 0.137 |
| TOXINS | 1 | 0.137 |
| TRANSLATIONAL NEUROSCIENCE | 1 | 0.137 |
| TRENDS IN NEUROSCIENCES | 1 | 0.137 |
| TURKIYE FIZIKSEL TIP VE REHABILITASYON DERGISI TURKISH JOURNAL OF PHYSICAL MEDICINE AND REHABILITATION | 1 | 0.137 |
| SPINAL CORD | 88 | 12.055 |
| PAIN | 60 | 8.219 |
| ARCHIVES OF PHYSICAL MEDICINE AND REHABILITATION | 41 | 5.616 |
| JOURNAL OF NEUROTRAUMA | 33 | 4.521 |
| JOURNAL OF SPINAL CORD MEDICINE | 28 | 3.836 |
| EXPERIMENTAL NEUROLOGY | 22 | 3.014 |
| CLINICAL JOURNAL OF PAIN | 18 | 2.466 |
| JOURNAL OF PAIN | 18 | 2.466 |
| JOURNAL OF REHABILITATION RESEARCH AND DEVELOPMENT | 15 | 2.055 |
| JOURNAL OF NEUROSCIENCE | 14 | 1.918 |
| NEUROSCIENCE LETTERS | 14 | 1.918 |
| DISABILITY AND REHABILITATION | 12 | 1.644 |
| EUROPEAN JOURNAL OF PAIN | 11 | 1.507 |
| JOURNAL OF REHABILITATION MEDICINE | 11 | 1.507 |
| JOURNAL OF PAIN RESEARCH | 9 | 1.233 |
| SPINE | 9 | 1.233 |
| AMERICAN JOURNAL OF PHYSICAL MEDICINE REHABILITATION | 7 | 0.959 |
| NEUROLOGY | 7 | 0.959 |
| PM R | 7 | 0.959 |
| MOLECULAR PAIN | 6 | 0.822 |
| NEUROREHABILITATION AND NEURAL REPAIR | 6 | 0.822 |
| NEUROSCIENCE | 6 | 0.822 |
| PAIN PHYSICIAN | 6 | 0.822 |
| ACTA NEUROCHIRURGICA | 5 | 0.685 |
| ANESTHESIA AND ANALGESIA | 5 | 0.685 |
| PARAPLEGIA | 5 | 0.685 |
| PLOS ONE | 5 | 0.685 |
| BRAIN | 4 | 0.548 |
| BRAIN RESEARCH | 4 | 0.548 |
| BRAIN RESEARCH BULLETIN | 4 | 0.548 |
| GLIA | 4 | 0.548 |
| JOURNAL OF NEUROSURGERY | 4 | 0.548 |
| JOURNAL OF NEUROSURGERY SPINE | 4 | 0.548 |
| SCIENTIFIC REPORTS | 4 | 0.548 |
| CLINICAL NEUROPHYSIOLOGY | 3 | 0.411 |
| CLINICAL REHABILITATION | 3 | 0.411 |
| EUROPEAN JOURNAL OF PHARMACOLOGY | 3 | 0.411 |
| FRONTIERS IN PHYSIOLOGY | 3 | 0.411 |
| JOURNAL OF BACK AND MUSCULOSKELETAL REHABILITATION | 3 | 0.411 |
| JOURNAL OF NEUROCHEMISTRY | 3 | 0.411 |
| JOURNAL OF NEUROINFLAMMATION | 3 | 0.411 |
| JOURNAL OF PHARMACOLOGY AND EXPERIMENTAL THERAPEUTICS | 3 | 0.411 |
| MEDICINE | 3 | 0.411 |
| NEURAL REGENERATION RESEARCH | 3 | 0.411 |
| PAIN MEDICINE | 3 | 0.411 |
| PHYSICAL THERAPY | 3 | 0.411 |
| PROCEEDINGS OF THE NATIONAL ACADEMY OF SCIENCES OF THE UNITED STATES OF AMERICA | 3 | 0.411 |
| SOMATOSENSORY AND MOTOR RESEARCH | 3 | 0.411 |
| SPINE JOURNAL | 3 | 0.411 |
| WORLD NEUROSURGERY | 3 | 0.411 |
| ACTA MEDICA MEDITERRANEA | 2 | 0.274 |
| ANNALS OF NEUROLOGY | 2 | 0.274 |
| ANNALS OF PHYSICAL AND REHABILITATION MEDICINE | 2 | 0.274 |
| ARQUIVOS DE NEURO PSIQUIATRIA | 2 | 0.274 |
| BMC NEUROLOGY | 2 | 0.274 |
| BRAIN RESEARCH REVIEWS | 2 | 0.274 |
| BRITISH JOURNAL OF PHARMACOLOGY | 2 | 0.274 |
| CELL DEATH DISEASE | 2 | 0.274 |
| CLINICAL ORTHOPAEDICS AND RELATED RESEARCH | 2 | 0.274 |
| COMPLEMENTARY THERAPIES IN MEDICINE | 2 | 0.274 |
| CURRENT PAIN AND HEADACHE REPORTS | 2 | 0.274 |
| EUROPEAN JOURNAL OF PHYSICAL AND REHABILITATION MEDICINE | 2 | 0.274 |
| EXPERIMENTAL AND THERAPEUTIC MEDICINE | 2 | 0.274 |
| FRONTIERS IN SYSTEMS NEUROSCIENCE | 2 | 0.274 |
| INTERNATIONAL JOURNAL OF REHABILITATION RESEARCH | 2 | 0.274 |
| JOURNAL OF NEUROLOGY NEUROSURGERY AND PSYCHIATRY | 2 | 0.274 |
| JOURNAL OF NEUROSCIENCE RESEARCH | 2 | 0.274 |
| JOURNAL OF ORTHOPAEDIC SURGERY AND RESEARCH | 2 | 0.274 |
| JOURNAL OF PAIN AND SYMPTOM MANAGEMENT | 2 | 0.274 |
| METABOLIC BRAIN DISEASE | 2 | 0.274 |
| MOLECULAR BRAIN RESEARCH | 2 | 0.274 |
| MOLECULAR THERAPY | 2 | 0.274 |
| NEUROBIOLOGY OF DISEASE | 2 | 0.274 |
| NEUROIMAGE | 2 | 0.274 |
| NEUROMODULATION | 2 | 0.274 |
| NEUROPHARMACOLOGY | 2 | 0.274 |
| NEUROSCIENCE BULLETIN | 2 | 0.274 |
| NEUROSCIENCE RESEARCH | 2 | 0.274 |
| NEUROSCIENCES | 2 | 0.274 |
| PAIN CLINIC | 2 | 0.274 |
| PAIN PRACTICE | 2 | 0.274 |
| PHARMACOLOGY BIOCHEMISTRY AND BEHAVIOR | 2 | 0.274 |
| QUALITY OF LIFE RESEARCH | 2 | 0.274 |
| REGIONAL ANESTHESIA AND PAIN MEDICINE | 2 | 0.274 |
| RESTORATIVE NEUROLOGY AND NEUROSCIENCE | 2 | 0.274 |
| STEM CELL RESEARCH THERAPY | 2 | 0.274 |
| STEM CELLS | 2 | 0.274 |
| ACTA ANAESTHESIOLOGICA SCANDINAVICA | 1 | 0.137 |
| ACTA BIOCHIMICA ET BIOPHYSICA SINICA | 1 | 0.137 |
| ACTA PHARMACOLOGICA SINICA | 1 | 0.137 |
| ACUPUNCTURE IN MEDICINE | 1 | 0.137 |
| ADVANCES IN EXPERIMENTAL MEDICINE AND BIOLOGY | 1 | 0.137 |
| ADVANCES IN PAIN RESEARCH AND THERAPY | 1 | 0.137 |
| AMERICAN JOURNAL OF NEURORADIOLOGY | 1 | 0.137 |
| AMERICAN JOURNAL OF SURGERY | 1 | 0.137 |
| ANAESTHESIA AND INTENSIVE CARE | 1 | 0.137 |
| ANESTHESIOLOGY | 1 | 0.137 |
| ANKARA UNIVERSITESI VETERINER FAKULTESI DERGISI | 1 | 0.137 |
| ANNALS OF SAUDI MEDICINE | 1 | 0.137 |
| ANNALS OF THE NEW YORK ACADEMY OF SCIENCES | 1 | 0.137 |
| AUSTRALIAN OCCUPATIONAL THERAPY JOURNAL | 1 | 0.137 |
| AUTONOMIC NEUROSCIENCE BASIC CLINICAL | 1 | 0.137 |
| BEHAVIOURAL BRAIN RESEARCH | 1 | 0.137 |
| BEHAVIOURAL PHARMACOLOGY | 1 | 0.137 |
| BIOCHIMICA ET BIOPHYSICA ACTA MOLECULAR BASIS OF DISEASE | 1 | 0.137 |
| BIOLOGICAL RESEARCH FOR NURSING | 1 | 0.137 |
| BIOMED RESEARCH INTERNATIONAL | 1 | 0.137 |
| BIOMEDICAL RESEARCH INDIA | 1 | 0.137 |
| BMJ OPEN | 1 | 0.137 |
| BRAIN BEHAVIOR AND IMMUNITY | 1 | 0.137 |
| CANADIAN JOURNAL OF PHYSIOLOGY AND PHARMACOLOGY | 1 | 0.137 |
| CANADIAN MEDICAL ASSOCIATION JOURNAL | 1 | 0.137 |
| CARDIOLOGY IN THE YOUNG | 1 | 0.137 |
| CELL STEM CELL | 1 | 0.137 |
| CELL TRANSPLANTATION | 1 | 0.137 |
| CEREBRAL CORTEX | 1 | 0.137 |
| CHINESE MEDICAL JOURNAL | 1 | 0.137 |
| CLINICAL NEUROLOGY AND NEUROSURGERY | 1 | 0.137 |
| CLINICAL PHARMACOLOGY IN DRUG DEVELOPMENT | 1 | 0.137 |
| CLINICAL THERAPEUTICS | 1 | 0.137 |
| CNS DRUGS | 1 | 0.137 |
| COCHRANE DATABASE OF SYSTEMATIC REVIEWS | 1 | 0.137 |
| COMPUTER METHODS AND PROGRAMS IN BIOMEDICINE | 1 | 0.137 |
| CURRENT OPINION IN NEUROLOGY | 1 | 0.137 |
| CURRENT PHARMACEUTICAL DESIGN | 1 | 0.137 |
| CURRENT PROTEIN PEPTIDE SCIENCE | 1 | 0.137 |
| DRUG DEVELOPMENT RESEARCH | 1 | 0.137 |
| DRUGS | 1 | 0.137 |
| ELECTROMAGNETIC BIOLOGY AND MEDICINE | 1 | 0.137 |
| ENDOCRINOLOGY | 1 | 0.137 |
| ERGONOMICS | 1 | 0.137 |
| EUROPEAN JOURNAL OF CLINICAL PHARMACOLOGY | 1 | 0.137 |
| EUROPEAN JOURNAL OF NEUROLOGY | 1 | 0.137 |
| EUROPEAN JOURNAL OF NEUROSCIENCE | 1 | 0.137 |
| EUROPEAN REVIEW FOR MEDICAL AND PHARMACOLOGICAL SCIENCES | 1 | 0.137 |
| EXPERIMENTAL BRAIN RESEARCH | 1 | 0.137 |
| EXPERT OPINION ON BIOLOGICAL THERAPY | 1 | 0.137 |
| EXPLORE THE JOURNAL OF SCIENCE AND HEALING | 1 | 0.137 |
| FRONTIERS IN CELLULAR NEUROSCIENCE | 1 | 0.137 |
| FRONTIERS IN HUMAN NEUROSCIENCE | 1 | 0.137 |
| FRONTIERS IN NEUROLOGY | 1 | 0.137 |
| FRONTIERS IN NEUROSCIENCE | 1 | 0.137 |
| FRONTIERS IN PHARMACOLOGY | 1 | 0.137 |
| GENE THERAPY | 1 | 0.137 |
| INTERNATIONAL JOURNAL OF INDUSTRIAL ERGONOMICS | 1 | 0.137 |
| INTERNATIONAL JOURNAL OF PHARMACOLOGY | 1 | 0.137 |
| JCPSP JOURNAL OF THE COLLEGE OF PHYSICIANS AND SURGEONS PAKISTAN | 1 | 0.137 |
| JOURNAL OF BIOLOGICAL CHEMISTRY | 1 | 0.137 |
| JOURNAL OF BIOMEDICINE AND BIOTECHNOLOGY | 1 | 0.137 |
| JOURNAL OF CHEMICAL NEUROANATOMY | 1 | 0.137 |
| JOURNAL OF CLINICAL ANESTHESIA | 1 | 0.137 |
| JOURNAL OF CLINICAL NEUROSCIENCE | 1 | 0.137 |
| JOURNAL OF COMPARATIVE NEUROLOGY | 1 | 0.137 |
| JOURNAL OF CONTROLLED RELEASE | 1 | 0.137 |
| JOURNAL OF ENDOCRINOLOGICAL INVESTIGATION | 1 | 0.137 |
| JOURNAL OF KOREAN NEUROSURGICAL SOCIETY | 1 | 0.137 |
| JOURNAL OF MOLECULAR NEUROSCIENCE | 1 | 0.137 |
| JOURNAL OF MUSCULOSKELETAL PAIN | 1 | 0.137 |
| JOURNAL OF NEUROENGINEERING AND REHABILITATION | 1 | 0.137 |
| JOURNAL OF NEUROIMMUNE PHARMACOLOGY | 1 | 0.137 |
| JOURNAL OF NEUROIMMUNOLOGY | 1 | 0.137 |
| JOURNAL OF NEUROLOGIC PHYSICAL THERAPY | 1 | 0.137 |
| JOURNAL OF NEUROPHYSIOLOGY | 1 | 0.137 |
| JOURNAL OF NEUROSCIENCE NURSING | 1 | 0.137 |
| JOURNAL OF NEUROSURGICAL SCIENCES | 1 | 0.137 |
| JOURNAL OF NURSING RESEARCH | 1 | 0.137 |
| JOURNAL OF ORTHOPAEDIC RESEARCH | 1 | 0.137 |
| JOURNAL OF PHARMACOLOGICAL SCIENCES | 1 | 0.137 |
| JOURNAL OF PHYSICAL THERAPY SCIENCE | 1 | 0.137 |
| JOURNAL OF TRANSLATIONAL MEDICINE | 1 | 0.137 |
| KOREAN JOURNAL OF PHYSIOLOGY PHARMACOLOGY | 1 | 0.137 |
| MEDICAL HYPOTHESES | 1 | 0.137 |
| MEDICAL SCIENCE MONITOR | 1 | 0.137 |
| MOLECULAR MEDICINE | 1 | 0.137 |
| MOLECULAR MEDICINE REPORTS | 1 | 0.137 |
| MOLECULAR NEUROBIOLOGY | 1 | 0.137 |
| MOLECULES AND CELLS | 1 | 0.137 |
| NEURAL PLASTICITY | 1 | 0.137 |
| NEUROCHEMICAL RESEARCH | 1 | 0.137 |
| NEUROIMAGE CLINICAL | 1 | 0.137 |
| NEUROLOGICAL RESEARCH | 1 | 0.137 |
| NEURON | 1 | 0.137 |
| NEURONS AND NETWORKS IN THE SPINAL CORD | 1 | 0.137 |
| NEUROPEPTIDES | 1 | 0.137 |
| NEUROPHYSIOLOGY | 1 | 0.137 |
| NEUROREHABILITATION | 1 | 0.137 |
| NEUROSIGNALS | 1 | 0.137 |
| NEUROSURGERY | 1 | 0.137 |
| NEUROTHERAPEUTICS | 1 | 0.137 |
| ONCOLOGY | 1 | 0.137 |
| ORTHOPEDICS | 1 | 0.137 |
| PAKISTAN JOURNAL OF MEDICAL SCIENCES | 1 | 0.137 |
| PHYSICAL MEDICINE AND REHABILITATION CLINICS OF NORTH AMERICA | 1 | 0.137 |
| PHYSIOLOGICAL RESEARCH | 1 | 0.137 |
| REGENERATIVE BIOLOGY OF THE SPINE AND SPINAL CORD | 1 | 0.137 |
| SEMINARS IN IMMUNOLOGY | 1 | 0.137 |
| STEREOTACTIC AND FUNCTIONAL NEUROSURGERY | 1 | 0.137 |
| TOXINS | 1 | 0.137 |
| TRANSLATIONAL NEUROSCIENCE | 1 | 0.137 |
| TRENDS IN NEUROSCIENCES | 1 | 0.137 |
| TURKIYE FIZIKSEL TIP VE REHABILITASYON DERGISI TURKISH JOURNAL OF PHYSICAL MEDICINE AND REHABILITATION | 1 | 0.137 |
| SPINAL CORD | 88 | 12.055 |
| PAIN | 60 | 8.219 |
| ARCHIVES OF PHYSICAL MEDICINE AND REHABILITATION | 41 | 5.616 |
| JOURNAL OF NEUROTRAUMA | 33 | 4.521 |
| JOURNAL OF SPINAL CORD MEDICINE | 28 | 3.836 |
| EXPERIMENTAL NEUROLOGY | 22 | 3.014 |
| CLINICAL JOURNAL OF PAIN | 18 | 2.466 |
| JOURNAL OF PAIN | 18 | 2.466 |
| JOURNAL OF REHABILITATION RESEARCH AND DEVELOPMENT | 15 | 2.055 |
| JOURNAL OF NEUROSCIENCE | 14 | 1.918 |
| NEUROSCIENCE LETTERS | 14 | 1.918 |
| DISABILITY AND REHABILITATION | 12 | 1.644 |
| EUROPEAN JOURNAL OF PAIN | 11 | 1.507 |
| JOURNAL OF REHABILITATION MEDICINE | 11 | 1.507 |
| JOURNAL OF PAIN RESEARCH | 9 | 1.233 |
| SPINE | 9 | 1.233 |
| AMERICAN JOURNAL OF PHYSICAL MEDICINE REHABILITATION | 7 | 0.959 |
| NEUROLOGY | 7 | 0.959 |
| PM R | 7 | 0.959 |
| MOLECULAR PAIN | 6 | 0.822 |
| NEUROREHABILITATION AND NEURAL REPAIR | 6 | 0.822 |
| NEUROSCIENCE | 6 | 0.822 |
| PAIN PHYSICIAN | 6 | 0.822 |
| ACTA NEUROCHIRURGICA | 5 | 0.685 |
| ANESTHESIA AND ANALGESIA | 5 | 0.685 |
| PARAPLEGIA | 5 | 0.685 |
| PLOS ONE | 5 | 0.685 |
| BRAIN | 4 | 0.548 |
| BRAIN RESEARCH | 4 | 0.548 |
| BRAIN RESEARCH BULLETIN | 4 | 0.548 |
| GLIA | 4 | 0.548 |
| JOURNAL OF NEUROSURGERY | 4 | 0.548 |
| JOURNAL OF NEUROSURGERY SPINE | 4 | 0.548 |
| SCIENTIFIC REPORTS | 4 | 0.548 |
| CLINICAL NEUROPHYSIOLOGY | 3 | 0.411 |
| CLINICAL REHABILITATION | 3 | 0.411 |
| EUROPEAN JOURNAL OF PHARMACOLOGY | 3 | 0.411 |
| FRONTIERS IN PHYSIOLOGY | 3 | 0.411 |
| JOURNAL OF BACK AND MUSCULOSKELETAL REHABILITATION | 3 | 0.411 |
| JOURNAL OF NEUROCHEMISTRY | 3 | 0.411 |
| JOURNAL OF NEUROINFLAMMATION | 3 | 0.411 |
| JOURNAL OF PHARMACOLOGY AND EXPERIMENTAL THERAPEUTICS | 3 | 0.411 |
| MEDICINE | 3 | 0.411 |
| NEURAL REGENERATION RESEARCH | 3 | 0.411 |
| PAIN MEDICINE | 3 | 0.411 |
| PHYSICAL THERAPY | 3 | 0.411 |
| PROCEEDINGS OF THE NATIONAL ACADEMY OF SCIENCES OF THE UNITED STATES OF AMERICA | 3 | 0.411 |
| SOMATOSENSORY AND MOTOR RESEARCH | 3 | 0.411 |
| SPINE JOURNAL | 3 | 0.411 |
| WORLD NEUROSURGERY | 3 | 0.411 |
| ACTA MEDICA MEDITERRANEA | 2 | 0.274 |
| ANNALS OF NEUROLOGY | 2 | 0.274 |
| ANNALS OF PHYSICAL AND REHABILITATION MEDICINE | 2 | 0.274 |
| ARQUIVOS DE NEURO PSIQUIATRIA | 2 | 0.274 |
| BMC NEUROLOGY | 2 | 0.274 |
| BRAIN RESEARCH REVIEWS | 2 | 0.274 |
| BRITISH JOURNAL OF PHARMACOLOGY | 2 | 0.274 |
| CELL DEATH DISEASE | 2 | 0.274 |
| CLINICAL ORTHOPAEDICS AND RELATED RESEARCH | 2 | 0.274 |
| COMPLEMENTARY THERAPIES IN MEDICINE | 2 | 0.274 |
| CURRENT PAIN AND HEADACHE REPORTS | 2 | 0.274 |
| EUROPEAN JOURNAL OF PHYSICAL AND REHABILITATION MEDICINE | 2 | 0.274 |
| EXPERIMENTAL AND THERAPEUTIC MEDICINE | 2 | 0.274 |
| FRONTIERS IN SYSTEMS NEUROSCIENCE | 2 | 0.274 |
| INTERNATIONAL JOURNAL OF REHABILITATION RESEARCH | 2 | 0.274 |
| JOURNAL OF NEUROLOGY NEUROSURGERY AND PSYCHIATRY | 2 | 0.274 |
| JOURNAL OF NEUROSCIENCE RESEARCH | 2 | 0.274 |
| JOURNAL OF ORTHOPAEDIC SURGERY AND RESEARCH | 2 | 0.274 |
| JOURNAL OF PAIN AND SYMPTOM MANAGEMENT | 2 | 0.274 |
| METABOLIC BRAIN DISEASE | 2 | 0.274 |
| MOLECULAR BRAIN RESEARCH | 2 | 0.274 |
| MOLECULAR THERAPY | 2 | 0.274 |
| NEUROBIOLOGY OF DISEASE | 2 | 0.274 |
| NEUROIMAGE | 2 | 0.274 |
| NEUROMODULATION | 2 | 0.274 |
| NEUROPHARMACOLOGY | 2 | 0.274 |
| NEUROSCIENCE BULLETIN | 2 | 0.274 |
| NEUROSCIENCE RESEARCH | 2 | 0.274 |
| NEUROSCIENCES | 2 | 0.274 |
| PAIN CLINIC | 2 | 0.274 |
| PAIN PRACTICE | 2 | 0.274 |
| PHARMACOLOGY BIOCHEMISTRY AND BEHAVIOR | 2 | 0.274 |
| QUALITY OF LIFE RESEARCH | 2 | 0.274 |
| REGIONAL ANESTHESIA AND PAIN MEDICINE | 2 | 0.274 |
| RESTORATIVE NEUROLOGY AND NEUROSCIENCE | 2 | 0.274 |
| STEM CELL RESEARCH THERAPY | 2 | 0.274 |
| STEM CELLS | 2 | 0.274 |
| ACTA ANAESTHESIOLOGICA SCANDINAVICA | 1 | 0.137 |
| ACTA BIOCHIMICA ET BIOPHYSICA SINICA | 1 | 0.137 |
| ACTA PHARMACOLOGICA SINICA | 1 | 0.137 |
| ACUPUNCTURE IN MEDICINE | 1 | 0.137 |
| ADVANCES IN EXPERIMENTAL MEDICINE AND BIOLOGY | 1 | 0.137 |
| ADVANCES IN PAIN RESEARCH AND THERAPY | 1 | 0.137 |
| AMERICAN JOURNAL OF NEURORADIOLOGY | 1 | 0.137 |
| AMERICAN JOURNAL OF SURGERY | 1 | 0.137 |
| ANAESTHESIA AND INTENSIVE CARE | 1 | 0.137 |
| ANESTHESIOLOGY | 1 | 0.137 |
| ANKARA UNIVERSITESI VETERINER FAKULTESI DERGISI | 1 | 0.137 |
| ANNALS OF SAUDI MEDICINE | 1 | 0.137 |
| ANNALS OF THE NEW YORK ACADEMY OF SCIENCES | 1 | 0.137 |
| AUSTRALIAN OCCUPATIONAL THERAPY JOURNAL | 1 | 0.137 |
| AUTONOMIC NEUROSCIENCE BASIC CLINICAL | 1 | 0.137 |
| BEHAVIOURAL BRAIN RESEARCH | 1 | 0.137 |
| BEHAVIOURAL PHARMACOLOGY | 1 | 0.137 |
| BIOCHIMICA ET BIOPHYSICA ACTA MOLECULAR BASIS OF DISEASE | 1 | 0.137 |
| BIOLOGICAL RESEARCH FOR NURSING | 1 | 0.137 |
| BIOMED RESEARCH INTERNATIONAL | 1 | 0.137 |
| BIOMEDICAL RESEARCH INDIA | 1 | 0.137 |
| BMJ OPEN | 1 | 0.137 |
| BRAIN BEHAVIOR AND IMMUNITY | 1 | 0.137 |
| CANADIAN JOURNAL OF PHYSIOLOGY AND PHARMACOLOGY | 1 | 0.137 |
| CANADIAN MEDICAL ASSOCIATION JOURNAL | 1 | 0.137 |
| CARDIOLOGY IN THE YOUNG | 1 | 0.137 |
| CELL STEM CELL | 1 | 0.137 |
| CELL TRANSPLANTATION | 1 | 0.137 |
| CEREBRAL CORTEX | 1 | 0.137 |
| CHINESE MEDICAL JOURNAL | 1 | 0.137 |
| CLINICAL NEUROLOGY AND NEUROSURGERY | 1 | 0.137 |
| CLINICAL PHARMACOLOGY IN DRUG DEVELOPMENT | 1 | 0.137 |
| CLINICAL THERAPEUTICS | 1 | 0.137 |
| CNS DRUGS | 1 | 0.137 |
| COCHRANE DATABASE OF SYSTEMATIC REVIEWS | 1 | 0.137 |
| COMPUTER METHODS AND PROGRAMS IN BIOMEDICINE | 1 | 0.137 |
| CURRENT OPINION IN NEUROLOGY | 1 | 0.137 |
| CURRENT PHARMACEUTICAL DESIGN | 1 | 0.137 |
| CURRENT PROTEIN PEPTIDE SCIENCE | 1 | 0.137 |
| DRUG DEVELOPMENT RESEARCH | 1 | 0.137 |
| DRUGS | 1 | 0.137 |
| ELECTROMAGNETIC BIOLOGY AND MEDICINE | 1 | 0.137 |
| ENDOCRINOLOGY | 1 | 0.137 |
| ERGONOMICS | 1 | 0.137 |
| EUROPEAN JOURNAL OF CLINICAL PHARMACOLOGY | 1 | 0.137 |
| EUROPEAN JOURNAL OF NEUROLOGY | 1 | 0.137 |
| EUROPEAN JOURNAL OF NEUROSCIENCE | 1 | 0.137 |
| EUROPEAN REVIEW FOR MEDICAL AND PHARMACOLOGICAL SCIENCES | 1 | 0.137 |
| EXPERIMENTAL BRAIN RESEARCH | 1 | 0.137 |
| EXPERT OPINION ON BIOLOGICAL THERAPY | 1 | 0.137 |
| EXPLORE THE JOURNAL OF SCIENCE AND HEALING | 1 | 0.137 |
| FRONTIERS IN CELLULAR NEUROSCIENCE | 1 | 0.137 |
| FRONTIERS IN HUMAN NEUROSCIENCE | 1 | 0.137 |
| FRONTIERS IN NEUROLOGY | 1 | 0.137 |
| FRONTIERS IN NEUROSCIENCE | 1 | 0.137 |
| FRONTIERS IN PHARMACOLOGY | 1 | 0.137 |
| GENE THERAPY | 1 | 0.137 |
| INTERNATIONAL JOURNAL OF INDUSTRIAL ERGONOMICS | 1 | 0.137 |
| INTERNATIONAL JOURNAL OF PHARMACOLOGY | 1 | 0.137 |
| JCPSP JOURNAL OF THE COLLEGE OF PHYSICIANS AND SURGEONS PAKISTAN | 1 | 0.137 |
| JOURNAL OF BIOLOGICAL CHEMISTRY | 1 | 0.137 |
| JOURNAL OF BIOMEDICINE AND BIOTECHNOLOGY | 1 | 0.137 |
| JOURNAL OF CHEMICAL NEUROANATOMY | 1 | 0.137 |
| JOURNAL OF CLINICAL ANESTHESIA | 1 | 0.137 |
| JOURNAL OF CLINICAL NEUROSCIENCE | 1 | 0.137 |
| JOURNAL OF COMPARATIVE NEUROLOGY | 1 | 0.137 |
| JOURNAL OF CONTROLLED RELEASE | 1 | 0.137 |
| JOURNAL OF ENDOCRINOLOGICAL INVESTIGATION | 1 | 0.137 |
| JOURNAL OF KOREAN NEUROSURGICAL SOCIETY | 1 | 0.137 |
| JOURNAL OF MOLECULAR NEUROSCIENCE | 1 | 0.137 |
| JOURNAL OF MUSCULOSKELETAL PAIN | 1 | 0.137 |
| JOURNAL OF NEUROENGINEERING AND REHABILITATION | 1 | 0.137 |
| JOURNAL OF NEUROIMMUNE PHARMACOLOGY | 1 | 0.137 |
| JOURNAL OF NEUROIMMUNOLOGY | 1 | 0.137 |
| JOURNAL OF NEUROLOGIC PHYSICAL THERAPY | 1 | 0.137 |
| JOURNAL OF NEUROPHYSIOLOGY | 1 | 0.137 |
| JOURNAL OF NEUROSCIENCE NURSING | 1 | 0.137 |
| JOURNAL OF NEUROSURGICAL SCIENCES | 1 | 0.137 |
| JOURNAL OF NURSING RESEARCH | 1 | 0.137 |
| JOURNAL OF ORTHOPAEDIC RESEARCH | 1 | 0.137 |
| JOURNAL OF PHARMACOLOGICAL SCIENCES | 1 | 0.137 |
| JOURNAL OF PHYSICAL THERAPY SCIENCE | 1 | 0.137 |
| JOURNAL OF TRANSLATIONAL MEDICINE | 1 | 0.137 |
| KOREAN JOURNAL OF PHYSIOLOGY PHARMACOLOGY | 1 | 0.137 |
| MEDICAL HYPOTHESES | 1 | 0.137 |
| MEDICAL SCIENCE MONITOR | 1 | 0.137 |
| MOLECULAR MEDICINE | 1 | 0.137 |
| MOLECULAR MEDICINE REPORTS | 1 | 0.137 |
| MOLECULAR NEUROBIOLOGY | 1 | 0.137 |
| MOLECULES AND CELLS | 1 | 0.137 |
| NEURAL PLASTICITY | 1 | 0.137 |
| NEUROCHEMICAL RESEARCH | 1 | 0.137 |
| NEUROIMAGE CLINICAL | 1 | 0.137 |
| NEUROLOGICAL RESEARCH | 1 | 0.137 |
| NEURON | 1 | 0.137 |
| NEURONS AND NETWORKS IN THE SPINAL CORD | 1 | 0.137 |
| NEUROPEPTIDES | 1 | 0.137 |
| NEUROPHYSIOLOGY | 1 | 0.137 |
| NEUROREHABILITATION | 1 | 0.137 |
| NEUROSIGNALS | 1 | 0.137 |
| NEUROSURGERY | 1 | 0.137 |
| NEUROTHERAPEUTICS | 1 | 0.137 |
| ONCOLOGY | 1 | 0.137 |
| ORTHOPEDICS | 1 | 0.137 |
| PAKISTAN JOURNAL OF MEDICAL SCIENCES | 1 | 0.137 |
| PHYSICAL MEDICINE AND REHABILITATION CLINICS OF NORTH AMERICA | 1 | 0.137 |
| PHYSIOLOGICAL RESEARCH | 1 | 0.137 |
| REGENERATIVE BIOLOGY OF THE SPINE AND SPINAL CORD | 1 | 0.137 |
| SEMINARS IN IMMUNOLOGY | 1 | 0.137 |
| STEREOTACTIC AND FUNCTIONAL NEUROSURGERY | 1 | 0.137 |
| TOXINS | 1 | 0.137 |
| TRANSLATIONAL NEUROSCIENCE | 1 | 0.137 |
| TRENDS IN NEUROSCIENCES | 1 | 0.137 |
| TURKIYE FIZIKSEL TIP VE REHABILITASYON DERGISI TURKISH JOURNAL OF PHYSICAL MEDICINE AND REHABILITATION | 1 | 0.137 |
| SPINAL CORD | 88 | 12.055 |
| PAIN | 60 | 8.219 |
| ARCHIVES OF PHYSICAL MEDICINE AND REHABILITATION | 41 | 5.616 |
| JOURNAL OF NEUROTRAUMA | 33 | 4.521 |
| JOURNAL OF SPINAL CORD MEDICINE | 28 | 3.836 |
| EXPERIMENTAL NEUROLOGY | 22 | 3.014 |
| CLINICAL JOURNAL OF PAIN | 18 | 2.466 |
| JOURNAL OF PAIN | 18 | 2.466 |
| JOURNAL OF REHABILITATION RESEARCH AND DEVELOPMENT | 15 | 2.055 |
| JOURNAL OF NEUROSCIENCE | 14 | 1.918 |
| NEUROSCIENCE LETTERS | 14 | 1.918 |
| DISABILITY AND REHABILITATION | 12 | 1.644 |
| EUROPEAN JOURNAL OF PAIN | 11 | 1.507 |
| JOURNAL OF REHABILITATION MEDICINE | 11 | 1.507 |
| JOURNAL OF PAIN RESEARCH | 9 | 1.233 |
| SPINE | 9 | 1.233 |
| AMERICAN JOURNAL OF PHYSICAL MEDICINE REHABILITATION | 7 | 0.959 |
| NEUROLOGY | 7 | 0.959 |
| PM R | 7 | 0.959 |
| MOLECULAR PAIN | 6 | 0.822 |
| NEUROREHABILITATION AND NEURAL REPAIR | 6 | 0.822 |
| NEUROSCIENCE | 6 | 0.822 |
| PAIN PHYSICIAN | 6 | 0.822 |
| ACTA NEUROCHIRURGICA | 5 | 0.685 |
| ANESTHESIA AND ANALGESIA | 5 | 0.685 |
| PARAPLEGIA | 5 | 0.685 |
| PLOS ONE | 5 | 0.685 |
| BRAIN | 4 | 0.548 |
| BRAIN RESEARCH | 4 | 0.548 |
| BRAIN RESEARCH BULLETIN | 4 | 0.548 |
| GLIA | 4 | 0.548 |
| JOURNAL OF NEUROSURGERY | 4 | 0.548 |
| JOURNAL OF NEUROSURGERY SPINE | 4 | 0.548 |
| SCIENTIFIC REPORTS | 4 | 0.548 |
| CLINICAL NEUROPHYSIOLOGY | 3 | 0.411 |
| CLINICAL REHABILITATION | 3 | 0.411 |
| EUROPEAN JOURNAL OF PHARMACOLOGY | 3 | 0.411 |
| FRONTIERS IN PHYSIOLOGY | 3 | 0.411 |
| JOURNAL OF BACK AND MUSCULOSKELETAL REHABILITATION | 3 | 0.411 |
| JOURNAL OF NEUROCHEMISTRY | 3 | 0.411 |
| JOURNAL OF NEUROINFLAMMATION | 3 | 0.411 |
| JOURNAL OF PHARMACOLOGY AND EXPERIMENTAL THERAPEUTICS | 3 | 0.411 |
| MEDICINE | 3 | 0.411 |
| NEURAL REGENERATION RESEARCH | 3 | 0.411 |
| PAIN MEDICINE | 3 | 0.411 |
| PHYSICAL THERAPY | 3 | 0.411 |
| PROCEEDINGS OF THE NATIONAL ACADEMY OF SCIENCES OF THE UNITED STATES OF AMERICA | 3 | 0.411 |
| SOMATOSENSORY AND MOTOR RESEARCH | 3 | 0.411 |
| SPINE JOURNAL | 3 | 0.411 |
| WORLD NEUROSURGERY | 3 | 0.411 |
| ACTA MEDICA MEDITERRANEA | 2 | 0.274 |
| ANNALS OF NEUROLOGY | 2 | 0.274 |
| ANNALS OF PHYSICAL AND REHABILITATION MEDICINE | 2 | 0.274 |
| ARQUIVOS DE NEURO PSIQUIATRIA | 2 | 0.274 |
| BMC NEUROLOGY | 2 | 0.274 |
| BRAIN RESEARCH REVIEWS | 2 | 0.274 |
| BRITISH JOURNAL OF PHARMACOLOGY | 2 | 0.274 |
| CELL DEATH DISEASE | 2 | 0.274 |
| CLINICAL ORTHOPAEDICS AND RELATED RESEARCH | 2 | 0.274 |
| COMPLEMENTARY THERAPIES IN MEDICINE | 2 | 0.274 |
| CURRENT PAIN AND HEADACHE REPORTS | 2 | 0.274 |
| EUROPEAN JOURNAL OF PHYSICAL AND REHABILITATION MEDICINE | 2 | 0.274 |
| EXPERIMENTAL AND THERAPEUTIC MEDICINE | 2 | 0.274 |
| FRONTIERS IN SYSTEMS NEUROSCIENCE | 2 | 0.274 |
| INTERNATIONAL JOURNAL OF REHABILITATION RESEARCH | 2 | 0.274 |
| JOURNAL OF NEUROLOGY NEUROSURGERY AND PSYCHIATRY | 2 | 0.274 |
| JOURNAL OF NEUROSCIENCE RESEARCH | 2 | 0.274 |
| JOURNAL OF ORTHOPAEDIC SURGERY AND RESEARCH | 2 | 0.274 |
| JOURNAL OF PAIN AND SYMPTOM MANAGEMENT | 2 | 0.274 |
| METABOLIC BRAIN DISEASE | 2 | 0.274 |
| MOLECULAR BRAIN RESEARCH | 2 | 0.274 |
| MOLECULAR THERAPY | 2 | 0.274 |
| NEUROBIOLOGY OF DISEASE | 2 | 0.274 |
| NEUROIMAGE | 2 | 0.274 |
| NEUROMODULATION | 2 | 0.274 |
| NEUROPHARMACOLOGY | 2 | 0.274 |
| NEUROSCIENCE BULLETIN | 2 | 0.274 |
| NEUROSCIENCE RESEARCH | 2 | 0.274 |
| NEUROSCIENCES | 2 | 0.274 |
| PAIN CLINIC | 2 | 0.274 |
| PAIN PRACTICE | 2 | 0.274 |
| PHARMACOLOGY BIOCHEMISTRY AND BEHAVIOR | 2 | 0.274 |
| QUALITY OF LIFE RESEARCH | 2 | 0.274 |
| REGIONAL ANESTHESIA AND PAIN MEDICINE | 2 | 0.274 |
| RESTORATIVE NEUROLOGY AND NEUROSCIENCE | 2 | 0.274 |
| STEM CELL RESEARCH THERAPY | 2 | 0.274 |
| STEM CELLS | 2 | 0.274 |
| ACTA ANAESTHESIOLOGICA SCANDINAVICA | 1 | 0.137 |
| ACTA BIOCHIMICA ET BIOPHYSICA SINICA | 1 | 0.137 |
| ACTA PHARMACOLOGICA SINICA | 1 | 0.137 |
| ACUPUNCTURE IN MEDICINE | 1 | 0.137 |
| ADVANCES IN EXPERIMENTAL MEDICINE AND BIOLOGY | 1 | 0.137 |
| ADVANCES IN PAIN RESEARCH AND THERAPY | 1 | 0.137 |
| AMERICAN JOURNAL OF NEURORADIOLOGY | 1 | 0.137 |
| AMERICAN JOURNAL OF SURGERY | 1 | 0.137 |
| ANAESTHESIA AND INTENSIVE CARE | 1 | 0.137 |
| ANESTHESIOLOGY | 1 | 0.137 |
| ANKARA UNIVERSITESI VETERINER FAKULTESI DERGISI | 1 | 0.137 |
| ANNALS OF SAUDI MEDICINE | 1 | 0.137 |
| ANNALS OF THE NEW YORK ACADEMY OF SCIENCES | 1 | 0.137 |
| AUSTRALIAN OCCUPATIONAL THERAPY JOURNAL | 1 | 0.137 |
| AUTONOMIC NEUROSCIENCE BASIC CLINICAL | 1 | 0.137 |
| BEHAVIOURAL BRAIN RESEARCH | 1 | 0.137 |
| BEHAVIOURAL PHARMACOLOGY | 1 | 0.137 |
| BIOCHIMICA ET BIOPHYSICA ACTA MOLECULAR BASIS OF DISEASE | 1 | 0.137 |
| BIOLOGICAL RESEARCH FOR NURSING | 1 | 0.137 |
| BIOMED RESEARCH INTERNATIONAL | 1 | 0.137 |
| BIOMEDICAL RESEARCH INDIA | 1 | 0.137 |
| BMJ OPEN | 1 | 0.137 |
| BRAIN BEHAVIOR AND IMMUNITY | 1 | 0.137 |
| CANADIAN JOURNAL OF PHYSIOLOGY AND PHARMACOLOGY | 1 | 0.137 |
| CANADIAN MEDICAL ASSOCIATION JOURNAL | 1 | 0.137 |
| CARDIOLOGY IN THE YOUNG | 1 | 0.137 |
| CELL STEM CELL | 1 | 0.137 |
| CELL TRANSPLANTATION | 1 | 0.137 |
| CEREBRAL CORTEX | 1 | 0.137 |
| CHINESE MEDICAL JOURNAL | 1 | 0.137 |
| CLINICAL NEUROLOGY AND NEUROSURGERY | 1 | 0.137 |
| CLINICAL PHARMACOLOGY IN DRUG DEVELOPMENT | 1 | 0.137 |
| CLINICAL THERAPEUTICS | 1 | 0.137 |
| CNS DRUGS | 1 | 0.137 |
| COCHRANE DATABASE OF SYSTEMATIC REVIEWS | 1 | 0.137 |
| COMPUTER METHODS AND PROGRAMS IN BIOMEDICINE | 1 | 0.137 |
| CURRENT OPINION IN NEUROLOGY | 1 | 0.137 |
| CURRENT PHARMACEUTICAL DESIGN | 1 | 0.137 |
| CURRENT PROTEIN PEPTIDE SCIENCE | 1 | 0.137 |
| DRUG DEVELOPMENT RESEARCH | 1 | 0.137 |
| DRUGS | 1 | 0.137 |
| ELECTROMAGNETIC BIOLOGY AND MEDICINE | 1 | 0.137 |
| ENDOCRINOLOGY | 1 | 0.137 |
| ERGONOMICS | 1 | 0.137 |
| EUROPEAN JOURNAL OF CLINICAL PHARMACOLOGY | 1 | 0.137 |
| EUROPEAN JOURNAL OF NEUROLOGY | 1 | 0.137 |
| EUROPEAN JOURNAL OF NEUROSCIENCE | 1 | 0.137 |
| EUROPEAN REVIEW FOR MEDICAL AND PHARMACOLOGICAL SCIENCES | 1 | 0.137 |
| EXPERIMENTAL BRAIN RESEARCH | 1 | 0.137 |
| EXPERT OPINION ON BIOLOGICAL THERAPY | 1 | 0.137 |
| EXPLORE THE JOURNAL OF SCIENCE AND HEALING | 1 | 0.137 |
| FRONTIERS IN CELLULAR NEUROSCIENCE | 1 | 0.137 |
| FRONTIERS IN HUMAN NEUROSCIENCE | 1 | 0.137 |
| FRONTIERS IN NEUROLOGY | 1 | 0.137 |
| FRONTIERS IN NEUROSCIENCE | 1 | 0.137 |
| FRONTIERS IN PHARMACOLOGY | 1 | 0.137 |
| GENE THERAPY | 1 | 0.137 |
| INTERNATIONAL JOURNAL OF INDUSTRIAL ERGONOMICS | 1 | 0.137 |
| INTERNATIONAL JOURNAL OF PHARMACOLOGY | 1 | 0.137 |
| JCPSP JOURNAL OF THE COLLEGE OF PHYSICIANS AND SURGEONS PAKISTAN | 1 | 0.137 |
| JOURNAL OF BIOLOGICAL CHEMISTRY | 1 | 0.137 |
| JOURNAL OF BIOMEDICINE AND BIOTECHNOLOGY | 1 | 0.137 |
| JOURNAL OF CHEMICAL NEUROANATOMY | 1 | 0.137 |
| JOURNAL OF CLINICAL ANESTHESIA | 1 | 0.137 |
| JOURNAL OF CLINICAL NEUROSCIENCE | 1 | 0.137 |
| JOURNAL OF COMPARATIVE NEUROLOGY | 1 | 0.137 |
| JOURNAL OF CONTROLLED RELEASE | 1 | 0.137 |
| JOURNAL OF ENDOCRINOLOGICAL INVESTIGATION | 1 | 0.137 |
| JOURNAL OF KOREAN NEUROSURGICAL SOCIETY | 1 | 0.137 |
| JOURNAL OF MOLECULAR NEUROSCIENCE | 1 | 0.137 |
| JOURNAL OF MUSCULOSKELETAL PAIN | 1 | 0.137 |
| JOURNAL OF NEUROENGINEERING AND REHABILITATION | 1 | 0.137 |
| JOURNAL OF NEUROIMMUNE PHARMACOLOGY | 1 | 0.137 |
| JOURNAL OF NEUROIMMUNOLOGY | 1 | 0.137 |
| JOURNAL OF NEUROLOGIC PHYSICAL THERAPY | 1 | 0.137 |
| JOURNAL OF NEUROPHYSIOLOGY | 1 | 0.137 |
| JOURNAL OF NEUROSCIENCE NURSING | 1 | 0.137 |
| JOURNAL OF NEUROSURGICAL SCIENCES | 1 | 0.137 |
| JOURNAL OF NURSING RESEARCH | 1 | 0.137 |
| JOURNAL OF ORTHOPAEDIC RESEARCH | 1 | 0.137 |
| JOURNAL OF PHARMACOLOGICAL SCIENCES | 1 | 0.137 |
| JOURNAL OF PHYSICAL THERAPY SCIENCE | 1 | 0.137 |
| JOURNAL OF TRANSLATIONAL MEDICINE | 1 | 0.137 |
| KOREAN JOURNAL OF PHYSIOLOGY PHARMACOLOGY | 1 | 0.137 |
| MEDICAL HYPOTHESES | 1 | 0.137 |
| MEDICAL SCIENCE MONITOR | 1 | 0.137 |
| MOLECULAR MEDICINE | 1 | 0.137 |
| MOLECULAR MEDICINE REPORTS | 1 | 0.137 |
| MOLECULAR NEUROBIOLOGY | 1 | 0.137 |
| MOLECULES AND CELLS | 1 | 0.137 |
| NEURAL PLASTICITY | 1 | 0.137 |
| NEUROCHEMICAL RESEARCH | 1 | 0.137 |
| NEUROIMAGE CLINICAL | 1 | 0.137 |
| NEUROLOGICAL RESEARCH | 1 | 0.137 |
| NEURON | 1 | 0.137 |
| NEURONS AND NETWORKS IN THE SPINAL CORD | 1 | 0.137 |
| NEUROPEPTIDES | 1 | 0.137 |
| NEUROPHYSIOLOGY | 1 | 0.137 |
| NEUROREHABILITATION | 1 | 0.137 |
| NEUROSIGNALS | 1 | 0.137 |
| NEUROSURGERY | 1 | 0.137 |
| NEUROTHERAPEUTICS | 1 | 0.137 |
| ONCOLOGY | 1 | 0.137 |
| ORTHOPEDICS | 1 | 0.137 |
| PAKISTAN JOURNAL OF MEDICAL SCIENCES | 1 | 0.137 |
| PHYSICAL MEDICINE AND REHABILITATION CLINICS OF NORTH AMERICA | 1 | 0.137 |
| PHYSIOLOGICAL RESEARCH | 1 | 0.137 |
| REGENERATIVE BIOLOGY OF THE SPINE AND SPINAL CORD | 1 | 0.137 |
| SEMINARS IN IMMUNOLOGY | 1 | 0.137 |
| STEREOTACTIC AND FUNCTIONAL NEUROSURGERY | 1 | 0.137 |
| TOXINS | 1 | 0.137 |
| TRANSLATIONAL NEUROSCIENCE | 1 | 0.137 |
| TRENDS IN NEUROSCIENCES | 1 | 0.137 |
| TURKIYE FIZIKSEL TIP VE REHABILITASYON DERGISI TURKISH JOURNAL OF PHYSICAL MEDICINE AND REHABILITATION | 1 | 0.137 |
| SPINAL CORD | 88 | 12.055 |
| PAIN | 60 | 8.219 |
| ARCHIVES OF PHYSICAL MEDICINE AND REHABILITATION | 41 | 5.616 |
| JOURNAL OF NEUROTRAUMA | 33 | 4.521 |
| JOURNAL OF SPINAL CORD MEDICINE | 28 | 3.836 |
| EXPERIMENTAL NEUROLOGY | 22 | 3.014 |
| CLINICAL JOURNAL OF PAIN | 18 | 2.466 |
| JOURNAL OF PAIN | 18 | 2.466 |
| JOURNAL OF REHABILITATION RESEARCH AND DEVELOPMENT | 15 | 2.055 |
| JOURNAL OF NEUROSCIENCE | 14 | 1.918 |
| NEUROSCIENCE LETTERS | 14 | 1.918 |
| DISABILITY AND REHABILITATION | 12 | 1.644 |
| EUROPEAN JOURNAL OF PAIN | 11 | 1.507 |
| JOURNAL OF REHABILITATION MEDICINE | 11 | 1.507 |
| JOURNAL OF PAIN RESEARCH | 9 | 1.233 |
| SPINE | 9 | 1.233 |
| AMERICAN JOURNAL OF PHYSICAL MEDICINE REHABILITATION | 7 | 0.959 |
| NEUROLOGY | 7 | 0.959 |
| PM R | 7 | 0.959 |
| MOLECULAR PAIN | 6 | 0.822 |
| NEUROREHABILITATION AND NEURAL REPAIR | 6 | 0.822 |
| NEUROSCIENCE | 6 | 0.822 |
| PAIN PHYSICIAN | 6 | 0.822 |
| ACTA NEUROCHIRURGICA | 5 | 0.685 |
| ANESTHESIA AND ANALGESIA | 5 | 0.685 |
| PARAPLEGIA | 5 | 0.685 |
| PLOS ONE | 5 | 0.685 |
| BRAIN | 4 | 0.548 |
| BRAIN RESEARCH | 4 | 0.548 |
| BRAIN RESEARCH BULLETIN | 4 | 0.548 |
| GLIA | 4 | 0.548 |
| JOURNAL OF NEUROSURGERY | 4 | 0.548 |
| JOURNAL OF NEUROSURGERY SPINE | 4 | 0.548 |
| SCIENTIFIC REPORTS | 4 | 0.548 |
| CLINICAL NEUROPHYSIOLOGY | 3 | 0.411 |
| CLINICAL REHABILITATION | 3 | 0.411 |
| EUROPEAN JOURNAL OF PHARMACOLOGY | 3 | 0.411 |
| FRONTIERS IN PHYSIOLOGY | 3 | 0.411 |
| JOURNAL OF BACK AND MUSCULOSKELETAL REHABILITATION | 3 | 0.411 |
| JOURNAL OF NEUROCHEMISTRY | 3 | 0.411 |
| JOURNAL OF NEUROINFLAMMATION | 3 | 0.411 |
| JOURNAL OF PHARMACOLOGY AND EXPERIMENTAL THERAPEUTICS | 3 | 0.411 |
| MEDICINE | 3 | 0.411 |
| NEURAL REGENERATION RESEARCH | 3 | 0.411 |
| PAIN MEDICINE | 3 | 0.411 |
| PHYSICAL THERAPY | 3 | 0.411 |
| PROCEEDINGS OF THE NATIONAL ACADEMY OF SCIENCES OF THE UNITED STATES OF AMERICA | 3 | 0.411 |
| SOMATOSENSORY AND MOTOR RESEARCH | 3 | 0.411 |
| SPINE JOURNAL | 3 | 0.411 |
| WORLD NEUROSURGERY | 3 | 0.411 |
| ACTA MEDICA MEDITERRANEA | 2 | 0.274 |
| ANNALS OF NEUROLOGY | 2 | 0.274 |
| ANNALS OF PHYSICAL AND REHABILITATION MEDICINE | 2 | 0.274 |
| ARQUIVOS DE NEURO PSIQUIATRIA | 2 | 0.274 |
| BMC NEUROLOGY | 2 | 0.274 |
| BRAIN RESEARCH REVIEWS | 2 | 0.274 |
| BRITISH JOURNAL OF PHARMACOLOGY | 2 | 0.274 |
| CELL DEATH DISEASE | 2 | 0.274 |
| CLINICAL ORTHOPAEDICS AND RELATED RESEARCH | 2 | 0.274 |
| COMPLEMENTARY THERAPIES IN MEDICINE | 2 | 0.274 |
| CURRENT PAIN AND HEADACHE REPORTS | 2 | 0.274 |
| EUROPEAN JOURNAL OF PHYSICAL AND REHABILITATION MEDICINE | 2 | 0.274 |
| EXPERIMENTAL AND THERAPEUTIC MEDICINE | 2 | 0.274 |
| FRONTIERS IN SYSTEMS NEUROSCIENCE | 2 | 0.274 |
| INTERNATIONAL JOURNAL OF REHABILITATION RESEARCH | 2 | 0.274 |
| JOURNAL OF NEUROLOGY NEUROSURGERY AND PSYCHIATRY | 2 | 0.274 |
| JOURNAL OF NEUROSCIENCE RESEARCH | 2 | 0.274 |
| JOURNAL OF ORTHOPAEDIC SURGERY AND RESEARCH | 2 | 0.274 |
| JOURNAL OF PAIN AND SYMPTOM MANAGEMENT | 2 | 0.274 |
| METABOLIC BRAIN DISEASE | 2 | 0.274 |
| MOLECULAR BRAIN RESEARCH | 2 | 0.274 |
| MOLECULAR THERAPY | 2 | 0.274 |
| NEUROBIOLOGY OF DISEASE | 2 | 0.274 |
| NEUROIMAGE | 2 | 0.274 |
| NEUROMODULATION | 2 | 0.274 |
| NEUROPHARMACOLOGY | 2 | 0.274 |
| NEUROSCIENCE BULLETIN | 2 | 0.274 |
| NEUROSCIENCE RESEARCH | 2 | 0.274 |
| NEUROSCIENCES | 2 | 0.274 |
| PAIN CLINIC | 2 | 0.274 |
| PAIN PRACTICE | 2 | 0.274 |
| PHARMACOLOGY BIOCHEMISTRY AND BEHAVIOR | 2 | 0.274 |
| QUALITY OF LIFE RESEARCH | 2 | 0.274 |
| REGIONAL ANESTHESIA AND PAIN MEDICINE | 2 | 0.274 |
| RESTORATIVE NEUROLOGY AND NEUROSCIENCE | 2 | 0.274 |
| STEM CELL RESEARCH THERAPY | 2 | 0.274 |
| STEM CELLS | 2 | 0.274 |
| ACTA ANAESTHESIOLOGICA SCANDINAVICA | 1 | 0.137 |
| ACTA BIOCHIMICA ET BIOPHYSICA SINICA | 1 | 0.137 |
| ACTA PHARMACOLOGICA SINICA | 1 | 0.137 |
| ACUPUNCTURE IN MEDICINE | 1 | 0.137 |
| ADVANCES IN EXPERIMENTAL MEDICINE AND BIOLOGY | 1 | 0.137 |
| ADVANCES IN PAIN RESEARCH AND THERAPY | 1 | 0.137 |
| AMERICAN JOURNAL OF NEURORADIOLOGY | 1 | 0.137 |
| AMERICAN JOURNAL OF SURGERY | 1 | 0.137 |
| ANAESTHESIA AND INTENSIVE CARE | 1 | 0.137 |
| ANESTHESIOLOGY | 1 | 0.137 |
| ANKARA UNIVERSITESI VETERINER FAKULTESI DERGISI | 1 | 0.137 |
| ANNALS OF SAUDI MEDICINE | 1 | 0.137 |
| ANNALS OF THE NEW YORK ACADEMY OF SCIENCES | 1 | 0.137 |
| AUSTRALIAN OCCUPATIONAL THERAPY JOURNAL | 1 | 0.137 |
| AUTONOMIC NEUROSCIENCE BASIC CLINICAL | 1 | 0.137 |
| BEHAVIOURAL BRAIN RESEARCH | 1 | 0.137 |
| BEHAVIOURAL PHARMACOLOGY | 1 | 0.137 |
| BIOCHIMICA ET BIOPHYSICA ACTA MOLECULAR BASIS OF DISEASE | 1 | 0.137 |
| BIOLOGICAL RESEARCH FOR NURSING | 1 | 0.137 |
| BIOMED RESEARCH INTERNATIONAL | 1 | 0.137 |
| BIOMEDICAL RESEARCH INDIA | 1 | 0.137 |
| BMJ OPEN | 1 | 0.137 |
| BRAIN BEHAVIOR AND IMMUNITY | 1 | 0.137 |
| CANADIAN JOURNAL OF PHYSIOLOGY AND PHARMACOLOGY | 1 | 0.137 |
| CANADIAN MEDICAL ASSOCIATION JOURNAL | 1 | 0.137 |
| CARDIOLOGY IN THE YOUNG | 1 | 0.137 |
| CELL STEM CELL | 1 | 0.137 |
| CELL TRANSPLANTATION | 1 | 0.137 |
| CEREBRAL CORTEX | 1 | 0.137 |
| CHINESE MEDICAL JOURNAL | 1 | 0.137 |
| CLINICAL NEUROLOGY AND NEUROSURGERY | 1 | 0.137 |
| CLINICAL PHARMACOLOGY IN DRUG DEVELOPMENT | 1 | 0.137 |
| CLINICAL THERAPEUTICS | 1 | 0.137 |
| CNS DRUGS | 1 | 0.137 |
| COCHRANE DATABASE OF SYSTEMATIC REVIEWS | 1 | 0.137 |
| COMPUTER METHODS AND PROGRAMS IN BIOMEDICINE | 1 | 0.137 |
| CURRENT OPINION IN NEUROLOGY | 1 | 0.137 |
| CURRENT PHARMACEUTICAL DESIGN | 1 | 0.137 |
| CURRENT PROTEIN PEPTIDE SCIENCE | 1 | 0.137 |
| DRUG DEVELOPMENT RESEARCH | 1 | 0.137 |
| DRUGS | 1 | 0.137 |
| ELECTROMAGNETIC BIOLOGY AND MEDICINE | 1 | 0.137 |
| ENDOCRINOLOGY | 1 | 0.137 |
| ERGONOMICS | 1 | 0.137 |
| EUROPEAN JOURNAL OF CLINICAL PHARMACOLOGY | 1 | 0.137 |
| EUROPEAN JOURNAL OF NEUROLOGY | 1 | 0.137 |
| EUROPEAN JOURNAL OF NEUROSCIENCE | 1 | 0.137 |
| EUROPEAN REVIEW FOR MEDICAL AND PHARMACOLOGICAL SCIENCES | 1 | 0.137 |
| EXPERIMENTAL BRAIN RESEARCH | 1 | 0.137 |
| EXPERT OPINION ON BIOLOGICAL THERAPY | 1 | 0.137 |
| EXPLORE THE JOURNAL OF SCIENCE AND HEALING | 1 | 0.137 |
| FRONTIERS IN CELLULAR NEUROSCIENCE | 1 | 0.137 |
| FRONTIERS IN HUMAN NEUROSCIENCE | 1 | 0.137 |
| FRONTIERS IN NEUROLOGY | 1 | 0.137 |
| FRONTIERS IN NEUROSCIENCE | 1 | 0.137 |
| FRONTIERS IN PHARMACOLOGY | 1 | 0.137 |
| GENE THERAPY | 1 | 0.137 |
| INTERNATIONAL JOURNAL OF INDUSTRIAL ERGONOMICS | 1 | 0.137 |
| INTERNATIONAL JOURNAL OF PHARMACOLOGY | 1 | 0.137 |
| JCPSP JOURNAL OF THE COLLEGE OF PHYSICIANS AND SURGEONS PAKISTAN | 1 | 0.137 |
| JOURNAL OF BIOLOGICAL CHEMISTRY | 1 | 0.137 |
| JOURNAL OF BIOMEDICINE AND BIOTECHNOLOGY | 1 | 0.137 |
| JOURNAL OF CHEMICAL NEUROANATOMY | 1 | 0.137 |
| JOURNAL OF CLINICAL ANESTHESIA | 1 | 0.137 |
| JOURNAL OF CLINICAL NEUROSCIENCE | 1 | 0.137 |
| JOURNAL OF COMPARATIVE NEUROLOGY | 1 | 0.137 |
| JOURNAL OF CONTROLLED RELEASE | 1 | 0.137 |
| JOURNAL OF ENDOCRINOLOGICAL INVESTIGATION | 1 | 0.137 |
| JOURNAL OF KOREAN NEUROSURGICAL SOCIETY | 1 | 0.137 |
| JOURNAL OF MOLECULAR NEUROSCIENCE | 1 | 0.137 |
| JOURNAL OF MUSCULOSKELETAL PAIN | 1 | 0.137 |
| JOURNAL OF NEUROENGINEERING AND REHABILITATION | 1 | 0.137 |
| JOURNAL OF NEUROIMMUNE PHARMACOLOGY | 1 | 0.137 |
| JOURNAL OF NEUROIMMUNOLOGY | 1 | 0.137 |
| JOURNAL OF NEUROLOGIC PHYSICAL THERAPY | 1 | 0.137 |
| JOURNAL OF NEUROPHYSIOLOGY | 1 | 0.137 |
| JOURNAL OF NEUROSCIENCE NURSING | 1 | 0.137 |
| JOURNAL OF NEUROSURGICAL SCIENCES | 1 | 0.137 |
| JOURNAL OF NURSING RESEARCH | 1 | 0.137 |
| JOURNAL OF ORTHOPAEDIC RESEARCH | 1 | 0.137 |
| JOURNAL OF PHARMACOLOGICAL SCIENCES | 1 | 0.137 |
| JOURNAL OF PHYSICAL THERAPY SCIENCE | 1 | 0.137 |
| JOURNAL OF TRANSLATIONAL MEDICINE | 1 | 0.137 |
| KOREAN JOURNAL OF PHYSIOLOGY PHARMACOLOGY | 1 | 0.137 |
| MEDICAL HYPOTHESES | 1 | 0.137 |
| MEDICAL SCIENCE MONITOR | 1 | 0.137 |
| MOLECULAR MEDICINE | 1 | 0.137 |
| MOLECULAR MEDICINE REPORTS | 1 | 0.137 |
| MOLECULAR NEUROBIOLOGY | 1 | 0.137 |
| MOLECULES AND CELLS | 1 | 0.137 |
| NEURAL PLASTICITY | 1 | 0.137 |
| NEUROCHEMICAL RESEARCH | 1 | 0.137 |
| NEUROIMAGE CLINICAL | 1 | 0.137 |
| NEUROLOGICAL RESEARCH | 1 | 0.137 |
| NEURON | 1 | 0.137 |
| NEURONS AND NETWORKS IN THE SPINAL CORD | 1 | 0.137 |
| NEUROPEPTIDES | 1 | 0.137 |
| NEUROPHYSIOLOGY | 1 | 0.137 |
| NEUROREHABILITATION | 1 | 0.137 |
| NEUROSIGNALS | 1 | 0.137 |
| NEUROSURGERY | 1 | 0.137 |
| NEUROTHERAPEUTICS | 1 | 0.137 |
| ONCOLOGY | 1 | 0.137 |
| ORTHOPEDICS | 1 | 0.137 |
| PAKISTAN JOURNAL OF MEDICAL SCIENCES | 1 | 0.137 |
| PHYSICAL MEDICINE AND REHABILITATION CLINICS OF NORTH AMERICA | 1 | 0.137 |
| PHYSIOLOGICAL RESEARCH | 1 | 0.137 |
| REGENERATIVE BIOLOGY OF THE SPINE AND SPINAL CORD | 1 | 0.137 |
| SEMINARS IN IMMUNOLOGY | 1 | 0.137 |
| STEREOTACTIC AND FUNCTIONAL NEUROSURGERY | 1 | 0.137 |
| TOXINS | 1 | 0.137 |
| TRANSLATIONAL NEUROSCIENCE | 1 | 0.137 |
| TRENDS IN NEUROSCIENCES | 1 | 0.137 |
| TURKIYE FIZIKSEL TIP VE REHABILITASYON DERGISI TURKISH JOURNAL OF PHYSICAL MEDICINE AND REHABILITATION | 1 | 0.137 |
| SPINAL CORD | 88 | 12.055 |
| PAIN | 60 | 8.219 |
| ARCHIVES OF PHYSICAL MEDICINE AND REHABILITATION | 41 | 5.616 |
| JOURNAL OF NEUROTRAUMA | 33 | 4.521 |
| JOURNAL OF SPINAL CORD MEDICINE | 28 | 3.836 |
| EXPERIMENTAL NEUROLOGY | 22 | 3.014 |
| CLINICAL JOURNAL OF PAIN | 18 | 2.466 |
| JOURNAL OF PAIN | 18 | 2.466 |
| JOURNAL OF REHABILITATION RESEARCH AND DEVELOPMENT | 15 | 2.055 |
| JOURNAL OF NEUROSCIENCE | 14 | 1.918 |
| NEUROSCIENCE LETTERS | 14 | 1.918 |
| DISABILITY AND REHABILITATION | 12 | 1.644 |
| EUROPEAN JOURNAL OF PAIN | 11 | 1.507 |
| JOURNAL OF REHABILITATION MEDICINE | 11 | 1.507 |
| JOURNAL OF PAIN RESEARCH | 9 | 1.233 |
| SPINE | 9 | 1.233 |
| AMERICAN JOURNAL OF PHYSICAL MEDICINE REHABILITATION | 7 | 0.959 |
| NEUROLOGY | 7 | 0.959 |
| PM R | 7 | 0.959 |
| MOLECULAR PAIN | 6 | 0.822 |
| NEUROREHABILITATION AND NEURAL REPAIR | 6 | 0.822 |
| NEUROSCIENCE | 6 | 0.822 |
| PAIN PHYSICIAN | 6 | 0.822 |
| ACTA NEUROCHIRURGICA | 5 | 0.685 |
| ANESTHESIA AND ANALGESIA | 5 | 0.685 |
| PARAPLEGIA | 5 | 0.685 |
| PLOS ONE | 5 | 0.685 |
| BRAIN | 4 | 0.548 |
| BRAIN RESEARCH | 4 | 0.548 |
| BRAIN RESEARCH BULLETIN | 4 | 0.548 |
| GLIA | 4 | 0.548 |
| JOURNAL OF NEUROSURGERY | 4 | 0.548 |
| JOURNAL OF NEUROSURGERY SPINE | 4 | 0.548 |
| SCIENTIFIC REPORTS | 4 | 0.548 |
| CLINICAL NEUROPHYSIOLOGY | 3 | 0.411 |
| CLINICAL REHABILITATION | 3 | 0.411 |
| EUROPEAN JOURNAL OF PHARMACOLOGY | 3 | 0.411 |
| FRONTIERS IN PHYSIOLOGY | 3 | 0.411 |
| JOURNAL OF BACK AND MUSCULOSKELETAL REHABILITATION | 3 | 0.411 |
| JOURNAL OF NEUROCHEMISTRY | 3 | 0.411 |
| JOURNAL OF NEUROINFLAMMATION | 3 | 0.411 |
| JOURNAL OF PHARMACOLOGY AND EXPERIMENTAL THERAPEUTICS | 3 | 0.411 |
| MEDICINE | 3 | 0.411 |
| NEURAL REGENERATION RESEARCH | 3 | 0.411 |
| PAIN MEDICINE | 3 | 0.411 |
| PHYSICAL THERAPY | 3 | 0.411 |
| PROCEEDINGS OF THE NATIONAL ACADEMY OF SCIENCES OF THE UNITED STATES OF AMERICA | 3 | 0.411 |
| SOMATOSENSORY AND MOTOR RESEARCH | 3 | 0.411 |
| SPINE JOURNAL | 3 | 0.411 |
| WORLD NEUROSURGERY | 3 | 0.411 |
| ACTA MEDICA MEDITERRANEA | 2 | 0.274 |
| ANNALS OF NEUROLOGY | 2 | 0.274 |
| ANNALS OF PHYSICAL AND REHABILITATION MEDICINE | 2 | 0.274 |
| ARQUIVOS DE NEURO PSIQUIATRIA | 2 | 0.274 |
| BMC NEUROLOGY | 2 | 0.274 |
| BRAIN RESEARCH REVIEWS | 2 | 0.274 |
| BRITISH JOURNAL OF PHARMACOLOGY | 2 | 0.274 |
| CELL DEATH DISEASE | 2 | 0.274 |
| CLINICAL ORTHOPAEDICS AND RELATED RESEARCH | 2 | 0.274 |
| COMPLEMENTARY THERAPIES IN MEDICINE | 2 | 0.274 |
| CURRENT PAIN AND HEADACHE REPORTS | 2 | 0.274 |
| EUROPEAN JOURNAL OF PHYSICAL AND REHABILITATION MEDICINE | 2 | 0.274 |
| EXPERIMENTAL AND THERAPEUTIC MEDICINE | 2 | 0.274 |
| FRONTIERS IN SYSTEMS NEUROSCIENCE | 2 | 0.274 |
| INTERNATIONAL JOURNAL OF REHABILITATION RESEARCH | 2 | 0.274 |
| JOURNAL OF NEUROLOGY NEUROSURGERY AND PSYCHIATRY | 2 | 0.274 |
| JOURNAL OF NEUROSCIENCE RESEARCH | 2 | 0.274 |
| JOURNAL OF ORTHOPAEDIC SURGERY AND RESEARCH | 2 | 0.274 |
| JOURNAL OF PAIN AND SYMPTOM MANAGEMENT | 2 | 0.274 |
| METABOLIC BRAIN DISEASE | 2 | 0.274 |
| MOLECULAR BRAIN RESEARCH | 2 | 0.274 |
| MOLECULAR THERAPY | 2 | 0.274 |
| NEUROBIOLOGY OF DISEASE | 2 | 0.274 |
| NEUROIMAGE | 2 | 0.274 |
| NEUROMODULATION | 2 | 0.274 |
| NEUROPHARMACOLOGY | 2 | 0.274 |
| NEUROSCIENCE BULLETIN | 2 | 0.274 |
| NEUROSCIENCE RESEARCH | 2 | 0.274 |
| NEUROSCIENCES | 2 | 0.274 |
| PAIN CLINIC | 2 | 0.274 |
| PAIN PRACTICE | 2 | 0.274 |
| PHARMACOLOGY BIOCHEMISTRY AND BEHAVIOR | 2 | 0.274 |
| QUALITY OF LIFE RESEARCH | 2 | 0.274 |
| REGIONAL ANESTHESIA AND PAIN MEDICINE | 2 | 0.274 |
| RESTORATIVE NEUROLOGY AND NEUROSCIENCE | 2 | 0.274 |
| STEM CELL RESEARCH THERAPY | 2 | 0.274 |
| STEM CELLS | 2 | 0.274 |
| ACTA ANAESTHESIOLOGICA SCANDINAVICA | 1 | 0.137 |
| ACTA BIOCHIMICA ET BIOPHYSICA SINICA | 1 | 0.137 |
| ACTA PHARMACOLOGICA SINICA | 1 | 0.137 |
| ACUPUNCTURE IN MEDICINE | 1 | 0.137 |
| ADVANCES IN EXPERIMENTAL MEDICINE AND BIOLOGY | 1 | 0.137 |
| ADVANCES IN PAIN RESEARCH AND THERAPY | 1 | 0.137 |
| AMERICAN JOURNAL OF NEURORADIOLOGY | 1 | 0.137 |
| AMERICAN JOURNAL OF SURGERY | 1 | 0.137 |
| ANAESTHESIA AND INTENSIVE CARE | 1 | 0.137 |
| ANESTHESIOLOGY | 1 | 0.137 |
| ANKARA UNIVERSITESI VETERINER FAKULTESI DERGISI | 1 | 0.137 |
| ANNALS OF SAUDI MEDICINE | 1 | 0.137 |
| ANNALS OF THE NEW YORK ACADEMY OF SCIENCES | 1 | 0.137 |
| AUSTRALIAN OCCUPATIONAL THERAPY JOURNAL | 1 | 0.137 |
| AUTONOMIC NEUROSCIENCE BASIC CLINICAL | 1 | 0.137 |
| BEHAVIOURAL BRAIN RESEARCH | 1 | 0.137 |
| BEHAVIOURAL PHARMACOLOGY | 1 | 0.137 |
| BIOCHIMICA ET BIOPHYSICA ACTA MOLECULAR BASIS OF DISEASE | 1 | 0.137 |
| BIOLOGICAL RESEARCH FOR NURSING | 1 | 0.137 |
| BIOMED RESEARCH INTERNATIONAL | 1 | 0.137 |
| BIOMEDICAL RESEARCH INDIA | 1 | 0.137 |
| BMJ OPEN | 1 | 0.137 |
| BRAIN BEHAVIOR AND IMMUNITY | 1 | 0.137 |
| CANADIAN JOURNAL OF PHYSIOLOGY AND PHARMACOLOGY | 1 | 0.137 |
| CANADIAN MEDICAL ASSOCIATION JOURNAL | 1 | 0.137 |
| CARDIOLOGY IN THE YOUNG | 1 | 0.137 |
| CELL STEM CELL | 1 | 0.137 |
| CELL TRANSPLANTATION | 1 | 0.137 |
| CEREBRAL CORTEX | 1 | 0.137 |
| CHINESE MEDICAL JOURNAL | 1 | 0.137 |
| CLINICAL NEUROLOGY AND NEUROSURGERY | 1 | 0.137 |
| CLINICAL PHARMACOLOGY IN DRUG DEVELOPMENT | 1 | 0.137 |
| CLINICAL THERAPEUTICS | 1 | 0.137 |
| CNS DRUGS | 1 | 0.137 |
| COCHRANE DATABASE OF SYSTEMATIC REVIEWS | 1 | 0.137 |
| COMPUTER METHODS AND PROGRAMS IN BIOMEDICINE | 1 | 0.137 |
| CURRENT OPINION IN NEUROLOGY | 1 | 0.137 |
| CURRENT PHARMACEUTICAL DESIGN | 1 | 0.137 |
| CURRENT PROTEIN PEPTIDE SCIENCE | 1 | 0.137 |
| DRUG DEVELOPMENT RESEARCH | 1 | 0.137 |
| DRUGS | 1 | 0.137 |
| ELECTROMAGNETIC BIOLOGY AND MEDICINE | 1 | 0.137 |
| ENDOCRINOLOGY | 1 | 0.137 |
| ERGONOMICS | 1 | 0.137 |
| EUROPEAN JOURNAL OF CLINICAL PHARMACOLOGY | 1 | 0.137 |
| EUROPEAN JOURNAL OF NEUROLOGY | 1 | 0.137 |
| EUROPEAN JOURNAL OF NEUROSCIENCE | 1 | 0.137 |
| EUROPEAN REVIEW FOR MEDICAL AND PHARMACOLOGICAL SCIENCES | 1 | 0.137 |
| EXPERIMENTAL BRAIN RESEARCH | 1 | 0.137 |
| EXPERT OPINION ON BIOLOGICAL THERAPY | 1 | 0.137 |
| EXPLORE THE JOURNAL OF SCIENCE AND HEALING | 1 | 0.137 |
| FRONTIERS IN CELLULAR NEUROSCIENCE | 1 | 0.137 |
| FRONTIERS IN HUMAN NEUROSCIENCE | 1 | 0.137 |
| FRONTIERS IN NEUROLOGY | 1 | 0.137 |
| FRONTIERS IN NEUROSCIENCE | 1 | 0.137 |
| FRONTIERS IN PHARMACOLOGY | 1 | 0.137 |
| GENE THERAPY | 1 | 0.137 |
| INTERNATIONAL JOURNAL OF INDUSTRIAL ERGONOMICS | 1 | 0.137 |
| INTERNATIONAL JOURNAL OF PHARMACOLOGY | 1 | 0.137 |
| JCPSP JOURNAL OF THE COLLEGE OF PHYSICIANS AND SURGEONS PAKISTAN | 1 | 0.137 |
| JOURNAL OF BIOLOGICAL CHEMISTRY | 1 | 0.137 |
| JOURNAL OF BIOMEDICINE AND BIOTECHNOLOGY | 1 | 0.137 |
| JOURNAL OF CHEMICAL NEUROANATOMY | 1 | 0.137 |
| JOURNAL OF CLINICAL ANESTHESIA | 1 | 0.137 |
| JOURNAL OF CLINICAL NEUROSCIENCE | 1 | 0.137 |
| JOURNAL OF COMPARATIVE NEUROLOGY | 1 | 0.137 |
| JOURNAL OF CONTROLLED RELEASE | 1 | 0.137 |
| JOURNAL OF ENDOCRINOLOGICAL INVESTIGATION | 1 | 0.137 |
| JOURNAL OF KOREAN NEUROSURGICAL SOCIETY | 1 | 0.137 |
| JOURNAL OF MOLECULAR NEUROSCIENCE | 1 | 0.137 |
| JOURNAL OF MUSCULOSKELETAL PAIN | 1 | 0.137 |
| JOURNAL OF NEUROENGINEERING AND REHABILITATION | 1 | 0.137 |
| JOURNAL OF NEUROIMMUNE PHARMACOLOGY | 1 | 0.137 |
| JOURNAL OF NEUROIMMUNOLOGY | 1 | 0.137 |
| JOURNAL OF NEUROLOGIC PHYSICAL THERAPY | 1 | 0.137 |
| JOURNAL OF NEUROPHYSIOLOGY | 1 | 0.137 |
| JOURNAL OF NEUROSCIENCE NURSING | 1 | 0.137 |
| JOURNAL OF NEUROSURGICAL SCIENCES | 1 | 0.137 |
| JOURNAL OF NURSING RESEARCH | 1 | 0.137 |
| JOURNAL OF ORTHOPAEDIC RESEARCH | 1 | 0.137 |
| JOURNAL OF PHARMACOLOGICAL SCIENCES | 1 | 0.137 |
| JOURNAL OF PHYSICAL THERAPY SCIENCE | 1 | 0.137 |
| JOURNAL OF TRANSLATIONAL MEDICINE | 1 | 0.137 |
| KOREAN JOURNAL OF PHYSIOLOGY PHARMACOLOGY | 1 | 0.137 |
| MEDICAL HYPOTHESES | 1 | 0.137 |
| MEDICAL SCIENCE MONITOR | 1 | 0.137 |
| MOLECULAR MEDICINE | 1 | 0.137 |
| MOLECULAR MEDICINE REPORTS | 1 | 0.137 |
| MOLECULAR NEUROBIOLOGY | 1 | 0.137 |
| MOLECULES AND CELLS | 1 | 0.137 |
| NEURAL PLASTICITY | 1 | 0.137 |
| NEUROCHEMICAL RESEARCH | 1 | 0.137 |
| NEUROIMAGE CLINICAL | 1 | 0.137 |
| NEUROLOGICAL RESEARCH | 1 | 0.137 |
| NEURON | 1 | 0.137 |
| NEURONS AND NETWORKS IN THE SPINAL CORD | 1 | 0.137 |
| NEUROPEPTIDES | 1 | 0.137 |
| NEUROPHYSIOLOGY | 1 | 0.137 |
| NEUROREHABILITATION | 1 | 0.137 |
| NEUROSIGNALS | 1 | 0.137 |
| NEUROSURGERY | 1 | 0.137 |
| NEUROTHERAPEUTICS | 1 | 0.137 |
| ONCOLOGY | 1 | 0.137 |
| ORTHOPEDICS | 1 | 0.137 |
| PAKISTAN JOURNAL OF MEDICAL SCIENCES | 1 | 0.137 |
| PHYSICAL MEDICINE AND REHABILITATION CLINICS OF NORTH AMERICA | 1 | 0.137 |
| PHYSIOLOGICAL RESEARCH | 1 | 0.137 |
| REGENERATIVE BIOLOGY OF THE SPINE AND SPINAL CORD | 1 | 0.137 |
| SEMINARS IN IMMUNOLOGY | 1 | 0.137 |
| STEREOTACTIC AND FUNCTIONAL NEUROSURGERY | 1 | 0.137 |
| TOXINS | 1 | 0.137 |
| TRANSLATIONAL NEUROSCIENCE | 1 | 0.137 |
| TRENDS IN NEUROSCIENCES | 1 | 0.137 |
| TURKIYE FIZIKSEL TIP VE REHABILITASYON DERGISI TURKISH JOURNAL OF PHYSICAL MEDICINE AND REHABILITATION | 1 | 0.137 |
| SPINAL CORD | 88 | 12.055 |
| PAIN | 60 | 8.219 |
| ARCHIVES OF PHYSICAL MEDICINE AND REHABILITATION | 41 | 5.616 |
| JOURNAL OF NEUROTRAUMA | 33 | 4.521 |
| JOURNAL OF SPINAL CORD MEDICINE | 28 | 3.836 |
| EXPERIMENTAL NEUROLOGY | 22 | 3.014 |
| CLINICAL JOURNAL OF PAIN | 18 | 2.466 |
| JOURNAL OF PAIN | 18 | 2.466 |
| JOURNAL OF REHABILITATION RESEARCH AND DEVELOPMENT | 15 | 2.055 |
| JOURNAL OF NEUROSCIENCE | 14 | 1.918 |
| NEUROSCIENCE LETTERS | 14 | 1.918 |
| DISABILITY AND REHABILITATION | 12 | 1.644 |
| EUROPEAN JOURNAL OF PAIN | 11 | 1.507 |
| JOURNAL OF REHABILITATION MEDICINE | 11 | 1.507 |
| JOURNAL OF PAIN RESEARCH | 9 | 1.233 |
| SPINE | 9 | 1.233 |
| AMERICAN JOURNAL OF PHYSICAL MEDICINE REHABILITATION | 7 | 0.959 |
| NEUROLOGY | 7 | 0.959 |
| PM R | 7 | 0.959 |
| MOLECULAR PAIN | 6 | 0.822 |
| NEUROREHABILITATION AND NEURAL REPAIR | 6 | 0.822 |
| NEUROSCIENCE | 6 | 0.822 |
| PAIN PHYSICIAN | 6 | 0.822 |
| ACTA NEUROCHIRURGICA | 5 | 0.685 |
| ANESTHESIA AND ANALGESIA | 5 | 0.685 |
| PARAPLEGIA | 5 | 0.685 |
| PLOS ONE | 5 | 0.685 |
| BRAIN | 4 | 0.548 |
| BRAIN RESEARCH | 4 | 0.548 |
| BRAIN RESEARCH BULLETIN | 4 | 0.548 |
| GLIA | 4 | 0.548 |
| JOURNAL OF NEUROSURGERY | 4 | 0.548 |
| JOURNAL OF NEUROSURGERY SPINE | 4 | 0.548 |
| SCIENTIFIC REPORTS | 4 | 0.548 |
| CLINICAL NEUROPHYSIOLOGY | 3 | 0.411 |
| CLINICAL REHABILITATION | 3 | 0.411 |
| EUROPEAN JOURNAL OF PHARMACOLOGY | 3 | 0.411 |
| FRONTIERS IN PHYSIOLOGY | 3 | 0.411 |
| JOURNAL OF BACK AND MUSCULOSKELETAL REHABILITATION | 3 | 0.411 |
| JOURNAL OF NEUROCHEMISTRY | 3 | 0.411 |
| JOURNAL OF NEUROINFLAMMATION | 3 | 0.411 |
| JOURNAL OF PHARMACOLOGY AND EXPERIMENTAL THERAPEUTICS | 3 | 0.411 |
| MEDICINE | 3 | 0.411 |
| NEURAL REGENERATION RESEARCH | 3 | 0.411 |
| PAIN MEDICINE | 3 | 0.411 |
| PHYSICAL THERAPY | 3 | 0.411 |
| PROCEEDINGS OF THE NATIONAL ACADEMY OF SCIENCES OF THE UNITED STATES OF AMERICA | 3 | 0.411 |
| SOMATOSENSORY AND MOTOR RESEARCH | 3 | 0.411 |
| SPINE JOURNAL | 3 | 0.411 |
| WORLD NEUROSURGERY | 3 | 0.411 |
| ACTA MEDICA MEDITERRANEA | 2 | 0.274 |
| ANNALS OF NEUROLOGY | 2 | 0.274 |
| ANNALS OF PHYSICAL AND REHABILITATION MEDICINE | 2 | 0.274 |
| ARQUIVOS DE NEURO PSIQUIATRIA | 2 | 0.274 |
| BMC NEUROLOGY | 2 | 0.274 |
| BRAIN RESEARCH REVIEWS | 2 | 0.274 |
| BRITISH JOURNAL OF PHARMACOLOGY | 2 | 0.274 |
| CELL DEATH DISEASE | 2 | 0.274 |
| CLINICAL ORTHOPAEDICS AND RELATED RESEARCH | 2 | 0.274 |
| COMPLEMENTARY THERAPIES IN MEDICINE | 2 | 0.274 |
| CURRENT PAIN AND HEADACHE REPORTS | 2 | 0.274 |
| EUROPEAN JOURNAL OF PHYSICAL AND REHABILITATION MEDICINE | 2 | 0.274 |
| EXPERIMENTAL AND THERAPEUTIC MEDICINE | 2 | 0.274 |
| FRONTIERS IN SYSTEMS NEUROSCIENCE | 2 | 0.274 |
| INTERNATIONAL JOURNAL OF REHABILITATION RESEARCH | 2 | 0.274 |
| JOURNAL OF NEUROLOGY NEUROSURGERY AND PSYCHIATRY | 2 | 0.274 |
| JOURNAL OF NEUROSCIENCE RESEARCH | 2 | 0.274 |
| JOURNAL OF ORTHOPAEDIC SURGERY AND RESEARCH | 2 | 0.274 |
| JOURNAL OF PAIN AND SYMPTOM MANAGEMENT | 2 | 0.274 |
| METABOLIC BRAIN DISEASE | 2 | 0.274 |
| MOLECULAR BRAIN RESEARCH | 2 | 0.274 |
| MOLECULAR THERAPY | 2 | 0.274 |
| NEUROBIOLOGY OF DISEASE | 2 | 0.274 |
| NEUROIMAGE | 2 | 0.274 |
| NEUROMODULATION | 2 | 0.274 |
| NEUROPHARMACOLOGY | 2 | 0.274 |
| NEUROSCIENCE BULLETIN | 2 | 0.274 |
| NEUROSCIENCE RESEARCH | 2 | 0.274 |
| NEUROSCIENCES | 2 | 0.274 |
| PAIN CLINIC | 2 | 0.274 |
| PAIN PRACTICE | 2 | 0.274 |
| PHARMACOLOGY BIOCHEMISTRY AND BEHAVIOR | 2 | 0.274 |
| QUALITY OF LIFE RESEARCH | 2 | 0.274 |
| REGIONAL ANESTHESIA AND PAIN MEDICINE | 2 | 0.274 |
| RESTORATIVE NEUROLOGY AND NEUROSCIENCE | 2 | 0.274 |
| STEM CELL RESEARCH THERAPY | 2 | 0.274 |
| STEM CELLS | 2 | 0.274 |
| ACTA ANAESTHESIOLOGICA SCANDINAVICA | 1 | 0.137 |
| ACTA BIOCHIMICA ET BIOPHYSICA SINICA | 1 | 0.137 |
| ACTA PHARMACOLOGICA SINICA | 1 | 0.137 |
| ACUPUNCTURE IN MEDICINE | 1 | 0.137 |
| ADVANCES IN EXPERIMENTAL MEDICINE AND BIOLOGY | 1 | 0.137 |
| ADVANCES IN PAIN RESEARCH AND THERAPY | 1 | 0.137 |
| AMERICAN JOURNAL OF NEURORADIOLOGY | 1 | 0.137 |
| AMERICAN JOURNAL OF SURGERY | 1 | 0.137 |
| ANAESTHESIA AND INTENSIVE CARE | 1 | 0.137 |
| ANESTHESIOLOGY | 1 | 0.137 |
| ANKARA UNIVERSITESI VETERINER FAKULTESI DERGISI | 1 | 0.137 |
| ANNALS OF SAUDI MEDICINE | 1 | 0.137 |
| ANNALS OF THE NEW YORK ACADEMY OF SCIENCES | 1 | 0.137 |
| AUSTRALIAN OCCUPATIONAL THERAPY JOURNAL | 1 | 0.137 |
| AUTONOMIC NEUROSCIENCE BASIC CLINICAL | 1 | 0.137 |
| BEHAVIOURAL BRAIN RESEARCH | 1 | 0.137 |
| BEHAVIOURAL PHARMACOLOGY | 1 | 0.137 |
| BIOCHIMICA ET BIOPHYSICA ACTA MOLECULAR BASIS OF DISEASE | 1 | 0.137 |
| BIOLOGICAL RESEARCH FOR NURSING | 1 | 0.137 |
| BIOMED RESEARCH INTERNATIONAL | 1 | 0.137 |
| BIOMEDICAL RESEARCH INDIA | 1 | 0.137 |
| BMJ OPEN | 1 | 0.137 |
| BRAIN BEHAVIOR AND IMMUNITY | 1 | 0.137 |
| CANADIAN JOURNAL OF PHYSIOLOGY AND PHARMACOLOGY | 1 | 0.137 |
| CANADIAN MEDICAL ASSOCIATION JOURNAL | 1 | 0.137 |
| CARDIOLOGY IN THE YOUNG | 1 | 0.137 |
| CELL STEM CELL | 1 | 0.137 |
| CELL TRANSPLANTATION | 1 | 0.137 |
| CEREBRAL CORTEX | 1 | 0.137 |
| CHINESE MEDICAL JOURNAL | 1 | 0.137 |
| CLINICAL NEUROLOGY AND NEUROSURGERY | 1 | 0.137 |
| CLINICAL PHARMACOLOGY IN DRUG DEVELOPMENT | 1 | 0.137 |
| CLINICAL THERAPEUTICS | 1 | 0.137 |
| CNS DRUGS | 1 | 0.137 |
| COCHRANE DATABASE OF SYSTEMATIC REVIEWS | 1 | 0.137 |
| COMPUTER METHODS AND PROGRAMS IN BIOMEDICINE | 1 | 0.137 |
| CURRENT OPINION IN NEUROLOGY | 1 | 0.137 |
| CURRENT PHARMACEUTICAL DESIGN | 1 | 0.137 |
| CURRENT PROTEIN PEPTIDE SCIENCE | 1 | 0.137 |
| DRUG DEVELOPMENT RESEARCH | 1 | 0.137 |
| DRUGS | 1 | 0.137 |
| ELECTROMAGNETIC BIOLOGY AND MEDICINE | 1 | 0.137 |
| ENDOCRINOLOGY | 1 | 0.137 |
| ERGONOMICS | 1 | 0.137 |
| EUROPEAN JOURNAL OF CLINICAL PHARMACOLOGY | 1 | 0.137 |
| EUROPEAN JOURNAL OF NEUROLOGY | 1 | 0.137 |
| EUROPEAN JOURNAL OF NEUROSCIENCE | 1 | 0.137 |
| EUROPEAN REVIEW FOR MEDICAL AND PHARMACOLOGICAL SCIENCES | 1 | 0.137 |
| EXPERIMENTAL BRAIN RESEARCH | 1 | 0.137 |
| EXPERT OPINION ON BIOLOGICAL THERAPY | 1 | 0.137 |
| EXPLORE THE JOURNAL OF SCIENCE AND HEALING | 1 | 0.137 |
| FRONTIERS IN CELLULAR NEUROSCIENCE | 1 | 0.137 |
| FRONTIERS IN HUMAN NEUROSCIENCE | 1 | 0.137 |
| FRONTIERS IN NEUROLOGY | 1 | 0.137 |
| FRONTIERS IN NEUROSCIENCE | 1 | 0.137 |
| FRONTIERS IN PHARMACOLOGY | 1 | 0.137 |
| GENE THERAPY | 1 | 0.137 |
| INTERNATIONAL JOURNAL OF INDUSTRIAL ERGONOMICS | 1 | 0.137 |
| INTERNATIONAL JOURNAL OF PHARMACOLOGY | 1 | 0.137 |
| JCPSP JOURNAL OF THE COLLEGE OF PHYSICIANS AND SURGEONS PAKISTAN | 1 | 0.137 |
| JOURNAL OF BIOLOGICAL CHEMISTRY | 1 | 0.137 |
| JOURNAL OF BIOMEDICINE AND BIOTECHNOLOGY | 1 | 0.137 |
| JOURNAL OF CHEMICAL NEUROANATOMY | 1 | 0.137 |
| JOURNAL OF CLINICAL ANESTHESIA | 1 | 0.137 |
| JOURNAL OF CLINICAL NEUROSCIENCE | 1 | 0.137 |
| JOURNAL OF COMPARATIVE NEUROLOGY | 1 | 0.137 |
| JOURNAL OF CONTROLLED RELEASE | 1 | 0.137 |
| JOURNAL OF ENDOCRINOLOGICAL INVESTIGATION | 1 | 0.137 |
| JOURNAL OF KOREAN NEUROSURGICAL SOCIETY | 1 | 0.137 |
| JOURNAL OF MOLECULAR NEUROSCIENCE | 1 | 0.137 |
| JOURNAL OF MUSCULOSKELETAL PAIN | 1 | 0.137 |
| JOURNAL OF NEUROENGINEERING AND REHABILITATION | 1 | 0.137 |
| JOURNAL OF NEUROIMMUNE PHARMACOLOGY | 1 | 0.137 |
| JOURNAL OF NEUROIMMUNOLOGY | 1 | 0.137 |
| JOURNAL OF NEUROLOGIC PHYSICAL THERAPY | 1 | 0.137 |
| JOURNAL OF NEUROPHYSIOLOGY | 1 | 0.137 |
| JOURNAL OF NEUROSCIENCE NURSING | 1 | 0.137 |
| JOURNAL OF NEUROSURGICAL SCIENCES | 1 | 0.137 |
| JOURNAL OF NURSING RESEARCH | 1 | 0.137 |
| JOURNAL OF ORTHOPAEDIC RESEARCH | 1 | 0.137 |
| JOURNAL OF PHARMACOLOGICAL SCIENCES | 1 | 0.137 |
| JOURNAL OF PHYSICAL THERAPY SCIENCE | 1 | 0.137 |
| JOURNAL OF TRANSLATIONAL MEDICINE | 1 | 0.137 |
| KOREAN JOURNAL OF PHYSIOLOGY PHARMACOLOGY | 1 | 0.137 |
| MEDICAL HYPOTHESES | 1 | 0.137 |
| MEDICAL SCIENCE MONITOR | 1 | 0.137 |
| MOLECULAR MEDICINE | 1 | 0.137 |
| MOLECULAR MEDICINE REPORTS | 1 | 0.137 |
| MOLECULAR NEUROBIOLOGY | 1 | 0.137 |
| MOLECULES AND CELLS | 1 | 0.137 |
| NEURAL PLASTICITY | 1 | 0.137 |
| NEUROCHEMICAL RESEARCH | 1 | 0.137 |
| NEUROIMAGE CLINICAL | 1 | 0.137 |
| NEUROLOGICAL RESEARCH | 1 | 0.137 |
| NEURON | 1 | 0.137 |
| NEURONS AND NETWORKS IN THE SPINAL CORD | 1 | 0.137 |
| NEUROPEPTIDES | 1 | 0.137 |
| NEUROPHYSIOLOGY | 1 | 0.137 |
| NEUROREHABILITATION | 1 | 0.137 |
| NEUROSIGNALS | 1 | 0.137 |
| NEUROSURGERY | 1 | 0.137 |
| NEUROTHERAPEUTICS | 1 | 0.137 |
| ONCOLOGY | 1 | 0.137 |
| ORTHOPEDICS | 1 | 0.137 |
| PAKISTAN JOURNAL OF MEDICAL SCIENCES | 1 | 0.137 |
| PHYSICAL MEDICINE AND REHABILITATION CLINICS OF NORTH AMERICA | 1 | 0.137 |
| PHYSIOLOGICAL RESEARCH | 1 | 0.137 |
| REGENERATIVE BIOLOGY OF THE SPINE AND SPINAL CORD | 1 | 0.137 |
| SEMINARS IN IMMUNOLOGY | 1 | 0.137 |
| STEREOTACTIC AND FUNCTIONAL NEUROSURGERY | 1 | 0.137 |
| TOXINS | 1 | 0.137 |
| TRANSLATIONAL NEUROSCIENCE | 1 | 0.137 |
| TRENDS IN NEUROSCIENCES | 1 | 0.137 |
| TURKIYE FIZIKSEL TIP VE REHABILITASYON DERGISI TURKISH JOURNAL OF PHYSICAL MEDICINE AND REHABILITATION | 1 | 0.137 |
| SPINAL CORD | 88 | 12.055 |
| PAIN | 60 | 8.219 |
| ARCHIVES OF PHYSICAL MEDICINE AND REHABILITATION | 41 | 5.616 |
| JOURNAL OF NEUROTRAUMA | 33 | 4.521 |
| JOURNAL OF SPINAL CORD MEDICINE | 28 | 3.836 |
| EXPERIMENTAL NEUROLOGY | 22 | 3.014 |
| CLINICAL JOURNAL OF PAIN | 18 | 2.466 |
| JOURNAL OF PAIN | 18 | 2.466 |
| JOURNAL OF REHABILITATION RESEARCH AND DEVELOPMENT | 15 | 2.055 |
| JOURNAL OF NEUROSCIENCE | 14 | 1.918 |
| NEUROSCIENCE LETTERS | 14 | 1.918 |
| DISABILITY AND REHABILITATION | 12 | 1.644 |
| EUROPEAN JOURNAL OF PAIN | 11 | 1.507 |
| JOURNAL OF REHABILITATION MEDICINE | 11 | 1.507 |
| JOURNAL OF PAIN RESEARCH | 9 | 1.233 |
| SPINE | 9 | 1.233 |
| AMERICAN JOURNAL OF PHYSICAL MEDICINE REHABILITATION | 7 | 0.959 |
| NEUROLOGY | 7 | 0.959 |
| PM R | 7 | 0.959 |
| MOLECULAR PAIN | 6 | 0.822 |
| NEUROREHABILITATION AND NEURAL REPAIR | 6 | 0.822 |
| NEUROSCIENCE | 6 | 0.822 |
| PAIN PHYSICIAN | 6 | 0.822 |
| ACTA NEUROCHIRURGICA | 5 | 0.685 |
| ANESTHESIA AND ANALGESIA | 5 | 0.685 |
| PARAPLEGIA | 5 | 0.685 |
| PLOS ONE | 5 | 0.685 |
| BRAIN | 4 | 0.548 |
| BRAIN RESEARCH | 4 | 0.548 |
| BRAIN RESEARCH BULLETIN | 4 | 0.548 |
| GLIA | 4 | 0.548 |
| JOURNAL OF NEUROSURGERY | 4 | 0.548 |
| JOURNAL OF NEUROSURGERY SPINE | 4 | 0.548 |
| SCIENTIFIC REPORTS | 4 | 0.548 |
| CLINICAL NEUROPHYSIOLOGY | 3 | 0.411 |
| CLINICAL REHABILITATION | 3 | 0.411 |
| EUROPEAN JOURNAL OF PHARMACOLOGY | 3 | 0.411 |
| FRONTIERS IN PHYSIOLOGY | 3 | 0.411 |
| JOURNAL OF BACK AND MUSCULOSKELETAL REHABILITATION | 3 | 0.411 |
| JOURNAL OF NEUROCHEMISTRY | 3 | 0.411 |
| JOURNAL OF NEUROINFLAMMATION | 3 | 0.411 |
| JOURNAL OF PHARMACOLOGY AND EXPERIMENTAL THERAPEUTICS | 3 | 0.411 |
| MEDICINE | 3 | 0.411 |
| NEURAL REGENERATION RESEARCH | 3 | 0.411 |
| PAIN MEDICINE | 3 | 0.411 |
| PHYSICAL THERAPY | 3 | 0.411 |
| PROCEEDINGS OF THE NATIONAL ACADEMY OF SCIENCES OF THE UNITED STATES OF AMERICA | 3 | 0.411 |
| SOMATOSENSORY AND MOTOR RESEARCH | 3 | 0.411 |
| SPINE JOURNAL | 3 | 0.411 |
| WORLD NEUROSURGERY | 3 | 0.411 |
| ACTA MEDICA MEDITERRANEA | 2 | 0.274 |
| ANNALS OF NEUROLOGY | 2 | 0.274 |
| ANNALS OF PHYSICAL AND REHABILITATION MEDICINE | 2 | 0.274 |
| ARQUIVOS DE NEURO PSIQUIATRIA | 2 | 0.274 |
| BMC NEUROLOGY | 2 | 0.274 |
| BRAIN RESEARCH REVIEWS | 2 | 0.274 |
| BRITISH JOURNAL OF PHARMACOLOGY | 2 | 0.274 |
| CELL DEATH DISEASE | 2 | 0.274 |
| CLINICAL ORTHOPAEDICS AND RELATED RESEARCH | 2 | 0.274 |
| COMPLEMENTARY THERAPIES IN MEDICINE | 2 | 0.274 |
| CURRENT PAIN AND HEADACHE REPORTS | 2 | 0.274 |
| EUROPEAN JOURNAL OF PHYSICAL AND REHABILITATION MEDICINE | 2 | 0.274 |
| EXPERIMENTAL AND THERAPEUTIC MEDICINE | 2 | 0.274 |
| FRONTIERS IN SYSTEMS NEUROSCIENCE | 2 | 0.274 |
| INTERNATIONAL JOURNAL OF REHABILITATION RESEARCH | 2 | 0.274 |
| JOURNAL OF NEUROLOGY NEUROSURGERY AND PSYCHIATRY | 2 | 0.274 |
| JOURNAL OF NEUROSCIENCE RESEARCH | 2 | 0.274 |
| JOURNAL OF ORTHOPAEDIC SURGERY AND RESEARCH | 2 | 0.274 |
| JOURNAL OF PAIN AND SYMPTOM MANAGEMENT | 2 | 0.274 |
| METABOLIC BRAIN DISEASE | 2 | 0.274 |
| MOLECULAR BRAIN RESEARCH | 2 | 0.274 |
| MOLECULAR THERAPY | 2 | 0.274 |
| NEUROBIOLOGY OF DISEASE | 2 | 0.274 |
| NEUROIMAGE | 2 | 0.274 |
| NEUROMODULATION | 2 | 0.274 |
| NEUROPHARMACOLOGY | 2 | 0.274 |
| NEUROSCIENCE BULLETIN | 2 | 0.274 |
| NEUROSCIENCE RESEARCH | 2 | 0.274 |
| NEUROSCIENCES | 2 | 0.274 |
| PAIN CLINIC | 2 | 0.274 |
| PAIN PRACTICE | 2 | 0.274 |
| PHARMACOLOGY BIOCHEMISTRY AND BEHAVIOR | 2 | 0.274 |
| QUALITY OF LIFE RESEARCH | 2 | 0.274 |
| REGIONAL ANESTHESIA AND PAIN MEDICINE | 2 | 0.274 |
| RESTORATIVE NEUROLOGY AND NEUROSCIENCE | 2 | 0.274 |
| STEM CELL RESEARCH THERAPY | 2 | 0.274 |
| STEM CELLS | 2 | 0.274 |
| ACTA ANAESTHESIOLOGICA SCANDINAVICA | 1 | 0.137 |
| ACTA BIOCHIMICA ET BIOPHYSICA SINICA | 1 | 0.137 |
| ACTA PHARMACOLOGICA SINICA | 1 | 0.137 |
| ACUPUNCTURE IN MEDICINE | 1 | 0.137 |
| ADVANCES IN EXPERIMENTAL MEDICINE AND BIOLOGY | 1 | 0.137 |
| ADVANCES IN PAIN RESEARCH AND THERAPY | 1 | 0.137 |
| AMERICAN JOURNAL OF NEURORADIOLOGY | 1 | 0.137 |
| AMERICAN JOURNAL OF SURGERY | 1 | 0.137 |
| ANAESTHESIA AND INTENSIVE CARE | 1 | 0.137 |
| ANESTHESIOLOGY | 1 | 0.137 |
| ANKARA UNIVERSITESI VETERINER FAKULTESI DERGISI | 1 | 0.137 |
| ANNALS OF SAUDI MEDICINE | 1 | 0.137 |
| ANNALS OF THE NEW YORK ACADEMY OF SCIENCES | 1 | 0.137 |
| AUSTRALIAN OCCUPATIONAL THERAPY JOURNAL | 1 | 0.137 |
| AUTONOMIC NEUROSCIENCE BASIC CLINICAL | 1 | 0.137 |
| BEHAVIOURAL BRAIN RESEARCH | 1 | 0.137 |
| BEHAVIOURAL PHARMACOLOGY | 1 | 0.137 |
| BIOCHIMICA ET BIOPHYSICA ACTA MOLECULAR BASIS OF DISEASE | 1 | 0.137 |
| BIOLOGICAL RESEARCH FOR NURSING | 1 | 0.137 |
| BIOMED RESEARCH INTERNATIONAL | 1 | 0.137 |
| BIOMEDICAL RESEARCH INDIA | 1 | 0.137 |
| BMJ OPEN | 1 | 0.137 |
| BRAIN BEHAVIOR AND IMMUNITY | 1 | 0.137 |
| CANADIAN JOURNAL OF PHYSIOLOGY AND PHARMACOLOGY | 1 | 0.137 |
| CANADIAN MEDICAL ASSOCIATION JOURNAL | 1 | 0.137 |
| CARDIOLOGY IN THE YOUNG | 1 | 0.137 |
| CELL STEM CELL | 1 | 0.137 |
| CELL TRANSPLANTATION | 1 | 0.137 |
| CEREBRAL CORTEX | 1 | 0.137 |
| CHINESE MEDICAL JOURNAL | 1 | 0.137 |
| CLINICAL NEUROLOGY AND NEUROSURGERY | 1 | 0.137 |
| CLINICAL PHARMACOLOGY IN DRUG DEVELOPMENT | 1 | 0.137 |
| CLINICAL THERAPEUTICS | 1 | 0.137 |
| CNS DRUGS | 1 | 0.137 |
| COCHRANE DATABASE OF SYSTEMATIC REVIEWS | 1 | 0.137 |
| COMPUTER METHODS AND PROGRAMS IN BIOMEDICINE | 1 | 0.137 |
| CURRENT OPINION IN NEUROLOGY | 1 | 0.137 |
| CURRENT PHARMACEUTICAL DESIGN | 1 | 0.137 |
| CURRENT PROTEIN PEPTIDE SCIENCE | 1 | 0.137 |
| DRUG DEVELOPMENT RESEARCH | 1 | 0.137 |
| DRUGS | 1 | 0.137 |
| ELECTROMAGNETIC BIOLOGY AND MEDICINE | 1 | 0.137 |
| ENDOCRINOLOGY | 1 | 0.137 |
| ERGONOMICS | 1 | 0.137 |
| EUROPEAN JOURNAL OF CLINICAL PHARMACOLOGY | 1 | 0.137 |
| EUROPEAN JOURNAL OF NEUROLOGY | 1 | 0.137 |
| EUROPEAN JOURNAL OF NEUROSCIENCE | 1 | 0.137 |
| EUROPEAN REVIEW FOR MEDICAL AND PHARMACOLOGICAL SCIENCES | 1 | 0.137 |
| EXPERIMENTAL BRAIN RESEARCH | 1 | 0.137 |
| EXPERT OPINION ON BIOLOGICAL THERAPY | 1 | 0.137 |
| EXPLORE THE JOURNAL OF SCIENCE AND HEALING | 1 | 0.137 |
| FRONTIERS IN CELLULAR NEUROSCIENCE | 1 | 0.137 |
| FRONTIERS IN HUMAN NEUROSCIENCE | 1 | 0.137 |
| FRONTIERS IN NEUROLOGY | 1 | 0.137 |
| FRONTIERS IN NEUROSCIENCE | 1 | 0.137 |
| FRONTIERS IN PHARMACOLOGY | 1 | 0.137 |
| GENE THERAPY | 1 | 0.137 |
| INTERNATIONAL JOURNAL OF INDUSTRIAL ERGONOMICS | 1 | 0.137 |
| INTERNATIONAL JOURNAL OF PHARMACOLOGY | 1 | 0.137 |
| JCPSP JOURNAL OF THE COLLEGE OF PHYSICIANS AND SURGEONS PAKISTAN | 1 | 0.137 |
| JOURNAL OF BIOLOGICAL CHEMISTRY | 1 | 0.137 |
| JOURNAL OF BIOMEDICINE AND BIOTECHNOLOGY | 1 | 0.137 |
| JOURNAL OF CHEMICAL NEUROANATOMY | 1 | 0.137 |
| JOURNAL OF CLINICAL ANESTHESIA | 1 | 0.137 |
| JOURNAL OF CLINICAL NEUROSCIENCE | 1 | 0.137 |
| JOURNAL OF COMPARATIVE NEUROLOGY | 1 | 0.137 |
| JOURNAL OF CONTROLLED RELEASE | 1 | 0.137 |
| JOURNAL OF ENDOCRINOLOGICAL INVESTIGATION | 1 | 0.137 |
| JOURNAL OF KOREAN NEUROSURGICAL SOCIETY | 1 | 0.137 |
| JOURNAL OF MOLECULAR NEUROSCIENCE | 1 | 0.137 |
| JOURNAL OF MUSCULOSKELETAL PAIN | 1 | 0.137 |
| JOURNAL OF NEUROENGINEERING AND REHABILITATION | 1 | 0.137 |
| JOURNAL OF NEUROIMMUNE PHARMACOLOGY | 1 | 0.137 |
| JOURNAL OF NEUROIMMUNOLOGY | 1 | 0.137 |
| JOURNAL OF NEUROLOGIC PHYSICAL THERAPY | 1 | 0.137 |
| JOURNAL OF NEUROPHYSIOLOGY | 1 | 0.137 |
| JOURNAL OF NEUROSCIENCE NURSING | 1 | 0.137 |
| JOURNAL OF NEUROSURGICAL SCIENCES | 1 | 0.137 |
| JOURNAL OF NURSING RESEARCH | 1 | 0.137 |
| JOURNAL OF ORTHOPAEDIC RESEARCH | 1 | 0.137 |
| JOURNAL OF PHARMACOLOGICAL SCIENCES | 1 | 0.137 |
| JOURNAL OF PHYSICAL THERAPY SCIENCE | 1 | 0.137 |
| JOURNAL OF TRANSLATIONAL MEDICINE | 1 | 0.137 |
| KOREAN JOURNAL OF PHYSIOLOGY PHARMACOLOGY | 1 | 0.137 |
| MEDICAL HYPOTHESES | 1 | 0.137 |
| MEDICAL SCIENCE MONITOR | 1 | 0.137 |
| MOLECULAR MEDICINE | 1 | 0.137 |
| MOLECULAR MEDICINE REPORTS | 1 | 0.137 |
| MOLECULAR NEUROBIOLOGY | 1 | 0.137 |
| MOLECULES AND CELLS | 1 | 0.137 |
| NEURAL PLASTICITY | 1 | 0.137 |
| NEUROCHEMICAL RESEARCH | 1 | 0.137 |
| NEUROIMAGE CLINICAL | 1 | 0.137 |
| NEUROLOGICAL RESEARCH | 1 | 0.137 |
| NEURON | 1 | 0.137 |
| NEURONS AND NETWORKS IN THE SPINAL CORD | 1 | 0.137 |
| NEUROPEPTIDES | 1 | 0.137 |
| NEUROPHYSIOLOGY | 1 | 0.137 |
| NEUROREHABILITATION | 1 | 0.137 |
| NEUROSIGNALS | 1 | 0.137 |
| NEUROSURGERY | 1 | 0.137 |
| NEUROTHERAPEUTICS | 1 | 0.137 |
| ONCOLOGY | 1 | 0.137 |
| ORTHOPEDICS | 1 | 0.137 |
| PAKISTAN JOURNAL OF MEDICAL SCIENCES | 1 | 0.137 |
| PHYSICAL MEDICINE AND REHABILITATION CLINICS OF NORTH AMERICA | 1 | 0.137 |
| PHYSIOLOGICAL RESEARCH | 1 | 0.137 |
| REGENERATIVE BIOLOGY OF THE SPINE AND SPINAL CORD | 1 | 0.137 |
| SEMINARS IN IMMUNOLOGY | 1 | 0.137 |
| STEREOTACTIC AND FUNCTIONAL NEUROSURGERY | 1 | 0.137 |
