# Supplementary material for: Bibliometric Study of Pain after Spinal Cord Injury
Source: Neural Plast. 2021 Feb 19;2021:6634644. doi: 10.1155/2021/6634644 (PMC7914384; doi:10.1155/2021/6634644)
Supplement: Supplementary 2 — Supplement Supplementary Table 1: raw data on journal sources of pain after spinal cord injury. Supplementary Table 2: raw data on countries/territories involved in pain after spinal cord injury. Supplementary Table 3: raw data on institutions involved in pain after spinal cord injury. [file 6634644.f2.zip › Supplementary Table 1.doc]

**Supplementary Table 1. Raw data on countries/territories involved in pain and spine core injury publications.**

| **Countries/Territories** | **Records** | **% of 3423** |
| --- | --- | --- |
| USA | 356 | 48.767 |
| AUSTRALIA | 53 | 7.26 |
| PEOPLES R CHINA | 52 | 7.123 |
| CANADA | 41 | 5.616 |
| DENMARK | 37 | 5.068 |
| JAPAN | 33 | 4.521 |
| SOUTH KOREA | 30 | 4.11 |
| SWEDEN | 30 | 4.11 |
| ENGLAND | 26 | 3.562 |
| GERMANY | 22 | 3.014 |
| SPAIN | 22 | 3.014 |
| SWITZERLAND | 20 | 2.74 |
| TURKEY | 19 | 2.603 |
| NETHERLANDS | 17 | 2.329 |
| IRAN | 16 | 2.192 |
| ITALY | 14 | 1.918 |
| ISRAEL | 11 | 1.507 |
| FRANCE | 9 | 1.233 |
| BRAZIL | 8 | 1.096 |
| NORWAY | 7 | 0.959 |
| SCOTLAND | 6 | 0.822 |
| INDIA | 5 | 0.685 |
| TAIWAN | 5 | 0.685 |
| IRELAND | 4 | 0.548 |
| PORTUGAL | 4 | 0.548 |
| ARGENTINA | 3 | 0.411 |
| AUSTRIA | 3 | 0.411 |
| IRAQ | 3 | 0.411 |
| PAKISTAN | 3 | 0.411 |
| BELGIUM | 2 | 0.274 |
| EGYPT | 2 | 0.274 |
| FINLAND | 2 | 0.274 |
| NEW ZEALAND | 2 | 0.274 |
| COLOMBIA | 1 | 0.137 |
| GREECE | 1 | 0.137 |
| PHILIPPINES | 1 | 0.137 |
| ROMANIA | 1 | 0.137 |
| RUSSIA | 1 | 0.137 |
| SERBIA | 1 | 0.137 |
| SINGAPORE | 1 | 0.137 |
| SLOVAKIA | 1 | 0.137 |
| THAILAND | 1 | 0.137 |
| USA | 356 | 48.767 |
| AUSTRALIA | 53 | 7.26 |
| PEOPLES R CHINA | 52 | 7.123 |
| CANADA | 41 | 5.616 |
| DENMARK | 37 | 5.068 |
| JAPAN | 33 | 4.521 |
| SOUTH KOREA | 30 | 4.11 |
| SWEDEN | 30 | 4.11 |
| ENGLAND | 26 | 3.562 |
| GERMANY | 22 | 3.014 |
| SPAIN | 22 | 3.014 |
| SWITZERLAND | 20 | 2.74 |
| TURKEY | 19 | 2.603 |
| NETHERLANDS | 17 | 2.329 |
| IRAN | 16 | 2.192 |
| ITALY | 14 | 1.918 |
| ISRAEL | 11 | 1.507 |
| FRANCE | 9 | 1.233 |
| BRAZIL | 8 | 1.096 |
| NORWAY | 7 | 0.959 |
| SCOTLAND | 6 | 0.822 |
| INDIA | 5 | 0.685 |
| TAIWAN | 5 | 0.685 |
| IRELAND | 4 | 0.548 |
| PORTUGAL | 4 | 0.548 |
| ARGENTINA | 3 | 0.411 |
| AUSTRIA | 3 | 0.411 |
| IRAQ | 3 | 0.411 |
| PAKISTAN | 3 | 0.411 |
