# Supplementary material for: Bibliometric Study of Pain after Spinal Cord Injury
Source: Neural Plast. 2021 Feb 19;2021:6634644. doi: 10.1155/2021/6634644 (PMC7914384; doi:10.1155/2021/6634644)
Supplement: Supplementary 2 — Supplement Supplementary Table 1: raw data on journal sources of pain after spinal cord injury. Supplementary Table 2: raw data on countries/territories involved in pain after spinal cord injury. Supplementary Table 3: raw data on institutions involved in pain after spinal cord injury. [file 6634644.f2.zip › Supplementary Table 2.doc]

**Supplementary Table 2. Raw data on institutions involved in pain and spine core injury publications.**

| **Institutions** | **records** | **% of 3423** |
| --- | --- | --- |
| UNIVERSITY OF MIAMI | 64 | 8.767 |
| UNIVERSITY OF WASHINGTON | 47 | 6.438 |
| UNIVERSITY OF WASHINGTON SEATTLE | 47 | 6.438 |
| UNIVERSITY OF TEXAS SYSTEM | 43 | 5.89 |
| UNIVERSITY OF SYDNEY | 42 | 5.753 |
| US DEPARTMENT OF VETERANS AFFAIRS | 40 | 5.479 |
| VETERANS HEALTH ADMINISTRATION VHA | 40 | 5.479 |
| ROYAL NORTH SHORE HOSPITAL | 38 | 5.205 |
| AARHUS UNIVERSITY | 35 | 4.795 |
| UNIVERSITY OF ALABAMA BIRMINGHAM | 29 | 3.973 |
| UNIVERSITY OF ALABAMA SYSTEM | 29 | 3.973 |
| KAROLINSKA INSTITUTET | 28 | 3.836 |
| UNIVERSITY OF TEXAS MEDICAL BRANCH GALVESTON | 25 | 3.425 |
| HARVARD UNIVERSITY | 22 | 3.014 |
| UNIVERSITY OF MICHIGAN | 18 | 2.466 |
| UNIVERSITY OF MICHIGAN SYSTEM | 18 | 2.466 |
| BAYLOR COLLEGE OF MEDICINE | 16 | 2.192 |
| DANDERYDS HOSPITAL | 16 | 2.192 |
| ICAHN SCHOOL OF MEDICINE AT MOUNT SINAI | 16 | 2.192 |
| KAROLINSKA UNIVERSITY HOSPITAL | 16 | 2.192 |
| UNIVERSITY OF CALIFORNIA SYSTEM | 16 | 2.192 |
| UNIVERSITY OF BRITISH COLUMBIA | 14 | 1.918 |
| UNIVERSITY OF COPENHAGEN | 13 | 1.781 |
| UNIVERSITY OF TEXAS HEALTH SCIENCE CENTER HOUSTON | 13 | 1.781 |
| RIGSHOSPITALET | 12 | 1.644 |
| STATE UNIVERSITY SYSTEM OF FLORIDA | 12 | 1.644 |
| UNIVERSITY COLLEGE LONDON | 12 | 1.644 |
| UNIVERSITY OF FLORIDA | 12 | 1.644 |
| UNIVERSITY OF LONDON | 12 | 1.644 |
| PFIZER | 11 | 1.507 |
| SEOUL NATIONAL UNIVERSITY SNU | 11 | 1.507 |
| TEL AVIV UNIVERSITY | 11 | 1.507 |
| YALE UNIVERSITY | 11 | 1.507 |
| KESSLER INSTITUTE FOR REHABILITATION | 10 | 1.37 |
| RUTGERS STATE UNIVERSITY MEDICAL CENTER | 10 | 1.37 |
| RUTGERS STATE UNIVERSITY NEW BRUNSWICK | 10 | 1.37 |
| AUTONOMOUS UNIVERSITY OF BARCELONA | 9 | 1.233 |
| UNIVERSITY OF MARYLAND BALTIMORE | 9 | 1.233 |
| UNIVERSITY OF TORONTO | 9 | 1.233 |
| UNIVERSITY SYSTEM OF MARYLAND | 9 | 1.233 |
| VA CONNECTICUT HEALTHCARE SYSTEM | 9 | 1.233 |
| WESTERN UNIVERSITY UNIVERSITY OF WESTERN ONTARIO | 9 | 1.233 |
| BETH ISRAEL DEACONESS MEDICAL CENTER | 8 | 1.096 |
| BRIGHAM WOMEN S HOSPITAL | 8 | 1.096 |
| JOHNS HOPKINS UNIVERSITY | 8 | 1.096 |
| NORTHWESTERN UNIVERSITY | 8 | 1.096 |
| PENNSYLVANIA COMMONWEALTH SYSTEM OF HIGHER EDUCATION PCSHE | 8 | 1.096 |
| SACKLER FACULTY OF MEDICINE | 8 | 1.096 |
| SWISS PARAPLEGIC RESEARCH | 8 | 1.096 |
| TEHRAN UNIVERSITY OF MEDICAL SCIENCES | 8 | 1.096 |
| TEXAS A M UNIVERSITY COLLEGE STATION | 8 | 1.096 |
| TEXAS A M UNIVERSITY SYSTEM | 8 | 1.096 |
| UNIVERSITY HEALTH NETWORK TORONTO | 8 | 1.096 |
| UNIVERSITY OF PITTSBURGH | 8 | 1.096 |
| UNIVERSITY OF ZURICH | 8 | 1.096 |
| IRAN UNIVERSITY OF MEDICAL SCIENCES | 7 | 0.959 |
| KOLLING INSTITUTE OF MEDICAL RESEARCH | 7 | 0.959 |
| LAVAL UNIVERSITY | 7 | 0.959 |
| MEDICAL UNIVERSITY OF SOUTH CAROLINA | 7 | 0.959 |
| SPAULDING REHABILITATION HOSPITAL | 7 | 0.959 |
| UNIVERSITY OF MINNESOTA SYSTEM | 7 | 0.959 |
| UNIVERSITY OF MINNESOTA TWIN CITIES | 7 | 0.959 |
| UTRECHT UNIVERSITY | 7 | 0.959 |
| UTRECHT UNIVERSITY MEDICAL CENTER | 7 | 0.959 |
| VAMC | 7 | 0.959 |
| ANKARA PHYSICAL THERAPY REHABILITATION HOSPITAL | 6 | 0.822 |
| CHAIM SHEBA MEDICAL CENTER | 6 | 0.822 |
| CRAIG HOSP | 6 | 0.822 |
| EAST CAROLINA UNIVERSITY | 6 | 0.822 |
| JEFFERSON UNIVERSITY | 6 | 0.822 |
| RUPRECHT KARLS UNIVERSITY HEIDELBERG | 6 | 0.822 |
| SHAHID BEHESHTI UNIVERSITY MEDICAL SCIENCES | 6 | 0.822 |
| UNIVERSITY OF KIEL | 6 | 0.822 |
| UNIVERSITY OF LUCERNE | 6 | 0.822 |
| UNIVERSITY OF NORTH CAROLINA | 6 | 0.822 |
| UNIVERSITY OF WISCONSIN MADISON | 6 | 0.822 |
| UNIVERSITY OF WISCONSIN SYSTEM | 6 | 0.822 |
| WAYNE STATE UNIVERSITY | 6 | 0.822 |
| WESTERN SYDNEY UNIVERSITY | 6 | 0.822 |
| BRUNEL UNIVERSITY | 5 | 0.685 |
| CAPITAL MEDICAL UNIVERSITY | 5 | 0.685 |
| CASE WESTERN RESERVE UNIVERSITY | 5 | 0.685 |
| CENTRE NATIONAL DE LA RECHERCHE SCIENTIFIQUE CNRS | 5 | 0.685 |
| CIBERNED | 5 | 0.685 |
| DEPT VET AFFAIRS MED CTR | 5 | 0.685 |
| FEINBERG SCHOOL OF MEDICINE | 5 | 0.685 |
| MAASTRICHT UNIVERSITY | 5 | 0.685 |
| REG HOSP VIBORG | 5 | 0.685 |
| SCHLESWIG HOLSTEIN UNIVERSITY HOSPITAL | 5 | 0.685 |
| UNIVERSIDADE DE SAO PAULO | 5 | 0.685 |
| UNIVERSITY OF CALIFORNIA IRVINE | 5 | 0.685 |
| UNIVERSITY OF CALIFORNIA SAN FRANCISCO | 5 | 0.685 |
| UNIVERSITY OF COLORADO SYSTEM | 5 | 0.685 |
| UNIVERSITY OF VERONA | 5 | 0.685 |
| VA PITTSBURGH HEALTHCARE SYSTEM | 5 | 0.685 |
| CATHOLIC UNIVERSITY OF KOREA | 4 | 0.548 |
| EDWARD HINES JR VA HOSPITAL | 4 | 0.548 |
| HARVARD MEDICAL SCHOOL | 4 | 0.548 |
| KOREA UNIVERSITY | 4 | 0.548 |
| MASSACHUSETTS GENERAL HOSPITAL | 4 | 0.548 |
| MAYO CLINIC | 4 | 0.548 |
| NATL REHABIL HOSP | 4 | 0.548 |
| ODENSE UNIVERSITY HOSPITAL | 4 | 0.548 |
| OSAKA UNIVERSITY | 4 | 0.548 |
| SHANDONG FIRST MEDICAL UNIVERSITY SHANDONG ACADEMY OF MEDICAL SCIENCES | 4 | 0.548 |
| SHIRLEY RYAN ABILITYLAB | 4 | 0.548 |
| STOKE MANDEVILLE HOSP | 4 | 0.548 |
| TIRR MEM HERMANN HOSP | 4 | 0.548 |
| UCL MEDICAL SCHOOL | 4 | 0.548 |
| UNIVERSIDAD DE CASTILLA LA MANCHA | 4 | 0.548 |
| UNIVERSITY COLLEGE DUBLIN | 4 | 0.548 |
| UNIVERSITY COLLEGE LONDON HOSPITALS NHS FOUNDATION TRUST | 4 | 0.548 |
| UNIVERSITY OF CALIFORNIA SAN DIEGO | 4 | 0.548 |
| UNIVERSITY OF GLASGOW | 4 | 0.548 |
| UNIVERSITY OF SOUTHERN DENMARK | 4 | 0.548 |
| UNIVERSITY OF TECHNOLOGY SYDNEY | 4 | 0.548 |
| ARMY MEDICAL UNIVERSITY | 3 | 0.411 |
| CHU LYON | 3 | 0.411 |
| CONSEJO NACIONAL DE INVESTIGACIONES CIENTIFICAS Y TECNICAS CONICET | 3 | 0.411 |
| DAEGU HAANY UNIVERSITY | 3 | 0.411 |
| DREXEL UNIVERSITY | 3 | 0.411 |
| EMORY UNIVERSITY | 3 | 0.411 |
| ETH ZURICH | 3 | 0.411 |
| GREENWICH HOSP | 3 | 0.411 |
| HAMMONDCARE | 3 | 0.411 |
| HOSP NACL PARAPLEJ | 3 | 0.411 |
| HYOGO COLLEGE OF MEDICINE | 3 | 0.411 |
| IMPERIAL COLLEGE LONDON | 3 | 0.411 |
| INST REHABIL RES | 3 | 0.411 |
| INSTITUT NATIONAL DE LA SANTE ET DE LA RECHERCHE MEDICALE INSERM | 3 | 0.411 |
| ISTANBUL PHYSICAL MEDICINE REHABILITATION TRAINING RESEARCH HOSPITAL | 3 | 0.411 |
| KOREA UNIVERSITY MEDICINE KU MEDICINE | 3 | 0.411 |
| KREMBIL RESEARCH INSTITUTE | 3 | 0.411 |
| KYUNG HEE UNIVERSITY | 3 | 0.411 |
| NANTONG UNIVERSITY | 3 | 0.411 |
| NATIONAL HOSPITAL NORWAY | 3 | 0.411 |
| OREGON HEALTH SCIENCE UNIVERSITY | 3 | 0.411 |
| PARACELSUS PRIVATE MEDICAL UNIVERSITY | 3 | 0.411 |
| PRINCE WALES MEDICAL RESEARCH INSTITUTE | 3 | 0.411 |
| QUEEN ELIZABETH UNIVERSITY HOSPITAL QEUH | 3 | 0.411 |
| QUEENS UNIVERSITY CANADA | 3 | 0.411 |
| REHABIL CTR DE HOOGSTRAAT | 3 | 0.411 |
| REHABIL INST MICHIGAN | 3 | 0.411 |
| ROYAL NATIONAL ORTHOPAEDIC HOSPITAL NHS TRUST | 3 | 0.411 |
| SALZBURG UNIVERSITY | 3 | 0.411 |
| SORBONNE UNIVERSITE | 3 | 0.411 |
| SPAIN REHABIL CTR | 3 | 0.411 |
| TOHOKU UNIVERSITY | 3 | 0.411 |
| TORONTO GENERAL HOSPITAL | 3 | 0.411 |
| UNIVERSIDADE DO MINHO | 3 | 0.411 |
| UNIVERSITY OF BARCELONA | 3 | 0.411 |
| UNIVERSITY OF BASEL | 3 | 0.411 |
| UNIVERSITY OF BERGEN | 3 | 0.411 |
| UNIVERSITY OF BUCKINGHAM | 3 | 0.411 |
| UNIVERSITY OF BUENOS AIRES | 3 | 0.411 |
| UNIVERSITY OF CINCINNATI | 3 | 0.411 |
| UNIVERSITY OF GENEVA | 3 | 0.411 |
| UNIVERSITY OF KENTUCKY | 3 | 0.411 |
| UNIVERSITY OF MISSOURI SYSTEM | 3 | 0.411 |
| UNIVERSITY OF NEW SOUTH WALES SYDNEY | 3 | 0.411 |
| UNIVERSITY OF OSLO | 3 | 0.411 |
| UNIVERSITY OF TEXAS DALLAS | 3 | 0.411 |
| UNIVERSITY OF VIRGINIA | 3 | 0.411 |
| UPPSALA UNIVERSITY | 3 | 0.411 |
| VA MED CTR | 3 | 0.411 |
| VET AFFAIRS PUGET SOUND HEALTH CARE SYSTEM | 3 | 0.411 |
| VRIJE UNIVERSITEIT AMSTERDAM | 3 | 0.411 |
| ZHENGZHOU UNIVERSITY | 3 | 0.411 |
| AALBORG UNIVERSITY | 2 | 0.274 |
| AIR FORCE MILITARY MEDICAL UNIVERSITY | 2 | 0.274 |
| AUSTIN RESEARCH INSTITUTE | 2 | 0.274 |
| BRUCE W CARTER VET AFFAIRS MED CTR | 2 | 0.274 |
| CENT HOSP TAIAN | 2 | 0.274 |
| CENTRAL SOUTH UNIVERSITY | 2 | 0.274 |
| CHIBA UNIVERSITY | 2 | 0.274 |
| CHINESE PLA ARMY GEN HOSP | 2 | 0.274 |
| CHUNGNAM NATIONAL UNIVERSITY | 2 | 0.274 |
| CLEVELAND FES CTR | 2 | 0.274 |
| CNRS NATIONAL INSTITUTE FOR BIOLOGY INSB | 2 | 0.274 |
| CONSEJO SUPERIOR DE INVESTIGACIONES CIENTIFICAS CSIC | 2 | 0.274 |
| CORNELL UNIVERSITY | 2 | 0.274 |
| CRAIG REHABIL HOSP | 2 | 0.274 |
| CTR COGNIT NEUROSCI | 2 | 0.274 |
| DALHOUSIE UNIVERSITY | 2 | 0.274 |
| DE HOOGSTR REHABIL | 2 | 0.274 |
| DUKE UNIVERSITY | 2 | 0.274 |
| F TAPPEINER HOSP | 2 | 0.274 |
| FUDAN UNIVERSITY | 2 | 0.274 |
| FUNDACIO INSTITUT D INVESTIGACIO EN CIENCIES DE LA SALUT GERMANS TRIAS I PUJOL IGTP | 2 | 0.274 |
| FUNING PEOPLES HOSP JIANGSU | 2 | 0.274 |
| GANNAN MEDICAL UNIVERSITY | 2 | 0.274 |
| HAMMEL NEUROREHABIL RES CTR | 2 | 0.274 |
| HONG KONG POLYTECHNIC UNIVERSITY | 2 | 0.274 |
| HOSPITAL CLINIC DE BARCELONA | 2 | 0.274 |
| HOSPITAL FOR JOINT DISEASE NYULMC | 2 | 0.274 |
| INDIANA UNIVERSITY BLOOMINGTON | 2 | 0.274 |
| INDIANA UNIVERSITY SYSTEM | 2 | 0.274 |
| INSTITUTE OF BIOLOGY EXPERIMENTAL MEDICINE | 2 | 0.274 |
| INT COLLABORAT REPAIR DISCOVERIES | 2 | 0.274 |
| IRCCS SANTA LUCIA | 2 | 0.274 |
| JILIN UNIVERSITY | 2 | 0.274 |
| KEIO UNIVERSITY | 2 | 0.274 |
| KENNEDY KRIEGER INSTITUTE | 2 | 0.274 |
| KYUSHU UNIVERSITY | 2 | 0.274 |
| LOUIS STOKES CLEVELAND VETERANS AFFAIRS MEDICAL CENTER | 2 | 0.274 |
| LOYOLA UNIVERSITY CHICAGO | 2 | 0.274 |
| MCGILL UNIVERSITY | 2 | 0.274 |
| MEDICAL COLLEGE OF WISCONSIN | 2 | 0.274 |
| MEDICAL PARK HOSPITALS GROUP | 2 | 0.274 |
| METROHEALTH SYSTEM | 2 | 0.274 |
| MICHAEL E DEBAKEY VA MEDICAL CENTER | 2 | 0.274 |
| NAGASAKI UNIVERSITY | 2 | 0.274 |
| NANJING MEDICAL UNIVERSITY | 2 | 0.274 |
| NANJING UNIVERSITY | 2 | 0.274 |
| NATIONAL DEFENSE MEDICAL CENTER | 2 | 0.274 |
| NATIONAL INSTITUTES OF HEALTH NIH USA | 2 | 0.274 |
| NATIONAL TAIWAN UNIVERSITY | 2 | 0.274 |
| NATL HOSP ORG | 2 | 0.274 |
| NETHERLANDS INSTITUTE FOR NEUROSCIENCE NIN KNAW | 2 | 0.274 |
| NEW YORK UNIVERSITY | 2 | 0.274 |
| NYU LANGONE MEDICAL CENTER | 2 | 0.274 |
| OHIO STATE UNIVERSITY | 2 | 0.274 |
| OSPEDALE FRANZ TAPPEINER | 2 | 0.274 |
| PARKWOOD HOSP | 2 | 0.274 |
| PARKWOOD INST | 2 | 0.274 |
| PINDERFIELDS HOSPITAL | 2 | 0.274 |
| PLA SECOND MILITARY MEDICAL UNIVERSITY | 2 | 0.274 |
| RIJNDAM REHABIL CTR | 2 | 0.274 |
| ROTHMAN INSTITUTE | 2 | 0.274 |
| ROYAL ADELAIDE HOSPITAL | 2 | 0.274 |
| ROYAL NETHERLANDS ACADEMY OF ARTS SCIENCES | 2 | 0.274 |
| ROYAL REHABIL CTR SYDNEY | 2 | 0.274 |
| RUSH UNIVERSITY | 2 | 0.274 |
| SALK INSTITUTE | 2 | 0.274 |
| SANTA CLARA VALLEY MEDICAL CENTER | 2 | 0.274 |
| SEATTLE UNIVERSITY | 2 | 0.274 |
| SELCUK UNIVERSITY | 2 | 0.274 |
| SEOUL NATIONAL UNIVERSITY HOSPITAL | 2 | 0.274 |
| SEOUL ST MARY S HOSPITAL | 2 | 0.274 |
| SHANDONG UNIVERSITY | 2 | 0.274 |
| SHENANDOAH UNIV | 2 | 0.274 |
| SHRINERS HOSP CHILDREN | 2 | 0.274 |
| ST JOSEPHS HEALTHCARE | 2 | 0.274 |
| TORONTO REHABILITATION INSTITUTE | 2 | 0.274 |
| TRI SERVICE GENERAL HOSPITAL | 2 | 0.274 |
| TRUVEN HLTH ANALYT | 2 | 0.274 |
| UNIV APPL SCI ARTS WESTERN SWITZERLAND HES SO | 2 | 0.274 |
| UNIVERSITAT DE GIRONA | 2 | 0.274 |
| UNIVERSITY OF ALBERTA | 2 | 0.274 |
| UNIVERSITY OF AMSTERDAM | 2 | 0.274 |
| UNIVERSITY OF CALIFORNIA LOS ANGELES | 2 | 0.274 |
| UNIVERSITY OF COLORADO ANSCHUTZ MEDICAL CAMPUS | 2 | 0.274 |
| UNIVERSITY OF COLORADO BOULDER | 2 | 0.274 |
| UNIVERSITY OF FUKUI | 2 | 0.274 |
| UNIVERSITY OF GRONINGEN | 2 | 0.274 |
| UNIVERSITY OF HAIFA | 2 | 0.274 |
| UNIVERSITY OF KANSAS | 2 | 0.274 |
| UNIVERSITY OF KANSAS MEDICAL CENTER | 2 | 0.274 |
| UNIVERSITY OF LIVERPOOL | 2 | 0.274 |
| UNIVERSITY OF MISSOURI KANSAS CITY | 2 | 0.274 |
| UNIVERSITY OF OXFORD | 2 | 0.274 |
| UNIVERSITY OF PAVIA | 2 | 0.274 |
| UNIVERSITY OF PENNSYLVANIA | 2 | 0.274 |
| UNIVERSITY OF SOUTHERN CALIFORNIA | 2 | 0.274 |
| UNIVERSITY OF TEXAS SOUTHWESTERN MEDICAL CENTER DALLAS | 2 | 0.274 |
| UPPSALA UNIVERSITY HOSPITAL | 2 | 0.274 |
| UTMD ANDERSON CANCER CENTER | 2 | 0.274 |
| VA ANN ARBOR HEALTHCARE SYSTEM | 2 | 0.274 |
| VA BOSTON HEALTHCARE SYSTEM | 2 | 0.274 |
| VA LONG BEACH HEALTHCARE SYSTEM | 2 | 0.274 |
| VA NORTH TEXAS HEALTH CARE SYSTEM | 2 | 0.274 |
| VIRGINIA COMMONWEALTH UNIVERSITY | 2 | 0.274 |
| VIRGINIA MASON MEDICAL CENTER | 2 | 0.274 |
| WASHINGTON STATE DEPT LABOR IND | 2 | 0.274 |
| WASIT UNIVERSITY | 2 | 0.274 |
| XI AN JIAOTONG UNIVERSITY | 2 | 0.274 |
| XINXIANG MEDICAL UNIVERSITY | 2 | 0.274 |
| YEUNGNAM UNIVERSITY | 2 | 0.274 |
| YONSEI UNIVERSITY | 2 | 0.274 |
| YONSEI UNIVERSITY HEALTH SYSTEM | 2 | 0.274 |
| 4D PHARMA RES LTD | 1 | 0.137 |
| 529C SPAIN REHABIL CTR | 1 | 0.137 |
| ABBVIE | 1 | 0.137 |
| ACAD TEACHING HOSP NORDSTADT | 1 | 0.137 |
| ACADEMIC MEDICAL CENTER AMSTERDAM | 1 | 0.137 |
| ACUPUNCTURE CTR | 1 | 0.137 |
| ADDENBROOKE S HOSPITAL | 1 | 0.137 |
| ADELANTE CTR EXPERTISE REHABIL AUDIOL | 1 | 0.137 |
| ADELANTE ZORGGROEP | 1 | 0.137 |
| ADELANTE ZORGGRP | 1 | 0.137 |
| ADIYAMAN UNIVERSITY | 1 | 0.137 |
| AGA KHAN UNIVERSITY | 1 | 0.137 |
| AIX MARSEILLE UNIVERSITE | 1 | 0.137 |
| AJAMED SCI UNIV | 1 | 0.137 |
| AJOU UNIVERSITY | 1 | 0.137 |
| ALESSANDRIA PUBL HOSP | 1 | 0.137 |
| ALEXANDRIA UNIVERSITY | 1 | 0.137 |
| ALL INDIA INSTITUTE OF MEDICAL SCIENCES AIIMS NEW DELHI | 1 | 0.137 |
| AMSTERDAM REHABIL RES CTR READE | 1 | 0.137 |
| AMSTERDAM REHABIL RES INST READE | 1 | 0.137 |
| ANKARA UNIVERSITY | 1 | 0.137 |
| ANN ARBOR VA MED CTR | 1 | 0.137 |
| ARISTOTLE UNIVERSITY OF THESSALONIKI | 1 | 0.137 |
| ASL 3 GENOVESE | 1 | 0.137 |
| ASSISTANCE PUBLIQUE HOPITAUX DE MARSEILLE | 1 | 0.137 |
| ASSISTANCE PUBLIQUE HOPITAUX PARIS APHP | 1 | 0.137 |
| ASTON UNIVERSITY | 1 | 0.137 |
| ASTRAZENECA | 1 | 0.137 |
| ATLANTIC HEALTH SYSTEM | 1 | 0.137 |
| AUCKLAND UNIVERSITY OF TECHNOLOGY | 1 | 0.137 |
| AUDIE L MURPHY MEMORIAL VETERANS HOSPITAL | 1 | 0.137 |
| AUSTRAL UNIVERSITY | 1 | 0.137 |
| AUSTRALIA COLL APPL PSYCHOL | 1 | 0.137 |
| AUSTRALIAN COLL APPL PSYCHOL | 1 | 0.137 |
| AUSTRALIAN NATIONAL UNIVERSITY | 1 | 0.137 |
| AUTONOMOUS UNIV | 1 | 0.137 |
| AVALON HLTH SOLUT INC | 1 | 0.137 |
| AZIENDA OSPEDALIERA DI PERUGIA | 1 | 0.137 |
| AZIENDA OSPEDALIERA UNIVERSITARIA INTEGRATA VERONA | 1 | 0.137 |
| AZIENDA OSPEDALIERO UNIVERSITARIA PISANA | 1 | 0.137 |
| BAMBERG HOSP | 1 | 0.137 |
| BAQIYATALLAH UNIVERSITY OF MEDICAL SCIENCES BMSU | 1 | 0.137 |
| BARTS HEALTH NHS TRUST | 1 | 0.137 |
| BASQUE CTR COGNIT BRAIN LANGUAGE | 1 | 0.137 |
| BAYSHORE MED CTR | 1 | 0.137 |
| BEIJING BOAI HOSP | 1 | 0.137 |
| BEIJING INST BRAIN DISORDERS | 1 | 0.137 |
| BEIJING KEY LAB NEURAL INJURY REHABIL | 1 | 0.137 |
| BEIJING NEUROSURGICAL INSTITUTE | 1 | 0.137 |
| BERUFSGENOSSENSCHAFTLICHE KLIN | 1 | 0.137 |
| BIEMAN COPLAND ASSOCIATES | 1 | 0.137 |
| BILL MELINDA GATES FOUNDATION | 1 | 0.137 |
| BLUSSON SPINAL CORD CTR | 1 | 0.137 |
| BOSTON CHILDREN S HOSPITAL | 1 | 0.137 |
| BOSTON UNIVERSITY | 1 | 0.137 |
| BROCK NIAGARA CTR HLTH WELL BEING | 1 | 0.137 |
| BROCK UNIVERSITY | 1 | 0.137 |
| BRUCE W CARTER DEPT VET AFFAIRS MED CTR | 1 | 0.137 |
| BUSHEHR UNIV MED SCI | 1 | 0.137 |
| C BESTA NEUROL INST FDN | 1 | 0.137 |
| CAGLIARI PUBL HOSP | 1 | 0.137 |
| CARING CHILDREN FDN | 1 | 0.137 |
| CAROLINAS REHABIL | 1 | 0.137 |
| CARROLL UNIV | 1 | 0.137 |
| CATALAN INSTITUTE OF ONCOLOGY | 1 | 0.137 |
| CATANIA PUBL HOSP | 1 | 0.137 |
| CENTRE HOSPITALIER UNIVERSITAIRE VAUDOIS CHUV | 1 | 0.137 |
| CHAPMAN UNIVERSITY | 1 | 0.137 |
| CHAPMAN UNIVERSITY SYSTEM | 1 | 0.137 |
| CHARLES STARK DRAPER LAB INC | 1 | 0.137 |
| CHEMOSERO THERAPEUT RES INST | 1 | 0.137 |
| CHEMPARTNER | 1 | 0.137 |
| CHILDREN S HOSPITAL COLORADO | 1 | 0.137 |
| CHILDRENS HOSP AUSTIN | 1 | 0.137 |
| CHINA THREE GORGES UNIVERSITY | 1 | 0.137 |
| CHINESE ACADEMY OF SCIENCES | 1 | 0.137 |
| CHINESE MED HOSP HULAN DIST | 1 | 0.137 |
| CHONGQING MEDICAL UNIVERSITY | 1 | 0.137 |
| CHONNAM NATIONAL UNIVERSITY | 1 | 0.137 |
| CHUNG ANG UNIVERSITY | 1 | 0.137 |
| CHUNGBUK NATIONAL UNIVERSITY | 1 | 0.137 |
| CHUNGBUK NATIONAL UNIVERSITY HOSPITAL | 1 | 0.137 |
| CITY COLLEGE OF NEW YORK CUNY | 1 | 0.137 |
| CITY UNIVERSITY OF NEW YORK CUNY SYSTEM | 1 | 0.137 |
| CLEMENT J ZABLOCKI VA MED CTR | 1 | 0.137 |
| CLEVELAND CLINIC FOUNDATION | 1 | 0.137 |
| CLIN ROMANDE READAPTAT | 1 | 0.137 |
| CLIN ROMANDE READAPTAT SUVACARE | 1 | 0.137 |
| COLL HLTH SCI | 1 | 0.137 |
| COLLEGE OF THE HOLY CROSS | 1 | 0.137 |
| COMMUNAUTE UNIVERSITE GRENOBLE ALPES | 1 | 0.137 |
| CONSORTIUM CTR BIOMED RES NETWORK NEURODEGENERAT | 1 | 0.137 |
| COVANCE | 1 | 0.137 |
| CRAWFORD RES INST | 1 | 0.137 |
| CRDR | 1 | 0.137 |
| CSIC INSTITUTO CAJAL IC | 1 | 0.137 |
| CTR INOVACAO ENSAIOS PRECLIN CIENP | 1 | 0.137 |
| CTR INTEGRAT MED INNOVAT TECHNOL | 1 | 0.137 |
| CTR INTERDISCIPLINAIRE RECH READAPTAT INTEGRAT | 1 | 0.137 |
| CTR INTERDISCIPLINARY RES REHABIL GREATER MONTREA | 1 | 0.137 |
| CTR NEUROSCI REGENERAT RES 127A | 1 | 0.137 |
| DA YEH UNIVERSITY | 1 | 0.137 |
| DAEGU HANNY UNIV | 1 | 0.137 |
| DALIAN UNIVERSITY | 1 | 0.137 |
| DARTMOUTH COLLEGE | 1 | 0.137 |
| DAVID GEFFEN SCHOOL OF MEDICINE AT UCLA | 1 | 0.137 |
| DE HOOGSTRAAT | 1 | 0.137 |
| DELFT UNIVERSITY OF TECHNOLOGY | 1 | 0.137 |
| DIV TRAUMA ACUTE CARE SURG | 1 | 0.137 |
| DOKKYO MEDICAL UNIVERSITY | 1 | 0.137 |
| EASTERN SCH ACUPUNCTURE | 1 | 0.137 |
| EBERHARD KARLS UNIVERSITY OF TUBINGEN | 1 | 0.137 |
| ECOLE POLYTECHNIQUE FEDERALE DE LAUSANNE | 1 | 0.137 |
| EGE UNIVERSITY | 1 | 0.137 |
| EHIME UNIVERSITY | 1 | 0.137 |
| ERASMUS MC | 1 | 0.137 |
| ERASMUS UNIVERSITY ROTTERDAM | 1 | 0.137 |
| ERCIYES UNIVERSITY | 1 | 0.137 |
| ESTEVE | 1 | 0.137 |
| ETERA MUTUAL PENS INSURANCE CO | 1 | 0.137 |
| EUROPEAN MULTICTR STUDY SPINAL CORD INJURY EMSCI | 1 | 0.137 |
| FAC EARTH LIFE SCI | 1 | 0.137 |
| FAC HLTH SCI | 1 | 0.137 |
| FDN ORTHOPED UNIV CLIN | 1 | 0.137 |
| FIRENZE UNIV HOSP | 1 | 0.137 |
| FLINDERS UNIVERSITY SOUTH AUSTRALIA | 1 | 0.137 |
| FLOREY INSTITUTE OF NEUROSCIENCE MENTAL HEALTH | 1 | 0.137 |
| FM KIRBY RES CTR FUNCT MRI | 1 | 0.137 |
| FMUSP | 1 | 0.137 |
| FOURTH PEOPLES HOSP SHAANXI | 1 | 0.137 |
| FROEDTERT MEM LUTHERAN HOSP | 1 | 0.137 |
| FROSUNDA CTR | 1 | 0.137 |
| FU JEN CATHOLIC UNIVERSITY | 1 | 0.137 |
| FUJIAN MEDICAL UNIVERSITY | 1 | 0.137 |
| FUKUOKA UNIVERSITY | 1 | 0.137 |
| FUKUSHIMA MEDICAL UNIVERSITY | 1 | 0.137 |
| GACHON UNIVERSITY | 1 | 0.137 |
| GAZI UNIVERSITY | 1 | 0.137 |
| GAZILER PHYSICAL THERAPY RESEARCH HOSPITAL | 1 | 0.137 |
| GEN HOSP CHINESE PEOPLES ARMED POLICE FORCES | 1 | 0.137 |
| GEORGE MASON UNIVERSITY | 1 | 0.137 |
| GERIATRIC RESEARCH EDUCATION CLINICAL CENTER | 1 | 0.137 |
| GLENROSE REHABIL HOSP | 1 | 0.137 |
| GLHANE MIL MED ACAD | 1 | 0.137 |
| GOETHE UNIVERSITY FRANKFURT | 1 | 0.137 |
| GOETHE UNIVERSITY FRANKFURT HOSPITAL | 1 | 0.137 |
| GOOD SAMARITAN HOSP | 1 | 0.137 |
| GUANGZHOU MEDICAL UNIVERSITY | 1 | 0.137 |
| GULHANE MILITARY MEDICAL ACADEMY | 1 | 0.137 |
| HACETTEPE UNIVERSITY | 1 | 0.137 |
| HAMMOND CARE | 1 | 0.137 |
| HANYANG UNIVERSITY | 1 | 0.137 |
| HARBIN MEDICAL UNIVERSITY | 1 | 0.137 |
| HARBORVIEW MEDICAL CENTER | 1 | 0.137 |
| HARVARD MED SCH | 1 | 0.137 |
| HELSINKI UNIVERSITY CENTRAL HOSPITAL | 1 | 0.137 |
| HENAN INST BIOL | 1 | 0.137 |
| HENAN UNIVERSITY OF TRADITIONAL CHINESE MEDICINE | 1 | 0.137 |
| HERMAN OSTROW SCH DENT | 1 | 0.137 |
| HIROSHIMA RED CROSS HOSP | 1 | 0.137 |
| HOFSTRA UNIVERSITY | 1 | 0.137 |
| HOKKAIDO CHUO ROSAI HOSP | 1 | 0.137 |
| HOLLYWOOD PRESBYTERIAN MED CTR | 1 | 0.137 |
| HOPITAL UNIVERSITAIRE BEAUJON APHP | 1 | 0.137 |
| HORIZON HLTH NETWORK | 1 | 0.137 |
| HOSHI UNIVERSITY | 1 | 0.137 |
| HOSP HOHE WARTE | 1 | 0.137 |
| HOSP NACL PARAPLEJICOS | 1 | 0.137 |
| HOSP NACL PARAPLEJICOS SESCAM | 1 | 0.137 |
| HOSP NEUROREHABILITACIO | 1 | 0.137 |
| HOSP SANTA MARIA | 1 | 0.137 |
| HOSPITAL PUERTA DE HIERRO MAJADAHONDA | 1 | 0.137 |
| HOWARD UNIVERSITY | 1 | 0.137 |
| HUANGSHI CENT HOSP | 1 | 0.137 |
| HUAZHONG UNIVERSITY OF SCIENCE TECHNOLOGY | 1 | 0.137 |
| HUMAN ENGN RES LABS | 1 | 0.137 |
| HYOGO UNIV HLTH SCI | 1 | 0.137 |
| ICORD | 1 | 0.137 |
| IDIBAPS | 1 | 0.137 |
| ILLINOIS STATE UNIVERSITY | 1 | 0.137 |
| INONU UNIVERSITY | 1 | 0.137 |
| INOVA FAIRFAX HOSPITAL | 1 | 0.137 |
| INOVA HEALTH SYSTEM | 1 | 0.137 |
| INST CANC ESTADO SAO PAULO | 1 | 0.137 |
| INST CLIN OUTCOMES RES | 1 | 0.137 |
| INST GUTTMAN | 1 | 0.137 |
| INST GUTTMANN | 1 | 0.137 |
| INST NEUROPATH PAIN | 1 | 0.137 |
| INST UNIV NEUROREHABILITACIO ADSCRIT UAB | 1 | 0.137 |
| INSTITUT D INVESTIGACIO SANITARIA PERE VIRGILI IISPV | 1 | 0.137 |
| INSTITUT NATIONAL POLYTECHNIQUE DE GRENOBLE | 1 | 0.137 |
| INSTITUTE OF METAL RESEARCH CAS | 1 | 0.137 |
| INT CLIN RES INST | 1 | 0.137 |
| INTERDISCIPLINARY CTR RES REHABIL SOCIAL INTEGR | 1 | 0.137 |
| IRCCS BAMBINO GESU | 1 | 0.137 |
| IRCCS CA GRANDA OSPEDALE MAGGIORE POLICLINICO | 1 | 0.137 |
| IRCCS CTR NEUROLESI MESSINA | 1 | 0.137 |
| IRCCS EUGENIO MEDEA | 1 | 0.137 |
| IRCCS FONDAZIONE CASIMIRO MONDINO | 1 | 0.137 |
| IRCCS FONDAZIONE DON CARLO GNOCCHI ONLUS | 1 | 0.137 |
| IRCCS FONDAZIONE SALVATORE MAUGERI | 1 | 0.137 |
| IRCCS ISTITUTO DELLE SCIENZE NEUROLOGICHE DI BOLOGNA ISNB | 1 | 0.137 |
| IRCCS ISTITUTO ORTOPEDICO RIZZOLI | 1 | 0.137 |
| IRONWOOD PHARMACEUT INC | 1 | 0.137 |
| IST SUPER OSTEOPATIA | 1 | 0.137 |
| ITHACA COLL | 1 | 0.137 |
| JACKSON MEM REHABIL CTR | 1 | 0.137 |
| JAMES A HALEY VETERANS HOSPITAL | 1 | 0.137 |
| JAPAN SOCIETY FOR THE PROMOTION OF SCIENCE | 1 | 0.137 |
| JAWAHARLAL NEHRU UNIVERSITY NEW DELHI | 1 | 0.137 |
| JERSEY MED SCH | 1 | 0.137 |
| JERUSALEM MENTAL HLTH CTR | 1 | 0.137 |
| JEWISH HOME HOSP | 1 | 0.137 |
| JIANGSU INST ANESTHESIOL | 1 | 0.137 |
| JIANGXI PROV KEY LAB AUTON NERVOUS FUNCT DIS | 1 | 0.137 |
| JINAN FIRST PEOPLES HOSP | 1 | 0.137 |
| JK STAT DATA CONSULTANCY | 1 | 0.137 |
| JMERC | 1 | 0.137 |
| JOHN CURTIN SCHOOL OF MEDICAL RESEARCH | 1 | 0.137 |
| JOHN D DINGELL VA MED CTR | 1 | 0.137 |
| JOHN H STROGER JUNIOR HOSPITAL COOK COUNTY | 1 | 0.137 |
| JOHNS HOPKINS MEDICINE | 1 | 0.137 |
| JOHNSON JOHNSON | 1 | 0.137 |
| JOHNSON JOHNSON USA | 1 | 0.137 |
| KAGOSHIMA UNIVERSITY | 1 | 0.137 |
| KEESLER INST REHABIL | 1 | 0.137 |
| KESSLER ADVENTIST REHABIL SERV | 1 | 0.137 |
| KHATAM OL ANBIA HOSP | 1 | 0.137 |
| KHON KAEN UNIVERSITY | 1 | 0.137 |
| KIO UNIV | 1 | 0.137 |
| KIRISHIMA ORTHOPED | 1 | 0.137 |
| KIRKLIN CLIN | 1 | 0.137 |
| KONYA NUMUNE HOSPITAL | 1 | 0.137 |
| KOREA INSTITUTE OF ORIENTAL MEDICINE KIOM | 1 | 0.137 |
| KOREA INSTITUTE OF SCIENCE TECHNOLOGY KIST | 1 | 0.137 |
| KUNMING MEDICAL UNIVERSITY | 1 | 0.137 |
| KURUME UNIVERSITY | 1 | 0.137 |
| KUSHIRO ROSAI HOSP | 1 | 0.137 |
| KYOTO UNIVERSITY | 1 | 0.137 |
| KYUNGPOOK NATIONAL UNIVERSITY | 1 | 0.137 |
| LAHEY HOSPITAL MEDICAL CENTER | 1 | 0.137 |
| LIVERPOOL JOHN MOORES UNIVERSITY | 1 | 0.137 |
| LOIS POPE LIFE CTR | 1 | 0.137 |
| LOMA LINDA UNIVERSITY | 1 | 0.137 |
| LUCERNE CANTONAL HOSPITAL | 1 | 0.137 |
| MACKAY MEDICAL COLLEGE | 1 | 0.137 |
| MACQUARIE UNIVERSITY | 1 | 0.137 |
| MAHARISHI MARKANDESHWAR UNIVERSITY | 1 | 0.137 |
| MARQUETTE UNIVERSITY | 1 | 0.137 |
| MARUISHI PHARMACEUT CO LTD | 1 | 0.137 |
| MAX PLANCK SOCIETY | 1 | 0.137 |
| MCMASTER UNIVERSITY | 1 | 0.137 |
| MED COLL TAISHAN | 1 | 0.137 |
| MED CTR | 1 | 0.137 |
| MED HOSP BEIBEI DIST | 1 | 0.137 |
| MEDICAL CENTER OF ALKMAAR | 1 | 0.137 |
| MEDSURANT LLC | 1 | 0.137 |
| MEDVAMC 145 | 1 | 0.137 |
| MEDVAMC 153 | 1 | 0.137 |
| MEHMET AKIF ERSOY UNIVERSITY | 1 | 0.137 |
| MERCK COMPANY | 1 | 0.137 |
| MIAMI DEPT VET AFFAIRS MED CTR | 1 | 0.137 |
| MIAMI MED CTR | 1 | 0.137 |
| MIAMI PROJECT CURE PARALYSIS | 1 | 0.137 |
| MIAMI PROJECT CURE PARALYSIS R 48 | 1 | 0.137 |
| MIAMI PROJECT CURE PARALYSIS R48 | 1 | 0.137 |
| MIAMI UNIV | 1 | 0.137 |
| MIL MED ACAD | 1 | 0.137 |
| MILLENNIUM PAIN CTR | 1 | 0.137 |
| MINISTRY OF HEALTH TURKEY | 1 | 0.137 |
| MINNEAPOLIS VA HEALTH CARE SYSTEM | 1 | 0.137 |
| MODEL SPINAL CORD INJURY CARE SYST SO CALIF | 1 | 0.137 |
| MONTECATONE INST NEUROREHABIL | 1 | 0.137 |
| MONTECATONE REHABIL INST | 1 | 0.137 |
| MOSSREHAB | 1 | 0.137 |
| MUDANJIANG MEDICAL UNIVERSITY | 1 | 0.137 |
| NANCHANG UNIVERSITY | 1 | 0.137 |
| NANTONG HOSP TRADIT CHINESE MED | 1 | 0.137 |
| NARA PREFECTURE GEN REHABIL CTR | 1 | 0.137 |
| NATIONAL INSTITUTE FOR PHYSIOLOGICAL SCIENCES NIPS | 1 | 0.137 |
| NATIONAL INSTITUTE OF HEALTH SCIENCES JAPAN | 1 | 0.137 |
| NATIONAL INSTITUTE OF MENTAL HEALTH NEUROSCIENCES INDIA | 1 | 0.137 |
| NATIONAL INSTITUTES OF NATURAL SCIENCES NINS JAPAN | 1 | 0.137 |
| NATIONAL TAIPEI UNIVERSITY OF NURSING HEALTH SCIENCE NTUNHS | 1 | 0.137 |
| NATIONAL TAIWAN UNIVERSITY HOSPITAL | 1 | 0.137 |
| NATIONAL UNIVERSITY OF SINGAPORE | 1 | 0.137 |
| NATL REHABIL CTR | 1 | 0.137 |
| NED UNIVERSITY OF ENGINEERING TECHNOLOGY | 1 | 0.137 |
| NEURONA THERAPEUT | 1 | 0.137 |
| NEUROSCIENCE RESEARCH AUSTRALIA | 1 | 0.137 |
| NEUROSEARCH AS | 1 | 0.137 |
| NEUROSURG DAMME | 1 | 0.137 |
| NICOX RES INST | 1 | 0.137 |
| NIGDE STATE HOSPITAL | 1 | 0.137 |
| NIH CLINICAL CENTER CC | 1 | 0.137 |
| NIH EUNICE KENNEDY SHRIVER NATIONAL INSTITUTE OF CHILD HEALTH HUMAN DEVELOPMENT NICHD | 1 | 0.137 |
| NIPPON MEDICAL SCHOOL | 1 | 0.137 |
| NORTHEASTERN UNIVERSITY | 1 | 0.137 |
| NORTHERN TECHNICAL UNIVERSITY | 1 | 0.137 |
| NORTHWELL HEALTH | 1 | 0.137 |
| NOVA TECH EEG | 1 | 0.137 |
| NSW AGCY CLIN INNOVAT | 1 | 0.137 |
| OHU UNIV | 1 | 0.137 |
| ORTHOPED CLIN | 1 | 0.137 |
| OSPEDALE NIGUARDA CA GRANDA | 1 | 0.137 |
| PALM BEACH NEUROL CTR | 1 | 0.137 |
| PARAPLEG CTR | 1 | 0.137 |
| PARC CIENT BARCELONA | 1 | 0.137 |
| PARKER UNIV | 1 | 0.137 |
| PATIENT PHYS PARTNERSHIP | 1 | 0.137 |
| PAVLOV INSTITUTE OF PHYSIOLOGY RUSSIAN ACADEMY OF SCIENCES | 1 | 0.137 |
| PFIZER JAPAN | 1 | 0.137 |
| PHARMERIT INT | 1 | 0.137 |
| PIETRA LIGURE PUBL HOSP | 1 | 0.137 |
| POLYTECH INST LEIRIA | 1 | 0.137 |
| PRINCE WALES HOSP | 1 | 0.137 |
| PURDUE UNIV NORTHWEST | 1 | 0.137 |
| PURDUE UNIVERSITY | 1 | 0.137 |
| PURDUE UNIVERSITY SYSTEM | 1 | 0.137 |
| PUSAN NATIONAL UNIVERSITY | 1 | 0.137 |
| PUSAN NATIONAL UNIVERSITY HOSPITAL | 1 | 0.137 |
| QIDONG PEOPLES HOSP | 1 | 0.137 |
| QINGDAO UNIVERSITY | 1 | 0.137 |
| QUEEN ELIZABETH II HEALTH SCIENCES CENTRE | 1 | 0.137 |
| QUEEN MARY UNIVERSITY LONDON | 1 | 0.137 |
| RABIN MEDICAL CENTER | 1 | 0.137 |
| RADBOUD UNIVERSITY NIJMEGEN | 1 | 0.137 |
| RANCHO LOS AMIGOS MED CTR | 1 | 0.137 |
| RANCHO LOS AMIGOS NATL REHABIL CTR | 1 | 0.137 |
| RANCHO LOS AMIGOS REHABIL CTR | 1 | 0.137 |
| RED CROSS HOSP | 1 | 0.137 |
| REHAB STN STOCKHOLM | 1 | 0.137 |
| REHAB STN STOCKHOLM SPINALIS | 1 | 0.137 |
| REHABIL CTR AMSTERDAM | 1 | 0.137 |
| REHABIL CTR HET ROESSINGH | 1 | 0.137 |
| REHABIL CTR HOOGSTR | 1 | 0.137 |
| REHABIL CTR ROESSINGH | 1 | 0.137 |
| REHABIL ORTON | 1 | 0.137 |
| REHMAN MED COLL | 1 | 0.137 |
| RICK HANSEN INST | 1 | 0.137 |
| RKU UNIV REHABIL KLINIKEN ULM | 1 | 0.137 |
| ROYAL LONDON HOSPITAL | 1 | 0.137 |
| ROYAL PERTH HOSPITAL | 1 | 0.137 |
| ROYAL REHAB CTR | 1 | 0.137 |
| ROYAL REHABIL CTR | 1 | 0.137 |
| ROYAL REHABILITAT CTR | 1 | 0.137 |
| RUHR UNIVERSITY BOCHUM | 1 | 0.137 |
| RUSSIAN ACADEMY OF SCIENCES | 1 | 0.137 |
| RUTGERS NEW JERSEY SCH MED | 1 | 0.137 |
| RUTGERS STATE UNIVERSITY NEWARK | 1 | 0.137 |
| SABBATSBERGS HOSP | 1 | 0.137 |
| SACRAMENTO VA MED CTR | 1 | 0.137 |
| SAMOT | 1 | 0.137 |
| SANJAY GANDHI POSTGRADUATE INSTITUTE OF MEDICAL SCIENCES | 1 | 0.137 |
| SAPIENZA UNIVERSITY ROME | 1 | 0.137 |
| SARAH NETWORK REHABIL HOSP | 1 | 0.137 |
| SCH ENGN | 1 | 0.137 |
| SCH LANGUAGE COMMUN DEV | 1 | 0.137 |
| SCHULTHESS CLINIC | 1 | 0.137 |
| SCHWARZ BIOSCI | 1 | 0.137 |
| SCRIPPS RESEARCH INSTITUTE | 1 | 0.137 |
| SCUOLA SUPERIORE SANT ANNA | 1 | 0.137 |
| SENBOKU KUMIAI GEN HOSP | 1 | 0.137 |
| SESCAM | 1 | 0.137 |
| SEVKET YILMAZ TRAINING RESEARCH HOSPITAL | 1 | 0.137 |
| SHANDONG JINING 1 PEOPLES HOSP | 1 | 0.137 |
| SHANGHAI GONGLI HOSP | 1 | 0.137 |
| SHANGHAI JIAO TONG UNIVERSITY | 1 | 0.137 |
| SHANGHAI KAIYUAN ORTHOPED HOSP | 1 | 0.137 |
| SHANGHAI UNIVERSITY OF SPORT | 1 | 0.137 |
| SHIN KONG WU HO MEM HOSP | 1 | 0.137 |
| SICHUAN PROVINCIAL PEOPLE S HOSPITAL | 1 | 0.137 |
| SICHUAN UNIVERSITY | 1 | 0.137 |
| SIXTH MEDICAL CENTER OF CHINESE PLA GENERAL HOSPITAL | 1 | 0.137 |
| SLOTERVAART HOSPITAL | 1 | 0.137 |
| SLOVAK ACADEMY OF SCIENCES | 1 | 0.137 |
| SMS MEDICAL COLLEGE HOSPITAL | 1 | 0.137 |
| SOGANG UNIVERSITY | 1 | 0.137 |
| SONDALO PUBL HOSP | 1 | 0.137 |
| SOTERIX MED | 1 | 0.137 |
| SOUTH LONDON MAUDSLEY NHS TRUST | 1 | 0.137 |
| SPAULDING NEW ENGLAND REG SCI CTR | 1 | 0.137 |
| SPINALIS SCI UNIT | 1 | 0.137 |
| SRC REHABIL HOSP | 1 | 0.137 |
| ST JOSEPHS PARKWOOD HOSP | 1 | 0.137 |
| ST LUKES REHABIL INST | 1 | 0.137 |
| ST PETERSBURG SCIENTIFIC CENTRE OF THE RUSSIAN ACADEMY OF SCIENCES | 1 | 0.137 |
| STANFORD UNIVERSITY | 1 | 0.137 |
| STATE UNIVERSITY OF NEW YORK SUNY BUFFALO | 1 | 0.137 |
| STATE UNIVERSITY OF NEW YORK SUNY SYSTEM | 1 | 0.137 |
| STOCKHOLM UNIVERSITY | 1 | 0.137 |
| STOKE MANDEVILLE HOSP NHS TRUST | 1 | 0.137 |
| SUN YAT SEN UNIVERSITY | 1 | 0.137 |
| SUNNAAS REHABIL HOSP | 1 | 0.137 |
| SUVACARE | 1 | 0.137 |
| SW COLL NATUROPATH MED | 1 | 0.137 |
| SWEDISH SCHOOL OF SPORT HEALTH SCIENCES | 1 | 0.137 |
| SYRACUSE DVAMC | 1 | 0.137 |
| TAIZHOU PEOPLES HOSP | 1 | 0.137 |
| TANTA UNIVERSITY | 1 | 0.137 |
| TEIKYO UNIVERSITY | 1 | 0.137 |
| TEXAS A M HEALTH SCIENCE CENTER | 1 | 0.137 |
| TEXAS INST REHABIL RES | 1 | 0.137 |
| TEXAS STATE UNIVERSITY SAN MARCOS | 1 | 0.137 |
| TEXAS STATE UNIVERSITY SYSTEM | 1 | 0.137 |
| TEXAS WOMANS UNIVERSITY | 1 | 0.137 |
| TIRR | 1 | 0.137 |
| TIRR MEM HERMANN | 1 | 0.137 |
| TOHO UNIVERSITY | 1 | 0.137 |
| TOKYO MEDICAL DENTAL UNIVERSITY TMDU | 1 | 0.137 |
| TOKYO WOMEN S MEDICAL UNIVERSITY | 1 | 0.137 |
| TORONTO CONGENITAL CARDIAC CENTRE FOR ADULTS | 1 | 0.137 |
| TULANE UNIVERSITY | 1 | 0.137 |
| TURAN EMEKSIZ SOKAK | 1 | 0.137 |
| TURKISH ARMED FORCES REHABILITATION MAINTENANCE CENTER | 1 | 0.137 |
| UBC SCI SOLUT | 1 | 0.137 |
| UBON RATCHATHANI UNIVERSITY | 1 | 0.137 |
| ULM UNIVERSITY | 1 | 0.137 |
| ULUDAG UNIVERSITY | 1 | 0.137 |
| UMEA UNIVERSITY | 1 | 0.137 |
| UNIBRASIL | 1 | 0.137 |
| UNITED STATES ARMY | 1 | 0.137 |
| UNITED STATES DEPARTMENT OF DEFENSE | 1 | 0.137 |
| UNIV MED SCI | 1 | 0.137 |
| UNIVERSIDAD DE ANTIOQUIA | 1 | 0.137 |
| UNIVERSIDAD DE MALAGA | 1 | 0.137 |
| UNIVERSIDAD REY JUAN CARLOS | 1 | 0.137 |
| UNIVERSIDADE CIDADE DE SAO PAULO | 1 | 0.137 |
| UNIVERSIDADE FEDERAL DE SANTA CATARINA UFSC | 1 | 0.137 |
| UNIVERSIDADE FEDERAL DO CEARA | 1 | 0.137 |
| UNIVERSIDADE PORTUCALENSE INFANTE D HENRIQUE | 1 | 0.137 |
| UNIVERSIDADE PRESBITERIANA MACKENZIE | 1 | 0.137 |
| UNIVERSITAT ROVIRA I VIRGILI | 1 | 0.137 |
| UNIVERSITE DE PARIS | 1 | 0.137 |
| UNIVERSITE DE STRASBOURG | 1 | 0.137 |
| UNIVERSITE GRENOBLE ALPES UGA | 1 | 0.137 |
| UNIVERSITES DE STRASBOURG ETABLISSEMENTS ASSOCIES | 1 | 0.137 |
| UNIVERSITY HOSPITAL OF BERN | 1 | 0.137 |
| UNIVERSITY OF ABERDEEN | 1 | 0.137 |
| UNIVERSITY OF ADELAIDE | 1 | 0.137 |
| UNIVERSITY OF ANTWERP | 1 | 0.137 |
| UNIVERSITY OF BERN | 1 | 0.137 |
| UNIVERSITY OF BOLOGNA | 1 | 0.137 |
| UNIVERSITY OF CALIFORNIA DAVIS | 1 | 0.137 |
| UNIVERSITY OF CAMBRIDGE | 1 | 0.137 |
| UNIVERSITY OF COLORADO DENVER | 1 | 0.137 |
| UNIVERSITY OF DELAWARE | 1 | 0.137 |
| UNIVERSITY OF DUNDEE | 1 | 0.137 |
| UNIVERSITY OF GENOA | 1 | 0.137 |
| UNIVERSITY OF GOTTINGEN | 1 | 0.137 |
| UNIVERSITY OF HELSINKI | 1 | 0.137 |
| UNIVERSITY OF HONG KONG | 1 | 0.137 |
| UNIVERSITY OF ILLINOIS CHICAGO | 1 | 0.137 |
| UNIVERSITY OF ILLINOIS CHICAGO HOSPITAL | 1 | 0.137 |
| UNIVERSITY OF ILLINOIS SYSTEM | 1 | 0.137 |
| UNIVERSITY OF JINAN | 1 | 0.137 |
| UNIVERSITY OF LAUSANNE | 1 | 0.137 |
| UNIVERSITY OF LIEGE | 1 | 0.137 |
| UNIVERSITY OF LOUISVILLE | 1 | 0.137 |
| UNIVERSITY OF MILAN | 1 | 0.137 |
| UNIVERSITY OF MISSOURI COLUMBIA | 1 | 0.137 |
| UNIVERSITY OF MONTREAL | 1 | 0.137 |
| UNIVERSITY OF NOTTINGHAM | 1 | 0.137 |
| UNIVERSITY OF OTAGO | 1 | 0.137 |
| UNIVERSITY OF OTTAWA | 1 | 0.137 |
| UNIVERSITY OF PERUGIA | 1 | 0.137 |
| UNIVERSITY OF PISA | 1 | 0.137 |
| UNIVERSITY OF PUERTO RICO | 1 | 0.137 |
| UNIVERSITY OF PUERTO RICO MEDICAL SCIENCES CAMPUS | 1 | 0.137 |
| UNIVERSITY OF ROCHESTER | 1 | 0.137 |
| UNIVERSITY OF SANTO TOMAS | 1 | 0.137 |
| UNIVERSITY OF SHERBROOKE | 1 | 0.137 |
| UNIVERSITY OF SOUTH AUSTRALIA | 1 | 0.137 |
| UNIVERSITY OF SOUTH CHINA | 1 | 0.137 |
| UNIVERSITY OF SOUTHAMPTON | 1 | 0.137 |
| UNIVERSITY OF STRATHCLYDE | 1 | 0.137 |
| UNIVERSITY OF TEHRAN | 1 | 0.137 |
| UNIVERSITY OF TEXAS AUSTIN | 1 | 0.137 |
| UNIVERSITY OF TEXAS HEALTH SAN ANTONIO | 1 | 0.137 |
| UNIVERSITY OF TOKYO | 1 | 0.137 |
| UNIVERSITY OF TURKU | 1 | 0.137 |
| UNIVERSITY OF UTAH | 1 | 0.137 |
| UNIVERSITY OF WATERLOO | 1 | 0.137 |
| UNIVERSITY OF WESTERN AUSTRALIA | 1 | 0.137 |
| UNIVERSITY SYSTEM OF GEORGIA | 1 | 0.137 |
| US ARMY | 1 | 0.137 |
| UT HLTH | 1 | 0.137 |
| UTAH SYSTEM OF HIGHER EDUCATION | 1 | 0.137 |
| VA LOMA LINDA HEALTHCARE SYST | 1 | 0.137 |
| VA PALO ALTO HEALTH CARE SYSTEM | 1 | 0.137 |
| VALDOSTA STATE UNIVERSITY | 1 | 0.137 |
| VANDERBILT UNIVERSITY | 1 | 0.137 |
| VET ADM | 1 | 0.137 |
| VET ADM MED CTR W ROXBURY | 1 | 0.137 |
| VICTOR BABES UNIVERSITY OF MEDICINE PHARMACY TIMISOARA | 1 | 0.137 |
| VIRGINAIA CONNECTICUT HEALTHCARE SYST | 1 | 0.137 |
| VIRGINIA CONNECTICUT HEALTHCARE SYST | 1 | 0.137 |
| VU UNIVERSITY MEDICAL CENTER | 1 | 0.137 |
| WAKAYAMA MEDICAL UNIVERSITY | 1 | 0.137 |
| WAKE FOREST BAPTIST MEDICAL CENTER | 1 | 0.137 |
| WAKE FOREST UNIVERSITY | 1 | 0.137 |
| WALTER REED NATIONAL MILITARY MEDICAL CENTER | 1 | 0.137 |
| WALTON CENTRE | 1 | 0.137 |
| WASHINGTON STATE UNIVERSITY | 1 | 0.137 |
| WERNER WICKER CLIN | 1 | 0.137 |
| WESTMEAD INSTITUTE FOR MEDICAL RESEARCH | 1 | 0.137 |
| WILLIAM PATERSON UNIV | 1 | 0.137 |
| WOODROW WILSON REHABIL CTR | 1 | 0.137 |
| WUHAN UNIVERSITY | 1 | 0.137 |
| XUZHOU MEDICAL COLLEGE | 1 | 0.137 |
| YAMAGUCHI UNIVERSITY | 1 | 0.137 |
| YORK UNIVERSITY CANADA | 1 | 0.137 |
| ZHENGZHOU ORTHOPAED HOSP | 1 | 0.137 |
| ZONMW | 1 | 0.137 |
| ZUCKER SCH MED HOFSTRA NORTHWELL | 1 | 0.137 |
| UNIVERSITY OF MIAMI | 64 | 8.767 |
| UNIVERSITY OF WASHINGTON | 47 | 6.438 |
| UNIVERSITY OF WASHINGTON SEATTLE | 47 | 6.438 |
| UNIVERSITY OF TEXAS SYSTEM | 43 | 5.89 |
| UNIVERSITY OF SYDNEY | 42 | 5.753 |
| US DEPARTMENT OF VETERANS AFFAIRS | 40 | 5.479 |
| VETERANS HEALTH ADMINISTRATION VHA | 40 | 5.479 |
| ROYAL NORTH SHORE HOSPITAL | 38 | 5.205 |
| AARHUS UNIVERSITY | 35 | 4.795 |
| UNIVERSITY OF ALABAMA BIRMINGHAM | 29 | 3.973 |
| UNIVERSITY OF ALABAMA SYSTEM | 29 | 3.973 |
| KAROLINSKA INSTITUTET | 28 | 3.836 |
| UNIVERSITY OF TEXAS MEDICAL BRANCH GALVESTON | 25 | 3.425 |
| HARVARD UNIVERSITY | 22 | 3.014 |
| UNIVERSITY OF MICHIGAN | 18 | 2.466 |
| UNIVERSITY OF MICHIGAN SYSTEM | 18 | 2.466 |
| BAYLOR COLLEGE OF MEDICINE | 16 | 2.192 |
| DANDERYDS HOSPITAL | 16 | 2.192 |
| ICAHN SCHOOL OF MEDICINE AT MOUNT SINAI | 16 | 2.192 |
| KAROLINSKA UNIVERSITY HOSPITAL | 16 | 2.192 |
| UNIVERSITY OF CALIFORNIA SYSTEM | 16 | 2.192 |
| UNIVERSITY OF BRITISH COLUMBIA | 14 | 1.918 |
| UNIVERSITY OF COPENHAGEN | 13 | 1.781 |
| UNIVERSITY OF TEXAS HEALTH SCIENCE CENTER HOUSTON | 13 | 1.781 |
| RIGSHOSPITALET | 12 | 1.644 |
| STATE UNIVERSITY SYSTEM OF FLORIDA | 12 | 1.644 |
| UNIVERSITY COLLEGE LONDON | 12 | 1.644 |
| UNIVERSITY OF FLORIDA | 12 | 1.644 |
| UNIVERSITY OF LONDON | 12 | 1.644 |
| PFIZER | 11 | 1.507 |
| SEOUL NATIONAL UNIVERSITY SNU | 11 | 1.507 |
| TEL AVIV UNIVERSITY | 11 | 1.507 |
| YALE UNIVERSITY | 11 | 1.507 |
| KESSLER INSTITUTE FOR REHABILITATION | 10 | 1.37 |
| RUTGERS STATE UNIVERSITY MEDICAL CENTER | 10 | 1.37 |
| RUTGERS STATE UNIVERSITY NEW BRUNSWICK | 10 | 1.37 |
| AUTONOMOUS UNIVERSITY OF BARCELONA | 9 | 1.233 |
| UNIVERSITY OF MARYLAND BALTIMORE | 9 | 1.233 |
| UNIVERSITY OF TORONTO | 9 | 1.233 |
| UNIVERSITY SYSTEM OF MARYLAND | 9 | 1.233 |
| VA CONNECTICUT HEALTHCARE SYSTEM | 9 | 1.233 |
| WESTERN UNIVERSITY UNIVERSITY OF WESTERN ONTARIO | 9 | 1.233 |
| BETH ISRAEL DEACONESS MEDICAL CENTER | 8 | 1.096 |
| BRIGHAM WOMEN S HOSPITAL | 8 | 1.096 |
| JOHNS HOPKINS UNIVERSITY | 8 | 1.096 |
| NORTHWESTERN UNIVERSITY | 8 | 1.096 |
| PENNSYLVANIA COMMONWEALTH SYSTEM OF HIGHER EDUCATION PCSHE | 8 | 1.096 |
| SACKLER FACULTY OF MEDICINE | 8 | 1.096 |
| SWISS PARAPLEGIC RESEARCH | 8 | 1.096 |
| TEHRAN UNIVERSITY OF MEDICAL SCIENCES | 8 | 1.096 |
| TEXAS A M UNIVERSITY COLLEGE STATION | 8 | 1.096 |
| TEXAS A M UNIVERSITY SYSTEM | 8 | 1.096 |
| UNIVERSITY HEALTH NETWORK TORONTO | 8 | 1.096 |
| UNIVERSITY OF PITTSBURGH | 8 | 1.096 |
| UNIVERSITY OF ZURICH | 8 | 1.096 |
| IRAN UNIVERSITY OF MEDICAL SCIENCES | 7 | 0.959 |
| KOLLING INSTITUTE OF MEDICAL RESEARCH | 7 | 0.959 |
| LAVAL UNIVERSITY | 7 | 0.959 |
| MEDICAL UNIVERSITY OF SOUTH CAROLINA | 7 | 0.959 |
| SPAULDING REHABILITATION HOSPITAL | 7 | 0.959 |
| UNIVERSITY OF MINNESOTA SYSTEM | 7 | 0.959 |
| UNIVERSITY OF MINNESOTA TWIN CITIES | 7 | 0.959 |
| UTRECHT UNIVERSITY | 7 | 0.959 |
| UTRECHT UNIVERSITY MEDICAL CENTER | 7 | 0.959 |
| VAMC | 7 | 0.959 |
| ANKARA PHYSICAL THERAPY REHABILITATION HOSPITAL | 6 | 0.822 |
| CHAIM SHEBA MEDICAL CENTER | 6 | 0.822 |
| CRAIG HOSP | 6 | 0.822 |
| EAST CAROLINA UNIVERSITY | 6 | 0.822 |
| JEFFERSON UNIVERSITY | 6 | 0.822 |
| RUPRECHT KARLS UNIVERSITY HEIDELBERG | 6 | 0.822 |
| SHAHID BEHESHTI UNIVERSITY MEDICAL SCIENCES | 6 | 0.822 |
| UNIVERSITY OF KIEL | 6 | 0.822 |
| UNIVERSITY OF LUCERNE | 6 | 0.822 |
| UNIVERSITY OF NORTH CAROLINA | 6 | 0.822 |
| UNIVERSITY OF WISCONSIN MADISON | 6 | 0.822 |
| UNIVERSITY OF WISCONSIN SYSTEM | 6 | 0.822 |
| WAYNE STATE UNIVERSITY | 6 | 0.822 |
| WESTERN SYDNEY UNIVERSITY | 6 | 0.822 |
| BRUNEL UNIVERSITY | 5 | 0.685 |
| CAPITAL MEDICAL UNIVERSITY | 5 | 0.685 |
| CASE WESTERN RESERVE UNIVERSITY | 5 | 0.685 |
| CENTRE NATIONAL DE LA RECHERCHE SCIENTIFIQUE CNRS | 5 | 0.685 |
| CIBERNED | 5 | 0.685 |
| DEPT VET AFFAIRS MED CTR | 5 | 0.685 |
| FEINBERG SCHOOL OF MEDICINE | 5 | 0.685 |
| MAASTRICHT UNIVERSITY | 5 | 0.685 |
| REG HOSP VIBORG | 5 | 0.685 |
| SCHLESWIG HOLSTEIN UNIVERSITY HOSPITAL | 5 | 0.685 |
| UNIVERSIDADE DE SAO PAULO | 5 | 0.685 |
| UNIVERSITY OF CALIFORNIA IRVINE | 5 | 0.685 |
| UNIVERSITY OF CALIFORNIA SAN FRANCISCO | 5 | 0.685 |
| UNIVERSITY OF COLORADO SYSTEM | 5 | 0.685 |
| UNIVERSITY OF VERONA | 5 | 0.685 |
| VA PITTSBURGH HEALTHCARE SYSTEM | 5 | 0.685 |
| CATHOLIC UNIVERSITY OF KOREA | 4 | 0.548 |
| EDWARD HINES JR VA HOSPITAL | 4 | 0.548 |
| HARVARD MEDICAL SCHOOL | 4 | 0.548 |
| KOREA UNIVERSITY | 4 | 0.548 |
| MASSACHUSETTS GENERAL HOSPITAL | 4 | 0.548 |
| MAYO CLINIC | 4 | 0.548 |
| NATL REHABIL HOSP | 4 | 0.548 |
| ODENSE UNIVERSITY HOSPITAL | 4 | 0.548 |
| OSAKA UNIVERSITY | 4 | 0.548 |
| SHANDONG FIRST MEDICAL UNIVERSITY SHANDONG ACADEMY OF MEDICAL SCIENCES | 4 | 0.548 |
| SHIRLEY RYAN ABILITYLAB | 4 | 0.548 |
| STOKE MANDEVILLE HOSP | 4 | 0.548 |
| TIRR MEM HERMANN HOSP | 4 | 0.548 |
| UCL MEDICAL SCHOOL | 4 | 0.548 |
| UNIVERSIDAD DE CASTILLA LA MANCHA | 4 | 0.548 |
| UNIVERSITY COLLEGE DUBLIN | 4 | 0.548 |
| UNIVERSITY COLLEGE LONDON HOSPITALS NHS FOUNDATION TRUST | 4 | 0.548 |
| UNIVERSITY OF CALIFORNIA SAN DIEGO | 4 | 0.548 |
| UNIVERSITY OF GLASGOW | 4 | 0.548 |
| UNIVERSITY OF SOUTHERN DENMARK | 4 | 0.548 |
| UNIVERSITY OF TECHNOLOGY SYDNEY | 4 | 0.548 |
| ARMY MEDICAL UNIVERSITY | 3 | 0.411 |
| CHU LYON | 3 | 0.411 |
| CONSEJO NACIONAL DE INVESTIGACIONES CIENTIFICAS Y TECNICAS CONICET | 3 | 0.411 |
| DAEGU HAANY UNIVERSITY | 3 | 0.411 |
| DREXEL UNIVERSITY | 3 | 0.411 |
| EMORY UNIVERSITY | 3 | 0.411 |
| ETH ZURICH | 3 | 0.411 |
| GREENWICH HOSP | 3 | 0.411 |
| HAMMONDCARE | 3 | 0.411 |
| HOSP NACL PARAPLEJ | 3 | 0.411 |
| HYOGO COLLEGE OF MEDICINE | 3 | 0.411 |
| IMPERIAL COLLEGE LONDON | 3 | 0.411 |
| INST REHABIL RES | 3 | 0.411 |
| INSTITUT NATIONAL DE LA SANTE ET DE LA RECHERCHE MEDICALE INSERM | 3 | 0.411 |
| ISTANBUL PHYSICAL MEDICINE REHABILITATION TRAINING RESEARCH HOSPITAL | 3 | 0.411 |
| KOREA UNIVERSITY MEDICINE KU MEDICINE | 3 | 0.411 |
| KREMBIL RESEARCH INSTITUTE | 3 | 0.411 |
| KYUNG HEE UNIVERSITY | 3 | 0.411 |
| NANTONG UNIVERSITY | 3 | 0.411 |
| NATIONAL HOSPITAL NORWAY | 3 | 0.411 |
| OREGON HEALTH SCIENCE UNIVERSITY | 3 | 0.411 |
| PARACELSUS PRIVATE MEDICAL UNIVERSITY | 3 | 0.411 |
| PRINCE WALES MEDICAL RESEARCH INSTITUTE | 3 | 0.411 |
| QUEEN ELIZABETH UNIVERSITY HOSPITAL QEUH | 3 | 0.411 |
| QUEENS UNIVERSITY CANADA | 3 | 0.411 |
| REHABIL CTR DE HOOGSTRAAT | 3 | 0.411 |
| REHABIL INST MICHIGAN | 3 | 0.411 |
| ROYAL NATIONAL ORTHOPAEDIC HOSPITAL NHS TRUST | 3 | 0.411 |
| SALZBURG UNIVERSITY | 3 | 0.411 |
| SORBONNE UNIVERSITE | 3 | 0.411 |
| SPAIN REHABIL CTR | 3 | 0.411 |
| TOHOKU UNIVERSITY | 3 | 0.411 |
| TORONTO GENERAL HOSPITAL | 3 | 0.411 |
| UNIVERSIDADE DO MINHO | 3 | 0.411 |
| UNIVERSITY OF BARCELONA | 3 | 0.411 |
| UNIVERSITY OF BASEL | 3 | 0.411 |
| UNIVERSITY OF BERGEN | 3 | 0.411 |
| UNIVERSITY OF BUCKINGHAM | 3 | 0.411 |
| UNIVERSITY OF BUENOS AIRES | 3 | 0.411 |
| UNIVERSITY OF CINCINNATI | 3 | 0.411 |
| UNIVERSITY OF GENEVA | 3 | 0.411 |
| UNIVERSITY OF KENTUCKY | 3 | 0.411 |
| UNIVERSITY OF MISSOURI SYSTEM | 3 | 0.411 |
| UNIVERSITY OF NEW SOUTH WALES SYDNEY | 3 | 0.411 |
| UNIVERSITY OF OSLO | 3 | 0.411 |
| UNIVERSITY OF TEXAS DALLAS | 3 | 0.411 |
| UNIVERSITY OF VIRGINIA | 3 | 0.411 |
| UPPSALA UNIVERSITY | 3 | 0.411 |
| VA MED CTR | 3 | 0.411 |
| VET AFFAIRS PUGET SOUND HEALTH CARE SYSTEM | 3 | 0.411 |
| VRIJE UNIVERSITEIT AMSTERDAM | 3 | 0.411 |
| ZHENGZHOU UNIVERSITY | 3 | 0.411 |
| AALBORG UNIVERSITY | 2 | 0.274 |
| AIR FORCE MILITARY MEDICAL UNIVERSITY | 2 | 0.274 |
| AUSTIN RESEARCH INSTITUTE | 2 | 0.274 |
| BRUCE W CARTER VET AFFAIRS MED CTR | 2 | 0.274 |
| CENT HOSP TAIAN | 2 | 0.274 |
| CENTRAL SOUTH UNIVERSITY | 2 | 0.274 |
| CHIBA UNIVERSITY | 2 | 0.274 |
| CHINESE PLA ARMY GEN HOSP | 2 | 0.274 |
| CHUNGNAM NATIONAL UNIVERSITY | 2 | 0.274 |
| CLEVELAND FES CTR | 2 | 0.274 |
| CNRS NATIONAL INSTITUTE FOR BIOLOGY INSB | 2 | 0.274 |
| CONSEJO SUPERIOR DE INVESTIGACIONES CIENTIFICAS CSIC | 2 | 0.274 |
| CORNELL UNIVERSITY | 2 | 0.274 |
| CRAIG REHABIL HOSP | 2 | 0.274 |
| CTR COGNIT NEUROSCI | 2 | 0.274 |
| DALHOUSIE UNIVERSITY | 2 | 0.274 |
| DE HOOGSTR REHABIL | 2 | 0.274 |
| DUKE UNIVERSITY | 2 | 0.274 |
| F TAPPEINER HOSP | 2 | 0.274 |
| FUDAN UNIVERSITY | 2 | 0.274 |
| FUNDACIO INSTITUT D INVESTIGACIO EN CIENCIES DE LA SALUT GERMANS TRIAS I PUJOL IGTP | 2 | 0.274 |
| FUNING PEOPLES HOSP JIANGSU | 2 | 0.274 |
| GANNAN MEDICAL UNIVERSITY | 2 | 0.274 |
| HAMMEL NEUROREHABIL RES CTR | 2 | 0.274 |
| HONG KONG POLYTECHNIC UNIVERSITY | 2 | 0.274 |
| HOSPITAL CLINIC DE BARCELONA | 2 | 0.274 |
| HOSPITAL FOR JOINT DISEASE NYULMC | 2 | 0.274 |
| INDIANA UNIVERSITY BLOOMINGTON | 2 | 0.274 |
| INDIANA UNIVERSITY SYSTEM | 2 | 0.274 |
| INSTITUTE OF BIOLOGY EXPERIMENTAL MEDICINE | 2 | 0.274 |
| INT COLLABORAT REPAIR DISCOVERIES | 2 | 0.274 |
| IRCCS SANTA LUCIA | 2 | 0.274 |
| JILIN UNIVERSITY | 2 | 0.274 |
| KEIO UNIVERSITY | 2 | 0.274 |
| KENNEDY KRIEGER INSTITUTE | 2 | 0.274 |
| KYUSHU UNIVERSITY | 2 | 0.274 |
| LOUIS STOKES CLEVELAND VETERANS AFFAIRS MEDICAL CENTER | 2 | 0.274 |
| LOYOLA UNIVERSITY CHICAGO | 2 | 0.274 |
| MCGILL UNIVERSITY | 2 | 0.274 |
| MEDICAL COLLEGE OF WISCONSIN | 2 | 0.274 |
| MEDICAL PARK HOSPITALS GROUP | 2 | 0.274 |
| METROHEALTH SYSTEM | 2 | 0.274 |
| MICHAEL E DEBAKEY VA MEDICAL CENTER | 2 | 0.274 |
| NAGASAKI UNIVERSITY | 2 | 0.274 |
| NANJING MEDICAL UNIVERSITY | 2 | 0.274 |
| NANJING UNIVERSITY | 2 | 0.274 |
| NATIONAL DEFENSE MEDICAL CENTER | 2 | 0.274 |
| NATIONAL INSTITUTES OF HEALTH NIH USA | 2 | 0.274 |
| NATIONAL TAIWAN UNIVERSITY | 2 | 0.274 |
| NATL HOSP ORG | 2 | 0.274 |
| NETHERLANDS INSTITUTE FOR NEUROSCIENCE NIN KNAW | 2 | 0.274 |
| NEW YORK UNIVERSITY | 2 | 0.274 |
| NYU LANGONE MEDICAL CENTER | 2 | 0.274 |
| OHIO STATE UNIVERSITY | 2 | 0.274 |
| OSPEDALE FRANZ TAPPEINER | 2 | 0.274 |
| PARKWOOD HOSP | 2 | 0.274 |
| PARKWOOD INST | 2 | 0.274 |
| PINDERFIELDS HOSPITAL | 2 | 0.274 |
| PLA SECOND MILITARY MEDICAL UNIVERSITY | 2 | 0.274 |
| RIJNDAM REHABIL CTR | 2 | 0.274 |
| ROTHMAN INSTITUTE | 2 | 0.274 |
| ROYAL ADELAIDE HOSPITAL | 2 | 0.274 |
| ROYAL NETHERLANDS ACADEMY OF ARTS SCIENCES | 2 | 0.274 |
| ROYAL REHABIL CTR SYDNEY | 2 | 0.274 |
| RUSH UNIVERSITY | 2 | 0.274 |
| SALK INSTITUTE | 2 | 0.274 |
| SANTA CLARA VALLEY MEDICAL CENTER | 2 | 0.274 |
| SEATTLE UNIVERSITY | 2 | 0.274 |
| SELCUK UNIVERSITY | 2 | 0.274 |
| SEOUL NATIONAL UNIVERSITY HOSPITAL | 2 | 0.274 |
| SEOUL ST MARY S HOSPITAL | 2 | 0.274 |
| SHANDONG UNIVERSITY | 2 | 0.274 |
| SHENANDOAH UNIV | 2 | 0.274 |
| SHRINERS HOSP CHILDREN | 2 | 0.274 |
| ST JOSEPHS HEALTHCARE | 2 | 0.274 |
| TORONTO REHABILITATION INSTITUTE | 2 | 0.274 |
| TRI SERVICE GENERAL HOSPITAL | 2 | 0.274 |
| TRUVEN HLTH ANALYT | 2 | 0.274 |
| UNIV APPL SCI ARTS WESTERN SWITZERLAND HES SO | 2 | 0.274 |
| UNIVERSITAT DE GIRONA | 2 | 0.274 |
| UNIVERSITY OF ALBERTA | 2 | 0.274 |
| UNIVERSITY OF AMSTERDAM | 2 | 0.274 |
| UNIVERSITY OF CALIFORNIA LOS ANGELES | 2 | 0.274 |
| UNIVERSITY OF COLORADO ANSCHUTZ MEDICAL CAMPUS | 2 | 0.274 |
| UNIVERSITY OF COLORADO BOULDER | 2 | 0.274 |
| UNIVERSITY OF FUKUI | 2 | 0.274 |
| UNIVERSITY OF GRONINGEN | 2 | 0.274 |
| UNIVERSITY OF HAIFA | 2 | 0.274 |
| UNIVERSITY OF KANSAS | 2 | 0.274 |
| UNIVERSITY OF KANSAS MEDICAL CENTER | 2 | 0.274 |
| UNIVERSITY OF LIVERPOOL | 2 | 0.274 |
| UNIVERSITY OF MISSOURI KANSAS CITY | 2 | 0.274 |
| UNIVERSITY OF OXFORD | 2 | 0.274 |
| UNIVERSITY OF PAVIA | 2 | 0.274 |
| UNIVERSITY OF PENNSYLVANIA | 2 | 0.274 |
| UNIVERSITY OF SOUTHERN CALIFORNIA | 2 | 0.274 |
| UNIVERSITY OF TEXAS SOUTHWESTERN MEDICAL CENTER DALLAS | 2 | 0.274 |
| UPPSALA UNIVERSITY HOSPITAL | 2 | 0.274 |
| UTMD ANDERSON CANCER CENTER | 2 | 0.274 |
| VA ANN ARBOR HEALTHCARE SYSTEM | 2 | 0.274 |
| VA BOSTON HEALTHCARE SYSTEM | 2 | 0.274 |
| VA LONG BEACH HEALTHCARE SYSTEM | 2 | 0.274 |
| VA NORTH TEXAS HEALTH CARE SYSTEM | 2 | 0.274 |
| VIRGINIA COMMONWEALTH UNIVERSITY | 2 | 0.274 |
| VIRGINIA MASON MEDICAL CENTER | 2 | 0.274 |
| WASHINGTON STATE DEPT LABOR IND | 2 | 0.274 |
| WASIT UNIVERSITY | 2 | 0.274 |
| XI AN JIAOTONG UNIVERSITY | 2 | 0.274 |
| XINXIANG MEDICAL UNIVERSITY | 2 | 0.274 |
| YEUNGNAM UNIVERSITY | 2 | 0.274 |
| YONSEI UNIVERSITY | 2 | 0.274 |
| YONSEI UNIVERSITY HEALTH SYSTEM | 2 | 0.274 |
| 4D PHARMA RES LTD | 1 | 0.137 |
| 529C SPAIN REHABIL CTR | 1 | 0.137 |
| ABBVIE | 1 | 0.137 |
| ACAD TEACHING HOSP NORDSTADT | 1 | 0.137 |
| ACADEMIC MEDICAL CENTER AMSTERDAM | 1 | 0.137 |
| ACUPUNCTURE CTR | 1 | 0.137 |
| ADDENBROOKE S HOSPITAL | 1 | 0.137 |
| ADELANTE CTR EXPERTISE REHABIL AUDIOL | 1 | 0.137 |
| ADELANTE ZORGGROEP | 1 | 0.137 |
| ADELANTE ZORGGRP | 1 | 0.137 |
| ADIYAMAN UNIVERSITY | 1 | 0.137 |
| AGA KHAN UNIVERSITY | 1 | 0.137 |
| AIX MARSEILLE UNIVERSITE | 1 | 0.137 |
| AJAMED SCI UNIV | 1 | 0.137 |
| AJOU UNIVERSITY | 1 | 0.137 |
| ALESSANDRIA PUBL HOSP | 1 | 0.137 |
| ALEXANDRIA UNIVERSITY | 1 | 0.137 |
| ALL INDIA INSTITUTE OF MEDICAL SCIENCES AIIMS NEW DELHI | 1 | 0.137 |
| AMSTERDAM REHABIL RES CTR READE | 1 | 0.137 |
| AMSTERDAM REHABIL RES INST READE | 1 | 0.137 |
| ANKARA UNIVERSITY | 1 | 0.137 |
| ANN ARBOR VA MED CTR | 1 | 0.137 |
| ARISTOTLE UNIVERSITY OF THESSALONIKI | 1 | 0.137 |
| ASL 3 GENOVESE | 1 | 0.137 |
| ASSISTANCE PUBLIQUE HOPITAUX DE MARSEILLE | 1 | 0.137 |
| ASSISTANCE PUBLIQUE HOPITAUX PARIS APHP | 1 | 0.137 |
| ASTON UNIVERSITY | 1 | 0.137 |
| ASTRAZENECA | 1 | 0.137 |
| ATLANTIC HEALTH SYSTEM | 1 | 0.137 |
| AUCKLAND UNIVERSITY OF TECHNOLOGY | 1 | 0.137 |
| AUDIE L MURPHY MEMORIAL VETERANS HOSPITAL | 1 | 0.137 |
| AUSTRAL UNIVERSITY | 1 | 0.137 |
| AUSTRALIA COLL APPL PSYCHOL | 1 | 0.137 |
| AUSTRALIAN COLL APPL PSYCHOL | 1 | 0.137 |
| AUSTRALIAN NATIONAL UNIVERSITY | 1 | 0.137 |
| AUTONOMOUS UNIV | 1 | 0.137 |
| AVALON HLTH SOLUT INC | 1 | 0.137 |
| AZIENDA OSPEDALIERA DI PERUGIA | 1 | 0.137 |
| AZIENDA OSPEDALIERA UNIVERSITARIA INTEGRATA VERONA | 1 | 0.137 |
| AZIENDA OSPEDALIERO UNIVERSITARIA PISANA | 1 | 0.137 |
| BAMBERG HOSP | 1 | 0.137 |
| BAQIYATALLAH UNIVERSITY OF MEDICAL SCIENCES BMSU | 1 | 0.137 |
| BARTS HEALTH NHS TRUST | 1 | 0.137 |
| BASQUE CTR COGNIT BRAIN LANGUAGE | 1 | 0.137 |
| BAYSHORE MED CTR | 1 | 0.137 |
| BEIJING BOAI HOSP | 1 | 0.137 |
| BEIJING INST BRAIN DISORDERS | 1 | 0.137 |
| BEIJING KEY LAB NEURAL INJURY REHABIL | 1 | 0.137 |
| BEIJING NEUROSURGICAL INSTITUTE | 1 | 0.137 |
| BERUFSGENOSSENSCHAFTLICHE KLIN | 1 | 0.137 |
| BIEMAN COPLAND ASSOCIATES | 1 | 0.137 |
| BILL MELINDA GATES FOUNDATION | 1 | 0.137 |
| BLUSSON SPINAL CORD CTR | 1 | 0.137 |
| BOSTON CHILDREN S HOSPITAL | 1 | 0.137 |
| BOSTON UNIVERSITY | 1 | 0.137 |
| BROCK NIAGARA CTR HLTH WELL BEING | 1 | 0.137 |
| BROCK UNIVERSITY | 1 | 0.137 |
| BRUCE W CARTER DEPT VET AFFAIRS MED CTR | 1 | 0.137 |
| BUSHEHR UNIV MED SCI | 1 | 0.137 |
| C BESTA NEUROL INST FDN | 1 | 0.137 |
| CAGLIARI PUBL HOSP | 1 | 0.137 |
| CARING CHILDREN FDN | 1 | 0.137 |
| CAROLINAS REHABIL | 1 | 0.137 |
| CARROLL UNIV | 1 | 0.137 |
| CATALAN INSTITUTE OF ONCOLOGY | 1 | 0.137 |
| CATANIA PUBL HOSP | 1 | 0.137 |
| CENTRE HOSPITALIER UNIVERSITAIRE VAUDOIS CHUV | 1 | 0.137 |
| CHAPMAN UNIVERSITY | 1 | 0.137 |
| CHAPMAN UNIVERSITY SYSTEM | 1 | 0.137 |
| CHARLES STARK DRAPER LAB INC | 1 | 0.137 |
| CHEMOSERO THERAPEUT RES INST | 1 | 0.137 |
| CHEMPARTNER | 1 | 0.137 |
| CHILDREN S HOSPITAL COLORADO | 1 | 0.137 |
| CHILDRENS HOSP AUSTIN | 1 | 0.137 |
| CHINA THREE GORGES UNIVERSITY | 1 | 0.137 |
| CHINESE ACADEMY OF SCIENCES | 1 | 0.137 |
| CHINESE MED HOSP HULAN DIST | 1 | 0.137 |
| CHONGQING MEDICAL UNIVERSITY | 1 | 0.137 |
| CHONNAM NATIONAL UNIVERSITY | 1 | 0.137 |
| CHUNG ANG UNIVERSITY | 1 | 0.137 |
| CHUNGBUK NATIONAL UNIVERSITY | 1 | 0.137 |
| CHUNGBUK NATIONAL UNIVERSITY HOSPITAL | 1 | 0.137 |
| CITY COLLEGE OF NEW YORK CUNY | 1 | 0.137 |
| CITY UNIVERSITY OF NEW YORK CUNY SYSTEM | 1 | 0.137 |
| CLEMENT J ZABLOCKI VA MED CTR | 1 | 0.137 |
| CLEVELAND CLINIC FOUNDATION | 1 | 0.137 |
| CLIN ROMANDE READAPTAT | 1 | 0.137 |
| CLIN ROMANDE READAPTAT SUVACARE | 1 | 0.137 |
| COLL HLTH SCI | 1 | 0.137 |
| COLLEGE OF THE HOLY CROSS | 1 | 0.137 |
| COMMUNAUTE UNIVERSITE GRENOBLE ALPES | 1 | 0.137 |
| CONSORTIUM CTR BIOMED RES NETWORK NEURODEGENERAT | 1 | 0.137 |
| COVANCE | 1 | 0.137 |
| CRAWFORD RES INST | 1 | 0.137 |
| CRDR | 1 | 0.137 |
| CSIC INSTITUTO CAJAL IC | 1 | 0.137 |
| CTR INOVACAO ENSAIOS PRECLIN CIENP | 1 | 0.137 |
| CTR INTEGRAT MED INNOVAT TECHNOL | 1 | 0.137 |
| CTR INTERDISCIPLINAIRE RECH READAPTAT INTEGRAT | 1 | 0.137 |
| CTR INTERDISCIPLINARY RES REHABIL GREATER MONTREA | 1 | 0.137 |
| CTR NEUROSCI REGENERAT RES 127A | 1 | 0.137 |
| DA YEH UNIVERSITY | 1 | 0.137 |
| DAEGU HANNY UNIV | 1 | 0.137 |
| DALIAN UNIVERSITY | 1 | 0.137 |
| DARTMOUTH COLLEGE | 1 | 0.137 |
| DAVID GEFFEN SCHOOL OF MEDICINE AT UCLA | 1 | 0.137 |
| DE HOOGSTRAAT | 1 | 0.137 |
| DELFT UNIVERSITY OF TECHNOLOGY | 1 | 0.137 |
| DIV TRAUMA ACUTE CARE SURG | 1 | 0.137 |
| DOKKYO MEDICAL UNIVERSITY | 1 | 0.137 |
| EASTERN SCH ACUPUNCTURE | 1 | 0.137 |
| EBERHARD KARLS UNIVERSITY OF TUBINGEN | 1 | 0.137 |
| ECOLE POLYTECHNIQUE FEDERALE DE LAUSANNE | 1 | 0.137 |
| EGE UNIVERSITY | 1 | 0.137 |
| EHIME UNIVERSITY | 1 | 0.137 |
| ERASMUS MC | 1 | 0.137 |
| ERASMUS UNIVERSITY ROTTERDAM | 1 | 0.137 |
| ERCIYES UNIVERSITY | 1 | 0.137 |
| ESTEVE | 1 | 0.137 |
| ETERA MUTUAL PENS INSURANCE CO | 1 | 0.137 |
| EUROPEAN MULTICTR STUDY SPINAL CORD INJURY EMSCI | 1 | 0.137 |
| FAC EARTH LIFE SCI | 1 | 0.137 |
| FAC HLTH SCI | 1 | 0.137 |
| FDN ORTHOPED UNIV CLIN | 1 | 0.137 |
| FIRENZE UNIV HOSP | 1 | 0.137 |
| FLINDERS UNIVERSITY SOUTH AUSTRALIA | 1 | 0.137 |
| FLOREY INSTITUTE OF NEUROSCIENCE MENTAL HEALTH | 1 | 0.137 |
| FM KIRBY RES CTR FUNCT MRI | 1 | 0.137 |
| FMUSP | 1 | 0.137 |
| FOURTH PEOPLES HOSP SHAANXI | 1 | 0.137 |
| FROEDTERT MEM LUTHERAN HOSP | 1 | 0.137 |
| FROSUNDA CTR | 1 | 0.137 |
| FU JEN CATHOLIC UNIVERSITY | 1 | 0.137 |
| FUJIAN MEDICAL UNIVERSITY | 1 | 0.137 |
| FUKUOKA UNIVERSITY | 1 | 0.137 |
| FUKUSHIMA MEDICAL UNIVERSITY | 1 | 0.137 |
| GACHON UNIVERSITY | 1 | 0.137 |
| GAZI UNIVERSITY | 1 | 0.137 |
| GAZILER PHYSICAL THERAPY RESEARCH HOSPITAL | 1 | 0.137 |
| GEN HOSP CHINESE PEOPLES ARMED POLICE FORCES | 1 | 0.137 |
| GEORGE MASON UNIVERSITY | 1 | 0.137 |
| GERIATRIC RESEARCH EDUCATION CLINICAL CENTER | 1 | 0.137 |
| GLENROSE REHABIL HOSP | 1 | 0.137 |
| GLHANE MIL MED ACAD | 1 | 0.137 |
| GOETHE UNIVERSITY FRANKFURT | 1 | 0.137 |
| GOETHE UNIVERSITY FRANKFURT HOSPITAL | 1 | 0.137 |
| GOOD SAMARITAN HOSP | 1 | 0.137 |
| GUANGZHOU MEDICAL UNIVERSITY | 1 | 0.137 |
| GULHANE MILITARY MEDICAL ACADEMY | 1 | 0.137 |
| HACETTEPE UNIVERSITY | 1 | 0.137 |
| HAMMOND CARE | 1 | 0.137 |
| HANYANG UNIVERSITY | 1 | 0.137 |
| HARBIN MEDICAL UNIVERSITY | 1 | 0.137 |
| HARBORVIEW MEDICAL CENTER | 1 | 0.137 |
| HARVARD MED SCH | 1 | 0.137 |
| HELSINKI UNIVERSITY CENTRAL HOSPITAL | 1 | 0.137 |
| HENAN INST BIOL | 1 | 0.137 |
| HENAN UNIVERSITY OF TRADITIONAL CHINESE MEDICINE | 1 | 0.137 |
| HERMAN OSTROW SCH DENT | 1 | 0.137 |
| HIROSHIMA RED CROSS HOSP | 1 | 0.137 |
| HOFSTRA UNIVERSITY | 1 | 0.137 |
| HOKKAIDO CHUO ROSAI HOSP | 1 | 0.137 |
| HOLLYWOOD PRESBYTERIAN MED CTR | 1 | 0.137 |
| HOPITAL UNIVERSITAIRE BEAUJON APHP | 1 | 0.137 |
| HORIZON HLTH NETWORK | 1 | 0.137 |
| HOSHI UNIVERSITY | 1 | 0.137 |
| HOSP HOHE WARTE | 1 | 0.137 |
| HOSP NACL PARAPLEJICOS | 1 | 0.137 |
| HOSP NACL PARAPLEJICOS SESCAM | 1 | 0.137 |
| HOSP NEUROREHABILITACIO | 1 | 0.137 |
| HOSP SANTA MARIA | 1 | 0.137 |
| HOSPITAL PUERTA DE HIERRO MAJADAHONDA | 1 | 0.137 |
| HOWARD UNIVERSITY | 1 | 0.137 |
| HUANGSHI CENT HOSP | 1 | 0.137 |
| HUAZHONG UNIVERSITY OF SCIENCE TECHNOLOGY | 1 | 0.137 |
| HUMAN ENGN RES LABS | 1 | 0.137 |
| HYOGO UNIV HLTH SCI | 1 | 0.137 |
| ICORD | 1 | 0.137 |
| IDIBAPS | 1 | 0.137 |
| ILLINOIS STATE UNIVERSITY | 1 | 0.137 |
| INONU UNIVERSITY | 1 | 0.137 |
| INOVA FAIRFAX HOSPITAL | 1 | 0.137 |
| INOVA HEALTH SYSTEM | 1 | 0.137 |
| INST CANC ESTADO SAO PAULO | 1 | 0.137 |
| INST CLIN OUTCOMES RES | 1 | 0.137 |
| INST GUTTMAN | 1 | 0.137 |
| INST GUTTMANN | 1 | 0.137 |
| INST NEUROPATH PAIN | 1 | 0.137 |
| INST UNIV NEUROREHABILITACIO ADSCRIT UAB | 1 | 0.137 |
| INSTITUT D INVESTIGACIO SANITARIA PERE VIRGILI IISPV | 1 | 0.137 |
| INSTITUT NATIONAL POLYTECHNIQUE DE GRENOBLE | 1 | 0.137 |
| INSTITUTE OF METAL RESEARCH CAS | 1 | 0.137 |
| INT CLIN RES INST | 1 | 0.137 |
| INTERDISCIPLINARY CTR RES REHABIL SOCIAL INTEGR | 1 | 0.137 |
| IRCCS BAMBINO GESU | 1 | 0.137 |
| IRCCS CA GRANDA OSPEDALE MAGGIORE POLICLINICO | 1 | 0.137 |
| IRCCS CTR NEUROLESI MESSINA | 1 | 0.137 |
| IRCCS EUGENIO MEDEA | 1 | 0.137 |
| IRCCS FONDAZIONE CASIMIRO MONDINO | 1 | 0.137 |
| IRCCS FONDAZIONE DON CARLO GNOCCHI ONLUS | 1 | 0.137 |
| IRCCS FONDAZIONE SALVATORE MAUGERI | 1 | 0.137 |
| IRCCS ISTITUTO DELLE SCIENZE NEUROLOGICHE DI BOLOGNA ISNB | 1 | 0.137 |
| IRCCS ISTITUTO ORTOPEDICO RIZZOLI | 1 | 0.137 |
| IRONWOOD PHARMACEUT INC | 1 | 0.137 |
| IST SUPER OSTEOPATIA | 1 | 0.137 |
| ITHACA COLL | 1 | 0.137 |
| JACKSON MEM REHABIL CTR | 1 | 0.137 |
| JAMES A HALEY VETERANS HOSPITAL | 1 | 0.137 |
| JAPAN SOCIETY FOR THE PROMOTION OF SCIENCE | 1 | 0.137 |
| JAWAHARLAL NEHRU UNIVERSITY NEW DELHI | 1 | 0.137 |
| JERSEY MED SCH | 1 | 0.137 |
| JERUSALEM MENTAL HLTH CTR | 1 | 0.137 |
| JEWISH HOME HOSP | 1 | 0.137 |
| JIANGSU INST ANESTHESIOL | 1 | 0.137 |
| JIANGXI PROV KEY LAB AUTON NERVOUS FUNCT DIS | 1 | 0.137 |
| JINAN FIRST PEOPLES HOSP | 1 | 0.137 |
| JK STAT DATA CONSULTANCY | 1 | 0.137 |
| JMERC | 1 | 0.137 |
| JOHN CURTIN SCHOOL OF MEDICAL RESEARCH | 1 | 0.137 |
| JOHN D DINGELL VA MED CTR | 1 | 0.137 |
| JOHN H STROGER JUNIOR HOSPITAL COOK COUNTY | 1 | 0.137 |
| JOHNS HOPKINS MEDICINE | 1 | 0.137 |
| JOHNSON JOHNSON | 1 | 0.137 |
| JOHNSON JOHNSON USA | 1 | 0.137 |
| KAGOSHIMA UNIVERSITY | 1 | 0.137 |
| KEESLER INST REHABIL | 1 | 0.137 |
| KESSLER ADVENTIST REHABIL SERV | 1 | 0.137 |
| KHATAM OL ANBIA HOSP | 1 | 0.137 |
| KHON KAEN UNIVERSITY | 1 | 0.137 |
| KIO UNIV | 1 | 0.137 |
| KIRISHIMA ORTHOPED | 1 | 0.137 |
| KIRKLIN CLIN | 1 | 0.137 |
| KONYA NUMUNE HOSPITAL | 1 | 0.137 |
| KOREA INSTITUTE OF ORIENTAL MEDICINE KIOM | 1 | 0.137 |
| KOREA INSTITUTE OF SCIENCE TECHNOLOGY KIST | 1 | 0.137 |
| KUNMING MEDICAL UNIVERSITY | 1 | 0.137 |
| KURUME UNIVERSITY | 1 | 0.137 |
| KUSHIRO ROSAI HOSP | 1 | 0.137 |
| KYOTO UNIVERSITY | 1 | 0.137 |
| KYUNGPOOK NATIONAL UNIVERSITY | 1 | 0.137 |
| LAHEY HOSPITAL MEDICAL CENTER | 1 | 0.137 |
| LIVERPOOL JOHN MOORES UNIVERSITY | 1 | 0.137 |
| LOIS POPE LIFE CTR | 1 | 0.137 |
| LOMA LINDA UNIVERSITY | 1 | 0.137 |
| LUCERNE CANTONAL HOSPITAL | 1 | 0.137 |
| MACKAY MEDICAL COLLEGE | 1 | 0.137 |
| MACQUARIE UNIVERSITY | 1 | 0.137 |
| MAHARISHI MARKANDESHWAR UNIVERSITY | 1 | 0.137 |
| MARQUETTE UNIVERSITY | 1 | 0.137 |
| MARUISHI PHARMACEUT CO LTD | 1 | 0.137 |
| MAX PLANCK SOCIETY | 1 | 0.137 |
| MCMASTER UNIVERSITY | 1 | 0.137 |
| MED COLL TAISHAN | 1 | 0.137 |
| MED CTR | 1 | 0.137 |
| MED HOSP BEIBEI DIST | 1 | 0.137 |
| MEDICAL CENTER OF ALKMAAR | 1 | 0.137 |
| MEDSURANT LLC | 1 | 0.137 |
| MEDVAMC 145 | 1 | 0.137 |
| MEDVAMC 153 | 1 | 0.137 |
| MEHMET AKIF ERSOY UNIVERSITY | 1 | 0.137 |
| MERCK COMPANY | 1 | 0.137 |
| MIAMI DEPT VET AFFAIRS MED CTR | 1 | 0.137 |
| MIAMI MED CTR | 1 | 0.137 |
| MIAMI PROJECT CURE PARALYSIS | 1 | 0.137 |
| MIAMI PROJECT CURE PARALYSIS R 48 | 1 | 0.137 |
| MIAMI PROJECT CURE PARALYSIS R48 | 1 | 0.137 |
| MIAMI UNIV | 1 | 0.137 |
| MIL MED ACAD | 1 | 0.137 |
| MILLENNIUM PAIN CTR | 1 | 0.137 |
| MINISTRY OF HEALTH TURKEY | 1 | 0.137 |
| MINNEAPOLIS VA HEALTH CARE SYSTEM | 1 | 0.137 |
| MODEL SPINAL CORD INJURY CARE SYST SO CALIF | 1 | 0.137 |
| MONTECATONE INST NEUROREHABIL | 1 | 0.137 |
| MONTECATONE REHABIL INST | 1 | 0.137 |
| MOSSREHAB | 1 | 0.137 |
| MUDANJIANG MEDICAL UNIVERSITY | 1 | 0.137 |
| NANCHANG UNIVERSITY | 1 | 0.137 |
| NANTONG HOSP TRADIT CHINESE MED | 1 | 0.137 |
| NARA PREFECTURE GEN REHABIL CTR | 1 | 0.137 |
| NATIONAL INSTITUTE FOR PHYSIOLOGICAL SCIENCES NIPS | 1 | 0.137 |
| NATIONAL INSTITUTE OF HEALTH SCIENCES JAPAN | 1 | 0.137 |
| NATIONAL INSTITUTE OF MENTAL HEALTH NEUROSCIENCES INDIA | 1 | 0.137 |
| NATIONAL INSTITUTES OF NATURAL SCIENCES NINS JAPAN | 1 | 0.137 |
| NATIONAL TAIPEI UNIVERSITY OF NURSING HEALTH SCIENCE NTUNHS | 1 | 0.137 |
| NATIONAL TAIWAN UNIVERSITY HOSPITAL | 1 | 0.137 |
| NATIONAL UNIVERSITY OF SINGAPORE | 1 | 0.137 |
| NATL REHABIL CTR | 1 | 0.137 |
| NED UNIVERSITY OF ENGINEERING TECHNOLOGY | 1 | 0.137 |
| NEURONA THERAPEUT | 1 | 0.137 |
| NEUROSCIENCE RESEARCH AUSTRALIA | 1 | 0.137 |
| NEUROSEARCH AS | 1 | 0.137 |
| NEUROSURG DAMME | 1 | 0.137 |
| NICOX RES INST | 1 | 0.137 |
| NIGDE STATE HOSPITAL | 1 | 0.137 |
| NIH CLINICAL CENTER CC | 1 | 0.137 |
| NIH EUNICE KENNEDY SHRIVER NATIONAL INSTITUTE OF CHILD HEALTH HUMAN DEVELOPMENT NICHD | 1 | 0.137 |
| NIPPON MEDICAL SCHOOL | 1 | 0.137 |
| NORTHEASTERN UNIVERSITY | 1 | 0.137 |
| NORTHERN TECHNICAL UNIVERSITY | 1 | 0.137 |
| NORTHWELL HEALTH | 1 | 0.137 |
| NOVA TECH EEG | 1 | 0.137 |
| NSW AGCY CLIN INNOVAT | 1 | 0.137 |
| OHU UNIV | 1 | 0.137 |
| ORTHOPED CLIN | 1 | 0.137 |
| OSPEDALE NIGUARDA CA GRANDA | 1 | 0.137 |
| PALM BEACH NEUROL CTR | 1 | 0.137 |
| PARAPLEG CTR | 1 | 0.137 |
| PARC CIENT BARCELONA | 1 | 0.137 |
| PARKER UNIV | 1 | 0.137 |
| PATIENT PHYS PARTNERSHIP | 1 | 0.137 |
| PAVLOV INSTITUTE OF PHYSIOLOGY RUSSIAN ACADEMY OF SCIENCES | 1 | 0.137 |
| PFIZER JAPAN | 1 | 0.137 |
| PHARMERIT INT | 1 | 0.137 |
| PIETRA LIGURE PUBL HOSP | 1 | 0.137 |
| POLYTECH INST LEIRIA | 1 | 0.137 |
| PRINCE WALES HOSP | 1 | 0.137 |
| PURDUE UNIV NORTHWEST | 1 | 0.137 |
| PURDUE UNIVERSITY | 1 | 0.137 |
| PURDUE UNIVERSITY SYSTEM | 1 | 0.137 |
| PUSAN NATIONAL UNIVERSITY | 1 | 0.137 |
| PUSAN NATIONAL UNIVERSITY HOSPITAL | 1 | 0.137 |
| QIDONG PEOPLES HOSP | 1 | 0.137 |
| QINGDAO UNIVERSITY | 1 | 0.137 |
| QUEEN ELIZABETH II HEALTH SCIENCES CENTRE | 1 | 0.137 |
| QUEEN MARY UNIVERSITY LONDON | 1 | 0.137 |
| RABIN MEDICAL CENTER | 1 | 0.137 |
| RADBOUD UNIVERSITY NIJMEGEN | 1 | 0.137 |
| RANCHO LOS AMIGOS MED CTR | 1 | 0.137 |
| RANCHO LOS AMIGOS NATL REHABIL CTR | 1 | 0.137 |
| RANCHO LOS AMIGOS REHABIL CTR | 1 | 0.137 |
| RED CROSS HOSP | 1 | 0.137 |
| REHAB STN STOCKHOLM | 1 | 0.137 |
| REHAB STN STOCKHOLM SPINALIS | 1 | 0.137 |
| REHABIL CTR AMSTERDAM | 1 | 0.137 |
| REHABIL CTR HET ROESSINGH | 1 | 0.137 |
| REHABIL CTR HOOGSTR | 1 | 0.137 |
| REHABIL CTR ROESSINGH | 1 | 0.137 |
| REHABIL ORTON | 1 | 0.137 |
| REHMAN MED COLL | 1 | 0.137 |
| RICK HANSEN INST | 1 | 0.137 |
| RKU UNIV REHABIL KLINIKEN ULM | 1 | 0.137 |
| ROYAL LONDON HOSPITAL | 1 | 0.137 |
| ROYAL PERTH HOSPITAL | 1 | 0.137 |
| ROYAL REHAB CTR | 1 | 0.137 |
| ROYAL REHABIL CTR | 1 | 0.137 |
| ROYAL REHABILITAT CTR | 1 | 0.137 |
| RUHR UNIVERSITY BOCHUM | 1 | 0.137 |
| RUSSIAN ACADEMY OF SCIENCES | 1 | 0.137 |
| RUTGERS NEW JERSEY SCH MED | 1 | 0.137 |
| RUTGERS STATE UNIVERSITY NEWARK | 1 | 0.137 |
| SABBATSBERGS HOSP | 1 | 0.137 |
| SACRAMENTO VA MED CTR | 1 | 0.137 |
| SAMOT | 1 | 0.137 |
| SANJAY GANDHI POSTGRADUATE INSTITUTE OF MEDICAL SCIENCES | 1 | 0.137 |
| SAPIENZA UNIVERSITY ROME | 1 | 0.137 |
| SARAH NETWORK REHABIL HOSP | 1 | 0.137 |
| SCH ENGN | 1 | 0.137 |
| SCH LANGUAGE COMMUN DEV | 1 | 0.137 |
| SCHULTHESS CLINIC | 1 | 0.137 |
| SCHWARZ BIOSCI | 1 | 0.137 |
| SCRIPPS RESEARCH INSTITUTE | 1 | 0.137 |
| SCUOLA SUPERIORE SANT ANNA | 1 | 0.137 |
| SENBOKU KUMIAI GEN HOSP | 1 | 0.137 |
| SESCAM | 1 | 0.137 |
| SEVKET YILMAZ TRAINING RESEARCH HOSPITAL | 1 | 0.137 |
| SHANDONG JINING 1 PEOPLES HOSP | 1 | 0.137 |
| SHANGHAI GONGLI HOSP | 1 | 0.137 |
| SHANGHAI JIAO TONG UNIVERSITY | 1 | 0.137 |
| SHANGHAI KAIYUAN ORTHOPED HOSP | 1 | 0.137 |
| SHANGHAI UNIVERSITY OF SPORT | 1 | 0.137 |
| SHIN KONG WU HO MEM HOSP | 1 | 0.137 |
| SICHUAN PROVINCIAL PEOPLE S HOSPITAL | 1 | 0.137 |
| SICHUAN UNIVERSITY | 1 | 0.137 |
| SIXTH MEDICAL CENTER OF CHINESE PLA GENERAL HOSPITAL | 1 | 0.137 |
| SLOTERVAART HOSPITAL | 1 | 0.137 |
| SLOVAK ACADEMY OF SCIENCES | 1 | 0.137 |
| SMS MEDICAL COLLEGE HOSPITAL | 1 | 0.137 |
| SOGANG UNIVERSITY | 1 | 0.137 |
| SONDALO PUBL HOSP | 1 | 0.137 |
| SOTERIX MED | 1 | 0.137 |
| SOUTH LONDON MAUDSLEY NHS TRUST | 1 | 0.137 |
| SPAULDING NEW ENGLAND REG SCI CTR | 1 | 0.137 |
| SPINALIS SCI UNIT | 1 | 0.137 |
| SRC REHABIL HOSP | 1 | 0.137 |
| ST JOSEPHS PARKWOOD HOSP | 1 | 0.137 |
| ST LUKES REHABIL INST | 1 | 0.137 |
| ST PETERSBURG SCIENTIFIC CENTRE OF THE RUSSIAN ACADEMY OF SCIENCES | 1 | 0.137 |
| STANFORD UNIVERSITY | 1 | 0.137 |
| STATE UNIVERSITY OF NEW YORK SUNY BUFFALO | 1 | 0.137 |
| STATE UNIVERSITY OF NEW YORK SUNY SYSTEM | 1 | 0.137 |
| STOCKHOLM UNIVERSITY | 1 | 0.137 |
| STOKE MANDEVILLE HOSP NHS TRUST | 1 | 0.137 |
| SUN YAT SEN UNIVERSITY | 1 | 0.137 |
| SUNNAAS REHABIL HOSP | 1 | 0.137 |
| SUVACARE | 1 | 0.137 |
| SW COLL NATUROPATH MED | 1 | 0.137 |
| SWEDISH SCHOOL OF SPORT HEALTH SCIENCES | 1 | 0.137 |
| SYRACUSE DVAMC | 1 | 0.137 |
| TAIZHOU PEOPLES HOSP | 1 | 0.137 |
| TANTA UNIVERSITY | 1 | 0.137 |
| TEIKYO UNIVERSITY | 1 | 0.137 |
| TEXAS A M HEALTH SCIENCE CENTER | 1 | 0.137 |
| TEXAS INST REHABIL RES | 1 | 0.137 |
| TEXAS STATE UNIVERSITY SAN MARCOS | 1 | 0.137 |
| TEXAS STATE UNIVERSITY SYSTEM | 1 | 0.137 |
| TEXAS WOMANS UNIVERSITY | 1 | 0.137 |
| TIRR | 1 | 0.137 |
| TIRR MEM HERMANN | 1 | 0.137 |
| TOHO UNIVERSITY | 1 | 0.137 |
| TOKYO MEDICAL DENTAL UNIVERSITY TMDU | 1 | 0.137 |
| TOKYO WOMEN S MEDICAL UNIVERSITY | 1 | 0.137 |
| TORONTO CONGENITAL CARDIAC CENTRE FOR ADULTS | 1 | 0.137 |
| TULANE UNIVERSITY | 1 | 0.137 |
| TURAN EMEKSIZ SOKAK | 1 | 0.137 |
| TURKISH ARMED FORCES REHABILITATION MAINTENANCE CENTER | 1 | 0.137 |
| UBC SCI SOLUT | 1 | 0.137 |
| UBON RATCHATHANI UNIVERSITY | 1 | 0.137 |
| ULM UNIVERSITY | 1 | 0.137 |
| ULUDAG UNIVERSITY | 1 | 0.137 |
| UMEA UNIVERSITY | 1 | 0.137 |
| UNIBRASIL | 1 | 0.137 |
| UNITED STATES ARMY | 1 | 0.137 |
| UNITED STATES DEPARTMENT OF DEFENSE | 1 | 0.137 |
| UNIV MED SCI | 1 | 0.137 |
| UNIVERSIDAD DE ANTIOQUIA | 1 | 0.137 |
| UNIVERSIDAD DE MALAGA | 1 | 0.137 |
| UNIVERSIDAD REY JUAN CARLOS | 1 | 0.137 |
| UNIVERSIDADE CIDADE DE SAO PAULO | 1 | 0.137 |
| UNIVERSIDADE FEDERAL DE SANTA CATARINA UFSC | 1 | 0.137 |
| UNIVERSIDADE FEDERAL DO CEARA | 1 | 0.137 |
| UNIVERSIDADE PORTUCALENSE INFANTE D HENRIQUE | 1 | 0.137 |
| UNIVERSIDADE PRESBITERIANA MACKENZIE | 1 | 0.137 |
| UNIVERSITAT ROVIRA I VIRGILI | 1 | 0.137 |
| UNIVERSITE DE PARIS | 1 | 0.137 |
| UNIVERSITE DE STRASBOURG | 1 | 0.137 |
| UNIVERSITE GRENOBLE ALPES UGA | 1 | 0.137 |
| UNIVERSITES DE STRASBOURG ETABLISSEMENTS ASSOCIES | 1 | 0.137 |
| UNIVERSITY HOSPITAL OF BERN | 1 | 0.137 |
| UNIVERSITY OF ABERDEEN | 1 | 0.137 |
| UNIVERSITY OF ADELAIDE | 1 | 0.137 |
| UNIVERSITY OF ANTWERP | 1 | 0.137 |
| UNIVERSITY OF BERN | 1 | 0.137 |
| UNIVERSITY OF BOLOGNA | 1 | 0.137 |
| UNIVERSITY OF CALIFORNIA DAVIS | 1 | 0.137 |
| UNIVERSITY OF CAMBRIDGE | 1 | 0.137 |
| UNIVERSITY OF COLORADO DENVER | 1 | 0.137 |
| UNIVERSITY OF DELAWARE | 1 | 0.137 |
| UNIVERSITY OF DUNDEE | 1 | 0.137 |
| UNIVERSITY OF GENOA | 1 | 0.137 |
| UNIVERSITY OF GOTTINGEN | 1 | 0.137 |
| UNIVERSITY OF HELSINKI | 1 | 0.137 |
| UNIVERSITY OF HONG KONG | 1 | 0.137 |
| UNIVERSITY OF ILLINOIS CHICAGO | 1 | 0.137 |
| UNIVERSITY OF ILLINOIS CHICAGO HOSPITAL | 1 | 0.137 |
| UNIVERSITY OF ILLINOIS SYSTEM | 1 | 0.137 |
| UNIVERSITY OF JINAN | 1 | 0.137 |
| UNIVERSITY OF LAUSANNE | 1 | 0.137 |
| UNIVERSITY OF LIEGE | 1 | 0.137 |
| UNIVERSITY OF LOUISVILLE | 1 | 0.137 |
| UNIVERSITY OF MILAN | 1 | 0.137 |
| UNIVERSITY OF MISSOURI COLUMBIA | 1 | 0.137 |
| UNIVERSITY OF MONTREAL | 1 | 0.137 |
| UNIVERSITY OF NOTTINGHAM | 1 | 0.137 |
| UNIVERSITY OF OTAGO | 1 | 0.137 |
| UNIVERSITY OF OTTAWA | 1 | 0.137 |
| UNIVERSITY OF PERUGIA | 1 | 0.137 |
| UNIVERSITY OF PISA | 1 | 0.137 |
| UNIVERSITY OF PUERTO RICO | 1 | 0.137 |
| UNIVERSITY OF PUERTO RICO MEDICAL SCIENCES CAMPUS | 1 | 0.137 |
| UNIVERSITY OF ROCHESTER | 1 | 0.137 |
| UNIVERSITY OF SANTO TOMAS | 1 | 0.137 |
| UNIVERSITY OF SHERBROOKE | 1 | 0.137 |
| UNIVERSITY OF SOUTH AUSTRALIA | 1 | 0.137 |
| UNIVERSITY OF SOUTH CHINA | 1 | 0.137 |
| UNIVERSITY OF SOUTHAMPTON | 1 | 0.137 |
| UNIVERSITY OF STRATHCLYDE | 1 | 0.137 |
| UNIVERSITY OF TEHRAN | 1 | 0.137 |
| UNIVERSITY OF TEXAS AUSTIN | 1 | 0.137 |
| UNIVERSITY OF TEXAS HEALTH SAN ANTONIO | 1 | 0.137 |
| UNIVERSITY OF TOKYO | 1 | 0.137 |
| UNIVERSITY OF TURKU | 1 | 0.137 |
| UNIVERSITY OF UTAH | 1 | 0.137 |
| UNIVERSITY OF WATERLOO | 1 | 0.137 |
| UNIVERSITY OF WESTERN AUSTRALIA | 1 | 0.137 |
| UNIVERSITY SYSTEM OF GEORGIA | 1 | 0.137 |
| US ARMY | 1 | 0.137 |
| UT HLTH | 1 | 0.137 |
| UTAH SYSTEM OF HIGHER EDUCATION | 1 | 0.137 |
| VA LOMA LINDA HEALTHCARE SYST | 1 | 0.137 |
| VA PALO ALTO HEALTH CARE SYSTEM | 1 | 0.137 |
| VALDOSTA STATE UNIVERSITY | 1 | 0.137 |
| VANDERBILT UNIVERSITY | 1 | 0.137 |
| VET ADM | 1 | 0.137 |
| VET ADM MED CTR W ROXBURY | 1 | 0.137 |
| VICTOR BABES UNIVERSITY OF MEDICINE PHARMACY TIMISOARA | 1 | 0.137 |
| VIRGINAIA CONNECTICUT HEALTHCARE SYST | 1 | 0.137 |
| VIRGINIA CONNECTICUT HEALTHCARE SYST | 1 | 0.137 |
| VU UNIVERSITY MEDICAL CENTER | 1 | 0.137 |
| WAKAYAMA MEDICAL UNIVERSITY | 1 | 0.137 |
| WAKE FOREST BAPTIST MEDICAL CENTER | 1 | 0.137 |
| WAKE FOREST UNIVERSITY | 1 | 0.137 |
| WALTER REED NATIONAL MILITARY MEDICAL CENTER | 1 | 0.137 |
| WALTON CENTRE | 1 | 0.137 |
| WASHINGTON STATE UNIVERSITY | 1 | 0.137 |
| WERNER WICKER CLIN | 1 | 0.137 |
| WESTMEAD INSTITUTE FOR MEDICAL RESEARCH | 1 | 0.137 |
| WILLIAM PATERSON UNIV | 1 | 0.137 |
| WOODROW WILSON REHABIL CTR | 1 | 0.137 |
| WUHAN UNIVERSITY | 1 | 0.137 |
| XUZHOU MEDICAL COLLEGE | 1 | 0.137 |
| YAMAGUCHI UNIVERSITY | 1 | 0.137 |
| YORK UNIVERSITY CANADA | 1 | 0.137 |
| ZHENGZHOU ORTHOPAED HOSP | 1 | 0.137 |
| ZONMW | 1 | 0.137 |
| ZUCKER SCH MED HOFSTRA NORTHWELL | 1 | 0.137 |
| UNIVERSITY OF MIAMI | 64 | 8.767 |
| UNIVERSITY OF WASHINGTON | 47 | 6.438 |
| UNIVERSITY OF WASHINGTON SEATTLE | 47 | 6.438 |
| UNIVERSITY OF TEXAS SYSTEM | 43 | 5.89 |
| UNIVERSITY OF SYDNEY | 42 | 5.753 |
| US DEPARTMENT OF VETERANS AFFAIRS | 40 | 5.479 |
| VETERANS HEALTH ADMINISTRATION VHA | 40 | 5.479 |
| ROYAL NORTH SHORE HOSPITAL | 38 | 5.205 |
| AARHUS UNIVERSITY | 35 | 4.795 |
| UNIVERSITY OF ALABAMA BIRMINGHAM | 29 | 3.973 |
| UNIVERSITY OF ALABAMA SYSTEM | 29 | 3.973 |
| KAROLINSKA INSTITUTET | 28 | 3.836 |
| UNIVERSITY OF TEXAS MEDICAL BRANCH GALVESTON | 25 | 3.425 |
| HARVARD UNIVERSITY | 22 | 3.014 |
| UNIVERSITY OF MICHIGAN | 18 | 2.466 |
| UNIVERSITY OF MICHIGAN SYSTEM | 18 | 2.466 |
| BAYLOR COLLEGE OF MEDICINE | 16 | 2.192 |
| DANDERYDS HOSPITAL | 16 | 2.192 |
| ICAHN SCHOOL OF MEDICINE AT MOUNT SINAI | 16 | 2.192 |
| KAROLINSKA UNIVERSITY HOSPITAL | 16 | 2.192 |
| UNIVERSITY OF CALIFORNIA SYSTEM | 16 | 2.192 |
| UNIVERSITY OF BRITISH COLUMBIA | 14 | 1.918 |
| UNIVERSITY OF COPENHAGEN | 13 | 1.781 |
| UNIVERSITY OF TEXAS HEALTH SCIENCE CENTER HOUSTON | 13 | 1.781 |
| RIGSHOSPITALET | 12 | 1.644 |
| STATE UNIVERSITY SYSTEM OF FLORIDA | 12 | 1.644 |
| UNIVERSITY COLLEGE LONDON | 12 | 1.644 |
| UNIVERSITY OF FLORIDA | 12 | 1.644 |
| UNIVERSITY OF LONDON | 12 | 1.644 |
| PFIZER | 11 | 1.507 |
| SEOUL NATIONAL UNIVERSITY SNU | 11 | 1.507 |
| TEL AVIV UNIVERSITY | 11 | 1.507 |
| YALE UNIVERSITY | 11 | 1.507 |
| KESSLER INSTITUTE FOR REHABILITATION | 10 | 1.37 |
| RUTGERS STATE UNIVERSITY MEDICAL CENTER | 10 | 1.37 |
| RUTGERS STATE UNIVERSITY NEW BRUNSWICK | 10 | 1.37 |
| AUTONOMOUS UNIVERSITY OF BARCELONA | 9 | 1.233 |
| UNIVERSITY OF MARYLAND BALTIMORE | 9 | 1.233 |
| UNIVERSITY OF TORONTO | 9 | 1.233 |
| UNIVERSITY SYSTEM OF MARYLAND | 9 | 1.233 |
| VA CONNECTICUT HEALTHCARE SYSTEM | 9 | 1.233 |
| WESTERN UNIVERSITY UNIVERSITY OF WESTERN ONTARIO | 9 | 1.233 |
| BETH ISRAEL DEACONESS MEDICAL CENTER | 8 | 1.096 |
| BRIGHAM WOMEN S HOSPITAL | 8 | 1.096 |
| JOHNS HOPKINS UNIVERSITY | 8 | 1.096 |
| NORTHWESTERN UNIVERSITY | 8 | 1.096 |
| PENNSYLVANIA COMMONWEALTH SYSTEM OF HIGHER EDUCATION PCSHE | 8 | 1.096 |
| SACKLER FACULTY OF MEDICINE | 8 | 1.096 |
| SWISS PARAPLEGIC RESEARCH | 8 | 1.096 |
| TEHRAN UNIVERSITY OF MEDICAL SCIENCES | 8 | 1.096 |
| TEXAS A M UNIVERSITY COLLEGE STATION | 8 | 1.096 |
| TEXAS A M UNIVERSITY SYSTEM | 8 | 1.096 |
| UNIVERSITY HEALTH NETWORK TORONTO | 8 | 1.096 |
| UNIVERSITY OF PITTSBURGH | 8 | 1.096 |
| UNIVERSITY OF ZURICH | 8 | 1.096 |
| IRAN UNIVERSITY OF MEDICAL SCIENCES | 7 | 0.959 |
| KOLLING INSTITUTE OF MEDICAL RESEARCH | 7 | 0.959 |
| LAVAL UNIVERSITY | 7 | 0.959 |
| MEDICAL UNIVERSITY OF SOUTH CAROLINA | 7 | 0.959 |
| SPAULDING REHABILITATION HOSPITAL | 7 | 0.959 |
| UNIVERSITY OF MINNESOTA SYSTEM | 7 | 0.959 |
| UNIVERSITY OF MINNESOTA TWIN CITIES | 7 | 0.959 |
| UTRECHT UNIVERSITY | 7 | 0.959 |
| UTRECHT UNIVERSITY MEDICAL CENTER | 7 | 0.959 |
| VAMC | 7 | 0.959 |
| ANKARA PHYSICAL THERAPY REHABILITATION HOSPITAL | 6 | 0.822 |
| CHAIM SHEBA MEDICAL CENTER | 6 | 0.822 |
| CRAIG HOSP | 6 | 0.822 |
| EAST CAROLINA UNIVERSITY | 6 | 0.822 |
| JEFFERSON UNIVERSITY | 6 | 0.822 |
| RUPRECHT KARLS UNIVERSITY HEIDELBERG | 6 | 0.822 |
| SHAHID BEHESHTI UNIVERSITY MEDICAL SCIENCES | 6 | 0.822 |
| UNIVERSITY OF KIEL | 6 | 0.822 |
| UNIVERSITY OF LUCERNE | 6 | 0.822 |
| UNIVERSITY OF NORTH CAROLINA | 6 | 0.822 |
| UNIVERSITY OF WISCONSIN MADISON | 6 | 0.822 |
| UNIVERSITY OF WISCONSIN SYSTEM | 6 | 0.822 |
| WAYNE STATE UNIVERSITY | 6 | 0.822 |
| WESTERN SYDNEY UNIVERSITY | 6 | 0.822 |
| BRUNEL UNIVERSITY | 5 | 0.685 |
| CAPITAL MEDICAL UNIVERSITY | 5 | 0.685 |
| CASE WESTERN RESERVE UNIVERSITY | 5 | 0.685 |
| CENTRE NATIONAL DE LA RECHERCHE SCIENTIFIQUE CNRS | 5 | 0.685 |
| CIBERNED | 5 | 0.685 |
| DEPT VET AFFAIRS MED CTR | 5 | 0.685 |
| FEINBERG SCHOOL OF MEDICINE | 5 | 0.685 |
| MAASTRICHT UNIVERSITY | 5 | 0.685 |
| REG HOSP VIBORG | 5 | 0.685 |
| SCHLESWIG HOLSTEIN UNIVERSITY HOSPITAL | 5 | 0.685 |
| UNIVERSIDADE DE SAO PAULO | 5 | 0.685 |
| UNIVERSITY OF CALIFORNIA IRVINE | 5 | 0.685 |
| UNIVERSITY OF CALIFORNIA SAN FRANCISCO | 5 | 0.685 |
| UNIVERSITY OF COLORADO SYSTEM | 5 | 0.685 |
| UNIVERSITY OF VERONA | 5 | 0.685 |
| VA PITTSBURGH HEALTHCARE SYSTEM | 5 | 0.685 |
| CATHOLIC UNIVERSITY OF KOREA | 4 | 0.548 |
| EDWARD HINES JR VA HOSPITAL | 4 | 0.548 |
| HARVARD MEDICAL SCHOOL | 4 | 0.548 |
| KOREA UNIVERSITY | 4 | 0.548 |
| MASSACHUSETTS GENERAL HOSPITAL | 4 | 0.548 |
| MAYO CLINIC | 4 | 0.548 |
| NATL REHABIL HOSP | 4 | 0.548 |
| ODENSE UNIVERSITY HOSPITAL | 4 | 0.548 |
| OSAKA UNIVERSITY | 4 | 0.548 |
| SHANDONG FIRST MEDICAL UNIVERSITY SHANDONG ACADEMY OF MEDICAL SCIENCES | 4 | 0.548 |
| SHIRLEY RYAN ABILITYLAB | 4 | 0.548 |
| STOKE MANDEVILLE HOSP | 4 | 0.548 |
| TIRR MEM HERMANN HOSP | 4 | 0.548 |
| UCL MEDICAL SCHOOL | 4 | 0.548 |
| UNIVERSIDAD DE CASTILLA LA MANCHA | 4 | 0.548 |
| UNIVERSITY COLLEGE DUBLIN | 4 | 0.548 |
| UNIVERSITY COLLEGE LONDON HOSPITALS NHS FOUNDATION TRUST | 4 | 0.548 |
| UNIVERSITY OF CALIFORNIA SAN DIEGO | 4 | 0.548 |
| UNIVERSITY OF GLASGOW | 4 | 0.548 |
| UNIVERSITY OF SOUTHERN DENMARK | 4 | 0.548 |
| UNIVERSITY OF TECHNOLOGY SYDNEY | 4 | 0.548 |
| ARMY MEDICAL UNIVERSITY | 3 | 0.411 |
| CHU LYON | 3 | 0.411 |
| CONSEJO NACIONAL DE INVESTIGACIONES CIENTIFICAS Y TECNICAS CONICET | 3 | 0.411 |
| DAEGU HAANY UNIVERSITY | 3 | 0.411 |
| DREXEL UNIVERSITY | 3 | 0.411 |
| EMORY UNIVERSITY | 3 | 0.411 |
| ETH ZURICH | 3 | 0.411 |
| GREENWICH HOSP | 3 | 0.411 |
| HAMMONDCARE | 3 | 0.411 |
| HOSP NACL PARAPLEJ | 3 | 0.411 |
| HYOGO COLLEGE OF MEDICINE | 3 | 0.411 |
| IMPERIAL COLLEGE LONDON | 3 | 0.411 |
| INST REHABIL RES | 3 | 0.411 |
| INSTITUT NATIONAL DE LA SANTE ET DE LA RECHERCHE MEDICALE INSERM | 3 | 0.411 |
| ISTANBUL PHYSICAL MEDICINE REHABILITATION TRAINING RESEARCH HOSPITAL | 3 | 0.411 |
| KOREA UNIVERSITY MEDICINE KU MEDICINE | 3 | 0.411 |
| KREMBIL RESEARCH INSTITUTE | 3 | 0.411 |
| KYUNG HEE UNIVERSITY | 3 | 0.411 |
| NANTONG UNIVERSITY | 3 | 0.411 |
| NATIONAL HOSPITAL NORWAY | 3 | 0.411 |
| OREGON HEALTH SCIENCE UNIVERSITY | 3 | 0.411 |
| PARACELSUS PRIVATE MEDICAL UNIVERSITY | 3 | 0.411 |
| PRINCE WALES MEDICAL RESEARCH INSTITUTE | 3 | 0.411 |
| QUEEN ELIZABETH UNIVERSITY HOSPITAL QEUH | 3 | 0.411 |
| QUEENS UNIVERSITY CANADA | 3 | 0.411 |
| REHABIL CTR DE HOOGSTRAAT | 3 | 0.411 |
| REHABIL INST MICHIGAN | 3 | 0.411 |
| ROYAL NATIONAL ORTHOPAEDIC HOSPITAL NHS TRUST | 3 | 0.411 |
| SALZBURG UNIVERSITY | 3 | 0.411 |
| SORBONNE UNIVERSITE | 3 | 0.411 |
| SPAIN REHABIL CTR | 3 | 0.411 |
| TOHOKU UNIVERSITY | 3 | 0.411 |
| TORONTO GENERAL HOSPITAL | 3 | 0.411 |
| UNIVERSIDADE DO MINHO | 3 | 0.411 |
| UNIVERSITY OF BARCELONA | 3 | 0.411 |
| UNIVERSITY OF BASEL | 3 | 0.411 |
| UNIVERSITY OF BERGEN | 3 | 0.411 |
| UNIVERSITY OF BUCKINGHAM | 3 | 0.411 |
| UNIVERSITY OF BUENOS AIRES | 3 | 0.411 |
| UNIVERSITY OF CINCINNATI | 3 | 0.411 |
| UNIVERSITY OF GENEVA | 3 | 0.411 |
| UNIVERSITY OF KENTUCKY | 3 | 0.411 |
| UNIVERSITY OF MISSOURI SYSTEM | 3 | 0.411 |
| UNIVERSITY OF NEW SOUTH WALES SYDNEY | 3 | 0.411 |
| UNIVERSITY OF OSLO | 3 | 0.411 |
| UNIVERSITY OF TEXAS DALLAS | 3 | 0.411 |
| UNIVERSITY OF VIRGINIA | 3 | 0.411 |
| UPPSALA UNIVERSITY | 3 | 0.411 |
| VA MED CTR | 3 | 0.411 |
| VET AFFAIRS PUGET SOUND HEALTH CARE SYSTEM | 3 | 0.411 |
| VRIJE UNIVERSITEIT AMSTERDAM | 3 | 0.411 |
| ZHENGZHOU UNIVERSITY | 3 | 0.411 |
| AALBORG UNIVERSITY | 2 | 0.274 |
| AIR FORCE MILITARY MEDICAL UNIVERSITY | 2 | 0.274 |
| AUSTIN RESEARCH INSTITUTE | 2 | 0.274 |
| BRUCE W CARTER VET AFFAIRS MED CTR | 2 | 0.274 |
| CENT HOSP TAIAN | 2 | 0.274 |
| CENTRAL SOUTH UNIVERSITY | 2 | 0.274 |
| CHIBA UNIVERSITY | 2 | 0.274 |
| CHINESE PLA ARMY GEN HOSP | 2 | 0.274 |
| CHUNGNAM NATIONAL UNIVERSITY | 2 | 0.274 |
| CLEVELAND FES CTR | 2 | 0.274 |
| CNRS NATIONAL INSTITUTE FOR BIOLOGY INSB | 2 | 0.274 |
| CONSEJO SUPERIOR DE INVESTIGACIONES CIENTIFICAS CSIC | 2 | 0.274 |
| CORNELL UNIVERSITY | 2 | 0.274 |
| CRAIG REHABIL HOSP | 2 | 0.274 |
| CTR COGNIT NEUROSCI | 2 | 0.274 |
| DALHOUSIE UNIVERSITY | 2 | 0.274 |
| DE HOOGSTR REHABIL | 2 | 0.274 |
| DUKE UNIVERSITY | 2 | 0.274 |
| F TAPPEINER HOSP | 2 | 0.274 |
| FUDAN UNIVERSITY | 2 | 0.274 |
| FUNDACIO INSTITUT D INVESTIGACIO EN CIENCIES DE LA SALUT GERMANS TRIAS I PUJOL IGTP | 2 | 0.274 |
| FUNING PEOPLES HOSP JIANGSU | 2 | 0.274 |
| GANNAN MEDICAL UNIVERSITY | 2 | 0.274 |
| HAMMEL NEUROREHABIL RES CTR | 2 | 0.274 |
| HONG KONG POLYTECHNIC UNIVERSITY | 2 | 0.274 |
| HOSPITAL CLINIC DE BARCELONA | 2 | 0.274 |
| HOSPITAL FOR JOINT DISEASE NYULMC | 2 | 0.274 |
| INDIANA UNIVERSITY BLOOMINGTON | 2 | 0.274 |
| INDIANA UNIVERSITY SYSTEM | 2 | 0.274 |
| INSTITUTE OF BIOLOGY EXPERIMENTAL MEDICINE | 2 | 0.274 |
| INT COLLABORAT REPAIR DISCOVERIES | 2 | 0.274 |
| IRCCS SANTA LUCIA | 2 | 0.274 |
| JILIN UNIVERSITY | 2 | 0.274 |
| KEIO UNIVERSITY | 2 | 0.274 |
| KENNEDY KRIEGER INSTITUTE | 2 | 0.274 |
| KYUSHU UNIVERSITY | 2 | 0.274 |
| LOUIS STOKES CLEVELAND VETERANS AFFAIRS MEDICAL CENTER | 2 | 0.274 |
| LOYOLA UNIVERSITY CHICAGO | 2 | 0.274 |
| MCGILL UNIVERSITY | 2 | 0.274 |
| MEDICAL COLLEGE OF WISCONSIN | 2 | 0.274 |
| MEDICAL PARK HOSPITALS GROUP | 2 | 0.274 |
| METROHEALTH SYSTEM | 2 | 0.274 |
| MICHAEL E DEBAKEY VA MEDICAL CENTER | 2 | 0.274 |
| NAGASAKI UNIVERSITY | 2 | 0.274 |
| NANJING MEDICAL UNIVERSITY | 2 | 0.274 |
| NANJING UNIVERSITY | 2 | 0.274 |
| NATIONAL DEFENSE MEDICAL CENTER | 2 | 0.274 |
| NATIONAL INSTITUTES OF HEALTH NIH USA | 2 | 0.274 |
| NATIONAL TAIWAN UNIVERSITY | 2 | 0.274 |
| NATL HOSP ORG | 2 | 0.274 |
| NETHERLANDS INSTITUTE FOR NEUROSCIENCE NIN KNAW | 2 | 0.274 |
| NEW YORK UNIVERSITY | 2 | 0.274 |
| NYU LANGONE MEDICAL CENTER | 2 | 0.274 |
| OHIO STATE UNIVERSITY | 2 | 0.274 |
| OSPEDALE FRANZ TAPPEINER | 2 | 0.274 |
| PARKWOOD HOSP | 2 | 0.274 |
| PARKWOOD INST | 2 | 0.274 |
| PINDERFIELDS HOSPITAL | 2 | 0.274 |
| PLA SECOND MILITARY MEDICAL UNIVERSITY | 2 | 0.274 |
| RIJNDAM REHABIL CTR | 2 | 0.274 |
| ROTHMAN INSTITUTE | 2 | 0.274 |
| ROYAL ADELAIDE HOSPITAL | 2 | 0.274 |
| ROYAL NETHERLANDS ACADEMY OF ARTS SCIENCES | 2 | 0.274 |
| ROYAL REHABIL CTR SYDNEY | 2 | 0.274 |
| RUSH UNIVERSITY | 2 | 0.274 |
| SALK INSTITUTE | 2 | 0.274 |
| SANTA CLARA VALLEY MEDICAL CENTER | 2 | 0.274 |
| SEATTLE UNIVERSITY | 2 | 0.274 |
| SELCUK UNIVERSITY | 2 | 0.274 |
| SEOUL NATIONAL UNIVERSITY HOSPITAL | 2 | 0.274 |
| SEOUL ST MARY S HOSPITAL | 2 | 0.274 |
| SHANDONG UNIVERSITY | 2 | 0.274 |
| SHENANDOAH UNIV | 2 | 0.274 |
| SHRINERS HOSP CHILDREN | 2 | 0.274 |
| ST JOSEPHS HEALTHCARE | 2 | 0.274 |
| TORONTO REHABILITATION INSTITUTE | 2 | 0.274 |
| TRI SERVICE GENERAL HOSPITAL | 2 | 0.274 |
| TRUVEN HLTH ANALYT | 2 | 0.274 |
| UNIV APPL SCI ARTS WESTERN SWITZERLAND HES SO | 2 | 0.274 |
| UNIVERSITAT DE GIRONA | 2 | 0.274 |
| UNIVERSITY OF ALBERTA | 2 | 0.274 |
| UNIVERSITY OF AMSTERDAM | 2 | 0.274 |
| UNIVERSITY OF CALIFORNIA LOS ANGELES | 2 | 0.274 |
| UNIVERSITY OF COLORADO ANSCHUTZ MEDICAL CAMPUS | 2 | 0.274 |
| UNIVERSITY OF COLORADO BOULDER | 2 | 0.274 |
| UNIVERSITY OF FUKUI | 2 | 0.274 |
| UNIVERSITY OF GRONINGEN | 2 | 0.274 |
| UNIVERSITY OF HAIFA | 2 | 0.274 |
| UNIVERSITY OF KANSAS | 2 | 0.274 |
| UNIVERSITY OF KANSAS MEDICAL CENTER | 2 | 0.274 |
| UNIVERSITY OF LIVERPOOL | 2 | 0.274 |
| UNIVERSITY OF MISSOURI KANSAS CITY | 2 | 0.274 |
| UNIVERSITY OF OXFORD | 2 | 0.274 |
| UNIVERSITY OF PAVIA | 2 | 0.274 |
| UNIVERSITY OF PENNSYLVANIA | 2 | 0.274 |
| UNIVERSITY OF SOUTHERN CALIFORNIA | 2 | 0.274 |
| UNIVERSITY OF TEXAS SOUTHWESTERN MEDICAL CENTER DALLAS | 2 | 0.274 |
| UPPSALA UNIVERSITY HOSPITAL | 2 | 0.274 |
| UTMD ANDERSON CANCER CENTER | 2 | 0.274 |
| VA ANN ARBOR HEALTHCARE SYSTEM | 2 | 0.274 |
| VA BOSTON HEALTHCARE SYSTEM | 2 | 0.274 |
| VA LONG BEACH HEALTHCARE SYSTEM | 2 | 0.274 |
| VA NORTH TEXAS HEALTH CARE SYSTEM | 2 | 0.274 |
| VIRGINIA COMMONWEALTH UNIVERSITY | 2 | 0.274 |
| VIRGINIA MASON MEDICAL CENTER | 2 | 0.274 |
| WASHINGTON STATE DEPT LABOR IND | 2 | 0.274 |
| WASIT UNIVERSITY | 2 | 0.274 |
| XI AN JIAOTONG UNIVERSITY | 2 | 0.274 |
| XINXIANG MEDICAL UNIVERSITY | 2 | 0.274 |
| YEUNGNAM UNIVERSITY | 2 | 0.274 |
| YONSEI UNIVERSITY | 2 | 0.274 |
| YONSEI UNIVERSITY HEALTH SYSTEM | 2 | 0.274 |
| 4D PHARMA RES LTD | 1 | 0.137 |
| 529C SPAIN REHABIL CTR | 1 | 0.137 |
| ABBVIE | 1 | 0.137 |
| ACAD TEACHING HOSP NORDSTADT | 1 | 0.137 |
| ACADEMIC MEDICAL CENTER AMSTERDAM | 1 | 0.137 |
| ACUPUNCTURE CTR | 1 | 0.137 |
| ADDENBROOKE S HOSPITAL | 1 | 0.137 |
| ADELANTE CTR EXPERTISE REHABIL AUDIOL | 1 | 0.137 |
| ADELANTE ZORGGROEP | 1 | 0.137 |
| ADELANTE ZORGGRP | 1 | 0.137 |
| ADIYAMAN UNIVERSITY | 1 | 0.137 |
| AGA KHAN UNIVERSITY | 1 | 0.137 |
| AIX MARSEILLE UNIVERSITE | 1 | 0.137 |
| AJAMED SCI UNIV | 1 | 0.137 |
| AJOU UNIVERSITY | 1 | 0.137 |
| ALESSANDRIA PUBL HOSP | 1 | 0.137 |
| ALEXANDRIA UNIVERSITY | 1 | 0.137 |
| ALL INDIA INSTITUTE OF MEDICAL SCIENCES AIIMS NEW DELHI | 1 | 0.137 |
| AMSTERDAM REHABIL RES CTR READE | 1 | 0.137 |
| AMSTERDAM REHABIL RES INST READE | 1 | 0.137 |
| ANKARA UNIVERSITY | 1 | 0.137 |
| ANN ARBOR VA MED CTR | 1 | 0.137 |
| ARISTOTLE UNIVERSITY OF THESSALONIKI | 1 | 0.137 |
| ASL 3 GENOVESE | 1 | 0.137 |
| ASSISTANCE PUBLIQUE HOPITAUX DE MARSEILLE | 1 | 0.137 |
| ASSISTANCE PUBLIQUE HOPITAUX PARIS APHP | 1 | 0.137 |
| ASTON UNIVERSITY | 1 | 0.137 |
| ASTRAZENECA | 1 | 0.137 |
| ATLANTIC HEALTH SYSTEM | 1 | 0.137 |
| AUCKLAND UNIVERSITY OF TECHNOLOGY | 1 | 0.137 |
| AUDIE L MURPHY MEMORIAL VETERANS HOSPITAL | 1 | 0.137 |
| AUSTRAL UNIVERSITY | 1 | 0.137 |
| AUSTRALIA COLL APPL PSYCHOL | 1 | 0.137 |
| AUSTRALIAN COLL APPL PSYCHOL | 1 | 0.137 |
| AUSTRALIAN NATIONAL UNIVERSITY | 1 | 0.137 |
| AUTONOMOUS UNIV | 1 | 0.137 |
| AVALON HLTH SOLUT INC | 1 | 0.137 |
| AZIENDA OSPEDALIERA DI PERUGIA | 1 | 0.137 |
| AZIENDA OSPEDALIERA UNIVERSITARIA INTEGRATA VERONA | 1 | 0.137 |
| AZIENDA OSPEDALIERO UNIVERSITARIA PISANA | 1 | 0.137 |
| BAMBERG HOSP | 1 | 0.137 |
| BAQIYATALLAH UNIVERSITY OF MEDICAL SCIENCES BMSU | 1 | 0.137 |
| BARTS HEALTH NHS TRUST | 1 | 0.137 |
| BASQUE CTR COGNIT BRAIN LANGUAGE | 1 | 0.137 |
| BAYSHORE MED CTR | 1 | 0.137 |
| BEIJING BOAI HOSP | 1 | 0.137 |
| BEIJING INST BRAIN DISORDERS | 1 | 0.137 |
| BEIJING KEY LAB NEURAL INJURY REHABIL | 1 | 0.137 |
| BEIJING NEUROSURGICAL INSTITUTE | 1 | 0.137 |
| BERUFSGENOSSENSCHAFTLICHE KLIN | 1 | 0.137 |
| BIEMAN COPLAND ASSOCIATES | 1 | 0.137 |
| BILL MELINDA GATES FOUNDATION | 1 | 0.137 |
| BLUSSON SPINAL CORD CTR | 1 | 0.137 |
| BOSTON CHILDREN S HOSPITAL | 1 | 0.137 |
| BOSTON UNIVERSITY | 1 | 0.137 |
| BROCK NIAGARA CTR HLTH WELL BEING | 1 | 0.137 |
| BROCK UNIVERSITY | 1 | 0.137 |
| BRUCE W CARTER DEPT VET AFFAIRS MED CTR | 1 | 0.137 |
| BUSHEHR UNIV MED SCI | 1 | 0.137 |
| C BESTA NEUROL INST FDN | 1 | 0.137 |
| CAGLIARI PUBL HOSP | 1 | 0.137 |
| CARING CHILDREN FDN | 1 | 0.137 |
| CAROLINAS REHABIL | 1 | 0.137 |
| CARROLL UNIV | 1 | 0.137 |
| CATALAN INSTITUTE OF ONCOLOGY | 1 | 0.137 |
| CATANIA PUBL HOSP | 1 | 0.137 |
| CENTRE HOSPITALIER UNIVERSITAIRE VAUDOIS CHUV | 1 | 0.137 |
| CHAPMAN UNIVERSITY | 1 | 0.137 |
| CHAPMAN UNIVERSITY SYSTEM | 1 | 0.137 |
| CHARLES STARK DRAPER LAB INC | 1 | 0.137 |
| CHEMOSERO THERAPEUT RES INST | 1 | 0.137 |
| CHEMPARTNER | 1 | 0.137 |
| CHILDREN S HOSPITAL COLORADO | 1 | 0.137 |
| CHILDRENS HOSP AUSTIN | 1 | 0.137 |
| CHINA THREE GORGES UNIVERSITY | 1 | 0.137 |
| CHINESE ACADEMY OF SCIENCES | 1 | 0.137 |
| CHINESE MED HOSP HULAN DIST | 1 | 0.137 |
| CHONGQING MEDICAL UNIVERSITY | 1 | 0.137 |
| CHONNAM NATIONAL UNIVERSITY | 1 | 0.137 |
| CHUNG ANG UNIVERSITY | 1 | 0.137 |
| CHUNGBUK NATIONAL UNIVERSITY | 1 | 0.137 |
| CHUNGBUK NATIONAL UNIVERSITY HOSPITAL | 1 | 0.137 |
| CITY COLLEGE OF NEW YORK CUNY | 1 | 0.137 |
| CITY UNIVERSITY OF NEW YORK CUNY SYSTEM | 1 | 0.137 |
| CLEMENT J ZABLOCKI VA MED CTR | 1 | 0.137 |
| CLEVELAND CLINIC FOUNDATION | 1 | 0.137 |
| CLIN ROMANDE READAPTAT | 1 | 0.137 |
| CLIN ROMANDE READAPTAT SUVACARE | 1 | 0.137 |
| COLL HLTH SCI | 1 | 0.137 |
| COLLEGE OF THE HOLY CROSS | 1 | 0.137 |
| COMMUNAUTE UNIVERSITE GRENOBLE ALPES | 1 | 0.137 |
| CONSORTIUM CTR BIOMED RES NETWORK NEURODEGENERAT | 1 | 0.137 |
| COVANCE | 1 | 0.137 |
| CRAWFORD RES INST | 1 | 0.137 |
| CRDR | 1 | 0.137 |
| CSIC INSTITUTO CAJAL IC | 1 | 0.137 |
| CTR INOVACAO ENSAIOS PRECLIN CIENP | 1 | 0.137 |
| CTR INTEGRAT MED INNOVAT TECHNOL | 1 | 0.137 |
| CTR INTERDISCIPLINAIRE RECH READAPTAT INTEGRAT | 1 | 0.137 |
| CTR INTERDISCIPLINARY RES REHABIL GREATER MONTREA | 1 | 0.137 |
| CTR NEUROSCI REGENERAT RES 127A | 1 | 0.137 |
| DA YEH UNIVERSITY | 1 | 0.137 |
| DAEGU HANNY UNIV | 1 | 0.137 |
| DALIAN UNIVERSITY | 1 | 0.137 |
| DARTMOUTH COLLEGE | 1 | 0.137 |
| DAVID GEFFEN SCHOOL OF MEDICINE AT UCLA | 1 | 0.137 |
| DE HOOGSTRAAT | 1 | 0.137 |
| DELFT UNIVERSITY OF TECHNOLOGY | 1 | 0.137 |
| DIV TRAUMA ACUTE CARE SURG | 1 | 0.137 |
| DOKKYO MEDICAL UNIVERSITY | 1 | 0.137 |
| EASTERN SCH ACUPUNCTURE | 1 | 0.137 |
| EBERHARD KARLS UNIVERSITY OF TUBINGEN | 1 | 0.137 |
| ECOLE POLYTECHNIQUE FEDERALE DE LAUSANNE | 1 | 0.137 |
| EGE UNIVERSITY | 1 | 0.137 |
| EHIME UNIVERSITY | 1 | 0.137 |
| ERASMUS MC | 1 | 0.137 |
| ERASMUS UNIVERSITY ROTTERDAM | 1 | 0.137 |
| ERCIYES UNIVERSITY | 1 | 0.137 |
| ESTEVE | 1 | 0.137 |
| ETERA MUTUAL PENS INSURANCE CO | 1 | 0.137 |
| EUROPEAN MULTICTR STUDY SPINAL CORD INJURY EMSCI | 1 | 0.137 |
| FAC EARTH LIFE SCI | 1 | 0.137 |
| FAC HLTH SCI | 1 | 0.137 |
| FDN ORTHOPED UNIV CLIN | 1 | 0.137 |
| FIRENZE UNIV HOSP | 1 | 0.137 |
| FLINDERS UNIVERSITY SOUTH AUSTRALIA | 1 | 0.137 |
| FLOREY INSTITUTE OF NEUROSCIENCE MENTAL HEALTH | 1 | 0.137 |
| FM KIRBY RES CTR FUNCT MRI | 1 | 0.137 |
| FMUSP | 1 | 0.137 |
| FOURTH PEOPLES HOSP SHAANXI | 1 | 0.137 |
| FROEDTERT MEM LUTHERAN HOSP | 1 | 0.137 |
| FROSUNDA CTR | 1 | 0.137 |
| FU JEN CATHOLIC UNIVERSITY | 1 | 0.137 |
| FUJIAN MEDICAL UNIVERSITY | 1 | 0.137 |
| FUKUOKA UNIVERSITY | 1 | 0.137 |
| FUKUSHIMA MEDICAL UNIVERSITY | 1 | 0.137 |
| GACHON UNIVERSITY | 1 | 0.137 |
| GAZI UNIVERSITY | 1 | 0.137 |
| GAZILER PHYSICAL THERAPY RESEARCH HOSPITAL | 1 | 0.137 |
| GEN HOSP CHINESE PEOPLES ARMED POLICE FORCES | 1 | 0.137 |
| GEORGE MASON UNIVERSITY | 1 | 0.137 |
| GERIATRIC RESEARCH EDUCATION CLINICAL CENTER | 1 | 0.137 |
| GLENROSE REHABIL HOSP | 1 | 0.137 |
| GLHANE MIL MED ACAD | 1 | 0.137 |
| GOETHE UNIVERSITY FRANKFURT | 1 | 0.137 |
| GOETHE UNIVERSITY FRANKFURT HOSPITAL | 1 | 0.137 |
| GOOD SAMARITAN HOSP | 1 | 0.137 |
| GUANGZHOU MEDICAL UNIVERSITY | 1 | 0.137 |
| GULHANE MILITARY MEDICAL ACADEMY | 1 | 0.137 |
| HACETTEPE UNIVERSITY | 1 | 0.137 |
| HAMMOND CARE | 1 | 0.137 |
| HANYANG UNIVERSITY | 1 | 0.137 |
| HARBIN MEDICAL UNIVERSITY | 1 | 0.137 |
| HARBORVIEW MEDICAL CENTER | 1 | 0.137 |
| HARVARD MED SCH | 1 | 0.137 |
| HELSINKI UNIVERSITY CENTRAL HOSPITAL | 1 | 0.137 |
| HENAN INST BIOL | 1 | 0.137 |
| HENAN UNIVERSITY OF TRADITIONAL CHINESE MEDICINE | 1 | 0.137 |
| HERMAN OSTROW SCH DENT | 1 | 0.137 |
| HIROSHIMA RED CROSS HOSP | 1 | 0.137 |
| HOFSTRA UNIVERSITY | 1 | 0.137 |
| HOKKAIDO CHUO ROSAI HOSP | 1 | 0.137 |
| HOLLYWOOD PRESBYTERIAN MED CTR | 1 | 0.137 |
| HOPITAL UNIVERSITAIRE BEAUJON APHP | 1 | 0.137 |
| HORIZON HLTH NETWORK | 1 | 0.137 |
| HOSHI UNIVERSITY | 1 | 0.137 |
| HOSP HOHE WARTE | 1 | 0.137 |
| HOSP NACL PARAPLEJICOS | 1 | 0.137 |
| HOSP NACL PARAPLEJICOS SESCAM | 1 | 0.137 |
| HOSP NEUROREHABILITACIO | 1 | 0.137 |
| HOSP SANTA MARIA | 1 | 0.137 |
| HOSPITAL PUERTA DE HIERRO MAJADAHONDA | 1 | 0.137 |
| HOWARD UNIVERSITY | 1 | 0.137 |
| HUANGSHI CENT HOSP | 1 | 0.137 |
| HUAZHONG UNIVERSITY OF SCIENCE TECHNOLOGY | 1 | 0.137 |
| HUMAN ENGN RES LABS | 1 | 0.137 |
| HYOGO UNIV HLTH SCI | 1 | 0.137 |
| ICORD | 1 | 0.137 |
| IDIBAPS | 1 | 0.137 |
| ILLINOIS STATE UNIVERSITY | 1 | 0.137 |
| INONU UNIVERSITY | 1 | 0.137 |
| INOVA FAIRFAX HOSPITAL | 1 | 0.137 |
| INOVA HEALTH SYSTEM | 1 | 0.137 |
| INST CANC ESTADO SAO PAULO | 1 | 0.137 |
| INST CLIN OUTCOMES RES | 1 | 0.137 |
| INST GUTTMAN | 1 | 0.137 |
| INST GUTTMANN | 1 | 0.137 |
| INST NEUROPATH PAIN | 1 | 0.137 |
| INST UNIV NEUROREHABILITACIO ADSCRIT UAB | 1 | 0.137 |
| INSTITUT D INVESTIGACIO SANITARIA PERE VIRGILI IISPV | 1 | 0.137 |
| INSTITUT NATIONAL POLYTECHNIQUE DE GRENOBLE | 1 | 0.137 |
| INSTITUTE OF METAL RESEARCH CAS | 1 | 0.137 |
| INT CLIN RES INST | 1 | 0.137 |
| INTERDISCIPLINARY CTR RES REHABIL SOCIAL INTEGR | 1 | 0.137 |
| IRCCS BAMBINO GESU | 1 | 0.137 |
| IRCCS CA GRANDA OSPEDALE MAGGIORE POLICLINICO | 1 | 0.137 |
| IRCCS CTR NEUROLESI MESSINA | 1 | 0.137 |
| IRCCS EUGENIO MEDEA | 1 | 0.137 |
| IRCCS FONDAZIONE CASIMIRO MONDINO | 1 | 0.137 |
| IRCCS FONDAZIONE DON CARLO GNOCCHI ONLUS | 1 | 0.137 |
| IRCCS FONDAZIONE SALVATORE MAUGERI | 1 | 0.137 |
| IRCCS ISTITUTO DELLE SCIENZE NEUROLOGICHE DI BOLOGNA ISNB | 1 | 0.137 |
| IRCCS ISTITUTO ORTOPEDICO RIZZOLI | 1 | 0.137 |
| IRONWOOD PHARMACEUT INC | 1 | 0.137 |
| IST SUPER OSTEOPATIA | 1 | 0.137 |
| ITHACA COLL | 1 | 0.137 |
| JACKSON MEM REHABIL CTR | 1 | 0.137 |
| JAMES A HALEY VETERANS HOSPITAL | 1 | 0.137 |
| JAPAN SOCIETY FOR THE PROMOTION OF SCIENCE | 1 | 0.137 |
| JAWAHARLAL NEHRU UNIVERSITY NEW DELHI | 1 | 0.137 |
| JERSEY MED SCH | 1 | 0.137 |
| JERUSALEM MENTAL HLTH CTR | 1 | 0.137 |
| JEWISH HOME HOSP | 1 | 0.137 |
| JIANGSU INST ANESTHESIOL | 1 | 0.137 |
| JIANGXI PROV KEY LAB AUTON NERVOUS FUNCT DIS | 1 | 0.137 |
| JINAN FIRST PEOPLES HOSP | 1 | 0.137 |
| JK STAT DATA CONSULTANCY | 1 | 0.137 |
| JMERC | 1 | 0.137 |
| JOHN CURTIN SCHOOL OF MEDICAL RESEARCH | 1 | 0.137 |
| JOHN D DINGELL VA MED CTR | 1 | 0.137 |
| JOHN H STROGER JUNIOR HOSPITAL COOK COUNTY | 1 | 0.137 |
| JOHNS HOPKINS MEDICINE | 1 | 0.137 |
| JOHNSON JOHNSON | 1 | 0.137 |
| JOHNSON JOHNSON USA | 1 | 0.137 |
| KAGOSHIMA UNIVERSITY | 1 | 0.137 |
| KEESLER INST REHABIL | 1 | 0.137 |
| KESSLER ADVENTIST REHABIL SERV | 1 | 0.137 |
| KHATAM OL ANBIA HOSP | 1 | 0.137 |
| KHON KAEN UNIVERSITY | 1 | 0.137 |
| KIO UNIV | 1 | 0.137 |
| KIRISHIMA ORTHOPED | 1 | 0.137 |
| KIRKLIN CLIN | 1 | 0.137 |
| KONYA NUMUNE HOSPITAL | 1 | 0.137 |
| KOREA INSTITUTE OF ORIENTAL MEDICINE KIOM | 1 | 0.137 |
| KOREA INSTITUTE OF SCIENCE TECHNOLOGY KIST | 1 | 0.137 |
| KUNMING MEDICAL UNIVERSITY | 1 | 0.137 |
| KURUME UNIVERSITY | 1 | 0.137 |
| KUSHIRO ROSAI HOSP | 1 | 0.137 |
| KYOTO UNIVERSITY | 1 | 0.137 |
| KYUNGPOOK NATIONAL UNIVERSITY | 1 | 0.137 |
| LAHEY HOSPITAL MEDICAL CENTER | 1 | 0.137 |
| LIVERPOOL JOHN MOORES UNIVERSITY | 1 | 0.137 |
| LOIS POPE LIFE CTR | 1 | 0.137 |
| LOMA LINDA UNIVERSITY | 1 | 0.137 |
| LUCERNE CANTONAL HOSPITAL | 1 | 0.137 |
| MACKAY MEDICAL COLLEGE | 1 | 0.137 |
| MACQUARIE UNIVERSITY | 1 | 0.137 |
| MAHARISHI MARKANDESHWAR UNIVERSITY | 1 | 0.137 |
| MARQUETTE UNIVERSITY | 1 | 0.137 |
| MARUISHI PHARMACEUT CO LTD | 1 | 0.137 |
| MAX PLANCK SOCIETY | 1 | 0.137 |
| MCMASTER UNIVERSITY | 1 | 0.137 |
| MED COLL TAISHAN | 1 | 0.137 |
| MED CTR | 1 | 0.137 |
| MED HOSP BEIBEI DIST | 1 | 0.137 |
| MEDICAL CENTER OF ALKMAAR | 1 | 0.137 |
| MEDSURANT LLC | 1 | 0.137 |
| MEDVAMC 145 | 1 | 0.137 |
| MEDVAMC 153 | 1 | 0.137 |
| MEHMET AKIF ERSOY UNIVERSITY | 1 | 0.137 |
| MERCK COMPANY | 1 | 0.137 |
| MIAMI DEPT VET AFFAIRS MED CTR | 1 | 0.137 |
| MIAMI MED CTR | 1 | 0.137 |
| MIAMI PROJECT CURE PARALYSIS | 1 | 0.137 |
| MIAMI PROJECT CURE PARALYSIS R 48 | 1 | 0.137 |
| MIAMI PROJECT CURE PARALYSIS R48 | 1 | 0.137 |
| MIAMI UNIV | 1 | 0.137 |
| MIL MED ACAD | 1 | 0.137 |
| MILLENNIUM PAIN CTR | 1 | 0.137 |
| MINISTRY OF HEALTH TURKEY | 1 | 0.137 |
| MINNEAPOLIS VA HEALTH CARE SYSTEM | 1 | 0.137 |
| MODEL SPINAL CORD INJURY CARE SYST SO CALIF | 1 | 0.137 |
| MONTECATONE INST NEUROREHABIL | 1 | 0.137 |
| MONTECATONE REHABIL INST | 1 | 0.137 |
| MOSSREHAB | 1 | 0.137 |
| MUDANJIANG MEDICAL UNIVERSITY | 1 | 0.137 |
| NANCHANG UNIVERSITY | 1 | 0.137 |
| NANTONG HOSP TRADIT CHINESE MED | 1 | 0.137 |
| NARA PREFECTURE GEN REHABIL CTR | 1 | 0.137 |
| NATIONAL INSTITUTE FOR PHYSIOLOGICAL SCIENCES NIPS | 1 | 0.137 |
| NATIONAL INSTITUTE OF HEALTH SCIENCES JAPAN | 1 | 0.137 |
| NATIONAL INSTITUTE OF MENTAL HEALTH NEUROSCIENCES INDIA | 1 | 0.137 |
| NATIONAL INSTITUTES OF NATURAL SCIENCES NINS JAPAN | 1 | 0.137 |
| NATIONAL TAIPEI UNIVERSITY OF NURSING HEALTH SCIENCE NTUNHS | 1 | 0.137 |
| NATIONAL TAIWAN UNIVERSITY HOSPITAL | 1 | 0.137 |
| NATIONAL UNIVERSITY OF SINGAPORE | 1 | 0.137 |
| NATL REHABIL CTR | 1 | 0.137 |
| NED UNIVERSITY OF ENGINEERING TECHNOLOGY | 1 | 0.137 |
| NEURONA THERAPEUT | 1 | 0.137 |
| NEUROSCIENCE RESEARCH AUSTRALIA | 1 | 0.137 |
| NEUROSEARCH AS | 1 | 0.137 |
| NEUROSURG DAMME | 1 | 0.137 |
| NICOX RES INST | 1 | 0.137 |
| NIGDE STATE HOSPITAL | 1 | 0.137 |
| NIH CLINICAL CENTER CC | 1 | 0.137 |
| NIH EUNICE KENNEDY SHRIVER NATIONAL INSTITUTE OF CHILD HEALTH HUMAN DEVELOPMENT NICHD | 1 | 0.137 |
| NIPPON MEDICAL SCHOOL | 1 | 0.137 |
| NORTHEASTERN UNIVERSITY | 1 | 0.137 |
| NORTHERN TECHNICAL UNIVERSITY | 1 | 0.137 |
| NORTHWELL HEALTH | 1 | 0.137 |
| NOVA TECH EEG | 1 | 0.137 |
| NSW AGCY CLIN INNOVAT | 1 | 0.137 |
| OHU UNIV | 1 | 0.137 |
| ORTHOPED CLIN | 1 | 0.137 |
| OSPEDALE NIGUARDA CA GRANDA | 1 | 0.137 |
| PALM BEACH NEUROL CTR | 1 | 0.137 |
| PARAPLEG CTR | 1 | 0.137 |
| PARC CIENT BARCELONA | 1 | 0.137 |
| PARKER UNIV | 1 | 0.137 |
| PATIENT PHYS PARTNERSHIP | 1 | 0.137 |
| PAVLOV INSTITUTE OF PHYSIOLOGY RUSSIAN ACADEMY OF SCIENCES | 1 | 0.137 |
| PFIZER JAPAN | 1 | 0.137 |
| PHARMERIT INT | 1 | 0.137 |
| PIETRA LIGURE PUBL HOSP | 1 | 0.137 |
| POLYTECH INST LEIRIA | 1 | 0.137 |
| PRINCE WALES HOSP | 1 | 0.137 |
| PURDUE UNIV NORTHWEST | 1 | 0.137 |
| PURDUE UNIVERSITY | 1 | 0.137 |
| PURDUE UNIVERSITY SYSTEM | 1 | 0.137 |
| PUSAN NATIONAL UNIVERSITY | 1 | 0.137 |
| PUSAN NATIONAL UNIVERSITY HOSPITAL | 1 | 0.137 |
| QIDONG PEOPLES HOSP | 1 | 0.137 |
| QINGDAO UNIVERSITY | 1 | 0.137 |
| QUEEN ELIZABETH II HEALTH SCIENCES CENTRE | 1 | 0.137 |
| QUEEN MARY UNIVERSITY LONDON | 1 | 0.137 |
| RABIN MEDICAL CENTER | 1 | 0.137 |
| RADBOUD UNIVERSITY NIJMEGEN | 1 | 0.137 |
| RANCHO LOS AMIGOS MED CTR | 1 | 0.137 |
| RANCHO LOS AMIGOS NATL REHABIL CTR | 1 | 0.137 |
| RANCHO LOS AMIGOS REHABIL CTR | 1 | 0.137 |
| RED CROSS HOSP | 1 | 0.137 |
| REHAB STN STOCKHOLM | 1 | 0.137 |
| REHAB STN STOCKHOLM SPINALIS | 1 | 0.137 |
| REHABIL CTR AMSTERDAM | 1 | 0.137 |
| REHABIL CTR HET ROESSINGH | 1 | 0.137 |
| REHABIL CTR HOOGSTR | 1 | 0.137 |
| REHABIL CTR ROESSINGH | 1 | 0.137 |
| REHABIL ORTON | 1 | 0.137 |
| REHMAN MED COLL | 1 | 0.137 |
| RICK HANSEN INST | 1 | 0.137 |
| RKU UNIV REHABIL KLINIKEN ULM | 1 | 0.137 |
| ROYAL LONDON HOSPITAL | 1 | 0.137 |
| ROYAL PERTH HOSPITAL | 1 | 0.137 |
| ROYAL REHAB CTR | 1 | 0.137 |
| ROYAL REHABIL CTR | 1 | 0.137 |
| ROYAL REHABILITAT CTR | 1 | 0.137 |
| RUHR UNIVERSITY BOCHUM | 1 | 0.137 |
| RUSSIAN ACADEMY OF SCIENCES | 1 | 0.137 |
| RUTGERS NEW JERSEY SCH MED | 1 | 0.137 |
| RUTGERS STATE UNIVERSITY NEWARK | 1 | 0.137 |
| SABBATSBERGS HOSP | 1 | 0.137 |
| SACRAMENTO VA MED CTR | 1 | 0.137 |
| SAMOT | 1 | 0.137 |
| SANJAY GANDHI POSTGRADUATE INSTITUTE OF MEDICAL SCIENCES | 1 | 0.137 |
| SAPIENZA UNIVERSITY ROME | 1 | 0.137 |
| SARAH NETWORK REHABIL HOSP | 1 | 0.137 |
| SCH ENGN | 1 | 0.137 |
| SCH LANGUAGE COMMUN DEV | 1 | 0.137 |
| SCHULTHESS CLINIC | 1 | 0.137 |
| SCHWARZ BIOSCI | 1 | 0.137 |
| SCRIPPS RESEARCH INSTITUTE | 1 | 0.137 |
| SCUOLA SUPERIORE SANT ANNA | 1 | 0.137 |
| SENBOKU KUMIAI GEN HOSP | 1 | 0.137 |
| SESCAM | 1 | 0.137 |
| SEVKET YILMAZ TRAINING RESEARCH HOSPITAL | 1 | 0.137 |
| SHANDONG JINING 1 PEOPLES HOSP | 1 | 0.137 |
| SHANGHAI GONGLI HOSP | 1 | 0.137 |
| SHANGHAI JIAO TONG UNIVERSITY | 1 | 0.137 |
| SHANGHAI KAIYUAN ORTHOPED HOSP | 1 | 0.137 |
| SHANGHAI UNIVERSITY OF SPORT | 1 | 0.137 |
| SHIN KONG WU HO MEM HOSP | 1 | 0.137 |
| SICHUAN PROVINCIAL PEOPLE S HOSPITAL | 1 | 0.137 |
| SICHUAN UNIVERSITY | 1 | 0.137 |
| SIXTH MEDICAL CENTER OF CHINESE PLA GENERAL HOSPITAL | 1 | 0.137 |
| SLOTERVAART HOSPITAL | 1 | 0.137 |
| SLOVAK ACADEMY OF SCIENCES | 1 | 0.137 |
| SMS MEDICAL COLLEGE HOSPITAL | 1 | 0.137 |
| SOGANG UNIVERSITY | 1 | 0.137 |
| SONDALO PUBL HOSP | 1 | 0.137 |
| SOTERIX MED | 1 | 0.137 |
| SOUTH LONDON MAUDSLEY NHS TRUST | 1 | 0.137 |
| SPAULDING NEW ENGLAND REG SCI CTR | 1 | 0.137 |
| SPINALIS SCI UNIT | 1 | 0.137 |
| SRC REHABIL HOSP | 1 | 0.137 |
| ST JOSEPHS PARKWOOD HOSP | 1 | 0.137 |
| ST LUKES REHABIL INST | 1 | 0.137 |
| ST PETERSBURG SCIENTIFIC CENTRE OF THE RUSSIAN ACADEMY OF SCIENCES | 1 | 0.137 |
| STANFORD UNIVERSITY | 1 | 0.137 |
| STATE UNIVERSITY OF NEW YORK SUNY BUFFALO | 1 | 0.137 |
| STATE UNIVERSITY OF NEW YORK SUNY SYSTEM | 1 | 0.137 |
| STOCKHOLM UNIVERSITY | 1 | 0.137 |
| STOKE MANDEVILLE HOSP NHS TRUST | 1 | 0.137 |
| SUN YAT SEN UNIVERSITY | 1 | 0.137 |
| SUNNAAS REHABIL HOSP | 1 | 0.137 |
| SUVACARE | 1 | 0.137 |
| SW COLL NATUROPATH MED | 1 | 0.137 |
| SWEDISH SCHOOL OF SPORT HEALTH SCIENCES | 1 | 0.137 |
| SYRACUSE DVAMC | 1 | 0.137 |
| TAIZHOU PEOPLES HOSP | 1 | 0.137 |
| TANTA UNIVERSITY | 1 | 0.137 |
| TEIKYO UNIVERSITY | 1 | 0.137 |
| TEXAS A M HEALTH SCIENCE CENTER | 1 | 0.137 |
| TEXAS INST REHABIL RES | 1 | 0.137 |
| TEXAS STATE UNIVERSITY SAN MARCOS | 1 | 0.137 |
| TEXAS STATE UNIVERSITY SYSTEM | 1 | 0.137 |
| TEXAS WOMANS UNIVERSITY | 1 | 0.137 |
| TIRR | 1 | 0.137 |
| TIRR MEM HERMANN | 1 | 0.137 |
| TOHO UNIVERSITY | 1 | 0.137 |
| TOKYO MEDICAL DENTAL UNIVERSITY TMDU | 1 | 0.137 |
| TOKYO WOMEN S MEDICAL UNIVERSITY | 1 | 0.137 |
| TORONTO CONGENITAL CARDIAC CENTRE FOR ADULTS | 1 | 0.137 |
| TULANE UNIVERSITY | 1 | 0.137 |
| TURAN EMEKSIZ SOKAK | 1 | 0.137 |
| TURKISH ARMED FORCES REHABILITATION MAINTENANCE CENTER | 1 | 0.137 |
| UBC SCI SOLUT | 1 | 0.137 |
| UBON RATCHATHANI UNIVERSITY | 1 | 0.137 |
| ULM UNIVERSITY | 1 | 0.137 |
| ULUDAG UNIVERSITY | 1 | 0.137 |
| UMEA UNIVERSITY | 1 | 0.137 |
| UNIBRASIL | 1 | 0.137 |
| UNITED STATES ARMY | 1 | 0.137 |
| UNITED STATES DEPARTMENT OF DEFENSE | 1 | 0.137 |
| UNIV MED SCI | 1 | 0.137 |
| UNIVERSIDAD DE ANTIOQUIA | 1 | 0.137 |
| UNIVERSIDAD DE MALAGA | 1 | 0.137 |
| UNIVERSIDAD REY JUAN CARLOS | 1 | 0.137 |
| UNIVERSIDADE CIDADE DE SAO PAULO | 1 | 0.137 |
| UNIVERSIDADE FEDERAL DE SANTA CATARINA UFSC | 1 | 0.137 |
| UNIVERSIDADE FEDERAL DO CEARA | 1 | 0.137 |
| UNIVERSIDADE PORTUCALENSE INFANTE D HENRIQUE | 1 | 0.137 |
| UNIVERSIDADE PRESBITERIANA MACKENZIE | 1 | 0.137 |
| UNIVERSITAT ROVIRA I VIRGILI | 1 | 0.137 |
| UNIVERSITE DE PARIS | 1 | 0.137 |
| UNIVERSITE DE STRASBOURG | 1 | 0.137 |
| UNIVERSITE GRENOBLE ALPES UGA | 1 | 0.137 |
| UNIVERSITES DE STRASBOURG ETABLISSEMENTS ASSOCIES | 1 | 0.137 |
| UNIVERSITY HOSPITAL OF BERN | 1 | 0.137 |
| UNIVERSITY OF ABERDEEN | 1 | 0.137 |
| UNIVERSITY OF ADELAIDE | 1 | 0.137 |
| UNIVERSITY OF ANTWERP | 1 | 0.137 |
| UNIVERSITY OF BERN | 1 | 0.137 |
| UNIVERSITY OF BOLOGNA | 1 | 0.137 |
| UNIVERSITY OF CALIFORNIA DAVIS | 1 | 0.137 |
| UNIVERSITY OF CAMBRIDGE | 1 | 0.137 |
| UNIVERSITY OF COLORADO DENVER | 1 | 0.137 |
| UNIVERSITY OF DELAWARE | 1 | 0.137 |
| UNIVERSITY OF DUNDEE | 1 | 0.137 |
| UNIVERSITY OF GENOA | 1 | 0.137 |
| UNIVERSITY OF GOTTINGEN | 1 | 0.137 |
| UNIVERSITY OF HELSINKI | 1 | 0.137 |
| UNIVERSITY OF HONG KONG | 1 | 0.137 |
| UNIVERSITY OF ILLINOIS CHICAGO | 1 | 0.137 |
| UNIVERSITY OF ILLINOIS CHICAGO HOSPITAL | 1 | 0.137 |
| UNIVERSITY OF ILLINOIS SYSTEM | 1 | 0.137 |
| UNIVERSITY OF JINAN | 1 | 0.137 |
| UNIVERSITY OF LAUSANNE | 1 | 0.137 |
| UNIVERSITY OF LIEGE | 1 | 0.137 |
| UNIVERSITY OF LOUISVILLE | 1 | 0.137 |
| UNIVERSITY OF MILAN | 1 | 0.137 |
| UNIVERSITY OF MISSOURI COLUMBIA | 1 | 0.137 |
| UNIVERSITY OF MONTREAL | 1 | 0.137 |
| UNIVERSITY OF NOTTINGHAM | 1 | 0.137 |
| UNIVERSITY OF OTAGO | 1 | 0.137 |
| UNIVERSITY OF OTTAWA | 1 | 0.137 |
| UNIVERSITY OF PERUGIA | 1 | 0.137 |
| UNIVERSITY OF PISA | 1 | 0.137 |
| UNIVERSITY OF PUERTO RICO | 1 | 0.137 |
| UNIVERSITY OF PUERTO RICO MEDICAL SCIENCES CAMPUS | 1 | 0.137 |
| UNIVERSITY OF ROCHESTER | 1 | 0.137 |
| UNIVERSITY OF SANTO TOMAS | 1 | 0.137 |
| UNIVERSITY OF SHERBROOKE | 1 | 0.137 |
| UNIVERSITY OF SOUTH AUSTRALIA | 1 | 0.137 |
| UNIVERSITY OF SOUTH CHINA | 1 | 0.137 |
| UNIVERSITY OF SOUTHAMPTON | 1 | 0.137 |
| UNIVERSITY OF STRATHCLYDE | 1 | 0.137 |
| UNIVERSITY OF TEHRAN | 1 | 0.137 |
| UNIVERSITY OF TEXAS AUSTIN | 1 | 0.137 |
| UNIVERSITY OF TEXAS HEALTH SAN ANTONIO | 1 | 0.137 |
| UNIVERSITY OF TOKYO | 1 | 0.137 |
| UNIVERSITY OF TURKU | 1 | 0.137 |
| UNIVERSITY OF UTAH | 1 | 0.137 |
| UNIVERSITY OF WATERLOO | 1 | 0.137 |
| UNIVERSITY OF WESTERN AUSTRALIA | 1 | 0.137 |
| UNIVERSITY SYSTEM OF GEORGIA | 1 | 0.137 |
| US ARMY | 1 | 0.137 |
| UT HLTH | 1 | 0.137 |
| UTAH SYSTEM OF HIGHER EDUCATION | 1 | 0.137 |
| VA LOMA LINDA HEALTHCARE SYST | 1 | 0.137 |
| VA PALO ALTO HEALTH CARE SYSTEM | 1 | 0.137 |
| VALDOSTA STATE UNIVERSITY | 1 | 0.137 |
| VANDERBILT UNIVERSITY | 1 | 0.137 |
| VET ADM | 1 | 0.137 |
| VET ADM MED CTR W ROXBURY | 1 | 0.137 |
| VICTOR BABES UNIVERSITY OF MEDICINE PHARMACY TIMISOARA | 1 | 0.137 |
| VIRGINAIA CONNECTICUT HEALTHCARE SYST | 1 | 0.137 |
| VIRGINIA CONNECTICUT HEALTHCARE SYST | 1 | 0.137 |
| VU UNIVERSITY MEDICAL CENTER | 1 | 0.137 |
| WAKAYAMA MEDICAL UNIVERSITY | 1 | 0.137 |
| WAKE FOREST BAPTIST MEDICAL CENTER | 1 | 0.137 |
| WAKE FOREST UNIVERSITY | 1 | 0.137 |
| WALTER REED NATIONAL MILITARY MEDICAL CENTER | 1 | 0.137 |
| WALTON CENTRE | 1 | 0.137 |
| WASHINGTON STATE UNIVERSITY | 1 | 0.137 |
| WERNER WICKER CLIN | 1 | 0.137 |
| WESTMEAD INSTITUTE FOR MEDICAL RESEARCH | 1 | 0.137 |
| WILLIAM PATERSON UNIV | 1 | 0.137 |
| WOODROW WILSON REHABIL CTR | 1 | 0.137 |
| WUHAN UNIVERSITY | 1 | 0.137 |
| XUZHOU MEDICAL COLLEGE | 1 | 0.137 |
| YAMAGUCHI UNIVERSITY | 1 | 0.137 |
| YORK UNIVERSITY CANADA | 1 | 0.137 |
| ZHENGZHOU ORTHOPAED HOSP | 1 | 0.137 |
| ZONMW | 1 | 0.137 |
